# The Magnetic Genome of Two-Dimensional van der Waals Materials

**DOI:** 10.1021/acsnano.1c09150

**Published:** 2022-04-20

**Authors:** Qing Hua Wang, Amilcar Bedoya-Pinto, Mark Blei, Avalon H. Dismukes, Assaf Hamo, Sarah Jenkins, Maciej Koperski, Yu Liu, Qi-Chao Sun, Evan J. Telford, Hyun Ho Kim, Mathias Augustin, Uri Vool, Jia-Xin Yin, Lu Hua Li, Alexey Falin, Cory R. Dean, Fèlix Casanova, Richard F. L. Evans, Mairbek Chshiev, Artem Mishchenko, Cedomir Petrovic, Rui He, Liuyan Zhao, Adam W. Tsen, Brian D. Gerardot, Mauro Brotons-Gisbert, Zurab Guguchia, Xavier Roy, Sefaattin Tongay, Ziwei Wang, M. Zahid Hasan, Joerg Wrachtrup, Amir Yacoby, Albert Fert, Stuart Parkin, Kostya S. Novoselov, Pengcheng Dai, Luis Balicas, Elton J. G. Santos

**Affiliations:** 1 Materials Science and Engineering, School for Engineering of Matter, Transport and Energy, Arizona State University, Tempe, Arizona 85287, United States; 2 NISE Department, Max Planck Institute of Microstructure Physics, 06120 Halle, Germany; 3 Department of Chemistry, Columbia University, New York, New York 10027, United States; 4 Department of Physics, Harvard University, Cambridge, Massachusetts 02138, United States; 5 Twist Group, Faculty of Physics, University of Duisburg-Essen, Campus Duisburg, 47057 Duisburg, Germany; 6 Institute for Functional Intelligent Materials, National University of Singapore, 117544 Singapore; 7 Condensed Matter Physics and Materials Science Department, Brookhaven National Laboratory, Upton, New York 11973, United States; 8 Physikalisches Institut, University of Stuttgart, 70569 Stuttgart, Germany; 9 Department of Physics, Columbia University, New York, New York 10027, United States; 10 School of Materials Science and Engineering, Department of Energy Engineering Convergence, Kumoh National Institute of Technology, Gumi 39177, Korea; 11 Institute for Condensed Matter Physics and Complex Systems, School of Physics and Astronomy, The University of Edinburgh, Edinburgh, EH9 3FD, United Kingdom; 12 Donostia International Physics Center (DIPC), 20018 Donostia-San Sebastián, Basque Country, Spain; 13 John Harvard Distinguished Science Fellows Program, Harvard University, Cambridge, Massachusetts 02138, United States; 14 Laboratory for Topological Quantum Matter and Spectroscopy, Department of Physics, Princeton University, Princeton, New Jersey 08544, United States; 15 Institute for Frontier Materials, Deakin University, Geelong Waurn Ponds Campus, Waurn Ponds, Victoria 3216, Australia; 16 Department of Physics, Columbia University, New York, New York 10027, United States; 17 CIC nanoGUNE BRTA, 20018 Donostia - San Sebastián, Basque Country, Spain; 18 IKERBASQUE, Basque Foundation for Science, 48013 Bilbao, Basque Country, Spain; 19 Department of Physics, University of York, Heslington, York YO10 5DD, United Kingdom; 20 Université Grenoble Alpes, CEA, CNRS, Spintec, 38000 Grenoble, France; 21 Institut Universitaire de France, 75231 Paris, France; 22 Department of Physics and Astronomy, University of Manchester, Manchester, M13 9PL, United Kingdom; 23 National Graphene Institute, University of Manchester, Manchester, M13 9PL, United Kingdom; 24 Department of Electrical and Computer Engineering, Texas Tech University, 910 Boston Avenue, Lubbock, Texas 79409, United States; 25 Department of Physics, University of Michigan, 450 Church Street, Ann Arbor, Michigan 48109, United States; 26 Institute for Quantum Computing and Department of Chemistry, University of Waterloo, Waterloo, Ontario N2L 3G1, Canada; 27 SUPA, Institute of Photonics and Quantum Sciences, Heriot-Watt University, Edinburgh EH14 4AS, United Kingdom; 28 Department of Chemistry, Columbia University, New York, New York 10027, United States; 29 Laboratory for Muon Spin Spectroscopy, Paul Scherrer Institute, CH-5232 Villigen PSI, Switzerland; 31 Materials Sciences Division, Lawrence Berkeley National Laboratory, Berkeley, California 94720, United States; 32 Princeton Institute for Science and Technology of Materials, Princeton University, Princeton, New Jersey 08544, United States; 33 Max Planck Institute for Solid State Research, 70569 Stuttgart, Germany; 34 John A. Paulson School of Engineering and Applied Sciences, Harvard University, Cambridge, Massachusetts 02138, United States; 35 Unité Mixte de Physique, CNRS, Thales, Université Paris-Saclay, 91767 Palaiseau, France; 36 Department of Materials Physics UPV/EHU, 20018 Donostia - San Sebastián, Basque Country, Spain; 38 Department of Physics and Astronomy, Rice University, Houston, Texas 77005, United States; 39 National High Magnetic Field Laboratory, Florida State University, Tallahassee, Florida 32310, United States; 40 Department of Physics, Florida State University, Tallahassee, Florida 32306, United States; 41 Higgs Centre for Theoretical Physics, The University of Edinburgh, Edinburgh EH9 3FD, United Kingdom; 42 Instituto de Ciencia Molecular (ICMol), Universitat de València, 46980 Paterna, Spain

**Keywords:** 2D magnetic materials, van der Waals, CrI_3_, magneto-optical effect, neutron scattering, Fe_3_GeTe_2_, magnetic genome, atomistic spin dynamics

## Abstract

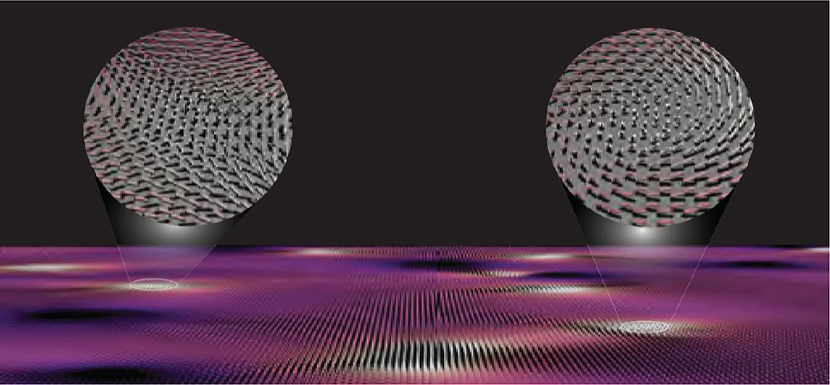

Magnetism in two-dimensional (2D) van der Waals (vdW) materials has recently emerged as one of the most promising areas in condensed matter research, with many exciting emerging properties and significant potential for applications ranging from topological magnonics to low-power spintronics, quantum computing, and optical communications. In the brief time after their discovery, 2D magnets have blossomed into a rich area for investigation, where fundamental concepts in magnetism are challenged by the behavior of spins that can develop at the single layer limit. However, much effort is still needed in multiple fronts before 2D magnets can be routinely used for practical implementations. In this comprehensive review, prominent authors with expertise in complementary fields of 2D magnetism (*i.e.*, synthesis, device engineering, magneto-optics, imaging, transport, mechanics, spin excitations, and theory and simulations) have joined together to provide a genome of current knowledge and a guideline for future developments in 2D magnetic materials research.

Two-dimensional (2D) materials have been the focus of intense and extensive research efforts around the world for the better of the past two decades, starting from the discovery of graphene and then rapidly expanding to an enormous variety of materials and properties. One of the most exciting recent developments in 2D materials has been the discovery of intrinsic long-range magnetic order in atomically thin layers. In Figure 1, we provide a timeline of the major discoveries in 2D magnets over the past few years. In a fairly short period of time, there have been significant advances in our knowledge of magnetic 2D materials, detailed characterization of magnetic states, and progress toward magnetic and spintronic devices with exceptional performance.

**Figure 1:**
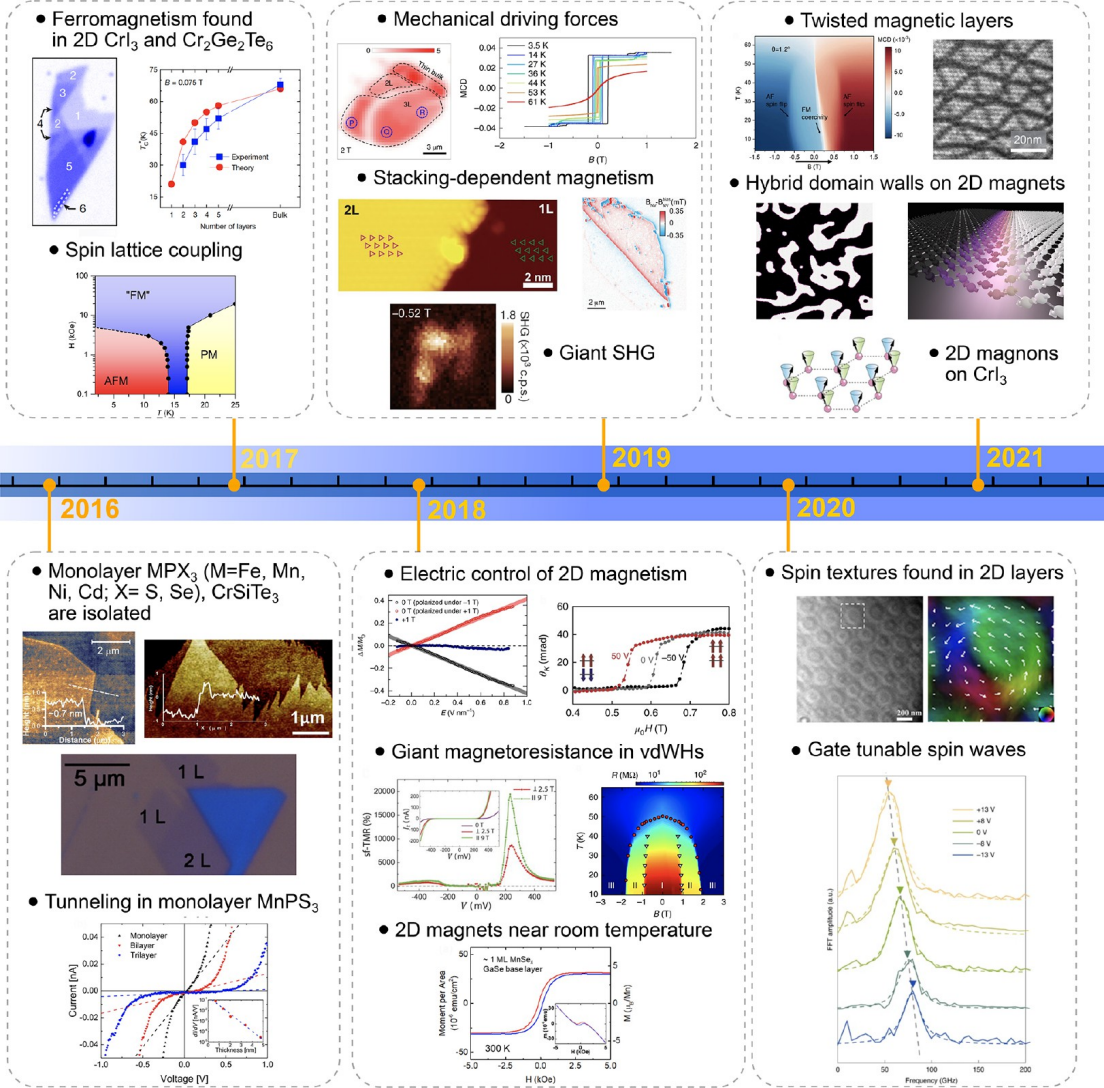
Timeline of developments in 2D magnets. Since early 2016, a few results on monolayer phosphides MPX_3_ (M = Fe, Mn, Ni, Cd; X = S, Se)^1,2^ and CrSiTe_3_^3^ appeared in the literature, with results on electron tunneling in MnPS_3_ also being reported.^4^ The conclusive measurements in 2017 of magnetism on CrI_3_^5^ and Cr_2_Ge_2_Te_6_^6^ sparked an increasing interest in several subjects involving magnetism in 2D. Results on spin–lattice coupling collected from CrCl_3_^7^ also provided different mechanisms involving vibrations and spins in 2D. In 2018, the electric control of magnetism,^8−12^ giant magnetoresistance,^13−16^ and a potential 2D magnet (*i.e.*, VSe_2_) displaying room-temperature magnetism^17−19^ attracted substantial interest in the community. In 2019, experimental evidence of stacking-dependent magnetic properties,^20,21^ pressure effects,^22,23^ and giant second-harmonic generation (SHG)^24^ drove the field toward intriguing magnetic properties. In 2020, spin-textures^25−27^ such as skyrmions, spirals, and spin-waves^28^ indicate that topologically nontrivial spins are a reality on 2D magnets. In 2021, a few reports on twisted magnetic layers,^29,30^ together with the hybrid character of narrow domain-walls^31^ on CrI_3_, raised possibilities for the angular control of magnetic features and domain-wall based applications (*i.e.*, racetrack). All images adapted from the references cited above with permission as follows. Panels from (2016) reprinted with permission from ref (32), copyright 2016 American Chemical Society; ref (3), copyright 2016 Royal Society of Chemistry; ref (1), copyright 2016 American Chemical Society; and ref (4), copyright 2016 AIP Publishing and reprinted with permission under a Creative Commons Attribution (CC BY) license. Panels from (2017) reprinted with permission from ref (5), copyright 2017 Springer Nature; ref (6), copyright 2017 Springer Nature; and ref (7), copyright 2017 American Physical Society. Panels from (2018) reprinted with permission from ref (8), copyright 2018 Springer Nature; ref (9), copyright 2018 Springer Nature; ref (13), copyright 2018 AAAS; with permission under a Creative Commons CC by 4.0 license from ref (15), copyright 2018 Springer Nature; and ref (17), copyright 2018 American Chemical Society. Panels from (2019) reprinted with permission from ref (22), copyright 2019 Springer Nature; ref (23), copyright 2019 Springer Nature; ref (20), copyright 2019 AAAS; ref (21), copyright 2019 AAAS; and ref (24), copyright 2019 Springer Nature. Panels from (2020) reprinted with permission from ref (25), copyright 2020 American Chemical Society; and ref (28), copyright 2020 Springer Nature. Panels from (2021) reprinted with permission from ref (29), copyright 2021 Springer Nature; ref (30), copyright 2021 Springer Nature; and ref (31), copyright 2021 John Wiley and Sons.

The purpose of this review is to assemble a thorough genome of all aspects of 2D magnetic van der Waals (vdW) materials, and to provide a guideline for future directions. Throughout this work, we have drawn on the expertise of many key researchers in this exciting emerging field to summarize their most critical findings to date and to lay out the important upcoming challenges.

This review article is divided into the following sections:

- Historical perspective: An overview of low-dimensional materials, models of how magnetic moments interact, and summary of key recent discoveries and developments in 2D magnets.
- Device engineering: The main types of magnetic devices, *e.g.*, magnetic tunnel junctions (MTJs), magnetoresistant lateral transport devices, and spin waves in tunnelling devices.
- Magneto-optical phenomena: Characterization and study of 2D magnets by several magneto-optical spectroscopy methods including Kerr effect, circular dichroism, magneto-photoluminescence (PL), and magneto-Raman spectroscopy which can reveal spin-phonon effects.
- Magnetic imaging: Magnetic force microscopy (MFM), nitrogen-vacancy center magnetometry (NV-center), nanosuperconducting quantum interference device (nanoSQUID), spin-polarized scanning transmission microscopy (SP-STM), and Lorentz transmission electron microscopy (Lorentz TEM or LTEM) are used to image magnetic domain features in 2D magnets.
- Magnetic and electrical transport characterization: Magnetic critical behavior, magnetocaloric effect, magnetism in bulk and thin-layer vdW magnets of different electronic properties (*i.e.*, insulator, metallic, semiconductor) *via* different techniques.
- The role of defects and vacancies: Muon spin rotation (μSR) methods are used to investigate microscopic magnetic properties in the presence of defects.
- Spintronics from fundamentals to devices: Basic magnetic properties of typical 2D magnets are described, followed by spintronic implementations and memory devices.
- Magnetic-topological phases: Topological materials and transition-metal-based kagome lattice family of materials exhibit ferromagnetism and anomalous magnetization effects.
- Synthesis and sample preparation: Methods of preparing samples of 2D magnets by top-down and bottom-up methods.
- Mechanical properties and strain engineering: Description of mechanical properties 2D magnets, along with theoretical predictions, and how strain can induce further magnetic properties.
- Spin excitations, topological properties: Measurement of collective excitations of electron spin states by neutron scattering, inelastic electron tunneling spectroscopy, and Raman spectroscopy.
- Heterostructures, twisted layers, and interfaces: Stacking different 2D magnets together, generating exotic quantum phases, and how to integrate 2D magnets into broader device architectures.
- Theory and simulations: Description of the underling theory, computational method and spin models to investigate 2D magnetic materials.
- Perspectives and a forward-looking approach: The final key section is an overview of the major challenges and opportunities in the field and what we can expect research directions to focus on in the coming few years.

## Historical Perspective

Since the 1970s, low-dimensional (low-*d*) physics has grown and matured into a major branch of science. In general, one can define a system with restricted dimensionality *d* as an object that is infinite only in one or two spatial directions (*d* = 1 and 2). Such a definition comprises, for example, isolated single chains or layers, in addition to fibers and thin layers (films) of varying but finite thicknesses. A multitude of physical phenomena, notably in solid-state physics, fall into the category of a low-*d* system. The study of such systems substantially advanced our fundamental knowledge of physics, but also led to relevant technological applications. No matter how widely different the many low-*d* physical systems may appear at first glance, there are several similarities and characteristics that they all share, which are inherent to their reduced dimensionality. It is for this very reason that one may consider low-*d* physics as a recognizable field of science on its own right. Within this larger field, the sub-branch of low-*d* magnetism played a quite important role from the outset. For example, theoretical work on magnetic chains and layers started as early as the twenties with the Ising chain,^33^ followed by studies of 2-*d* Ising magnets in the 1930s and 1940s.^34^ Systematic experimental studies on quasi low-*d* magnetic systems started in the 1940s and 1950s, but ultrathin magnetic films composed of just a few monolayers were studied in more recent past. These systems should be contrasted with the magnetic films used in thin-film technology, which have thicknesses ranging from 10^3^ to 10^4^ atomic layers.

Perhaps, one of the most important contributions of low-*d* magnetism to fundamental physics is in the subject of phase transitions and critical phenomena. The way in which cooperative phenomena are influenced by the crystal dimensionality, the symmetry of the interactions in the Hamiltonian, or the quantum-mechanical nature of the spin, led to such important concepts such as scaling, universality classes (models having the same set of critical exponents), and the renormalization group.^35^ This general theory of critical phenomena based upon the principles of quantum-field theory led to the Nobel prize granted to K. G. Wilson and is considered as a great advance in mathematical physics.^35^ Phase transitions are a very common phenomena that have a wide range of applicability, which explains the past interest in the study of low-*d* magnets. Most physical problems involving interacting elements that form a spatial array can be mapped into a magnetic language by describing the problem within a pseudospin formalism (*e.g.*, structural or electric phase transitions).

Another unifying concept emerging from low-*d* magnetism is that of nonlinear excitations or domain walls such as solitons or kinks; excitations that can either be static (and topological) or dynamic. These occur when the ground state of the system is degenerate, as is the case for an Ising ferromagnet where the spin-up and -down ground states are distinct but have the same energy. Here, low-*d* magnets provided some of the simplest experimental systems to study these physical phenomena having very broad applicability. For instance, the absence of long-range magnetic order in an Ising ferromagnetic (FM) chain becomes immediately clear when one realizes that this system is unstable with respect to thermal excitations such as kinks, which are an effective means of destroying long-range correlations. For *d* ∼ 2 the analogue of the kink in Ising systems is the boundary around a droplet created, for example, by a fraction of up spins in a background sea of down spins. In summary, low-*d* magnetism has been characterized by a long-standing, strong interaction between theory and experiments, with both developing in parallel but with a continuous cross fertilization.

### Historical Models of Interacting Magnetic Moments

#### The Ising Model

The Ising model corresponds to an ideal lattice of identical magnetic moments positioned at all lattice sites. For each elementary moment, or spin, only two states are possible (spin “up” and “down”). The lattice site *r* is associated with a variable σ_*r*_ whose two values ±1 corresponding to both spin orientations. The Hamiltonian:
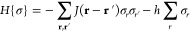
1is attributed to a fixed configuration of spins {σ}. Usually, only the nearest-neighbor interaction is considered, or *J*(*r*) = 0 for all **r** ≠ **a**_**i**_, where the **a**_**i**_’s are the basic vectors of a lattice. This is the simplest model for a highly anisotropic magnet. The *J*(*r*) are the exchange integrals and *h* is the magnetic field (in units of *gμ*_*B*_). In the simplest scenario, the nonzero quantities *J*(*r*) are assumed to be independent of the direction of *r*. For *h* = 0, the Hamiltonian is invariant with respect to a change in the sign of the spins. This transformation together with its identical one forms the symmetry group *Z*_2_ for the Ising model. The Ising model was proposed by Lenz,^36^ but Ising^33^ presented the exact calculation of the partition function for the 1 – *d* case. A great success was achieved by Onsager,^34^ who calculated the partition function for the 2 – *d* case. Onsager demonstrated the existence of the second-order phase transition, and this stimulated intense subsequent theoretical and experimental investigations on critical phenomena.

#### The XY Model

Magnets with an easy plane of magnetic anisotropy can be described by the XY model which corresponds to a lattice of classical spins rotating in a plane and interacting with each other *via* the Hamiltonian:
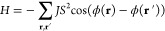
2

The spins are fixed with respect to their angles of rotation ϕ(*r*). Their value *S* is assumed to be constant. The summation in eq 2 proceeds over the nearest-neighbor sites of the lattice. *J* is the exchange constant. The ground state is FM for *J* > 0 on an arbitrary lattice. In the opposite case *J* < 0, the ground state is antiferromagnetic (AF) if the lattice can be decomposed into two sublattices with the nearest-neighbor spins belonging to each sub-lattice. In the case of the triangular lattice with *J* < 0 the ground state consists of three sublattices with the spins in the different sublattices tilted at angles of ±120°. An external magnetic field fixes the orientation of the spins in ferromagnets and in antiferromagnets with two sublattices. In the last scenario, the spins are directed almost perpendicularly to the field direction. In the case of a three-sublattice antiferromagnet, the external magnetic field does not interfere with the freedom of spin rotation inherent to the ground state. The simplest excitations of a planar magnet, as described through the XY model, are spin-waves with a gapless spectrum, according to the Goldstone theorem. The static aspect of the Goldstone theorem implies an enhancement of the spin fluctuations at long wavelength, and this enhancement is precisely the starting point in the proof of the Landau-Peierls theorem^37,38^ stating that there is no long-range order in 2-*d* systems having a continuous symmetry. As discussed below, a rigorous proof for this theorem was given by Hohenberg^39^ for superfluids which have the same symmetry as a planar magnet, and by Mermin and Wagner^40^ for Heisenberg magnets. These proofs are based on the thermodynamic inequalities proposed by Bogolyubov.^41^

#### The Heisenberg Model

**Figure 2:**
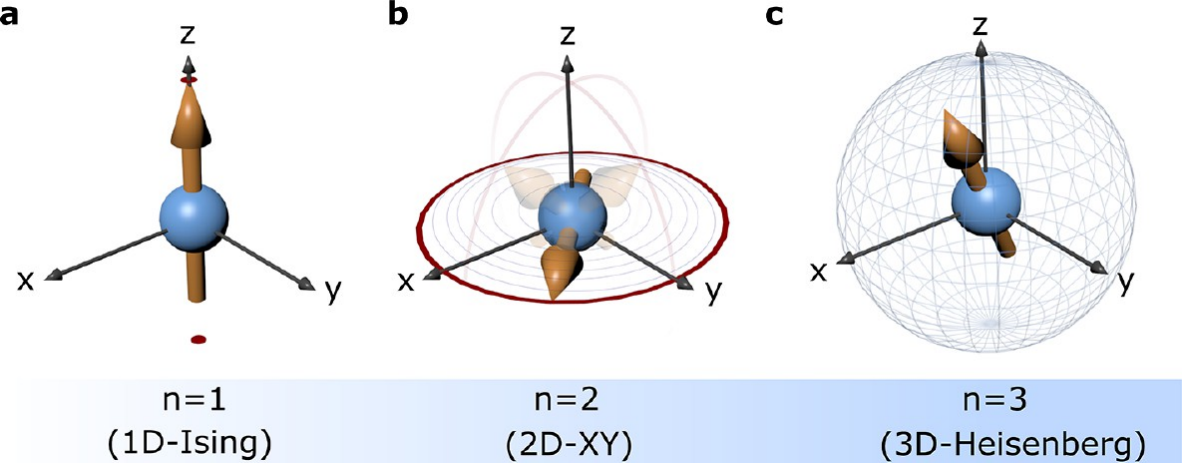
Role of spin dimensionality *n* on the 3D-axis. (a) *n* = 1; 1D Ising type, where spins point in either up or down along a given direction (*e.g.*, easy axis). (b) *n* = 2; 2D XY type, where spins are constrained to a given plane (easy-plane anisotropy) without any restriction on which plane (*e.g.*, XY, XZ, YZ). (c) *n* = 3; 3D isotropic Heisenberg type, where spins have no constraints on the direction assuming any position along the 3D sphere.

In the subsequent text, the symbol “*d*” is used for the dimensionality of the magnetic lattice, *d* = 1, 2, 3, corresponding to magnetic chains, magnetic layers, and three-dimensional (3D) arrays of magnetic moments, respectively. These three classes of magnetic systems may be further subdivided according to the type of magnetic interactions assumed to exist between the spins. If one restricts oneself only to interactions between nearest neighbors, one can write a generalized Heisenberg Hamiltonian that captures the essence of the several other historical models discussed below:
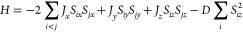
3where the *J*s are the exchange couplings between spins *S*_*i*_ and *S*_*j*_ on neighboring sites (with either FM, *i.e.*, *J* > 0, or AF, *J* < 0, order), while *J*_*z*_ and *D* can, for a 2*d* system, be considered as the “on-site” and the “inter-site” magnetic anisotropies, respectively. In addition, depending on the number *n* of components (*x*, *y*, *z*) of the individual spins that are being considered, one has a one, two, or three component spin system. *n* is the spin-dimensionality (Figure 2), not to be confused with the lattice-dimensionality *d*. For a given *n*(>1), and for *D* = 0, one may in addition vary the number of interacting spin components by considering different combinations of *J*_*x*_, *J*_*y*_, and *J*_*z*_ as illustrated in Table 1.

**Table 1:**
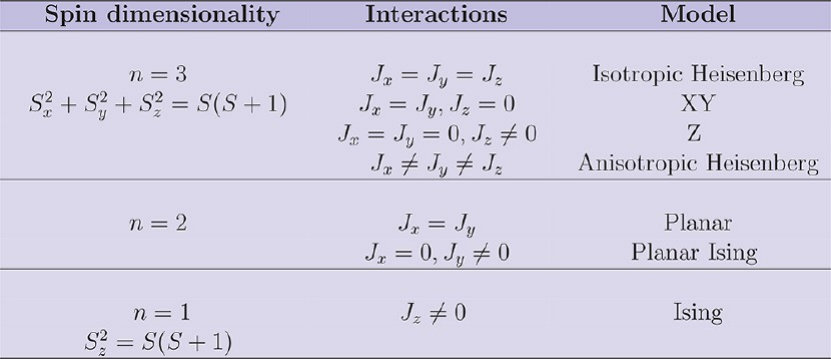
Classification of model systems based on single-neighbor Heisenberg Hamiltonian including inter-site magnetic anisotropy^a^

a Adapted with permission from ref (42). Copyright 1990 Springer.

This Hamiltonian can be further generalized by adding terms that describe exchange couplings with second, third, and more neighbors. The spin itself can also be varied by including quantum mechanical operators (*S* = 1/2, 1, 3/2, *etc*.) or classical spins (*S* = *∞*).

Table 2 below conveys how past theoretical and experimental investigations on phase transitions revealed how the lattice-dimensionality *d* and the spin-dimensionality *n* influence the critical behavior of many-body systems.

**Table 2:**
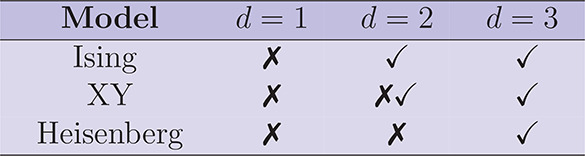
Either the absence (X) or the presence (checkmark symbol) of a phase-transition towards conventional long-range order at finite temperatures in model Hamiltonians^a^

a The simultaneous presence of both symbols in the 2*d*-XY model indicates the presence of quasi-long-range order characterized by a correlation function that falls off as power law below *T* = *T*_*KT*_. Adapted with permission from ref (42). Copyright 1990 Springer.

For systems displaying long-range order, mean-field (MF) theory becomes inadequate around the critical point; it cannot accurately describe the critical behavior, or the singularities occurring in the thermodynamic functions at the critical temperature *T*_*c*_. The body of past experimental and theoretical studies established that in most cases the critical behavior of a thermodynamic function *f*(*t*) follows a power law in the reduced temperature *t*, where *t* = 1 – *T*_*c*_/*T* for *T* > *T*_*c*_ and *t* = 1 – *T*/*T*_*c*_ for *T* < *T*_*c*_. The critical exponents appearing in those power laws are defined as in Table 3.^43^

**Table 3:**
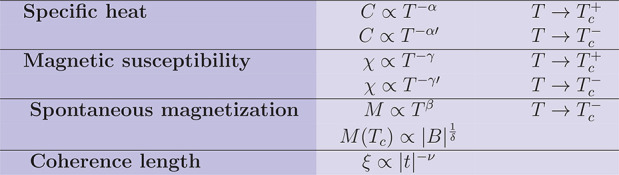
Definition of the critical exponents associated to specific thermodynamic or physical variables^a^

a Adapted with permission from ref (42). Copyright 1990 Springer.

The value of the critical exponents numerically extracted from the different models is displayed in Table 4.

**Table 4:**
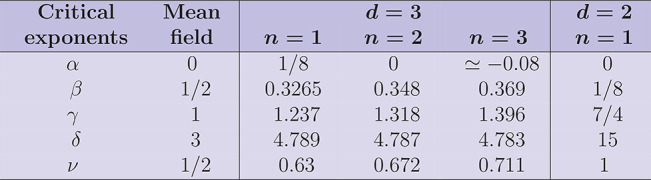
Comparison among the theoretical values for the critical exponents α, β, γ, δ, and ν according to the different models

These critical exponents follow scaling relations such as α + 2β + γ = 2, or γ = β(δ – 1), or 2β + γ = *dν*, implying that only two of the exponents are independent, so that from the knowledge of two arbitrary indices, all the others can be derived (see Magnetic Critical Behavior section for additional discussion).^44,45,47,48,49^

**Table 5:**
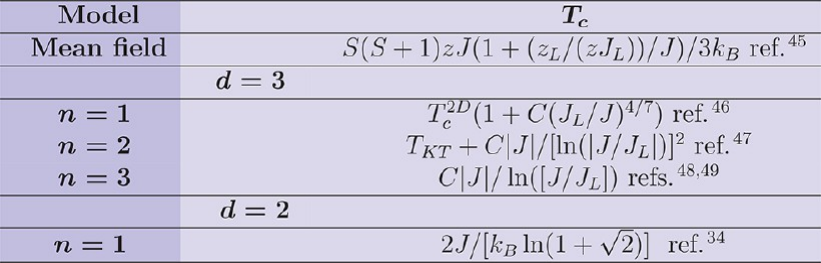
Critical temperature *T*_*c*_ for the different models^a^

a where *C* is a numerical coefficient, *S* is the spin on each site, and *z* and *z*_*L*_ are the intra- and interlayer coordination numbers. Assuming in the 3D case a layered structure with |*J*_*L*_/*J*|≪ 1, where *J* and *J*_*L*_ are the intralayer and the interlayer exchange couplings, respectively. In the case of the 2D Ising model (*d* = 2, *n* = 1), the reported expression refers to a square lattice.

### Magnetic Critical Behavior

#### Critical Exponents and Magnetic Equation of State

We start with descriptions of several standard critical exponents, and we sketch the phenomenological scaling theory of critical behavior.^50−53^ Let us consider a ferromagnet in equilibrium at temperature *T* and in uniform magnetic field *H*. We use the reduced temperature variable ε = (*T* – *T*_*c*_)/*T*_*c*_, where *T*_*c*_ is the critical temperature. We are interested in properties as ε → 0 with *H* = 0. Near phase transition, magnetic systems are characterized by power law behavior sufficiently close to the critical point. Specific heat *C*_*p*_, the spontaneous magnetization *M*_*s*_, and the inverse initial magnetic susceptibility  as well as the correlation length ξ can be described by *C*_*p*_(*T*) ∝ (−ε)^α^, *M*_*s*_(*T*) ∝ (−ε)^β^, , *M* ∝ *H*^1/δ^, and ξ ∝ε^–ν^, where ε = (*T* – *T*_*c*_)/*T*_*c*_ is the reduced temperature.^54^ Another exponent η describes the spatial decay of the correlation function at criticality Γ(*r*) ∝ *r*^–*d*+2−η^, where *d* denotes the spatial dimensionality of the system.^55^ As described above, the exponents α, β, γ, δ, η, and ν are called critical exponents. The phenomenological scaling theory predicts that the critical exponents are connected by the scaling laws α + 2β + γ = 2 (Rushbrooke), γ = (2 – η)ν (Fisher), γ = β(δ – 1) (Widom), 2 – α = *νd* (Josephson), and Δ = *βδ* = β + γ (Δ is the gap exponent).^55−61^ The existence of long-range magnetic order at finite temperature in 2D vdW magnets heavily depends on the spin dimensionality *n* (*n* = 1, uniaxial or Ising spins; *n* = 2, XY or planar spins; *n* = 3, ordinary or Heisenberg spins; Figure 2) and on the strength of magnetic anisotropy. The long-range magnetic order in ideal Heisenberg 2D magnetic system is prevented by thermal fluctuations based on Mermin-Wagner theorem.^40^ The presence of strong uniaxial anisotropy of 2D Ising type with *n* = 1 can open a gap in the spin-wave spectrum, thus suppressing the effect of thermal fluctuations.^62^ When *n* = 2 with an easy-plane anisotropy, a quasi-long-range topological magnetic order could be established below the Berezinskii–Kosterlitz–Thouless transition (*T*_*KT*_) that is characterized by an algebraic decay of spin correlations and by the presence of bound pairs of vortex and antivortex arrangement of spins.^62−64^ Critical exponents for different theoretical models in 2D and 3D are listed in Table 4.^43,62,65,66^

Besides a number of relations between critical exponents, scaling finds specific predictions on the magnetic equation of state: *M*(*H*, ε) = ε^β^*f*_±_(*H*/ε^β+γ^). By using the scaled magnetization *m* ≡ ε^–β^*M*(*H*, ε) and field *h* ≡ ε^–(β+γ)^*H*, the magnetic equation of state takes the familiar form *m* = *f*_±_(*h*), where *f*_+_ for *T* > *T*_*c*_ and *f*_–_ for *T* < *T*_*c*_, respectively, are the regular functions. This relates *M*, *H*, and *T*, and it also implies that for the true scaling relations and for the right choice of β, γ, and δ values, scaled *m* and *h* will fall on universal curves above *T*_*c*_ and below *T*_*c*_, respectively. Another commonly used form of the magnetic equation of state is *H*/*M*^δ^ = *k*(ε/*H*^1/β^), where *k*(*x*) is the scaling function. The scaled critical isotherms, *MH*^–1/δ^*versusεH*^–1/(*βδ*)^, will collapse into a single curve, and the *T*_*c*_ is located at the zero point of the horizontal axis.

### Modified Arrott Plot, Kouvel–Fisher Plot, and Critical Isotherm

In the modified Arrot plot *M*^1/β^*versus* (*H*/*M*)^1/γ^ based on the Arrot–Noaks equation of state^67^
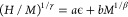
the *M*(*H*, *T*) data taken in the critical region should fall on a set of parallel straight-line isotherms with the one at *T* = *T*_*c*_ passing through origin for a proper choice of the exponents β and γ. Values β = 0.5 and γ = 1.0 describe the Arrot plot in the mean-field approximation.^68^ Critical temperature can be determined accurately since the isotherm at *T*_*c*_ will pass through the origin. Moreover, this plot directly gives  and *M*_*s*_(*T*) as the intercepts on the *H*/*M* axis and positive *M*^2^ axis, respectively. It is also applicable to estimate the order of magnetic transition through the slope of the straight line based on Banerjee’s criterion.^69^ First- (second-) order phase transition corresponds to a negative (positive) slope.

Once *M*_*s*_(*T*) and  have been obtained by the modified Arrott plot, critical exponents β, γ, and *T*_*c*_ can be determined by the Kouvel–Fisher analytical method with relatively high accuracy: *Y*(*T*) = *M*_*s*_(*T*)/[*dM*_*s*_(*T*)/*dT*] = (*T* – *T*_*c*_)/β and .^70^ The plots of *Y*(*T*) and *X*(*T*) against *T* are straight lines with slopes 1/β and 1/γ, respectively. The most important advantage of the Kouvel–Fisher plot is that no prior knowledge of *T*_*c*_ is needed, as the intercept on the *T*-axis is at *T*_*c*_. Both *Y*(*T*) *versusT* and *X*(*T*) *versusT* plots should yield the same value of *T*_*c*_ which can be used for a precise determination of the exponents.

An iterative method can be used to obtain the most accurate values of β, γ, and *T*_*c*_.^71^ The linear extrapolation from the high field region to the intercepts with the axis *M*^1/β^ and (*H*/*M*)^1/γ^ in the modified Arrott plot yields reliable values of *M*_*s*_(*T*) and . A set of β and γ can be obtained by using the Kouvel–Fisher analytical method. The values of these exponents are then used to reconstruct the modified Arrott plot. Intercepts on the axes lead to an additional set of *M*_*s*_(*T*) and  from which β and γ values are derived. This iteration procedure is continued until β and γ are stable and unaltered by increasing number of iterations. Such refining process converges very rapidly from a proper initial model and yields the accurate exponent values β and γ. Another exponent δ can be determined from the critical isotherm analysis (*M* ∝ *H*^1/δ^ at *T* = *T*_*c*_) and the Widom scaling relation δ = 1 + γ/β.

### Absence of Magnetic Order in 2D: The Hohenberg, Mermin, and Wagner Theorem

Hohenberg published over 50 years ago a rigorous proof for the nonexistence of long-range order in a 2D superfluid or superconductor at finite temperatures.^39^ This proof was quickly extended by Mermin and Wagner to the Heisenberg Hamiltonian, in one and two dimensions for a Heisenberg ferromagnet (or antiferromagnet) with SU(2) symmetry, or a magnetic system with U(1) symmetry and an order parameter perpendicular to the symmetry axis.^40^ Soon thereafter, Mermin would prove the absence of translational long-range order in a two-dimensional (2D) crystal, whether in quantum or classical mechanics.^72^ Nevertheless, after the work by Berezinskii,^73^ Kosterlitz and Thouless,^64,74^ and Nelson and Kosterlitz,^75^ we now know that in two dimensions there will be a sharp transition temperature at a certain *T*_*KT*_, where for *T* > *T*_*KT*_ the associated correlation function will fall off exponentially with the distance. For *T* < *T*_*KT*_ the correlation function is expected to fall off as a power law, leading to what is commonly called as quasi-long-range order, with an exponent α ≥ 3. In contrast, for the Heisenberg model, the order-parameter correlation function decays exponentially with the distance at any nonzero temperature, and therefore there would be no phase transitions at finite temperatures.^76^ Nevertheless, the Hohenberg, Mermin, and Wagner (HMW) theorem sheds no light on whether quasi-long-range order can exist in any particular system. Hohenberg did note however that his theorem concerning the absence of long-range order for a 2D superfluid or superconductor would remain unaffected by the introduction of long-range interactions between the particles. In contrast, as discussed in ref (76), the absence of long-range order in the Heisenberg ferromagnet depends on the range of the interactions. Assuming *J*(*r*) to be the coupling constant between pairs of spins separated by a certain distance *r*, one can thus define a second magnetic moments as
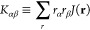
4where α and β denote both planar spatial directions. The absence of long-range magnetic order, as given by the Mermin–Wagner theorem, would require that the values of *K*_*αβ*_ remain finite. However, if the spins are allowed to rotate around an axis symmetry, one would define two coupling constants *J*^∥^(*r*) and *J*^⊥^(*r*) for spin components parallel and perpendicular to such axis of symmetry. Then, Mermin–Wagner would rule out long-range magnetic order for the spin components perpendicular to this axis of symmetry, provided that the second moments for *J*^⊥^ remain finite. Such condition is not required for *J*^∥^. For FM interactions decaying as *r* – (*n* < 4), *K*_*αβ*_ would diverge implying that long-range FM order should be possible in two dimensions, at small, but nonzero temperatures.^44^ However, it is important to state that the HMW theorem is unable to clarify the existence, or nonexistence, of long-range order in a quantum mechanical system in the limit *T* = 0 K. Yet, according to Halperin,^76^ nonrigorous arguments similar to those invoked at finite temperatures suggest that in many circumstances, long-range order would also be impossible at *T* = 0 K. In fact, in ref (76), Halperin provides a generalization of the HMW argument to rigorously rule out the possibility of ferromagnetism at *T* = 0 K for any 2D electron model that excludes spin–orbit coupling or magnetic dipole interactions.

### Discovery of Magnetism in Exfoliated Monolayers

Therefore, it is in the context of the HMW that one should place the discovery of ferromagnetism in exfoliated monolayers of both CrI_3_^5^ and Cr_2_Ge_2_Te_6_^6^ through the magneto-optical Kerr effect (MOKE). For CrI_3_, the Curie temperature is found to decrease down to 41 K in the monolayer limit with respect to its bulk value of 61 K, which is a rather modest effect.^5^*T*_*c*_ in Cr_2_Ge_2_Te_6_ is found to decrease from ∼65 K in the bulk to approximately 30 K in bilayers.^6^ Interestingly, the nature of the FM order in CrI_3_ is highly sensitive to the number of layers in the system. In a bilayer, the remnant magnetization present in a single layer is suppressed and is consistent with each layer having oppositely oriented spins or with the material becoming an antiferromagnet. From a theoretical perspective, these results imply that these compounds cannot be described by an isotropic Heisenberg Hamiltonian but are subjected to magneto-crystalline anisotropy which in the case of Cr_2_Ge_2_Te_6_ is claimed to increase considerably upon the application of an external magnetic field. In the CrI_3_ case, it displays a substantial remnant magnetization in the absence of a magnetic field which is directed perpendicular to the plane of the lattice. Therefore, this magnetic system would be well described by the 2D Ising model.

### 2D Magnets: Recent Progress and Current Challenges

Pioneering work establishing intrinsic ferromagnetism in two-dimensions has been performed on monolayer CrI_3_,^5^ Fe_3_GeTe_2_,^77^ and a few-layer Cr_2_Ge_2_Te_6_,^6^ which are considered now as prototypical 2D magnets. More recently, experimental studies on monolayer CrBr_3_^20^ and CrCl_3_^78^ and in few-layer V_5_Se_8_, CrTe_2_, and Cr_2_Te_3_ have also been reported.^79−81^ Whereas in the bulk and few-layer regime the Curie temperatures *T*_*c*_ approach room-temperature, in some of the systems such as in Fe_3_GeTe_2_^77^ and CrTe_2_,^81^ this ordering is substantially suppressed in the strictly 2D limit, i.e., in the monolayer regime. For instance, exfoliated monolayers of CrBr_3_, CrI_3_, and Fe_3_GeTe_2_ display, through magnetization measurements, *T*_*c*_ values of 25 K,^82^ 45 K,^5^ and 130 K,^6^ respectively, whereas monolayers of CrBr_3_^83^ and CrCl_3_^78^ prepared by molecular beam epitaxy (MBE) display *T*_*c*_ values of 16 and 13 K, respectively.^78^ In contrast, few-layer Cr_2_Ge_2_Te_6_ and V_5_Se_8_ are nearly ideal Heisenberg ferromagnets with *T*_*c*_’s below 60 K, but with weak FM ordering in the truly 2D limit.^6,79^

The magnetic interactions in bilayer CrI_3_ and CrBr_3_ have been found to be highly dependent on the stacking order^20^ and can be tuned between antiferromagnetic (AF) and ferromagnetic (FM) with magnetic or electric fields^8,9^ and *via* applied mechanical pressure.^22,23^ There is currently a rich palette of 2D magnetic materials ranging from Ising, Heisenberg to XY behavior, and with a diversity of exchange interactions (itinerant, double-, or superexchange)^62,84,85^ which allow to tailor their magnetic properties on demand. There is no doubt that this enormous progress is of great fundamental interest, but the applications based upon 2D magnets remain limited due to two main current constraints: (i) the magnetic ordering temperature remains well below 300 K and (ii) the lack of synthesis methods that are scalable and produce homogeneous magnetic monolayers over large areas by bottom-up methods.

### Enhancing the Magnetic Ordering Temperature in Two Dimensions

Efforts to achieve room-temperature ferromagnetism in some of these ultrathin layered magnets are currently being pursued. In semiconducting magnets, strong charge doping *via* ionic liquid gating has been shown to drastically increase the Curie temperature of Cr_2_Ge_2_Te_6_ from 60 K to nearly 200 K.^86^ In metallic systems such as Fe_3_GeTe_2_ (FGT), where electric gating is less efficient,^12^ alternative methods to increase the ordering temperature have been recently reported, such as changing the Fe stoichiometry in the lattice,^87,88^ by cobalt codoping^89^ or by bringing it in proximity to a topological insulator.^90^ In stoichiometry tuning studies, the composition is varied from Fe_*x*_GeTe_2_ (*x* ≃ 3–5), whereby the *T*_*c*_ increases with Fe content, up to 310 K.^87,88^ A partial substitution with Co-atoms (up to 26%) has been found to further increase the *T*_*c*_ to 328 K, while a further increase in Co-doping induces a concomitant structural and magnetic phase transition to an AF ground state.^89^ As for the *T*_*c*_ enhancement by proximity effect, an substantial improvement has been achieved in FGT/Bi_2_Te_3_ heterostructures, where a thickness dependent increase of the *T*_*c*_ up to 380 K (for FGT having a thickness of 4 nm) was reported.^90^ These intriguing results are not fully understood, especially whether effects such as strain or doping at the FGT/Bi_2_Te_3_ interface play a role in this observation, or if this can be attributed to the exotic topological character of Bi_2_Te_3_.

In another recent study, an anomalous enhancement of *T*_*c*_ in Cr_2_Te_3_ flakes was found as the thickness of the flake is decreased from 10 to 5.5 nm,^80^ behaving in an opposite way to nearly all other 2D magnets reported to date. This effect was attributed to doping and reconstruction at the surface of Cr_2_Te_3_, leading to a slightly different stoichiometry and interlayer distance. On another front, magnetic impurity doping and defect-induced magnetism of otherwise nonmagnetic transition-metal dichalcogenides (TMDs) is being attempted. Whereas defect-induced magnetism found in PtSe_2_ flakes^91,92^ results in a very low Curie temperature, transition-metal doping is found to boost the *T*_*c*_ as high as room temperature, as reported in V-doped WSe_2_ monolayers grown by a powder vaporization method.^93^ In another experimental study, room-temperature ferromagnetism was found in MoTe_2_ doped with a nonmagnetic element, *i.e.*, Ta.^94^ This set of results coming from monolayers and nanosheets synthesized and doped through chemical vapor methods are quite intriguing and merit more experimental investigations. In this regard, it is also worth mentioning early works on room-temperature ferromagnetism in monolayer 1T-VSe_2_^18^ and MnSe_2_^17^, which could not be reproduced by other groups. Such systems have remained controversial as they relied mainly on volumetric magnetometry measurements of monolayers on large-volume substrates.

This urgent need for a room-temperature 2D magnet has recently caught the attention of many theoretical groups, from which a large number of high-*T*_*c*_ 2D magnetic materials has been predicted.^95^ For the sake of briefness, we are going to highlight only a couple of structural material families: (i) Janus monolayers of TMDs (MXY compounds, M: Cr, V, Mn and X, Y: Se, Te, S)^96,97^ and (ii) Cr-based III–V semiconducting layered compounds (CrP, CrAs),^98^ both of which have certain compounds in their structural families that reach well beyond room-temperature, at least from *ab initio* calculations. As for the TMD Janus systems, VSeS^96^ and VSeTe^96,97^ are the ones attaining a *T*_*c*_ above room temperature, with values of 420 K^96^ and 310 K^96^ (350 K),^97^ respectively, as shown in Figure 2. With regard to the III–V semiconducting compounds, CrP is found to order ferromagnetically up to 230 K,^98^ whereas calculations on CrAs show an easy-plane magnet behavior with an astonishingly high transition temperature of 855 K^98^ (albeit being expected to be of Berezinskii–Kosterlitz–Thouless type, or to display quasi-long-range order). These particular theoretical studies highlighted here are certainly going to trigger experimental investigations, given the ability to grow TMDs and III–V compounds by several bottom-up methods, such as MBE.

### Toward the Fabrication of Scalable, Large-Area 2D Magnets

**Figure 3:**
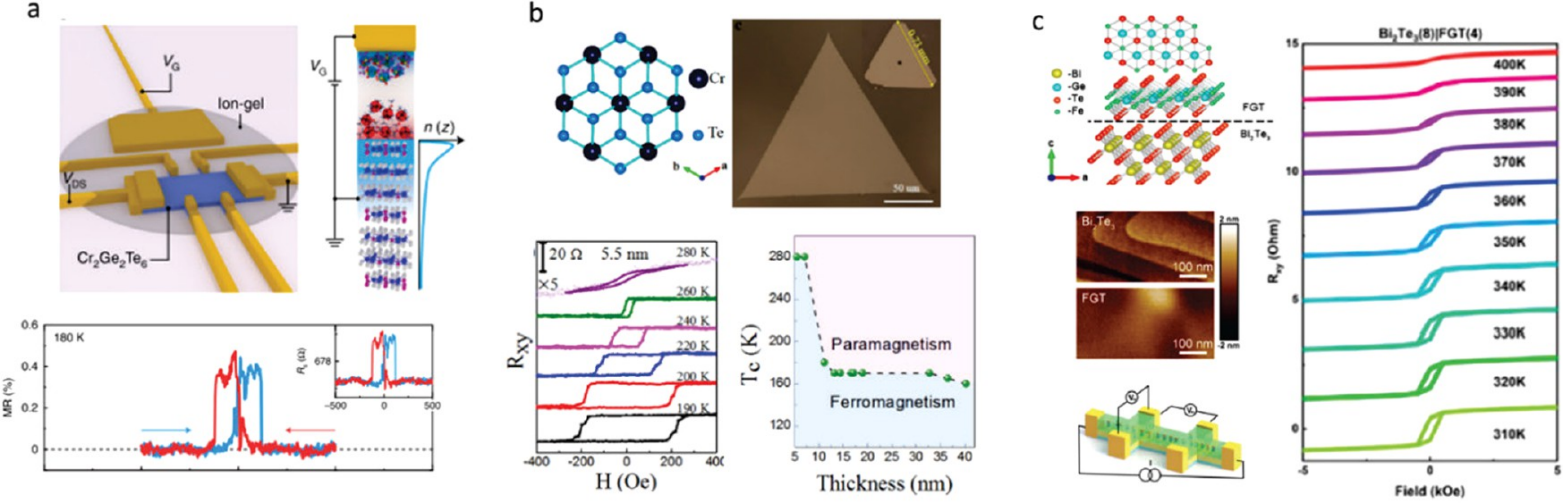
Routes toward room-temperature FM ordering in 2D layered materials by (a) electrostatic gating, (b) tuning dimensionality, and (c) proximity effect. Representative experimental examples include: (a) ionic liquid gating in Cr_2_Ge_2_Te_6_, achieving a dramatic increase from 60K to 180 K in the ordering temperature. Adapted with permission from ref (86). Copyright 2020 Springer Nature. (b) Substantial *T*_*c*_ enhancement in Cr_2_Te_3_ flakes as the thickness is reduced, measured by anomalous Hall effect. Adapted with permission from ref (80). Copyright 2020 American Chemical Society. (c) Persistent magnetic signals up to 380 K in 4 nm-thick Fe_3_GeTe_2_ interfaced with the topological insulator Bi_2_Te_3_. Adapted with permission from ref (90). Copyright 2020 American Chemical Society.

The synthesis of bulk layered magnets have a long history, but it is the recent technical breakthrough in exfoliation, isolation and encapsulation of 2D materials, that made possible to study magnetism in the truly 2D regime. Despite the enormous progress in preparing high-quality flakes and heterostructures of 2D materials, the exfoliated samples have dimensions of a few microns and frequently present an irregular shape, which represents a major limitation for their use in scalable device applications. An alternative fabrication strategy, which has been less popular than exfoliation due to its increased cost and the long material optimization process, is to use bottom up methods such as chemical vapor deposition (CVD) or MBE for the synthesis of 2D magnets. While the growth of only one family, that of doped (V- and Cr) TMDs, has been achieved by CVD so far,^100^ MBE has been successful in preparing a couple of 2D magnetic systems such as monolayered CrBr_3_^20,83^ and CrCl_3_^78^ and few-layer V_5_Se_8_^79^ and Fe_3_GeTe_2_^90,99^ with Curie temperatures lower than in their bulk counterparts (Figure 5). In the monolayer regime, the magnetism of CrBr_3_ was studied by local spin-polarized scanning tunneling microscopy (STM)^20^ and magneto-optical Kerr-effect (MOKE),^83^ the latter yielding a *T*_*c*_ of 20 K. In the case of CrCl_3_, a homogeneous monolayer coverage over large areas was achieved on Graphene/SiC(0001) substrates (see Figure 3), whereas the magnetic properties were evaluated *via* X-ray magnetic circular dichroism (XMCD) over larger areas, yielding a *T*_*c*_ of 13 K and a sizable easy-plane magnetic anisotropy.^78^ On top of the efforts to grow wafer scale 2D magnets by MBE (Figure 5), the selection and deposition of suitable capping layers to protect these materials from oxidation is a key step toward device processing.

**Figure 4:**
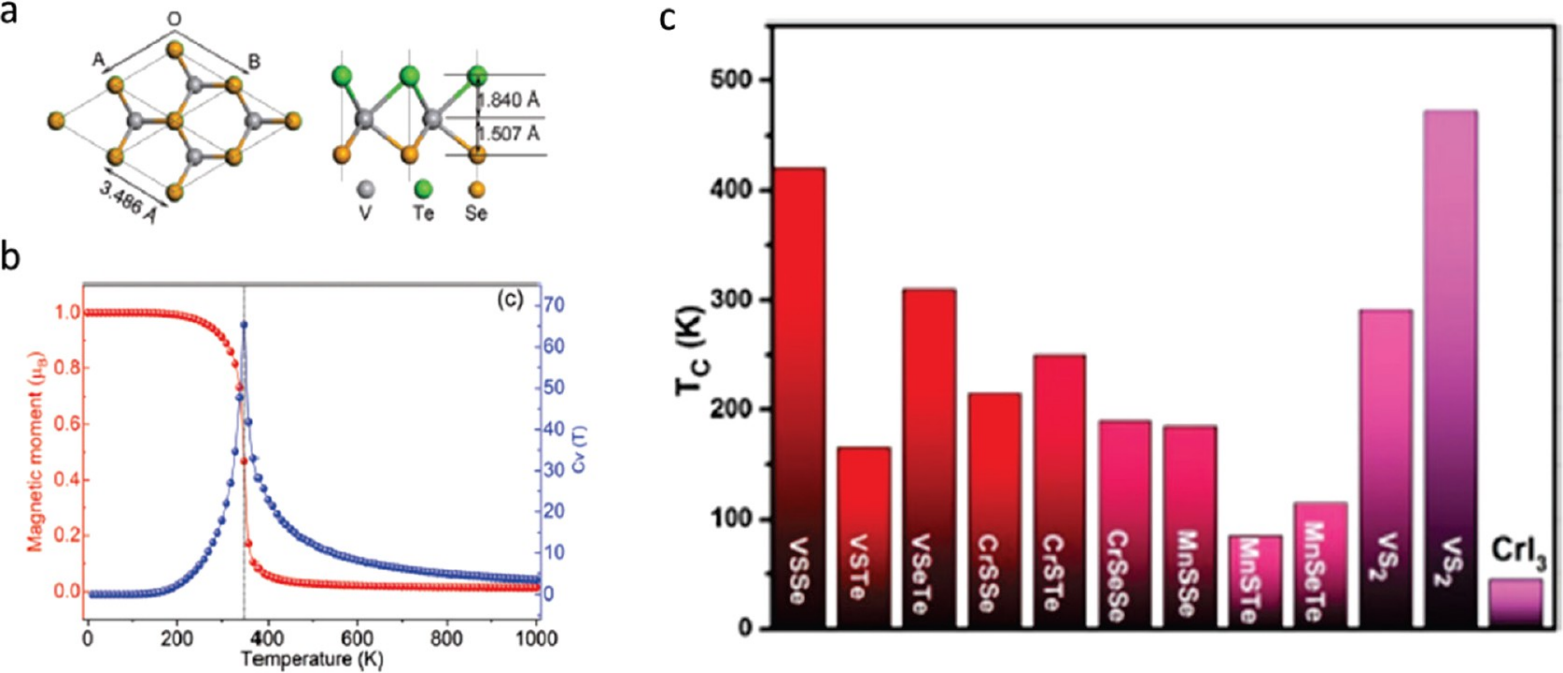
Theoretical calculations for the Curie temperature in TMD Janus systems. (a) Diagram of the VSeTe crystal structure in top-down and cross-sectional views. (b) Temperature-dependent magnetic moment and specific heat of VSeTe, obtained *via* Monte Carlo simulations in a nearest-neighbor Heisenberg exchange model. Panels (a) and (b) reprinted with permission from ref (97). Copyright 2009 Royal Society of Chemistry. (c) Curie temperatures of V-, Cr- and Mn-based Janus TMDs, highlighting VSSe and VSeTe as the candidates with highest *T*_*c*_. Adapted with permission from ref (96). Copyright 2018 Elsevier.

**Figure 5:**
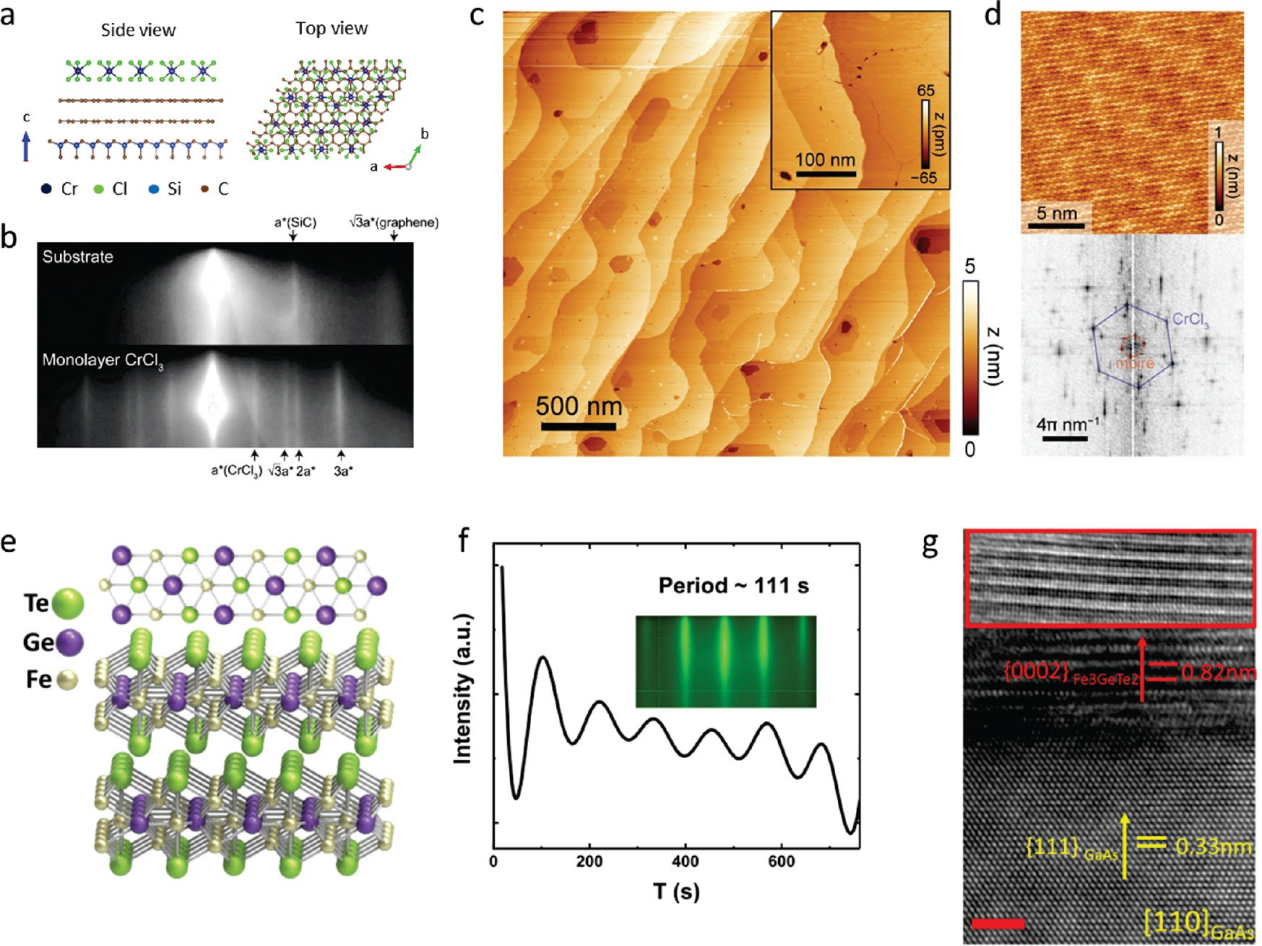
Examples of large-area 2D magnets grown by MBE. (a–d) Monolayer CrCl_3_ on Graphene/SiC(0001) and (e–g) few-layer Fe_3_GeTe_2_ on GaAs (111). (a) Schematic crystal structure of CrCl_3_/graphene/6H-SiC layers in top-down view and cross-section view. (b) *In situ* RHEED pattern of the substrate and monolayer CrCl_3_ grown by MBE, along Γ–M of graphene (Γ–K of SiC). Streaks from different high-symmetry directions of CrCl_3_ are observed, implying a twisted in-plane orientation of the grains. (c) STM topography of a monolayer CrCl_3_ grown on graphene/6H-SiC(0001), indicating a homogeneous coverage on long length scales. Inset: A magnified topography image, which reveals the grain boundaries. (d) Atom resolved image of the CrCl_3_ lattice featuring a moiré pattern (upper panel) and its Fourier transformed image (lower panel). Panels (a–d) adapted with permission from ref (78). Copyright 2021 AAAS. (e) Crystal structure of Fe_3_GeTe_2_. (f) RHEED oscillations indicating layer-by-layer growth of Fe_3_GeTe_2_ (0001) on GaAs (111), and the corresponding electron diffraction pattern (inset). The inferred growth rate is 111 s per monolayer. (g) Transmission electron microscopy of a Fe_3_GeTe_2_/GaAs cross-section, indicating the (111)/(0002) epitaxial relationship. Panels (e–g) adapted with permission under a Creative Commins CC BY license from ref (99). Copyright 2017 Springer Nature.

## Device Implementations and Basic Concepts

### Magnetoresistance

For spintronic device applications, a crucial material property is magnetoresistance—a dynamic and reversible change in sample resistance under varying magnetic field. Magnetic materials and their heterostructures can manifest exceptionally large switchable magnetoresistance due to a change in the magnetic structure or spin configuration upon the application of a magnetic field. There are two prominent strategies for exploiting magnetoresistance from magnetic compounds in functional electronics. One is engineering atypically large magnetoresistance through the fabrication of multilayer heterostructures such as MTJs. The concept was demonstrated independently by Baibich *et al*.^101^ and Binasch *et al*.,^102^ in which multilayer films consisting of adjacent FM electrodes leads to giant negative magnetoresistance when switching from an AF to FM configuration.^101−103^ Since then, MTJs have been further optimized to increase the magnetoresistance by changing the geometry to either two FM metals separated by an insulating barrier (conventional spin valves) or two nonmagnetic metals with an (anti)ferromagnetic barrier (less conventional spin filter). Despite numerous efforts to enhance the performance of MTJs over many decades, they still suffer from issues such as retaining the crystallinity and magnetization of ultrathin magnetic films and enlarging the tunnelling magnetoresistance (TMR) which prevent miniaturization of high-performance devices. The second approach is utilizing the high degree of electronic tunability of intrinsically magnetic 2D semiconductors (or 2D metals with high density ionic gates) to provide control over both charge and spin carriers, allowing for complete spin polarization of their conduction electrons. By exploiting the spins of electrons as information carriers, instead of their charge, these materials promise to improve the speed, density, and energy efficiency of electronic devices through single-spin transport.^104−106^ They are particularly attractive for device applications that utilize both the electronic tunability and spin-polarized transport in lateral MTJs and spin field effect transistors.^107,108^ The recent discovery of magnetism in atomically thin vdW materials, coupled with the diversity of their observed electronic properties including insulators (CrCl_3_, CrBr_3_, CrI_3_), semiconductors (CrSBr, Cr_2_Ge_2_Te_6_), and metals (Fe_3_GeTe_2_, Fe_5_GeTe_2_, CrTe_2_), makes vdW magnets exceptionally attractive for nanospintronic applications (see Spintronics: From Fundamentals to Devices section for details). An unusual property of 2D magnets is the abundant observation of strong intralayer magnetism with high magnetic anisotropy, which allows for the existence of magnetic order in the 2D limit, with weak interlayer coupling, which often results in layer-dependent spin ordering.^5^ These features, tied with the observed high crystallinity and low disorder characteristics of the parent compounds without forming dangling bonds in the 2D limit, make vdW magnets especially attractive for the fabrication of MTJs.^13−16,109^ The layered magnetic properties of vdW magnets also manifest as large intrinsic magnetoresistance in conducting vdW magnets,^12,86,110,111^ which, when paired with the high degree of electronic tunability obtained through electrostatic gating in the 2D limit, offers flexible control over both electronic and magnetic properties^12,86,111^ in transport devices fabricated with vdW magnets.

### Spin Filtering Effect

**Figure 6:**
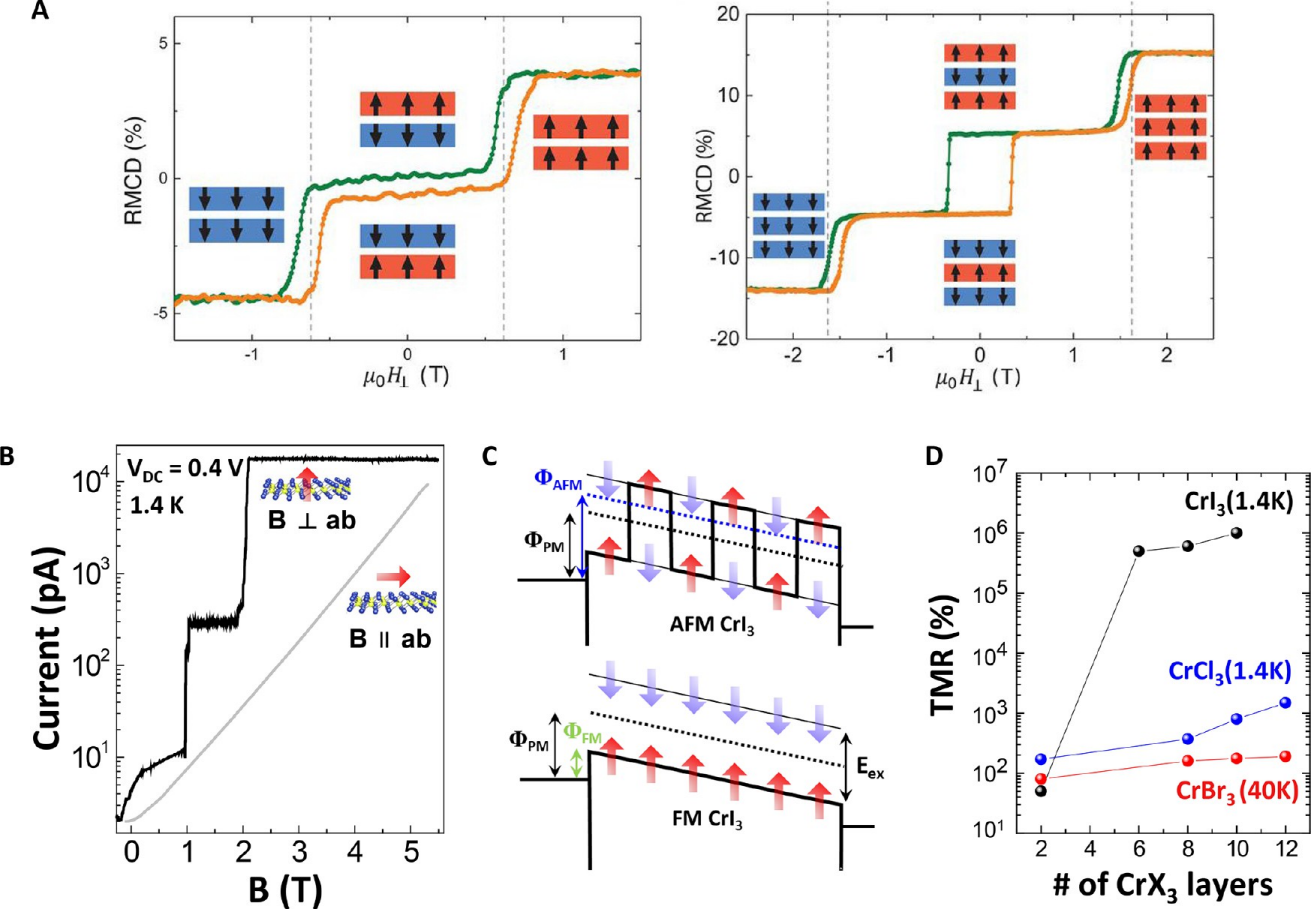
Enormous TMR in vdW MTJs induced by layered antiferromagnetism. (A) Reflective magnetic circular dichroism (RMCD) of bilayer (left) and trilayer (right) CrI_3_ showing layer-dependent magnetism. Reproduced with permission from ref (13). Copyright 2018 AAAS. (B) Magnetic-field-dependent current change in a vdW MTJ incorporating magnetic CrI_3_. (C) Schematic energy diagrams of CrI_3_-based MTJs with AF barrier (top) and FM barrier (bottom). Panels (B and C) are adapted with permission from ref (16). Copyright 2018 American Chemical Society. (D) Summary of TMR values as a function of thickness. Reproduced from ref (112). Copyright 2019 American Chemical Society.

In fabricating MTJs from vdW materials, a particularly useful property is the existence of a layered AF ground state such as the one observed in CrI_3_, CrCl_3_, and CrSBr (Figure 6A for representative CrI_3_ case). It is worth noting that this layered AF structure is usually artificially made^113,114^ in traditional heterostructures, as it is rarely naturally occurring. Consequently, fabricating multiple-layered AF structures is prohibitively difficult with existing fabrication techniques. Although it is possible to realize the conventional spin-valve device using two FM metallic flakes separated by an insulating or semiconducting material^109,115^ (see Spintronics: From Fundamentals to Devices section for details), the exotic spin configuration of a layered AF ground state in 2D magnets gives rise to a substantial spin-filtering effect. From the point of view of MTJs, vdW magnets having layered AF structure are attractive as a tunnel barrier since the number of AF layers is determined purely by the thickness of the flakes and the naturally formed multiple antiparallel spin configurations can trigger enormous TMR values. Accordingly, several groups have reported TMR in vdW MTJ devices incorporating multilayer CrI_3_ as the insulating tunnel barrier sandwiched between graphene electrodes.^13−16^ The TMR at 1.4 K has been found to be as large as 10^6^% under a 2T magnetic field as shown in Figure 6B, which is ∼10^3^ times larger than a previous world record of giant magnetoresistance in a pseudospin-valve MTJ device with a MgO tunnel barrier.^116^

Given that the origin of the TMR value is due to the barrier height difference between the antiparallel and the parallel multistacks^16^ (which results in an exponential increase in TMR) as shown in Figure 6C, it is suspected that the performance could be enhanced further by finding materials with larger exchange splitting and lower barrier heights. Other chromium trihalides such as CrBr_3_ and CrCl_3_ have been studied, but CrI_3_-based MTJs still exhibit the highest TMR values due to the largest splitting combined with a narrow bandgap,^112,117−120^ as shown in Figure 6D. Another advantage of vdW MTJs is that the magnetic properties are easily tuned by external factors (electric fields,^11^ doping,^8,9^ and pressure^22,23^). Such a high degree of tunability can potentially give significant performance breakthroughs in future MTJ-based devices. Despite the promising possibilities of vdW MTJs, there are still myriad challenges remaining before vdW MTJs are useful for practical applications. These include finding higher *T*_*c*_ materials for room-temperature operation, developing nonvolatile functionality for memory applications, reducing the operation field for low power consumption, increasing the junction conductance while retaining high TMR, and enhancing the ambient/chemical stability for more reliable fabrication processes.

### Lateral Transport Properties

**Figure 7:**
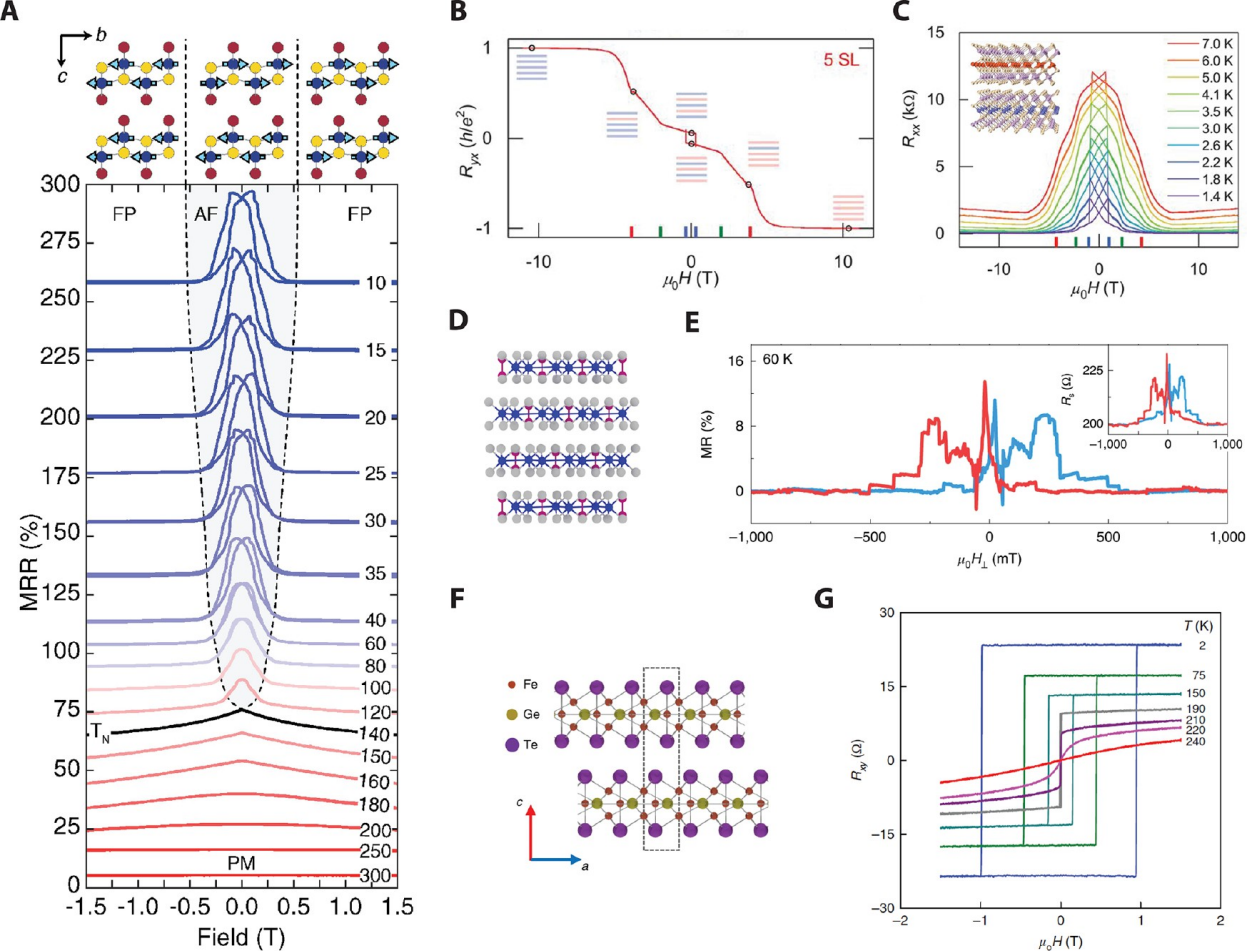
Summary of intrinsic magnetoresistance in vdW magnets. (A) Magnetoresistance ratio MRR(*B*)  in bulk CrSBr *versus* magnetic field (parallel to the *b*-axis) at various temperatures. Each MRR curve is offset for clarity. The solid black line is an MRR curve taken near the Néel temperature. The antiferromagnetic (AF), fully polarized (FP), and paramagnetic (PM) phases are labeled, and the phase boundary is denoted by dashed black lines. Schematics showing the orientation of the spins in the AF and FP states are given above the plot.^110^ Reproduced with permission from ref (110). Copyright 2020 John Wiley and Sons. (B) *R*_*yx*_ of a 5-layer MnBi_2_Te_4_ sample as a function of external magnetic field applied perpendicular to the sample plane at *T* = 1.6 K. Data are symmetrized to remove the *R*_*xx*_ component.^111^ (C) *R*_*xx*_ of a 5-layer MnBi_2_Te_4_ flake as a function of magnetic field acquired at various temperatures. Data are symmetrized to remove the *R*_*yx*_ component. Inset shows the layered crystal structure of MnBi_2_Te_4_ in the AF state.^111^ Panels (B) and (C) are reproduced with permission from ref (111). Copyright 2020 AAAS. (D) Ball and stick model of the Cr_2_Ge_2_Te_6_ crystal structure.^86^ (E) Magnetoresistance  curves for *T* = 60 K and back-gate voltage of 3.9 V for a 22 nm-thick Cr_2_Ge_2_Te_6_ flake. The background is removed for clarity. The magnetic field is applied in the out-of-plane direction. Unprocessed data are shown in the inset.^86^ Panels (D) and (E) are reproduced with permission from ref (86). Copyright 2020 Springer Nature. (F) Side view of the atomic lattice of bilayer Fe_3_GeTe_2_. The dashed rectangular box denotes the crystal unit cell.^77^ (G) Temperature-dependent magnetic field (out-of-plane) sweeps of the Hall resistance measured on a 12 nm thick Fe_3_GeTe_2_ flake.^77^ Panels (F) and (G) are reproduced with permission from ref (77). Copyright 2018 Springer Nature.

A few conducting vdW magnets manifest intrinsic magnetoresistance, including the semiconducting CrSBr^110^ and Cr_2_Ge_2_Te_6_,^86^ the metallic Fe_3_GeTe_2_,^12,77^ and the topological insulator MnBi_2_Te_4_.^111^ CrSBr and MnBi_2_Te_4_ are both vdW antiferromagnets which manifest strong intraplanar ferromagnetism with weak layered antiferromagnetism.^110,121^ CrSBr, a functional semiconductor with an easy axis aligned parallel to the sample planes,^110,122^ exhibits a large negative magnetoresistance of ∼ −40% at 10 K with a low saturation field of 0.5 T along the easy axis (Figure 7A).^110^ This behavior is attributed to the suppression of interlayer tunnelling in the AF phase due to spins in adjacent planes having opposite magnetization. In the fully polarized state (in which all spins are aligned with an external magnetic field), interlayer tunnelling is restored, leading to a decrease in the overall sample resistance. In the few-layer limit, vdW materials with layered antiferromagnetism have a layer-dependent ordering; odd layers functionally behave as a ferromagnet due to a nonvanishing magnetic moment at zero magnetic field.^111^ For example, a 5-layer flake of MnBi_2_Te_4_ exhibits magnetic behavior reminiscent of a ferromagnet at zero field followed by a series of magnetic states due to the flipping of spins in adjacent layers upon the application of an out-of-plane magnetic field. The various magnetic states manifest as steps in the Hall resistance (Figure 7B). Due to the nontrivial band topology, the Hall resistance quantizes to  at the saturation field (∼6 T) from the formation of quantum anomalous Hall states. Accompanying this discovery was the observation of a giant negative magnetoresistance in the longitudinal resistance (Figure 7C).^111^ In layered ferromagnets, similar magnetoresistance features can be observed. In a 22 nm-thick flake of Cr_2_Ge_2_Te_6_(Figure 7D), a sizable magnetoresistance of ∼13% was observed in the longitudinal transport (Figure 7E) with a saturation field <500 mT. Since the magnetoresistance displayed significant hysteresis and the easy axis is out-of-plane, the large magnetoresistance is credited to the spontaneous magnetization of magnetic domains characteristic of ferromagnets. In metallic layered ferromagnets such as Fe_3_GeTe_2_ (Figure 7F),^12^ large magnetoresistance can arise from the anomalous Hall effect;^12,77,123^ the Hall resistance displays a sharp switching hysteresis at the saturation field (Figure 7G). Beyond the magnetoresistance behaviors observed in vdW magnets with layered magnetic configurations, an advantage of 2D vdW magnets is the observed sensitivity to electrostatic doping,^12,86,111^ which has demonstrated the ability to increase the magnetic ordering temperature up to room temperature.^12^

### The Role of Spin Waves in Tunnelling Devices with Ferromagnetic Barriers

Spin waves play a crucial role in the perseverance of the alignment of magnetic moments in their 2D arrangements as described by the Mermin–Wagner theorem. As many other fundamental excitations, they exhibit wave-particle duality. The spin waves may be described as wave-like fluctuation of the spin state of the magnetic moments localized in a crystal lattice with a quantum of such excitation treated as a magnon quasiparticle. Akin to quanta of lattice vibrations (i.e., phonons), magnons can manifest naturally in inelastic scattering processes monitored, *e.g.,* by optical or electrical methods. Therefore, Raman spectroscopy, neutron scattering,^124,125^ and/or tunnelling spectroscopy^126−130^ are common techniques used to get insight into the properties of magnons in solids exhibiting localized magnetic moments.

**Figure 8:**
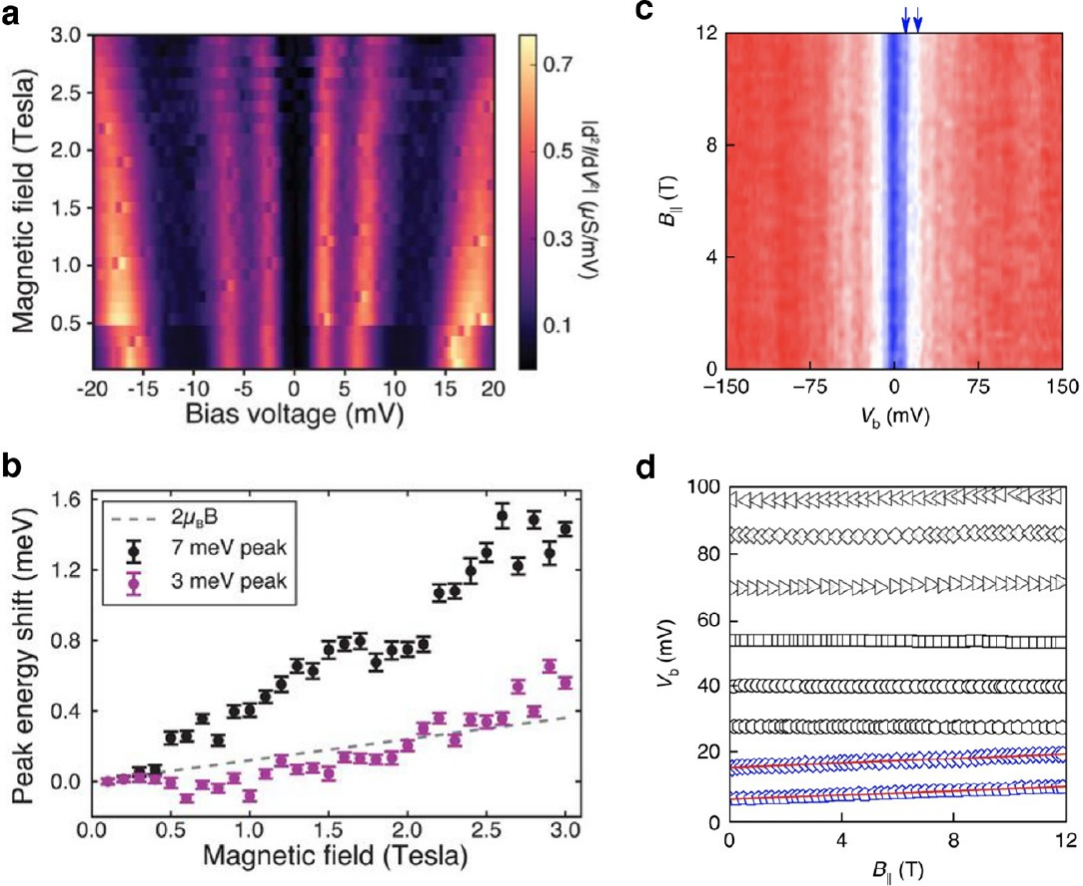
Comparison between tunnelling differential conductance spectra between Gr/CrI_3_/Gr and Gr/CrBr_3_/Gr devices in a magnetic field applied out-of-plane of the magnetic film for CrI_3_ sample and in-the-plane of the magnetic film for CrBr_3_ sample. For both materials, narrow low energy resonances appear, which are dispersive in the magnetic field. The color maps demonstrating the dependence of the differential conductance for (a, b) Gr/CrI_3_/Gr and (c) Gr/CrBr_3_/Gr devices illustrate the evolution of such resonances. The quantitative parameters describing the magnonic states, *i.e.*, their zero-field energy and the slope Δ*E*/Δ*B* can be extracted based on the linear fits to the evolution of the energy of the resonances in the magnetic field (b, d). Panels (a) and (b) are adapted with permission from ref (14). Copyright 2018 AAAS. Panels (c) and (d) are adapted with permission from ref (131). Copyright 2018 Springer Nature.

There are a few reports demonstrating the appearance of magnon resonances^14,120,131^ in 2D magnets, as seen in the conductance spectra in devices with graphene electrodes and magnetic barriers made of thin layers of CrI_3_,^14,120,131^ CrBr_3_,^120,131^ and CrCl_3_^120^ crystals. All three materials display evidence of magnon-assisted tunnelling processes in form of step-like features in the conductance spectra or narrow resonances in differential conductance spectra, as demonstrated in Figure 8. Characteristically for magnonic resonances, their energy is magnetic field dependent, and the observed slope Δ*E*/Δ*B* is indicative of the magnetic moment of the quasiparticles involved. Qualitatively, CrI_3_ and CrBr_3_ display two low energy resonances that are dispersive in magnetic field and may be associated with magnons. The tunnelling resonances in CrI_3_ occur at higher energy (3 and 7 meV) than those observed in CrBr_3_ (7.5 and 17 meV) as summarized in Table 6. The value of the Δ*E*/Δ*B* slope constitutes another differentiating factor between the two materials. The energies of the magnon resonances are consistent with the magnon density of states, calculated based on experimental values of the exchange integrals between the magnetic moments of Cr^3+^ ions in the lattice of CrI_3_ and CrBr_3_ crystals. However, the values of the Δ*E*/Δ*B* slope are more difficult to interpret.

**Table 6:** Zero-Field Energy and the Δ*E*/Δ*B* Slope Is Summarized for the Two Magnonic Resonances Observed in Gr/CrI_3_/Gr and Gr/CrBr_3_/Gr Devices, Based on the Evolution of the Differential Conductance Spectra in a Magnetic Field

	lower energy magnon resonance		higher energy magnon resonance	
material	energy (meV)	slope (μ_B_)	energy (meV)	slope (μ_B_)
CrI_3_^14^	3	2	7	8
CrBr_3_^131^	7.5	5.1	17	5.7

At the temperature of 0 K, the tunnelling processes are limited to those with an emission of a magnon quasiparticle, which arises from a spin flip mechanism within Cr^3+^ ions which gives rise to a quasiparticle characterized by a magnetic moment of 2.0 μ_B_. At finite (low) temperatures, the magnon–magnon interactions provide additional contribution to the Zeeman term, leading to the increase of the magnetic moment. Self-consistent spin-wave calculations^132^ for CrBr_3_ predict a magnetic moment of 2.4 μ_B_.^131^

The larger values of the experimentally observed Δ*E*/Δ*B* slopes in CrI_3_ and CrBr_3_ remain a puzzle; however, it is notable that the magnetic moment of magnons is strongly dependent on temperature, especially in the regime close to the critical Curie temperature (*T*_C_).^133^ First, the enhanced population of magnons at higher temperatures suppresses the short-range and long-range magnetic ordering. Second, the gap in the spin wave spectrum and interlayer exchange coupling (*J*_*L*_) suppresses the spin fluctuations (*i.e.*, making the magnon excitations stiffer). Consequently, two regimes of magnetic coupling may be identified, with the transition between them defined by a condition 2 μ_B_B ≈ *J*_*L*_, which differentiate the magnonic states by the value of their magnetic moment. Specifically, at low magnetic field and temperatures close to *T*_C_, the slope Δ*E*/Δ*B* for magnons in CrBr_3_ is predicted to be 4.5 μ_B_ and 7.1 μ_B_ for the lower energy and higher energy magnons, respectively.^131^

**Figure 9:**
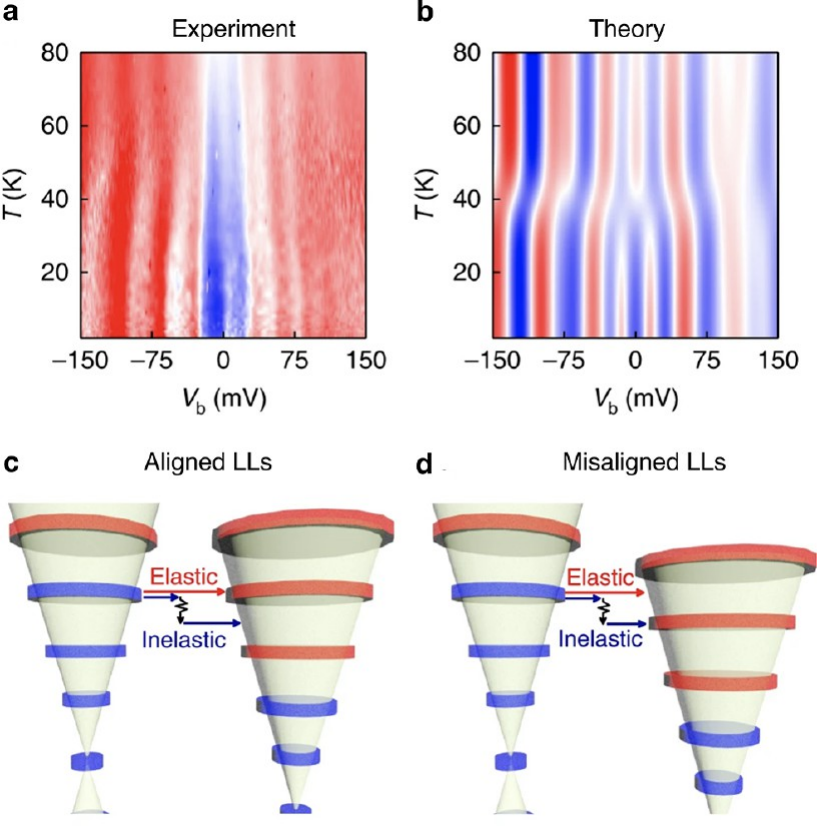
Temperature evolution of the differential conductance spectra in an out-of-plane magnetic field of 17.5 T is indicative of elastic and inelastic tunnelling processes mediated by magnons. The experimental data (a) shows qualitative agreement with theoretical predictions (b), which consider inelastic scattering processes with an emission of a magnon and elastic tunnelling processes involving two magnons. Both processes can be distinguished by applying a bias which shifts the mutual alignment of Landau levels (LLs) in the graphene electrodes (c, d). All panels adapted with permission from ref (131). Copyright 2018 Springer Nature.

Except for modulating the ordering of magnetic moments in the crystal lattice, the presence of magnons can also mediate the inter-Landau level (LL) tunnelling processes. In experiments, where the magnetic field is applied perpendicularly to the graphene electrodes, Landau quantization occurs with the mutual alignment of the LLs in bottom and top graphene electrodes given by the applied bias. Example data are presented in Figure 9a,b. In such configuration, the LLs may be tuned in and out of resonance, favoring either an elastic tunnelling process directly between aligned LLs or an inelastic tunnelling process requiring an emission of a magnon as schematically illustrated in Figure 9a,b. These two tunnelling processes may be distinguished by changing the temperature and applying bias across both graphene electrodes. At low temperature, the inelastic tunnelling events with an emission of a magnon dominate the tunnelling spectra. With the increase of the temperature, two-magnon processes^134^ involving both absorption and emission of a magnon become more probable, due to enhanced population of magnons. Such processes contribute to the elastic scattering events, which can be further enhanced by spin disorder scattering^135^ for the temperatures close to *T*_C_.

Collectively, these observations provide evidence that spin waves participate actively in the magnetic properties of 2D magnetic materials. The magnon quasiparticles contribute to tunnelling spectroscopy by opening additional channels facilitating the tunnelling processes of charge carriers. As low-energy excitations, whose contribution to the tunnelling spectra can be tuned by electric and magnetic fields, magnons constitute a foundation for the principle of operation for devices relevant for spintronics applications.^136^ Particularly, the implementation of vdW magnets in unforeseen paradigms of magnon-based data processing^137^ and computing,^138^ utilizing selective spin-wave propagation to realize alternatives for commonly used logic gates,^139^ show promising avenues for investigations.

## Magneto-Optical Phenomena

Magneto-optical phenomena develop when electromagnetic waves couple to the spin degree of freedom in solids because of spin–orbit-coupling. They have long been utilized in studying magnetic materials, more so in recent research of 2D magnetic crystals and moiré superlattices. In this section, we will review the recent progress on 2D magnetic phases in two important systems, 2D TMD heterostructures and 2D magnets (particularly, CrI_3_), studied by magneto-optical spectroscopy. Although TMDs are intrinsically nonmagnetic, magnetic states can be engineered in the strongly correlated regime in highly tunable moiré superlattices that compose of two stacked ML TMDs. In such systems, excitons in TMDs can be exploited as powerful optical probes of the emergent magnetic states or of nearby magnetic layers. On the other hand, 2D magnetic atomic layers isolated from bulk van der vdW magnetic crystals have formed another large pool of intrinsic 2D magnetic phases, of both fundamental scientific interest and potential for spintronic device applications. Static and dynamic magneto-optics (*e.g.*, MOKE, magnetic circular dichroism (MCD), magneto-PL, and magneto-Raman spectroscopy) have been applied to reveal the underlying physics of 2D magnets.

### Excitons in TMDs: Optical Probes for Emergent Magnetic Phases in van der Waals Crystals and Heterostructures

#### Magnetism in Strongly Correlated TMD Heterostructures

Strongly correlated electron systems offer a fertile ground to discover, engineer, and probe emergent phases of matter, including distinct magnetic phases. Strong correlations among electrons arise when their mutual Coulomb interaction is similar or larger than their kinetic energy, and the delicate balance between these two energy scales determine the ground state of the system and its low-energy excitations. Strongly correlated electron systems exist in many well explored material families, *e.g.* transition metal oxides,^140^ cuprate high-*T*_*c*_ superconductors,^141^ and heavy Fermion materials.^142^ A general feature of these strongly correlated materials is that the relevant electron orbitals are typically d or f orbitals which, unlike s or p orbitals, have a degree of localization and thus lead to enhanced Coulomb interaction and reduced band widths. In this picture, at an appropriate Fermi energy, localized moments with large Coulomb interaction energies arise. While these “building blocks” of strongly correlated matter are well understood, many questions remain^143^ and the road to technological exploitation is onerous. Unfortunately, conventional quantum materials typically have strict limitations to engineering and probing strong correlations and their emergent phases.

Fortunately, the rise of 2D materials provides an ability to tune the two critical energy scales (Coulomb interaction and kinetic energies), and the Fermi energy, over several orders of magnitude, providing a solid-state quantum material platform with vast potential, as already demonstrated with twisted bilayer graphene heterostructures near the magic angle.^144^ In such twisted bilayer systems, a periodic potential landscape called a moiré superlattice arises, creating a means to engineer flat-bands and quench the kinetic energy. In the case of twisted bilayer graphene, the widely tunable parameters have given rise to a plethora of phases: Mott gap insulators, superconductivity, ferromagnetism, Chern insulators, and nematic ordering, among others; more are likely to be discovered.^144^ The scope for engineering the electron correlations in 2D materials rivals the impressive precision in cold-atom quantum simulators,^145^ but with very different energy scales. Beyond magic-angle graphene, TMD heterostructures present opportunities to create and probe highly tunable electron (or hole) correlations. Compared to graphene, monolayer TMDs have a much simpler band structure. Twisted bilayer graphene has 2-fold sublattice symmetry, 2-fold layer symmetry, and 2-fold time-reversal (spin) symmetry, leading to an 8-fold total degeneracy which puts strict limitations on the twist angle (∼1.05 ± 0.05°, the so-called “magic-angle” at which strong correlations arise).^144^ On-the other hand, in twisted TMDs, the sublattice and layer symmetries are broken, resulting in simple 2-fold total degeneracy. The impact of this is significant: The strong correlations are more robust in twisted TMDs; they can be realized over a wide range of twist angles, relaxing the demands on fabrication precision and setting possibilities for larger tunability of the correlations. Also, unlike graphene, TMDs have a finite energy gap which, at the monolayer limit, becomes direct in momentum space. Combined with strong spin–orbit coupling, TMDs present clean spin-resolved optical selection rules. Finally, electron–hole pairs form strongly bound excitons in TMDs due to their 2D nature, leading to the possibility to sensitively probe their environment. Altogether, in addition to engineering strongly correlated states in low-defect density TMDs and their heterostructures, these features suggest the probing and sensing of emergent magnetic phases within the TMD itself, or in nearby 2D materials, via optical spectroscopy. This is the motivation of our section review.

In the following, we will review how emergent states arise in a Fermi sea in a monolayer TMD or in a moiré heterostructure and how the exciton transitions in TMDs can act as sensitive probes of emergent states. We will first present a basic introduction into the fundamental magneto-optical properties of monolayer TMDs. Next we will introduce emergent magnetic phases that can arise due to strong correlations in Fermionic baths, why the properties of TMDs can promote additional investigations in these topics, and recent observations in this direction with a particular focus on the properties of an exciton–polaron, a bosonic impurity in a Fermi sea, in gated TMD devices. Following this, we will introduce the 2D Hubbard model and review the recent observations of strongly correlated states in moiré TMD heterostructures. Finally, we review how monolayer TMDs can act as sensitive probes of magnetic states in nearby 2D layers.

#### Magneto-Optical Properties of TMDs

**Figure 10:**
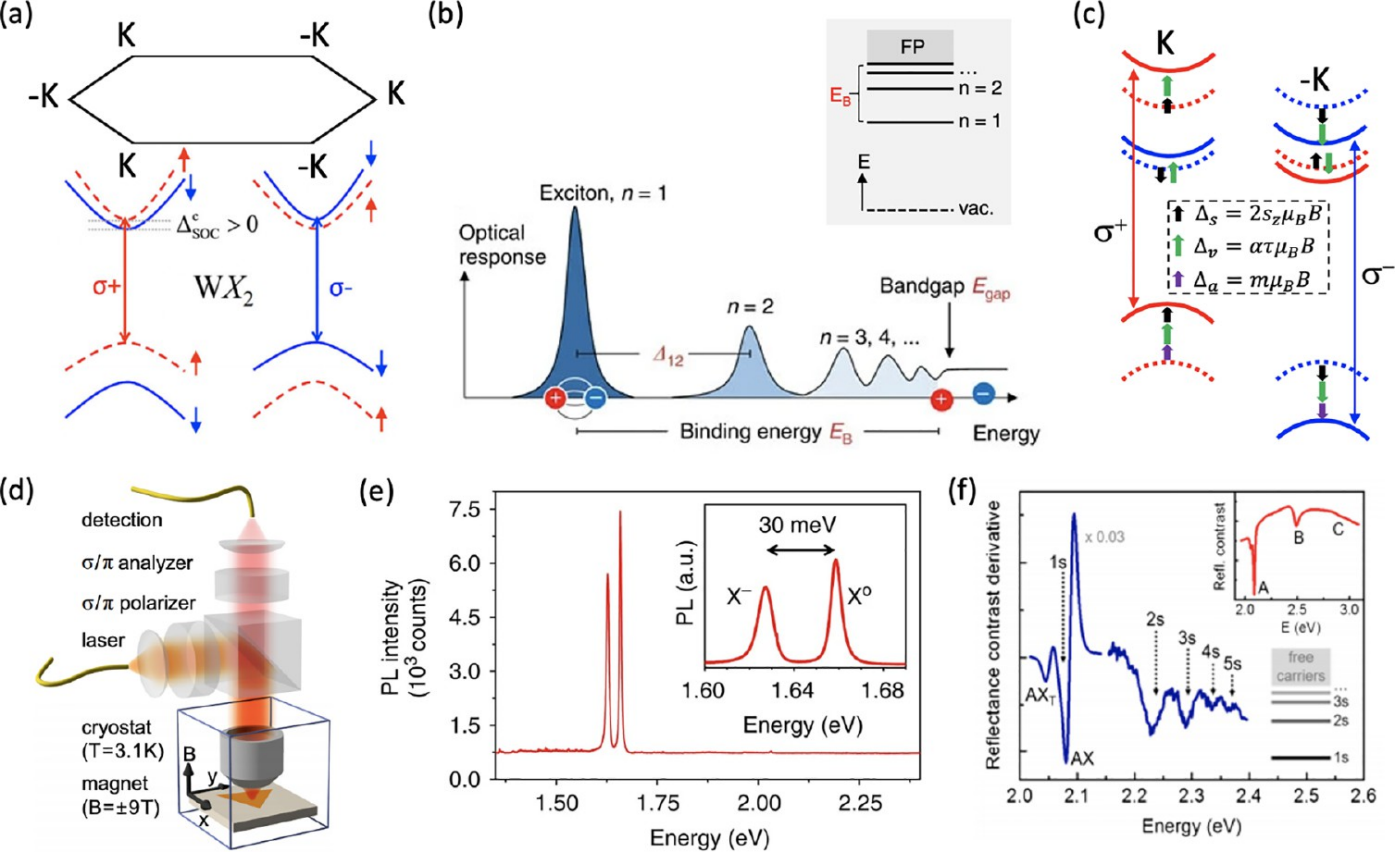
(a) Schematic illustration in a single-particle picture of the direct band gap edge states for a W-based TMD (bottom) in the degenerate but inequivalent corners of the hexagonal Brillouin zone (top). The red dashed (blue solid) lines depict spin-up (down) band-edge states. Up (down) short arrows indicate spin-up (down) conduction band and valence band electrons. Long vertical arrows represent spin-allowed optical transitions with σ^+^ (red) and σ^–^ (blue) polarization. Adapted with permission from ref (146). Copyright 2018 American Physical Society. (b) Schematic illustration of the optical response of an ideal 2D semiconductor, showing the exciton ground (*n* = 1) and excited state resonances (*n* = 2, 3, 4, ...) below the renormalized quasiparticle band gap. Adapted with permission under a Creative Commons CC BY 4.0 license from ref (147). Copyright 2017 Springer Nature. The top right inset shows the energy level scheme of the exciton states, designated by their principal quantum number *n*, with the binding energy of the exciton ground state below the free-particle (FP) band gap. Adapted with permission from ref (146). Copyright 2018 American Physical Society. (c) Schematic energy level diagram depicting the three contributions to the valley Zeeman shifts of the band-edge states: spin (black), valley (green) and atomic orbital (purple). The dashed (solid) lines represent the energies of the states before (after) applying a positive magnetic field perpendicular to the material interface. Adapted with permission from ref (148). Copyright 2015 Springer Nature. (d) Schematics of a typical microscope for optical spectroscopy of 2D materials in epifluorescence geometry. The 2D materials can be studied at temperatures *T* = 4–300 K by placing them on nonmagnetic nanopositioners inside a cryostat. A solenoid allows the application of magnetic fields (*B*) perpendicular to the crystal plane (Faraday geometry). The excitation and collection paths can feature several polarizing optical components for PL and reflectance experiments in circular (σ) and linear (π) bases. Adapted with permission from ref (149). Copyright 2017 Springer Nature. (e) PL spectrum of ML MoSe_2_ at *T* = 4 K under continuous-wave laser excitation with 2.33 eV. The spectrum shows the neutral exciton (*X*^0^) and the lower-energy negatively charged exciton (*X*^–^). The *X*^–^ shows a binding energy of about 30 meV (see inset). Adapted with permission from ref (150). Copyright 2013 Springer Nature. (f) Derivative of the reflectance contrast spectrum (*d*/*dE*) (Δ*R*/*R*) for a WS_2_ monolayer (ML). The exciton states are labeled by their respective quantum numbers. The inset shows the as-measured reflectance contrast Δ*R*/*R* for comparison. Adapted with permission from ref (151). Copyright 2014 American Physical Society.

Among the plethora of vdW materials, 2D group-VIB TMDs such as MoS_2_, MoSe_2_, WS_2_, and WSe_2_ have emerged as a class of gapped semiconductors with appealing optoelectronic properties. In their monolayer forms, they present direct bandgaps with energies in the visible to near-infrared spectral range, with the band edges located at the degenerate but inequivalent corners of the hexagonal Brillouin zone (typically referred to as K and −K valleys, as shown in Figure 10a).^146^ The combination of a strong spin–orbit coupling and broken inversion symmetry in TMDs leads to an effective coupling between the carrier spin and the valley index of the electrons and holes at the ±K corners (see Figure 10a for a W-based TMD),^152^ which results in valley-dependent optical selection rules.^153^ After absorbing a σ^±^-polarized photon, a valence band electron at ±K can be promoted to the conduction band at ±K, leaving behind a hole in the corresponding valence band. The attractive Coulomb interaction between the conduction band electron and the valence band hole gives rise to the formation of a hydrogen-like state, known as exciton, in which the electron and hole are tightly bound together with a typical binding energy on the order of 0.5 eV.^150,151,154^

As a consequence of the large binding energy, excitons determine the fundamental optical properties of TMDs at both cryogenic and room temperatures. While the radii of 2D excitons in TMDs are small, their properties remain in the Wannier-Mott regime,^146^ resulting in a Rydberg series of excited states that resembles the physics of the hydrogen atom, although with a larger sensitivity to the surrounding dielectric environment^151,154^ (see Figure 10(b)). Moreover, the carrier spin, the valley index and the atomic orbital of the band edges involved in the optical excitonic transitions are associated with a magnetic moment.^148^ Such magnetic moments can couple to external magnetic fields and break the energy degeneracy between optical transitions at ± *Kvia* the Zeeman effect (see Figure 10(c)), endowing excitons in TMDs with a large sensitivity to external magnetic fields.

Therefore, properties such as the binding energy, oscillator strength, line width, polarization, and resonance energy of excitons in TMDs represent powerful optical probes to investigate emergent phases and magnetism in TMDs and adjacent vdW materials. Among the possible experimental techniques, optical spectroscopy represents the most powerful noninvasive technique to investigate the properties of excitons in 2D TMDs.^155^ It provides access to key properties of 2D TMDs such as the crystal quality, crystal orientation, the semiconductor band gap, the exciton binding energy and the absorption strength of the material.^155^ Moreover, when combined with a confocal microscope (see Figure 10(d)), it can also provide high spatial and polarization resolution, giving access to the spin and valley physics in TMDs. In this sense, PL spectroscopy has been largely employed to study the optical properties of neutral and charged excitons in TMDs at both room and cryogenic temperatures (see Figure 10e).^150,152,154^ However, PL emission tends to favor low-energy states (especially at low temperatures), limiting the access to excited exciton states. On the other hand, reflectance spectroscopy allows to characterize the energy, oscillator strength, and line width of the ground (*n* = 1*s*) and excited (*n* = 2*s*, 3*s*...) exciton states in TMDs. For example, one-photon reflectance contrast has been used to investigate the exciton Rydberg series in monolayer (ML) WS_2_ (see Figure 10f), revealing exciton resonances from the ground 1*s* state to the 5*s* excited state.

#### Emergent States in Fermion-Doped Monolayer TMDs

Strong interactions in dilute electron systems can lead to emergent phases and magnetism.^156−158^ At very low densities, Wigner predicted that itinerant electrons will condense into an ordered array of electrons,^156^ while Bloch predicted that a paramagnetic Fermi sea of electrons can spontaneously polarize into a FM state.^158^ To achieve strong correlations, the exchange energy must dominate over kinetic energy. In an itinerant electron system, the effective strength of the electron–electron interaction is characterized by the Wigner–Seitz radius *r*_*s*_, which describes the average distance between electrons measured in units of the effective Bohr radius. In two-dimensions, , where *m*_e_* is the electron effective mass, ϵ is the dielectric constant, and *n*_*e*_ is the electron density. The kinetic energy of an electron gas scales as 1/*r*_*s*_^2^: At small *r*_*s*_ (*e.g.*, *r*_*s*_ < 1), the kinetic energy of electrons exceeds the Coulomb interaction energy and the properties of itinerant electrons can be described by Fermi liquid theory. However, Monte Carlo calculations reveal that when *r*_*s*_ ≳ 26, a two-dimensional electron system (2DES) becomes fully polarized, while for *r*_*s*_ ≳ 30, the ground state of the 2DES is a Wigner crystal,^159−161^ a lattice of ordered electrons that forms to minimize the exchange interaction energy.

A simple analysis shows that for materials with large *m*_e_* and modest ϵ, electrons in a 2DES should spontaneously arrange into a Wigner crystal at small *n*_*e*_. However, at small *n*_*e*_, carrier scattering due to intrinsic disorder in a material often dominates, making Wigner crystal formation elusive, especially in the absence of an external magnetic field (Landau quantization can also quench kinetic energy and lead to flat-bands). For example, in a 2DES in GaAs (*m*_e_* = 0.067 and ϵ = 13), *n*_*e*_ < 5 × 10^8^ cm^–2^ for *r*_*s*_ > 26, a huge challenge for defect densities in even the best MBE systems.^162^ Alternatively, 2D hole gases in GaAs (*m*_h_* = 0.4) offer better prospects, but still few reports exist and strong spin–orbit interaction complicates spin polarization measurements.^163,164^ In comparison, the weak dielectric screening (ϵ ∼ 10–30^165,166^) and large effective mass (*m*_e_* 0.3–0.8)^167,168^ of TMDs offer renewed prospects.^169^

Gate-tunable 2D TMDs and related heterostructures represent an ideal material platform for exploring and controlling strongly correlated phenomena in a 2DES. In addition to exploring itinerant magnetic phases, gate-tunable TMDs provide a leap to investigate the many-body physics problem of a bosonic impurity interacting with a Fermionic sea. On the one hand, the Fermi energy (*E*_*F*_) in ML TMDs can be electrically tuned over a wide range simply by gating, enabling a precise control of the carrier density in the semiconductor. On the other hand, the large exciton binding energy (*E*_*X*_) of these materials (on the order of hundreds of meV^150,151,154^) allows one to reach the energy regime *E*_*X*_/*E*_*F*_ ≫ 1, in which the excitons remain well-defined mobile atom-like particles (bosons) even in the presence of a substantial carrier Fermi energy. Moreover, the existence of positively and negatively charged exciton states with binding energies (*E*_*T*_) of a few tens of meV enables investigation of the strong coupling regime (*E*_*F*_/*E*_*T*_ ∼ 1), where the trion binding energy competes with the kinetic energy of the excess electrons or holes in the Fermi reservoir.

**Figure 11:**
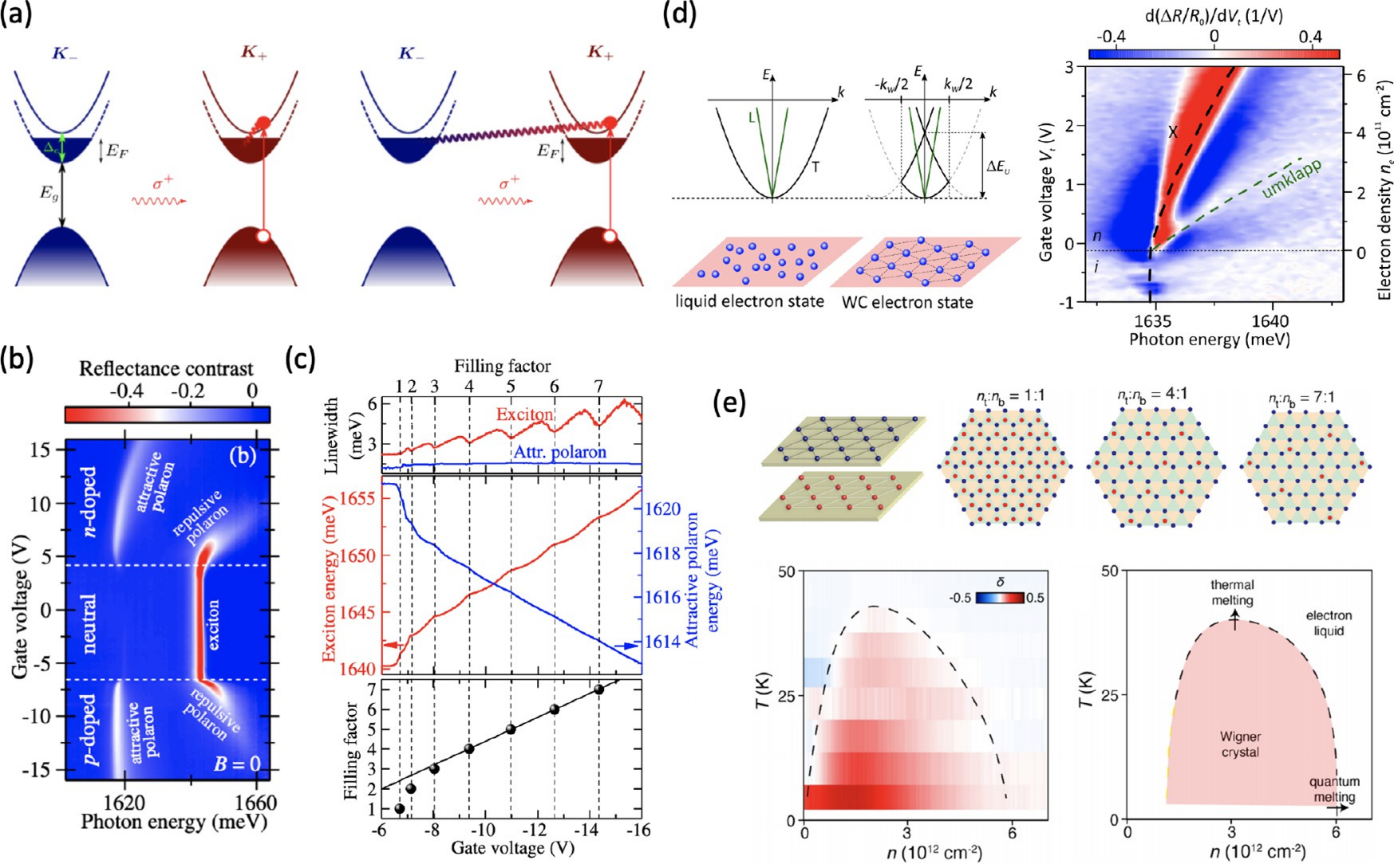
(a) Schematic band structure depicting intravalley (left) and intervalley (right) interactions between an exciton and a spin-polarized 2DES in a lightly doped W-based ML TMD. Adapted with permission from ref (170). Copyright 2020 AIP Publishing. (b) Voltage-gate-dependent color-scale map presenting reflectance contrast spectra measured in an hBN-encapsulated ML MoSe_2_. Attractive and repulsive exciton polarons are visible in both n- and p-doped regimes. Adapted with permission under a Creative Commons CC BY 4.0 license from ref (171). Copyright 2019 American Physical Society. (c) Gate-voltage dependencies of the line widths (top panel) and energies (middle panel) of the exciton (red) and attractive polaron (blue) resonances on the hole-doped side of an hBN-encapusulated ML MoSe_2_ at *B* = 16 T. Bottom panel: Gate voltages corresponding to integer filling factors determined based on the positions of the local minima of the exciton line width. Adapted with permission under a Creative Commons CC BY 4.0 license from ref (171). Copyright 2019 American Physical Society. (d) Gate-voltage-dependence derivative of the reflectance contrast spectrum with respect to the gate voltage of a charge-tunable, dual-graphene-gated, and fully hBN-encapsulated MoSe_2_ ML (right panel). The weak, higher-energy resonance is due to Umklapp scattering of the excitons off the electron Wigner crystal. Left panel: Schematics of the exciton dispersion in a ML TMD semiconductor hosting an electron system in various structural phases. The parabolic- and linear-in-momentum exciton branches arise from the splitting of the exciton branches due to the electron–hole exchange interaction, and correspond to the exciton dipole oriented along transverse (*T*) or longitudinal (*L*) directions with respect to the momentum vector, respectively. Adapted with permission from ref (172). Copyright 2021 Springer Nature. (e) Top panels: schematics of a Wigner crystal in a MoSe_2_ bilayer with an intercalated 1 nm thick layer of hBN (left). The top right panels show schematics of commensurate stacking in bilayer Wigner crystals with triangular lattices for filling ratios *n*_*t*_:*n*_*b*_ of 1:1, 4:1, and 7:1, with *n*_*t*_ and *n*_*b*_ the carrier density in the top (blue dots) and bottom (red dots) MoSe_2_ layers. Bottom panels: 2D map of δ(*n*_*t*_, *n*_*b*_) as a function of total carrier density *n* and temperature *T* for *n*_*t*_:*n*_*b*_ = 1:1 (right). The Wigner crystal forms in the region δ > 0 region. Theoretical schematic phase diagram of a bilayer Wigner crystal, showing both quantum and thermal phase transitions (left). Adapted with permission from ref (173). Copyright 2021 Springer Nature.

The many-body phenomena resulting from the interaction between an exciton and a 2DES of excess carriers in the energy range *E*_*F*_ ≤ *E*_*T*_ ≪ *E*_*X*_ can be understood in terms of Fermi polarons.^174−178^ In this framework, there are two types of exciton-electron interactions: electrons in the Fermi sea interact with excitons composed of electrons with antiparallel spin (singlet collisions) or parallel spin (triplet collisions).^174,179^ For ML TMDs doped with a 2DES with an *E*_*F*_ much smaller than the conduction band spin–orbit splitting Δ_*CB*_, the photocreated exciton can interact with the electron gas either in the same valley (antiparallel spins) or in the opposite valley (parallel spins), as sketched in Figure 11a.^170^ For antiparallel spins, these interactions split the bare exciton resonance into two branches, a low-energy state interacting attractively with the bath of Fermions (attractive polaron), and a high-energy repulsive polaron (Figure 11b).^171,175−177^ The attractive-polaron resonance can be understood as a bound state of an exciton and a Fermi-sea electron, and it is normally identified as a trion branch. The repulsive exciton–polaron branch, normally identified as an exciton branch, stems from an exciton being dressed predominately by excitations of the Fermi sea, which leads to a repulsive blue-shift of the bare exciton resonance as the number of excess charge carriers increases. On the contrary, in parallel-spin collisions, there are no bound states of the exciton–electron interaction, and the spectrum only presents a repulsive polaron branch.^174,179^

Experimental evidence of Fermi polarons in 2D semiconductors have recently been reported in the reflection and PL spectrum of intralayer excitons in gate-tunable ML TMDs.^179−182^ In addition to the formation of Fermi polarons, the strong interactions between tightly bound excitons and a dilute sea of carriers in gate-tunable TMDs have enabled the optical investigation of a wide variety of strongly correlated phenomena, including the interplay between LLs and many-body interactions in 2D semiconductors.^171,182^ For example, in ref (171), the authors carried out polarization-resolved resonant reflection spectroscopy in a gate-tunable ML MoSe_2_ hosting a dilute sea of fully spin- and valley-polarized holes in the presence of a strong magnetic field. Their results revealed filling-factor-dependent Shubnikov–de Haas-like oscillations in the energy and line width of the exciton–polaron transitions (see Figure 11c), which emerge as a consequence of the influence of Landau Level occupation on the strength of interactions between the excitons and the Fermi sea of holes. Exciton–polarons have also proved to be useful optical probes to explore interaction-induced magnetic phenomena in gate-tunable TMDs.^179,181,183,184^ In ref (181), a magnetic field of 7 T leads to a near-complete valley polarization of electrons in a gate-tunable ML MoSe_2_ with an electron density 1.6 × 10^12^ cm^–2^. By means of PL and resonant reflection measurements, they find that the Zeeman splitting of exciton–polarons can be strongly modified by interaction and phase-space filling effects, yielding effective exciton–polaron *g* factors as high as 18. These results suggest an interaction induced giant paramagnetic response of MoSe_2_. In addition to the paramagnetic phase observed for MoSe_2_, a FM ordering of 2D electrons was recently reported in gated ML MoS_2_ by optical spectroscopy measurements of exciton polarons.^179^ In ref (184), the same authors demonstrated that the magnetic phase of ML MoS_2_ can be controlled *via* the voltage applied to a gate electrode, leading to a fist-order magnetic phase transition between a FM phase at low electron density and a paramagnetic phase at high electron density.

Optical spectroscopy investigations of Fermi polarons in ML or weakly coupled BL MoSe_2_ have also recently enabled the demonstration of electronic Wigner crystallization in a 2D TMD semiconductor.^172,173,185^ As predicted theoretically,^169^ the large electron mass and reduced dielectric screening in these 2D semiconductors have led to the formation of Wigner crystals without the need of an applied external magnetic field.^172,173,185^ Moreover, even in the absence of an extrinsic periodic modulation of the band structure of the ML TMD, the charge order resulting from the Wigner crystal state gives rise to a periodic potential for the excitons in these 2D systems. In a charge-tunable ML MoSe_2_, the interaction between the periodic lattice of electrons and resonantly injected excitons has led to the emergence of resonances in the reduced excitonic Brillouin zone.^172^ The exciton resonances arise from Umklapp scattering of dark exciton states with momentum *k* = *k*_*W*_ (where *k*_*W*_ is the reciprocal lattice of the Wigner crystal), which folds the dark exciton states back to the light cone where they hybridize with the *k* = 0 exciton and thus can couple to photons (see left panel of Figure 11d). An example of these exciton resonances was observed in the high-energy side of the repulsive exciton polaron branch in a charge-tunable ML MoSe_2_ (see right panel of Figure 11d).^172^ Although both the repulsive polaron and the Umklapp resonance blue-shift upon electron doping, the energy splitting between these two resonances increases linearly with *n*_*e*_, in agreement with the reduction of the lattice constant of the Wigner crystal expected for increasing *n*_*e*_.^172^ Moreover, the same authors have shown that the application of a magnetic field further increases the stability of the Wigner crystal, since confinement of the electron motion into circular orbits partially suppresses its kinetic energy. Similar to ML MoSe_2_, resonant reflectance spectroscopy of a MoSe_2_ bilayer separated by hBN has also revealed the emergence of Umklapp exciton–polaron resonances due to spatially modulated interaction between excitons and electrons in an incompressible Wigner–Mott state.^185^ Finally, optical signatures of Wigner crystallization at cryogenic temperatures have also been observed in a nominally aligned MoSe_2_ bilayer separated by a 1 nm-thick hBN, in which robust correlated insulating states were formed at symmetric (1:1) and asymmetric (4:1 and 7:1) electron-doping ratios for the two MoSe_2_ layers (see top panel of Figure 11(e)).^173^ These bilayer Wigner crystal phases showed quantum and thermal melting transitions above a critical electron density of up to 6 × 10^12^ cm^–2^ and at temperatures of ∼40 K, as shown in the 2D plot of the dimensionless parameter δ(*n*_*t*_, *n*_*b*_) in the bottom right panel of Figure 11e for *n*_*t*_:*n*_*b*_ = 1:1 (with δ(*n*_*t*_, *n*_*b*_) ≡ (*I*_0_(*n*_*t*_, *n*_*b*_) – *I*_*t*_(*n*_*t*_)*I*_*b*_(*n*_*b*_))/*I*_0_(0, 0), *I*_0_(*n*_*t*_, *n*_*b*_) the total exciton PL intensity and *I*_*t*_(*n*_*t*_) (*I*_*b*_(*n*_*b*_)) the PL intensity of the exciton from only the top (bottom) MoSe_2_ layer when its electron density is *n*_*t*_ (*n*_*b*_)). The estimated phase boundary between an electron solid and a liquid, i.e., δ(*n*_*t*_, *n*_*b*_) = 0 (dashed line), resembles the theoretical melting curve calculated for a Wigner crystal (right panel of Figure 11e).^173^

#### Emergent States in Fermion-Doped TMD Moiré Heterostructures

**Figure 12:**
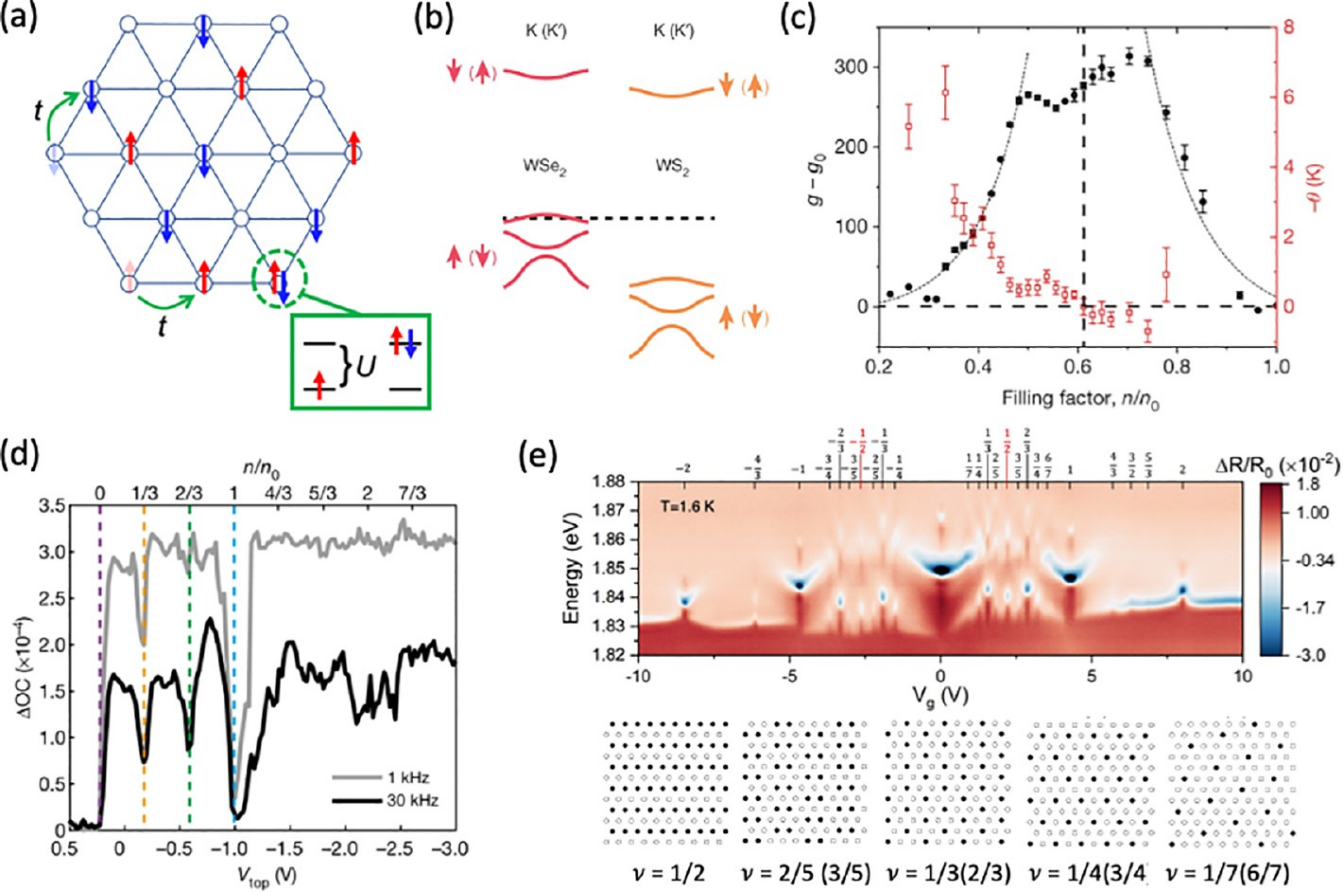
(a) Schematic illustration of a 2D triangular moiré superlattice resulting from stacking two TMD MLs with different lattice constants and/or twist angle. The moiré potentials (empty blue circles) can be loaded with either electrons or holes with spin up (red arows) or down (blue arrows). The on-site Coulomb interaction energy *U* and hopping amplitude *t* between spins in the lattice is highly tunable by the stacking angle and choice of 2D materials, enabling the investigation of the Fermi–Hubbard model. (b) Sketch of the type-II band structure of a WSe_2_/WS_2_ heterobilayer, where K and K′ represent two valley degrees of freedom. Up (down) arrows denote the spin up (down) direction. (c) Dependence of the magnetic susceptibility χ ∝ *g* – *g*_0_ (left axis, black filled symbols) and Weiss constant θ (right axis, red empty symbols) on the filling factor of WSe_2_/WS_2_ at 1.65 K. Panels (b) and (c) adapted with permission from ref (197). Copyright 2020 Springer Nature. (d) Optically detected resistance and capacitance signal at 1 kHz (gray) and 30 kHz (black) from charge-neutral to moderate hole doping in WSe_2_/WS_2_, showing gap-like features at hole doping levels of *n* = *n*_0_/3 (orange dashed line), *n* = 2*n*_0_/3 (green dashed line) and *n* = *n*_0_ (blue dashed line). Adapted with permission from ref (198). Copyright 2020 Springer Nature. (e) Abundance of insulating states in WSe_2_/WS_2_ as revealed by the blue-shifts of the 2*s* exciton resonance in the reflectance contrast of a ML WSe_2_ sensor placed in close proximity to the heterobilayer (top panel). The top axis shows the proposed filling factor for the insulating states, with the corresponding configurations schematically shown in the bottom panels. Adapted with permission from ref (199). Copyright 2020 Springer Nature.

A simple model to capture correlated electron phenomena in crystals is to extend the tight-binding model, in which all electron hopping processes have a kinetic energy −*t*, by introducing an on-site Coulomb interaction energy *U*,^186−188^ as depicted in Figure 12a). This is the Hubbard model. The Hubbard Hamiltonian is , with adjacent site indexes *i* and *j*, spin index σ,  (*c*_*iσ*_) the operator to create (destroy) an electron of spin σ on lattice site *i*, and  the number operator. The first term describes the kinetic energy, the second the interaction energy, and the third the chemical potential which controls the filling factor (ν). The situation where the filling is one electron per site is typically refereed to as half-filling factor (ν = 1/2).

Similar to the emergence of strong correlations in a 2DES, the exchange interaction dominates over the kinetic energy, when *U*/*t* ≫ 1 strong correlations emerge in the Hubbard model. Because of the simplicity of the model, it provides valuable insights into emergent phases, including insulating, magnetic, and superconducting effects in quantum materials. For instance at ν = 1/2, it can be shown that a Mott insulator emerges, which is important in the context of high-temperature superconductors.^143^ This result is simple conceptually: at ν = 1/2 an electron can only hop to a site if it is already occupied. This costs an energy *U*. Hence, an energy gap *U* opens up at ν = 1/2, creating a Mott insulator. However, uncovering the magnetic phase of the Mott insulator becomes nontrivial. In general, the Hubbard model is nontrivial to solve in two- or three-dimensions, and a wide range of techniques (mean field or field theory approaches or numerical approaches such as diagonalization or quantum Monte Carlo) have been used. Experimentally probing the model is also challenging, due to the limited range of parameter control in conventional quantum materials. Motivated by this, quantum simulation of the Hubbard model with ultracold Fermions in optical lattices has materialized, with length scales on the order of 1 μm,^189^ compared to angstrom-scale length scales in the solid-state.

The 2D moiré superlattices formed by stacking two ML TMDs together provide an approach to create triangular Hubbard model simulators with an impressive range of tunability.^190^ The length scale, on the order of 10 nm, is precisely tunable by the combination of lattice mismatch and rotation angle between the two layers, while the filling factor can easily be tuned from ν < −1 to ν > 1 (hole-doping to electron doping), creating a straightforward means to tune *U*/*t*. Further, the choice of TMD material combinations (including homobilayers or heterobilayers) allows tuning the moiré potential depths, which can strongly affect the phase diagram. Theoretical work to understand the emergent phases and their dependence on the wide range of parameters is only just beginning.^191−196^

Equally, there has been an explosion in experimental efforts recently, with several early efforts investigating gated WSe_2_/WS_2_ moiré superlattices.^197−201^ In these initial works, the two TMD monolayers are closely aligned (∼0 or 60° relative twist), such that the moiré periodicity (∼8 nm) is largely determined by the lattice mismatch (∼4%). These heterostructures feature type II band-alignment and flat-bands (Figure 12b). With this system, Tang *et al*. characterized the phase diagram of strongly correlated holes as a function of ν, observing a Curie–Weiss behavior in the temperature dependence of the exciton Zeeman splitting (see Figure 12c). Interestingly, it was observed that the Weiss constant changes sign from negative to positive around ν = −0.6, consistent with a quantum phase transition from AF to FM ordering. Tang *et al*. also observed the Mott (or possibly charge transfer)^191^ insulating state at ν = 0.5 in a transport experiment and correlated this with an observation of signatures of charge-ordering in the differential reflectivity of the intralayer excitons as a function of ν.^197^ Regan *et al*. developed an optical technique to detect charge-ordered states in the flat valence-band at ν = −1/6, −1/3, and −1/2.^198^ The ν = −1/6 and −1/3 states likely represent Wigner crystals, while the ν = −1/2 state is the Mott or charge transfer insulator. Further, low-energy spin dynamics in the charge ordered states were observed using a pump–probe experiment to probe circular dichroism resulting from spin-ordering.^198^

Building on these initial observations, WSe_2_/WS_2_ moiré heterostructure device, with a nearby ML WSe_2_ sensor, was used to discover a number of correlated states at commensurate filling fractions. The charge ordering of these states is symmetric about ν = ± 1/4 and are proposed to range from generalized Wigner crystals to charge density waves (see Figure 12e).^199^ This stunning observation highlights the versatility to engineer and the power to optically probe emergent states in TMD moiré heterostructures. Jin *et al*. have recently characterized in-depth the properties of these strongly correlated states by combining optical anisotropy and electronic compressibility measurements.^200^ They find a strong electrical anisotropy, maximum at ν = 1/2, which is assigned to an insulating stripe phase. Further, wide-field imaging of the stripe phase domains reveals preferential alignment along the high-symmetry axis of the moiré superlattice. These results provide insight into the phase diagram of the extended triangular Hubbard model. Finally, recent results highlight the possibility to observe correlated states at fractional fillings in the PL emission of interlayer excitons.^201^ Interlayer excitons, Coulomb bound electrons and holes spatially separated in different monolayers, arise in TMD heterobilayers due to the type-II band alignment. In contrast to optical signatures of emergent phases in the reflectance contrast of intralayer moiré excitons, interlayer exciton PL provides access to the valley (K and −K) dynamics of excitonic states in the moiré superlattice. First indications are that the fractional states, with their insulating nature, can also enhance the intervalley scattering and suppress the valley polarization.^201^

#### TMDs as Sensors of Emergent States

**Figure 13:**
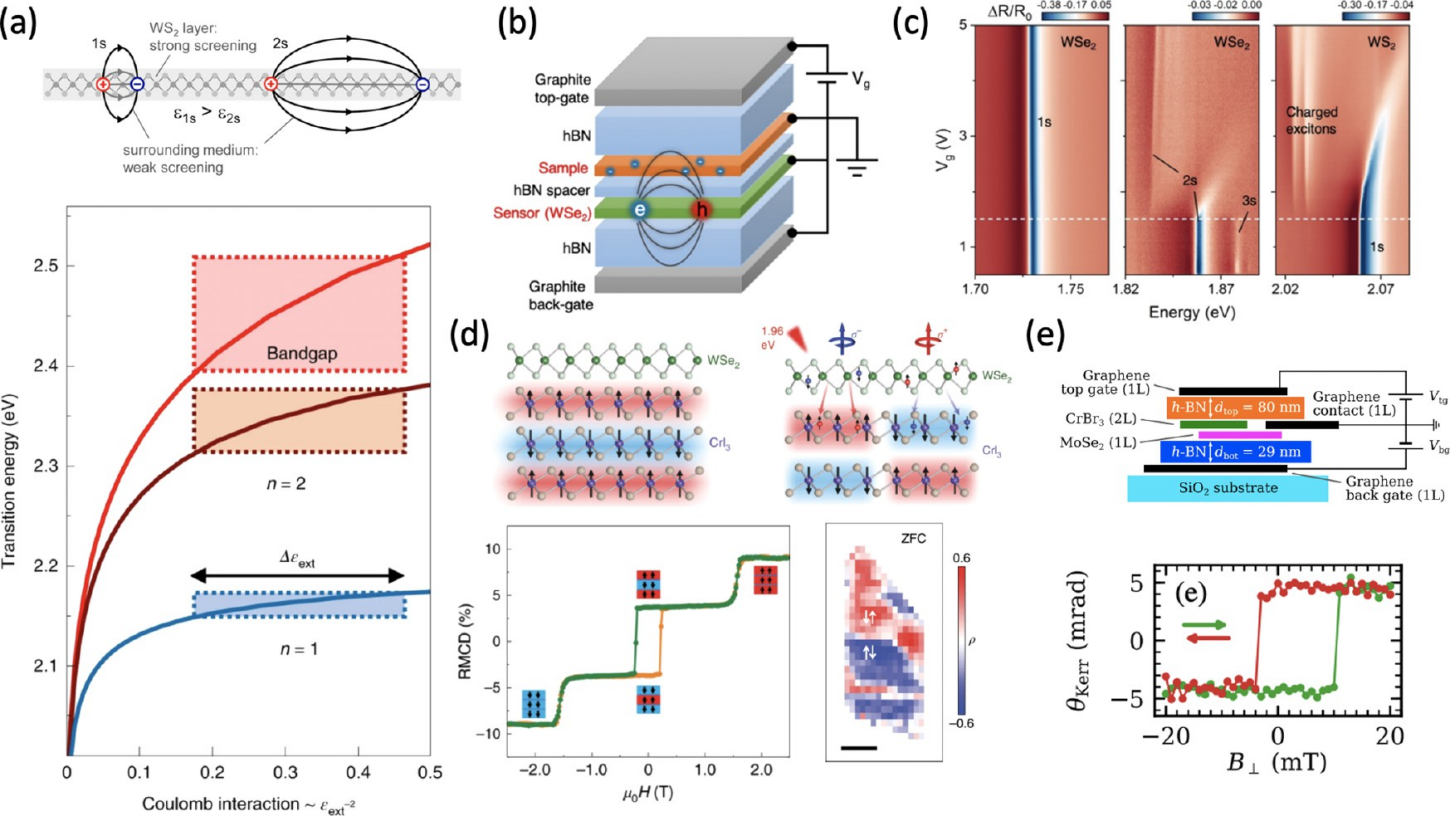
(a) Top: Schematic illustration of electron–hole pairs forming 1*s* and 2*s* excitonic states in a 2D dielectric slab. Adapted with permission from ref (151). Copyright 2014 American Physical Society. Bottom: Theoretically calculated energies of the bandgap and exciton states in a WS_2_ ML as a function of inverse squared external dielectric constant. Shaded areas indicate fluctuations from variations of the external screening. Adapted with permission from ref (202). Copyright 2019 Springer Nature. (b) Schematic of a device heterostructure to demonstrate the sensing capabilities of excitons in ML WSe_2_. (c) Reflectance contrast of the 1*s* (left) and 2*s* and 3*s* (middle) excitonic transitions in the WSe_2_ sensor, and the 1*s* exciton in the WS_2_ sample as a function of the applied gate voltage (*i.e.*, the electron concentration in WS_2_). (b) and (c) Adapted with permission from ref (199). Copyright 2020 Springer Nature. (d) Left: Reflective magneto-circular dichroism as a function of magnetic field (bottom) measured in a monolayer WSe_2_ and trilayer CrI_3_ heterostructure, depicted above schematically. Orange and green curves represent magnetic field sweeping up (increase) and down (decrease), respectively. Right: Schematic of a ML WSe_2_ and bilayer CrI_3_ heterostructure (top). The layered AF spatial domains that are indistinguishable by reflective magneto-circular dichroism can be resolved by circular polarization-resolved PL from WSe_2_ (bottom). Adapted with permission from ref (203). Copyright 2020 Springer Nature. (e) Measurement of the CrBr_3_ magnetization hysteresis using the MOKE (bottom) in a device like schematically shown on top. Adapted with permission under a Creative Commons CC BY 4.0 license from ref (204). Copyright 2020 American Physical Society.

The 2D nature of ML TMD semiconductors endows excitons in these systems with properties that differ fundamentally from those of the corresponding bulk semiconductor. A particular property of excitons in 2D semiconductors such as ML TMDs is that the electric field lines joining the bound electron–hole pairs (which are strongly confined to the plane of the atomically thin ML) extend outside the 2D semiconductor slab (see top panel of Figure 13a.^151^ This property makes excitonic transitions in ML TMDs very sensitive to their surroundings, since moderate changes of the local dielectric permittivity in the vicinity of the ML lead to dielectric-induced renormalizations of both the electronic band gap and the exciton binding energy by hundreds of meV.^147^ The bottom panel of Figure 13a summarizes these effects for ML WS_2_, a typical 2D TMD. The figure shows the theoretically calculated energies of the band gap and both the ground and first excited exciton states of ML WS_2_ (denoted by their principal quantum numbers of *n* = 1 and 2, respectively) as a function of the inverse squared external dielectric constant.^202^ Such sensitivity of excitonic transitions in 2D TMDs to their dielectric environment has been exploited to realize an in-plane dielectric heterostructure with a spatially dependent bandgap^147^ and to monitor the dielectric disorder in semiconducting nanostructures with micrometer spatial resolution.^202^

In addition to the previously mentioned applications, the sensing capabilities of ML TMDs can also be harnessed to explore the emergence of correlated many-body states in vdW heterostructures. Xu *et al.* have recently unveiled an abundance of correlated insulating states at fractional fillings in a WSe_2_/WS_2_ moiré superlattice^199^ by optically probing the resonance energy and oscillator strength of the exciton excited states of a ML WSe_2_ sensor placed in close proximity to the TMD heterobilayer. Figure 13b shows a sketch of the device employed by Xu *et al.*, in which a WSe_2_ ML was separated from a WSe_2_/WS_2_ heterobilayer by an hBN spacer with a thickness of 1 nm.^199^Figure 13c shows a proof-of-concept example of the sensing technique in a control device similar to the one depicted in Figure 13b, in which the TMD heterobilayer was replaced by a WS_2_ ML. This figure shows the reflectance contrast of the excitonic transitions of both the WSe_2_ sensor and the WS_2_ sample as a function of the applied gate voltage (*i.e.*, the electron concentration in WS_2_). While the energy of the 1*s* excitonic transition in the WSe_2_ sensor shows negligible dependence with the applied gate voltage (left panel), the behavior of the 2*s* and 3*s* charged exciton states in the WSe_2_ sensor (middle panel) mirrors that of the 1*s* exciton in the WS_2_ sample (right panel). These results not only demonstrate the potential of the optical sensing technique enabled by excitons in TMDs but also highlight the further sensing capabilities of the exciton excited states (*n* = 2, 3) compared to the ground state (*n* = 1). The origin of the better sensing capabilities of the excited excitonic transitions is two-fold: First, the exciton excited states (2*s*, 3*s*, and so on) have Bohr radii many times the ML thickness, which allows them to sense the dielectric permittivity of nanostructures placed a few nm away from the layer. In contrast to the excited states, the Bohr radius of the ground exciton state is typically of the order of the ML thickness,^168^ which restricts the dielectric sensing to layers in direct contact with the sensor. Second, as shown in the bottom panel of Figure 13a for ML WS_2_, the excited-state excitonic resonances follow the external screening-induced variations of the band gap to a much larger extent,^202^ which results in a larger sensitivity to small variations of the local dielectric environment.

The potential of ML TMDs as atomically thin sensors for emergent correlated and magnetic phases in vdW materials and related heterostructures is not restricted to detecting variations in their dielectric surroundings. Both the proximity-induced exchange interaction and the charge transfer between vdW ferromagnets and ML TMD semiconductors can also be exploited to probe the magnetic phases of the atomically thin magnetic materials.^203−205^ An interface between a vdW ferromagnet such as CrI_3_ or CrBr_3_ and a TMD semiconductor, such as MoSe_2_ or WSe_2_, results in a type-II band structure alignment, where the lowest conduction band is in the magnetic material.^203^ Since the relevant conduction bands in CrI_3_ and CrBr_3_ are spin polarized, the type-II band structure leads to a spin-dependent charge transfer between the TMD and the 2D ferromagnet, resulting in a large spontaneous exciton valley-spin polarization in the TMD.^203^ The top left panel of Figure 13d shows the sketch of a ferromagnet/TMD vdW heterostructure in which a WSe_2_ ML was employed to monitor the layered antiferromagnetism of a trilayer CrI_3_.^203^ By measuring the reflective magneto-circular dichroism of ML WSe_2_ as a function of applied magnetic field at 15 K, the authors where able to observe three transitions in the magnetization of CrI_3_ (see bottom left panel of Figure 13d), with each transition corresponding to a flip in the magnetization of a single CrI_3_ layer. Moreover, the fast spin-polarized electron transfer between the CrI_3_ layer in contact with the WSe_2_ ML also enabled the use of WSe_2_ as a magnetic sensor to map out layered AF/FM spatial domains at zero magnetic field as well as at finite magnetic fields in bilayer CrI_3_/WSe_2_ heterostructure (see right panels of Figure 13d).

Finally, in 2D ferromagnet/TMD heterostructures, the 2D ferromagnet also induces a magnetic exchange field (proximity effect) in the adjacent TMD, which gives rise to a sizable valley Zeeman splitting for excitonic transitions in the TMD.^204−207^ Such proximity-induced magnetic effect has also been exploited to monitor the FM properties of CrBr_3_ (see Figure 13e).^204,208^

### Raman Spectroscopy of 2D Magnetism: Exploring Lattice, Spin, and Charge Interactions

#### Magnetism in 2D Magnetic Atomic Crystals

Recently, a long-sought-after member of 2D material family, 2D magnetic atomic crystals, have been discovered in several mechanically exfoliated vdW magnetic materials, including both FM^6,12,14,77,209^ and AF^32,210−212^ ones.^12,213,214^ Among the 2D magnets revealed so far, CrI_3_ is of particular interest and extensively explored, because its layer-dependent magnetic states in ultrathin flakes can be controlled by external magnetic field,^13−16,120^ electric field,^8,9^ electrostatic doping,^11^ and hydrostatic pressure,^22^ which immediately triggered tremendous interest in employing 2D magnetism in spintronics applications such as spin filters^13−16,120^ and transistors.^8,9,11^

Optical spectroscopy has played an unmissable role in the discovery and exploration of 2D magnetism. MOKE and MCD have provided direct experimental evidence for 2D FM orders in mono- to few-layer CrI_3_,^14^ MnBi_2_Te_4_,^209^ Cr_2_Ge_2_Te_6_,^6^ and Fe_3_GeTe_2_.^77^ Spontaneous helical PL has shown the impact of FM on the electronic states in 2D magnetic semiconductors such as CrI_3_.^215^ Giant nonreciprocal second harmonic generation (SHG) has also shown the outstanding magnetic contribution to the nonlinear optical effects in noncentrosymmetric 2D magnetic states such as even number-of-layer CrI_3_^24^ and MnPS_3_.^216^ Raman spectroscopy in fact has provided the earliest, although indirect, experimental signatures of zone-folded phonons in the search of 2D AF orders in (Fe, Mn, Ni)PS_3_^32,210−212^ and has revealed the anomalous magneto-Raman effects in layered AF materials of CrI_3_^217−224^ and VI_3_.^225^

Comparing to the static optical probes of MOKE, MCD and magnetic SHG that primarily focus on the broken time-reversal and/or spatial point symmetries of magnetic orders, Raman spectroscopy takes the dynamic perspective in examining 2D magnetic materials, probing collective excitations including phonons in CrI_3_, VI_3_, Fe_3_GeTe_2_, Cr_2_Ge_2_Te_6_, and (Fe, Mn, Ni)PS_3_, magnons in CrI_3_, VI_3_, and (Fe, Mn, Ni)PS_3_, polarons in CrI_3_, *etc*., identifying the symmetry properties of both the crystallographic and magnetic structures, and resolving the coupling among lattice, charge, and spin degrees of freedom such as electron–phonon coupling and spin-phonon coupling. Comparing to the dynamic probe of helical PL that results from the carrier recombination across the semiconducting gaps (∼1 eV energy scale), Raman spectroscopy focuses on low-energy (∼2–3000 cm^–1^) collective excitations from the ordering of lattice, spin, and charge and works well for metallic, semiconducting, and insulating 2D magnetic materials.

In the following, we review three types of phonon-related collective excitations, using specific examples in bulk and few-layer CrI_3_. First, we look at the conventional phonons in CrI_3_ and show its evolution across the magnetic-field-induced magnetic phase transition. Second, we discuss the anomalous magneto-Raman effect reported in CrI_3_ by multiple groups^217−224^ and settle down its origin as the static layered AF order coupled with finite-momentum phonons. Third, we review the Raman scattering between phonon-dressed exciton states in CrI_3_ and show its response across thermal and magnetic-field-induced magnetic phase transitions. We have reserved the discussion on magnons in CrI_3_ for the Heterostructures, Twisted Layers, and Interfaces section.

#### Phonons and Structural Phase Transitions in CrI_3_

**Figure 14:**
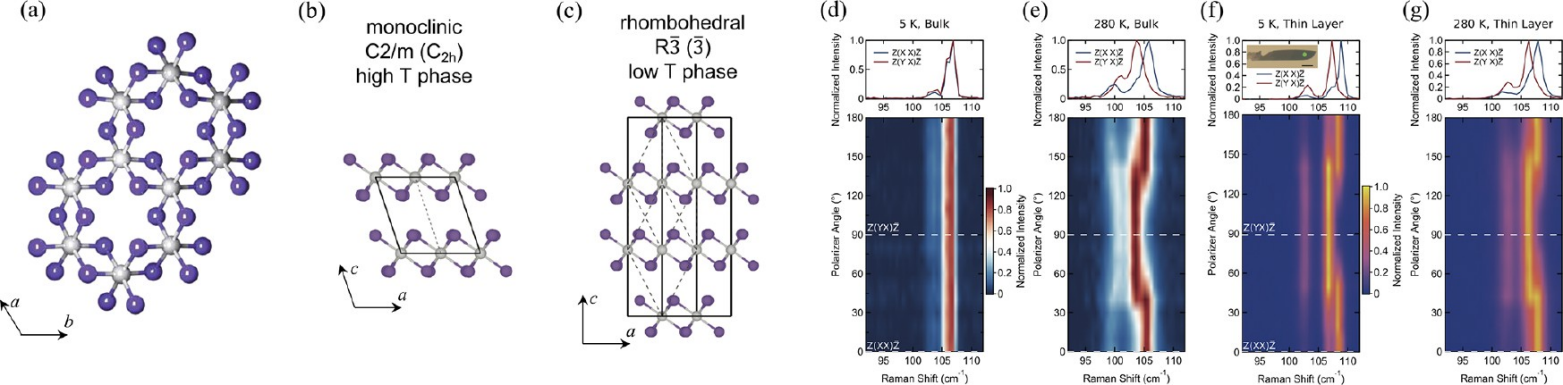
(a) Top view of CrI_3_ monolayer with gray and purple spheres representing Cr and I atoms. Adapted with permission from ref (5). Copyright 2017 Springer Nature. (b, c) Lateral view of bulk CrI_3_ in the monoclinic (b) and rhombohedral (c) phase. (d, e) Polarization resolved Raman spectra for bulk CrI_3_ taken at 5 K (d) and 280 K (e). (f, g) Polarization resolved Raman spectra for thin layer CrI_3_ taken at 5 K (f) and 280 K (g). Panels (b–g) adapted with permission under a Creative Commons CC BY license from ref (224). Copyright 2019 IOP Publishing.

Figure 14a shows the top view of the CrI_3_ monolayer. The Cr^3+^ cations form a honeycomb structure with edge-sharing octahedral coordination formed by six *I*^–^ anions. In bulk CrI_3_, the as-grown CrI_3_ single crystal has a monoclinic structure (space group *C*2/*m*, point group *C*_2*h*_) at room temperature, with the adjacent layers shifted along the *a*-axis direction by 1/3 lattice constant (Figure 14b), and undergoes a first-order structural phase transition to a rhombohedral structure (space group *R*3̅, point group *C*_3*i*_) at a critical temperature *T*_C_ ∼ 200–220 K, with the layers stacked in the ABC sequence (Figure 14c). Unlike bulk CrI_3_, few-layer CrI_3_ does not experience such a structural phase transition even when the temperature is lowered to 5 K, suggesting that the monoclinic structure persists down to low temperature of 5 K.

Raman spectroscopy has been used to track the temperature dependent structural phase transition in bulk CrI_3_^218,226^ and to identify the monoclinic structure of few-layer CrI_3_.^224^ Below *T*_*S*_, the Raman scattering off the rhombohedral lattice (*C*_3*i*_) reveals phonon modes of *A*_*g*_(*C*_3*i*_) and *E*_*g*_(*C*_3*i*_) symmetries with corresponding Raman tensors of the form
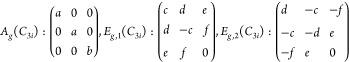
Above *T*_*S*_, the Raman active phonons of the monoclinic structure are of *A*_*g*_(*C*_2*h*_) and *B*_*g*_(*C*_2*h*_) with Raman tensors of the form
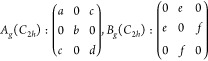


The difference in the Raman response between the two phases contains two aspects. First, one would expect the selection rule difference in the *A*_*g*_ phonons which is isotropic and only present in the linearly parallel channel for *A*_*g*_(*C*_3*i*_) but anisotropic and could appear in both linearly parallel and crossed channels for *A*_*g*_(*C*_2*h*_). Second, one could observe the degeneracy lift from the doubly degenerated *E*_*g*_(*C*_3*i*_) phonons into the nondegenerate *A*_*g*_(*C*_2*h*_) and *B*_*g*_(*C*_2*h*_) phonons. While the interlayer stacking induced phase transition is hardly reflected in the selection rule of *A*_*g*_ phonons, it is well captured by the degeneracy lift of the *E*_*g*_(*C*_3*i*_) phonons. Figure 14d,e shows the polarization dependence of the *E*_*g*_(*C*_3*i*_) modes at 5 K and the *A*_*g*_(*C*_2*h*_) and *B*_*g*_(*C*_2*h*_) phonons at 280 K in bulk CrI_3_, clearly showing the mode splitting and the selection rule symmetry reduction for the case at 280 K. Same polarization-dependent measurements on CrI_3_ thin flakes, however, show no distinct behaviors at 5 and 280 K (Figure 14f,g) and resemble that of bulk CrI_3_ in the monoclinic phase above *T*_*S*_ (Figure 14e), which confirms the monoclinic structure down to the 5 K in few-layer CrI_3_.

In addition to the structural evolution as a function of the temperature and thickness, CrI_3_ undergoes a magnetic phase transition across a critical temperature of *T*_*c*,3*D*_ = 61 K for bulk CrI_3_ and *T*_*c*,2*D*_ ∼ 45 K for 2D flakes. The 2D magnetic phase in few-layer CrI_3_ has been shown to be a layered AF state where the *S* = 3/2 spins at the Cr^3+^ sites align ferromagnetically within the layers and antiferromagnetically between adjacent layers.^14^ The 3D bulk magnetic phase was believed to be a FM state within and across layers^227^ and has been recently revised to a hybrid surface layered AF and deep bulk FM state.^218^ The difference of the interlayer magnetic exchange coupling between few-layer 2D (and surface of 3D) and deep bulk 3D CrI_3_ has been attributed to the stacking difference, *i.e.*, monoclinic for interlayer AF and rhombohedral for interlayer FM.^228^ When an external out-of-plane magnetic field is applied, a layered AF to FM phase transition across critical fields of *B*_*c*_ has been observed for both few-layer and bulk CrI_3_.^14^ One natural question would be how the crystal structure responds to this magnetic field-induced layered AF to FM magnetic phase transition, which can be addressed by tracking the magnetic field dependence of the phonon modes.

**Figure 15:**
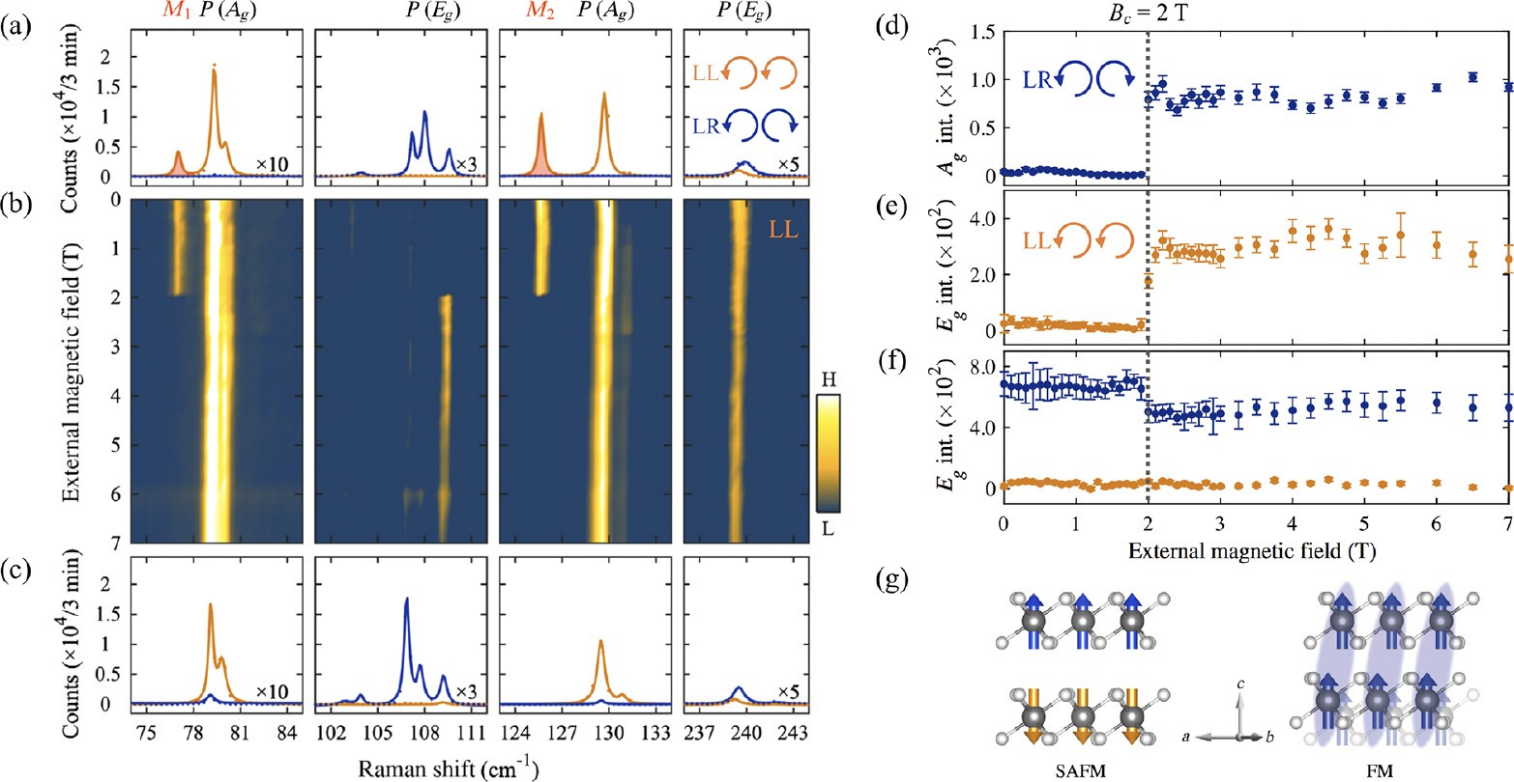
(a) Raman spectra taken on bulk CrI_3_ in LL and LR channels at 10 K and 0 T. P(*A*_*g*_), P(*E*_*g*_), and M_1,2_ label phonon modes of *A*_*g*_, *E*_*g*_ symmetry and coupled with layered magnetism, respectively. (b) Color map of magnetic field dependent Raman spectra taken in the LL channel. (c) Raman spectra taken on bulk CrI_3_ in LL and LR channels at 7 T. (d–f) Magnetic field dependence of selected *A*_*g*_ and *E*_*g*_ phonon modes. (g) A schematic illustration of the monoclinic distortion across the layered surface AF to FM transition. Adapted with permission under a Creative Commons CC BY 4.0 license from ref (218). Copyright 2020 American Physical Society.

Figure 15a,c shows Raman spectra in the circularly parallel (LL) and crossed (LR) channels taken at 10 K and 0 and 7 T in bulk CrI_3_, with L(R) standing for left-(right-)handed circularly polarized light. Consistent with the Raman tensors of the rhombohedral crystal structure, *A*_*g*_ and *E*_*g*_ phonon modes at 0 T are observed exclusively in the LL and LR channels, respectively, while another two time-reversal symmetry broken modes (labeled as M) with antisymmetric Raman tensor of the form  is only present in the LL channel. The magnetic field dependence of Raman spectra in the LL channel is displayed in the false color map in Figure 15b, showing abrupt changes in the phonon intensities at a critical field *B*_*c*_ = 2 T corresponding to the surface layered AF to FM phase transition. The phonon intensities can be extracted from the Raman spectra in both channels and plotted against the applied external fields (Figure 15d–f). Three types of phonon selection rule evolution support a monoclinic structural distortion above *B*_*c*_ (shown in Figure 15d–f). The first type is the leakage of *A*_*g*_ phonons (*e.g.*, at ∼129 cm^–1^) into the originally forbidden LR channel at *B* > *B*_*c*_ (Figure 15d), corresponding to the Raman tensor form changing from  to  and corroborating with the monoclinic structural distortion. The second type corresponds to the appearance of *E*_*g*_ phonons (*e.g.*, at ∼109 cm^–1^) in the LL channel at *B* > *B*_*c*_ (Figure 15e), reflecting the evolution from *E*_*g*_(*C*_3*i*_) into *A*_*g*_(*C*_2*h*_) upon a monoclinic distortion. The third type features the *E*_*g*_ phonons remaining in its original LR channel but experiencing an abrupt change in intensity (Figure 15f), corresponding to the transformation of *E*_*g*_(*C*_3*i*_) into *B*_*g*_(*C*_2*h*_) across a monoclinic structural phase transition (Figure 15g).^218^

It remains to be determined thus far whether this monoclinic structural distortion is also present in few-layer CrI_3_ and how to understand this structural monoclinicity in the magnetic field-induced FM state in the context of monoclinic structure leading to layered AF at zero field. Nonetheless, ordinary phonons probed by Raman spectroscopy are sensitive indicators of structural changes and can be exploited in studying lattice-magnetism coupling in 2D magnetic systems beyond CrI_3_.

#### Time-Reversal Symmetry Broken Static Layered Antiferromagnetic Coupled Phonons in CrI_3_

**Figure 16:**
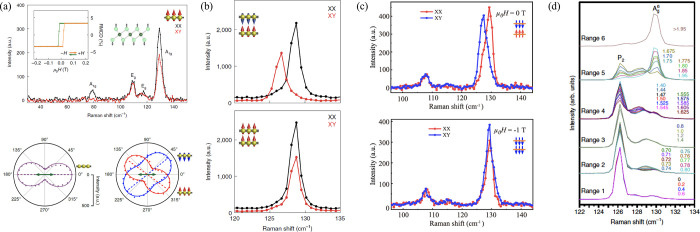
(a) Raman spectra taken in both linear parallel and crossed channels in monolayer CrI_3_ at 15 K and 0 T. Polar plots of polarization dependent Ag mode intensity (∼129 cm^–1^) at 60 and 15 K, above and below the magnetic onset temperature *T*_*C*_ = 45 K, respectively. (b) Raman spectra of 2L CrI_3_ at 15 K and at 0 and 1.5 T in linearly parallel and crossed channels. Panels (a, b) adapted with permission from ref (221). Copyright 2020 Springer Nature. (c) Raman spectra of 2L CrI_3_ at 1.7 K and at 0 T and −1 T in linearly parallel and crossed channel. Adapted with permissions from ref (222). copyright 2020 American Chemical Society. (d) Raman spectra of 10L CrI_3_ at 9 K and at selected magnetic fields. Adapted with permission under a Creative Commons CC BY license from ref (223). Copyright 2020 Springer Nature.

In few-layered CrI_3_, a time-reversal symmetry broken phonon mode with an antisymmetric Raman tensor has been observed in the close proximity to a fully symmetric *A*_*g*_ phonon mode at ∼129 cm^–1^. Moreover, it introduces exceptionally large polarization rotation in monolayer CrI_3_ (Figure 16a), shows distinct magnetic field dependence from the *A*_*g*_ phonon in bilayer (2L) CrI_3_ (Figure 16b,c), and exhibits more complex Raman spectral shape and magnetic field dependence in thicker CrI_3_ flakes (Figure 16d), therefore generally referred as anomalous magneto-Raman effect.^221−223^

**Figure 17:**
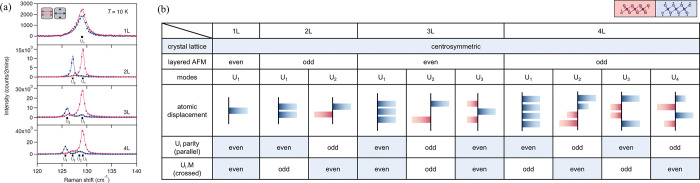
(a) Raman spectra taken on 1–4L CrI_3_ in both linear parallel and crossed channels at 10 K and 0 T. Adapted with permission under a Creative Commons CC-BY license from ref (219). Copyright 2017 National Academy of Sciences. (b) A summary of linear chain model calculation results and selection rule analysis for 1–4L CrI_3_.

A layered magnetism-coupled phonon scattering mechanism, in combination with Davydov splitting, is proposed to account for this observed anomalous magneto-Raman effect in 2D CrI_3_ of arbitrary thickness. Figure 17a shows Raman spectra taken on 1–4L CrI_3_ in both linearly parallel and crossed channels at 10 K and 0 T. One can see three prominent features in these layer-number dependent Raman spectra: First, the total number of phonons increases proportionally to the number of layers; second, the highest frequency remains constant while the lowest frequency decreases monotonically at higher layer numbers; and third, the parallel and crossed channels select the same (distinct) frequency mode(s) in CrI_3_ with odd(even) number of layers. A simple linear chain model of *N*-layer CrI_3_ of Hamiltonian, , is used to compute the Davydov splitting of the single *A*_*g*_ phonon mode into *N* phonon multiplets (*i.e.*, *N* eigenvalues and *N* eigenvectors), where *u*_*i*_ represents the displacement field in the *i*th layer, and *k*_0_ and *k* stand for the coupling constant within each layer and between adjacent layers, respectively. Among these *N* eigenvectors  for *N*-layer CrI_3_, the highest frequency mode  (*i.e.*, *i* = 1) features the in-phase atomic displacement of the *A*_*g*_ phonon of monolayer across all *N* layers and is even under spatial inversion symmetry operation (*i.e.*, parity even) whereas the lowest frequency mode  (*i.e.*, *i* = *N*) has out-of-phase atomic displacement between adjacent layers and is parity even (odd) for odd (even) *N* (see Figure 17b). For this, one would expect that the frequency of  keeps unchanged (decreasing) with increasing *N* because its in-phase (out-of-phase) atomic displacement saves no (most) energy from the interlayer coupling.

In the linearly parallel channel, one expects the ordinary phonons with symmetric Raman tensor (*R*_*S*_) of parity even *A*_*g*_ symmetry because *N*-layer CrI_3_ has centrosymmetric crystal structures. These include every other phonon modes starting from the highest frequency one (*i.e*., *i* = 1, 3, 5, ...), as is the case in Figure 17a that the linearly parallel channel picks  for 1L and 2L and  for 3L and 4L.^219^ In the linearly crossed channel, the measured modes have antisymmetric Raman tensors (*R*_*AS*_) and therefore contains at least one copy of static layered magnetic order contributing to the scattering process (, where  is the *i*^th^ eigenvector and  is the axial vector for the layered AF order in *N*-layer CrI_3_). Because the layered AF order is parity even (odd) for odd (even) *N*, it should pair with the parity even (odd) phonon modes for odd (even) *N* to restore the centrosymmetry of the layered magnetism-phonon coupled entity, leading to the crossed channeling selecting the modes same as (complementary to) those in the parallel channel for odd (even) *N*.

**Figure 18:**
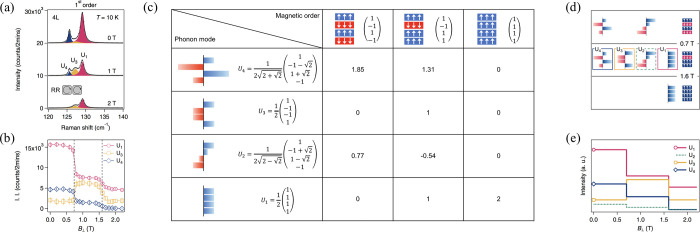
(a) Raman spectra taken on 4L CrI_3_ in the RR channel at 10 K and at 0, 1, and 2 T, corresponding to the three magnetic field ranges *B* < *B*_*c*1_, *B*_*c*1_ < *B* < *B*_*c*2_, and *B* > *B*_*c*2_, respectively. (b) Magnetic field dependence of three modes *U*_1,3,4_. (c) A summary of the analysis of the layered magnetism-assisted Raman scattering for four phonon modes and three layered magnetic structures. (d) List of phonon modes in 4L CrI_3_ that contribute to the layered magnetism-assisted scattering channel with antisymmetric Raman tensors. (e) Calculated magnetic field dependence of the four phonons of 4L CrI_3_, *U*_1–4_, in the RR channel. Adapted with permission under a Creative Commons CC-BY license from ref (219). Copyright 2017 National Academy of Sciences.

Based on this formalism of Davydov splitting and layered magnetism-assisted phonon scattering, the complex magnetic field dependence of thicker CrI_3_ can be explained in a unified way as that of bilayer CrI_3_. For every Davydov split mode , its Raman tensor is composed of the conventional pure structural contribution of the symmetric Raman tensor  and the layered magnetism-coupled phonon contribution of antisymmetric Raman tensor , *i.e.*, , where  is magnetic field independent and is only present for parity-even modes,  which changes across the critical magnetic fields *B*_*c*_, and λ_*i*_ is ratio of the magnetic to structural contribution for the *i*th mode that depends on microscopic parameters such as spin–orbit-coupling. Taking 4L CrI_3_ as an example, it undergoes two magnetic phase transitions at critical magnetic fields of *B*_*c*1_ = 0.7 T and *B*_*c*2_ = 1.6 T, across which *M⃗* changes from (1, −1, 1, −1) to (1, −1, 1, 1) to (1, 1, 1, 1). Figure 18a shows selected Raman spectra taken on 4L CrI_3_ at 0 T (*B* < *B*_*c*1_), 1 T (*B*_*c*1_ < *B* < *B*_*c*2_), and 2 T (*B* > *B*_*c*2_) at 10 K in the circularly parallel RR channel. Three (*U*_1_, *U*_3_, and *U*_4_) out of four modes are resolved, as *U*_2_ is spectrally very close to *U*_1_ and gets overwhelmed by the much stronger *U*_1_. The Raman spectra in three magnetic field ranges show clearly distinct spectral shape that results from changes in the relative spectral intensity of the three modes. Careful magnetic field dependent mode intensities are shown in Figure 18b for the three observed modes, well capturing the critical fields of *B*_*c*1_ and *B*_*c*2_ and displaying different field dependencies. Looking at  for different modes in different magnetic field region (*i.e.*, different *M⃗*) in Figure 18c, one can see that  is nonzero for *U*_2_ and *U*_4_ at *B* < *B*_*c*1_, for all four modes at *B*_*c*1_ < *B* < *B*_*c*2_, and for only *U*_1_ at *B* > *B*_*c*2_ as summarized in Figure 18d. By just tuning the ratio λ_*i*_ and the overall intensity *a*_*i*_, the computed magnetic field dependence of *U*_1–4_ in Figure 18e well matches the experimental data.

The detection of this time-reversal symmetry broken layered magnetism-coupled phonon in few-layer CrI_3_ is a kind of phonon excitation found in such layered magnets. It distinguishes from the Davydov split in few-layer TMD semiconductors by having the magnetic contribution and also differentiates from the AF order-induced zone-folded time-reversal symmetric phonons in (Ni, Fe)PS_3_ by breaking the time-reversal symmetry.

#### Polaronic Effect of Excitons in CrI_3_

The PL spectra of monolayer CrI_3_ shows a large Stokes shift, broad line width, and skewed line shape, ascribed to the strong electron–phonon (e-ph) coupling present in 2D CrI_3_.^215^ Such strong e-ph coupling is further observed in wide-range (70–1100 cm^–1^) Raman spectra taken on 2L, as well as few-layer, CrI_3_, as a series of periodic broad modes up to the eighth order at low temperature.^220^

**Figure 19:**
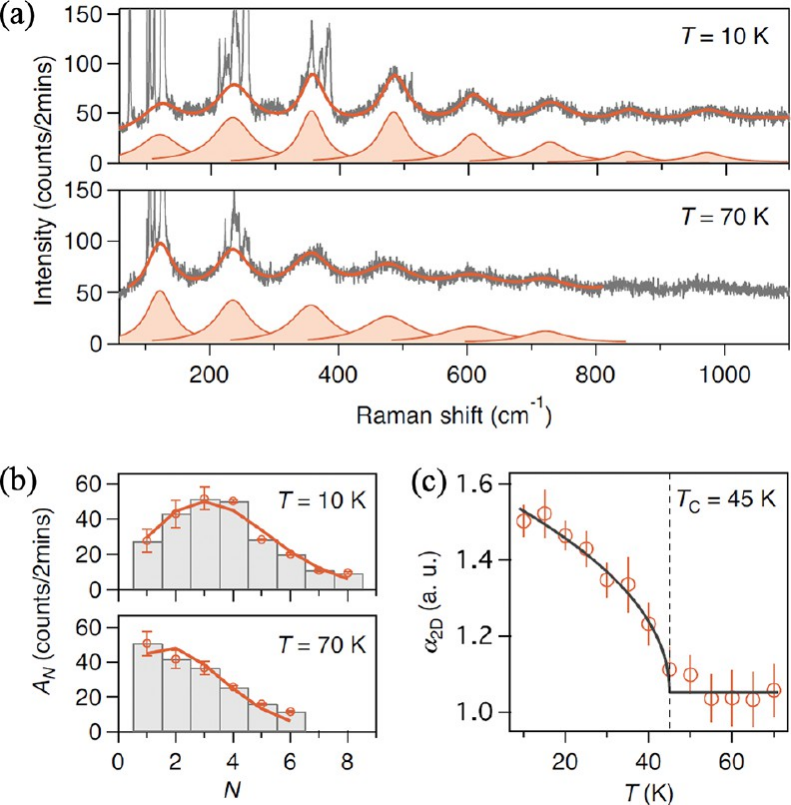
(a) Raman spectra of 2L CrI_3_ taken at 10 and 70 K with an excitation wavelength of 633 nm. Solid orange lines are fits to the raw Raman spectra, using a sum of *N* Lorentzian profiles and a constant background, . (b) Histogram plot of the fitted Lorentzian mode intensity (*A*_*N*_) as a function of *N* at 10 and 70 K. Solid curves are fits of the peak intensity profiles to the Poisson distribution functions, . (c) Plot of 2D e-ph coupling constant (α_2*D*_) as a function of temperature. The dashed vertical line marks the magnetic onset *T*_*C*_ = 45 K. Adapted with permission under a Creative Commons CC BY license from ref (220). Copyright 2020 Springer Nature.

Absorption spectroscopy of 2L CrI_3_ features three prominent broad peaks at 1.51, 1.96, and 2.68 eV,^220^ whose line shape matches well with the reported differential reflectance spectroscopy in monolayer CrI_3_.^215^ These three peaks were originally assigned to be the ligand-field electronic transitions in the differential reflectance study and are revised to be bright exciton states by first principle GW and Bethe–Salpeter calculations.^229^ By choosing the Raman excitation energy matching the charge transfer exciton at 1.96 eV, a clear periodic modulation is observed in the low intensity part of the Raman spectra, which extends up to the eighth order at 10 K and sixth order at 70 K and spans a wide frequency range of 70–1100 cm^–1^ in 2L CrI_3_ (Figure 19a). Please note that the phonons and the layered magnetism coupled phonons reviewed in the two subsections above are all within the frequency range of 70–180 cm^–1^. By fitting the periodic modulation with broad Lorentzian profiles, the characteristic parameters, central frequency ω_*N*_, mode intensity *A*_*N*_, and line width Γ_*N*_, for the *N*th mode can be extracted. A further linear fit of ω_*N*_ as a function of *N* leads to the extracted periodicity of 120.6 ± 0.9 cm^–1^ whose energy matches that of the *E*_*u*_ LO phonon in the phonon calculations.^230^ The broadness of each mode in the periodic modulation precludes the phonon origins and instead suggests the electronic origin, for which the *N*th mode is interpreted as Raman scattering off the exciton states dressed by *N E*_*u*_ LO phonons, with its energy agreeing with *N* times of the LO phonon energy and its line width dominated by the exciton line width. The existence of phonon dressed exciton states is a signature of the polaronic effect of charge transfer excitons in 2L CrI_3_ (as well as few-layer and bulk CrI_3_).

By taking careful temperature-dependent measurements of the polaronic effect, it is notable that the spectral weight shifts toward higher order modes in the periodic modulation. To capture this phenomenon, the fitted mode intensity *A*_*N*_ at every temperature is fitted to a Poisson distribution function , where *A*_0_ is the peak intensity of the original electronic band, *A*_*N*_ is the peak intensity, and α is the e-ph coupling in 3D (*i.e.*, α_3*D*_) that can be scaled by a factor of 3π/4 for 2D (*i.e.*, α_2*D*_)^220^ (Figure 19b). The temperature dependence of α_2*D*_ shows a clear onset at the magnetic onset temperature *T*_*C*_ = 45 K, and α_2*D*_ at 10 K increases by nearly 50% from that above *T*_*C*_ (Figure 19c). The significant enhancement of the e-ph coupling in the magnetic state suggests an intimate coupling among charge, lattice, and spin degrees of freedom.

**Figure 20:**
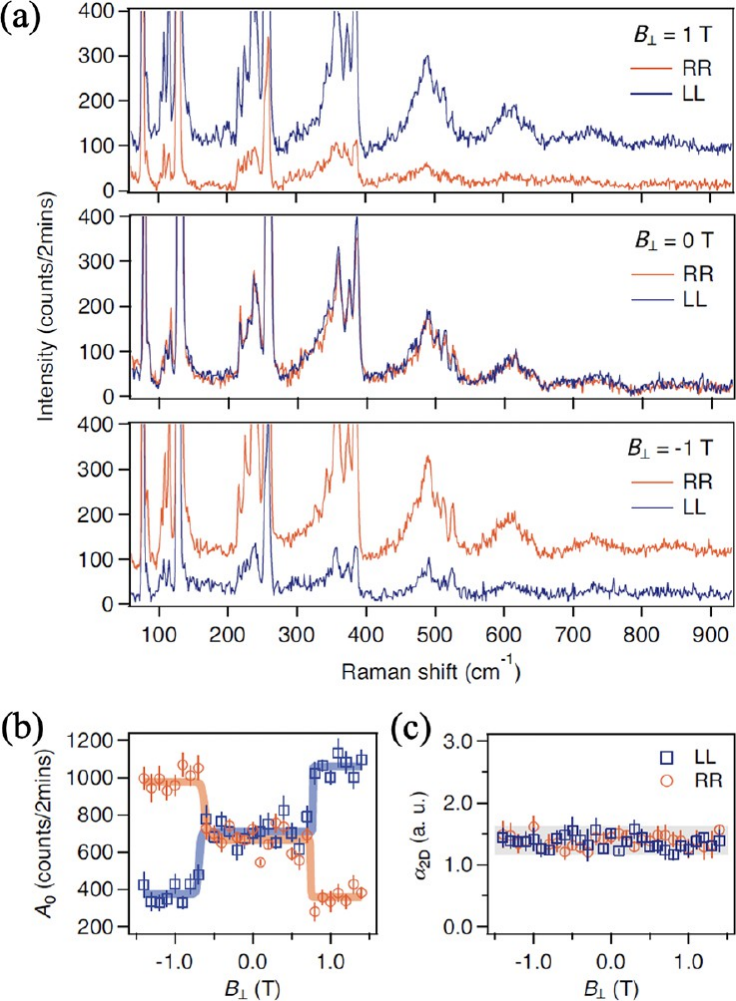
(a) Raman spectra of 2L CrI_3_ acquired at 10 K in the RR and LL polarization channels with an applied out-of-plane magnetic field (*B*_⊥_) of 1 T (top), 0 T (middle), and −1 T (bottom), respectively. (b, c) Plots of the Poisson fit amplitude *A*_0_ (b) and the 2D e-ph coupling strength α_2*D*_ (c) as a function of the applied *B*_⊥_ in the RR (orange data points) and LL (blue) channels. Solid lines are step (orange and blue in b) and linear (gray in c) function fits to the magnetic field dependence of *A*_0_ and α_2*D*_, respectively. Adapted with permission under a Creative Commons CC BY license from ref (220). Copyright 2020 Springer Nature.

From the magnetic-field-dependent Raman measurements of the polaronic effect, one can see a spectral degeneracy in the two circularly parallel polarized channels (RR and LL) at 0 T, but a dramatic and opposite dichroism between RR and LL spectra at 1 T and −1 T (Figure 20a). By measuring the Raman spectra at fine steps of the magnetic field and fitting them with the Lorentzian profiles and then the Poisson distribution functions, the overall spectral intensity *A*_0_(*B*) in the RR and LL channels remains the same below *B*_*c*_ = ± 0.7 T and changes abruptly to different values above *B*_*c*_ with opposite relative strength under opposite magnetic field directions (Figure 20b). The magnetic field dependence of *A*_0_ well captures the layered AF to FM transition at *B*_*c*_, and the dichroic behavior between RR and LL above *B*_*c*_ is consistent with the net magnetization present in the FM state. The e-ph coupling α_2*D*_ however shows no observable magnetic field dependence (Figure 20c), suggesting that the interlayer magnetic order barely affects the e-ph coupling strength that contains the intralayer phonons and excitons.

Although electronic Raman scattering often has a much weaker scattering cross section, it provides a perspective to see the electronic dynamics and its coupling to other collective excitations within the system. The polaronic effect in CrI_3_ reviewed here is the beginning of using electronic Raman scattering to study 2D materials.

### Magnetic Birefringence and Photoluminescence Spectroscopy of 2D Magnetism

Magneto-optical characterization of atomically thin magnetic layers has proven to be a versatile tool for inspecting the magnetic interactions between localized magnetic moments in a crystal lattice. In a general view, nonzero net magnetization gives rise to birefringence effects when the magnetization impacts the polarization state of photons during reflection and/or transmission events. In a commonly used backscattering geometry, one can realize MOKE^231^ or refractive magnetic circular dichroism (RMCD) experiments to get insight into the arrangement of magnetic moments in single-layer (1L) or multilayer films. The MOKE technique relies on the observation that upon reflection from a layer, characterized by a finite net magnetization, additional phase arises between circularly polarized photons with left and right helicity, resulting in the rotation of a linear polarization axis between the incident and reflected photons. The RMCD technique is based on a difference in the absorption coefficient for circularly polarized photons with left and right helicity for a layer with a finite net magnetization, which creates nonzero circular polarization degree within reflected light beam.

**Figure 21:**
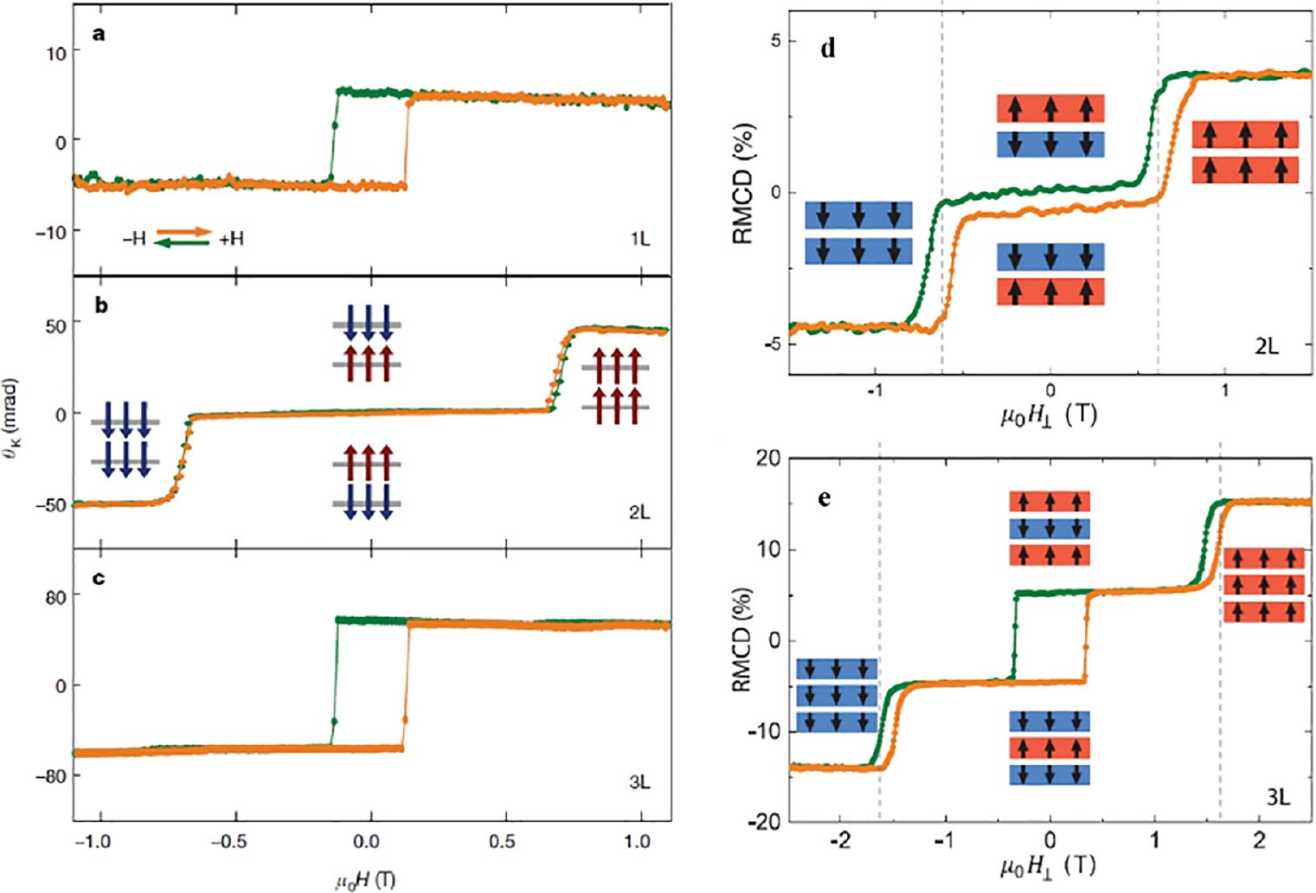
Birefringence effects in atomically thin CrI_3_. (a) The measurement of Kerr rotation angle in external magnetic field reveals finite magnetization in CrI_3_ monolayers.^5^ The inspection of CrI_3_ multilayers demonstrates AF interlayer coupling in (b) bilayers and (c) trilayers.^5^ Panels (a–c) adapted with permission from ref (5). Copyright 2017 by Springer Nature. (d, e) Qualitatively, the same behavior for atomically thin layers is observed when inspecting MCD.^13^ Adapted with permission from ref (13). Copyright 2018 AAAS.

Both types of experiments can demonstrate the existence of net magnetization in monolayer and multilayer films as illustrated in Figure 21 for CrI_3_ crystals. The evolution of the birefringence effects (Kerr rotation angle in MOKE experiments^5^ or circular polarization degree in RMCD experiments)^77^ with an external magnetic field applied perpendicularly to the plane of the magnetic layers, uncovers hysteric behavior characteristic of FM interactions for films with an odd number of layers (1L and 3L presented here). For bilayers and trilayers, an AF interlayer coupling is observed until a sufficient external magnetic field is applied to flip the orientation of magnetization in individual layer(s). A general view of magnetic interactions in thin films of CrI_3_, which arises from birefringence effects, is that individual atomic layers host magnetic moments that are coupled via in-plane FM interaction and that the character of the interlayer coupling in *N*-layers (*N* ≥ 2) depends on *N*. The type of interlayer coupling is likely determined by the different stacking displayed by thin and thick CrI_3_ films.^232^

Notably, the quantitative analysis of the relation between the birefringent effects and magnetization is more challenging in 2D magnets than in their 3D counterparts. For example, upon a single reflection from a surface of a 3D magnet, the Kerr rotation angle is proportional to the net magnetization (θ_*K*_ ∝ *M*) as a result of Maxwell’s equations. In case of 2D films, multiple reflections from individual layers combined with potential limitations to the applicability of Maxwell’s equations in atomic scale may lead to much more complex θ_*K*_(*M*) functions.

**Figure 22:**
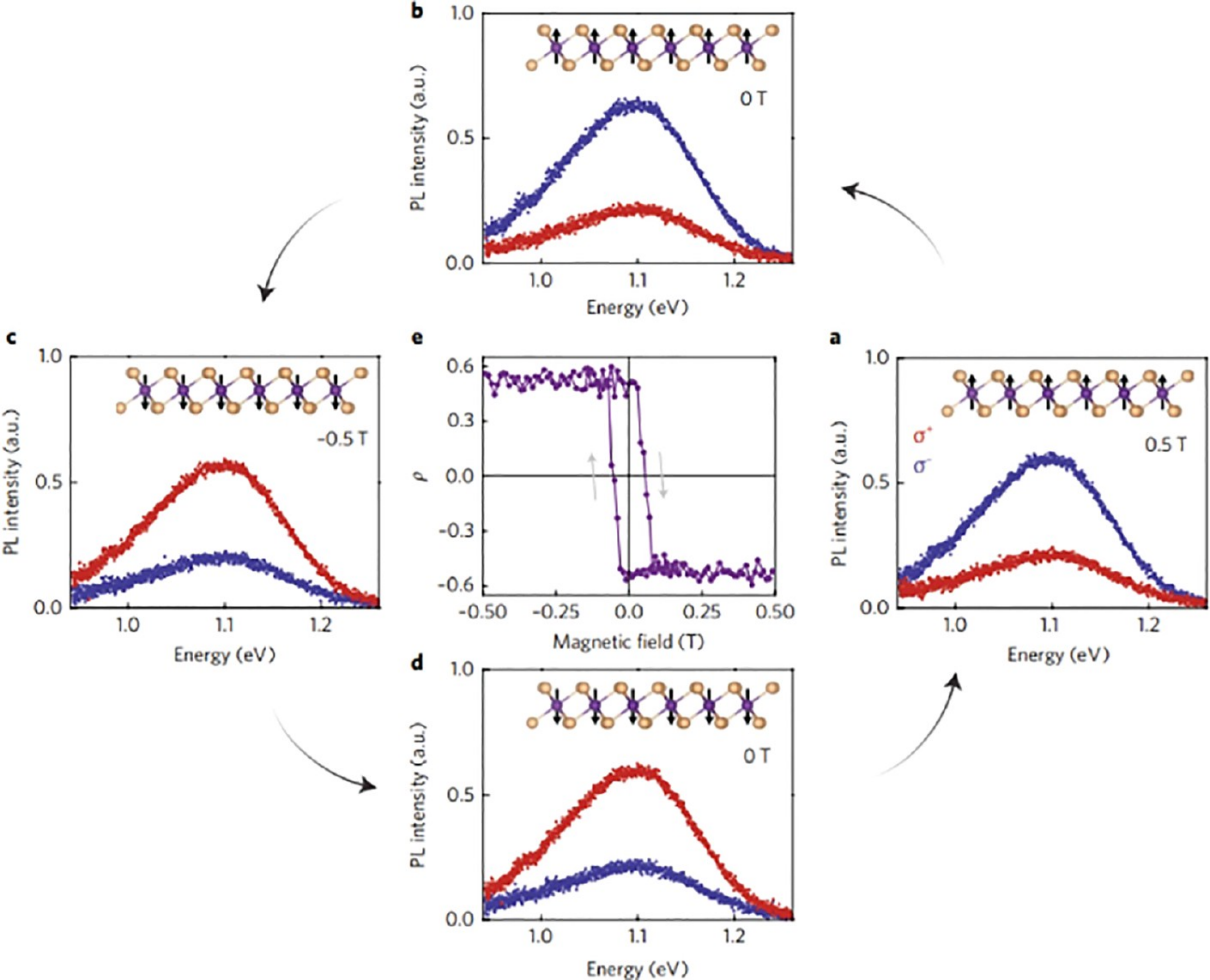
Photoluminescence bands in CrI_3_ monolayers below Curie temperature display large degree of circular polarization which can be linked to the emergence of magnetization. A rapid reorientation of magnetic moments leads to the change in the sign of the polarization degree combined with a hysteric behavior with a coercive field of about 0.1 T, in agreement with analogous observation by birefringence effects. Adapted with permission from ref (215). Copyright 2017 Springer Nature.

An alternative probe into the magnetic effects related to the macroscopic alignment of magnetic moments comes from the inspection of magneto-PL.^215^ CrI_3_ crystals are formed *via* ionic bonds which lead to a band structure characterized by flat bands composed predominantly of Cr^3+^ ions’ d-orbitals.^227,233−239^ Consequently, the photoexcited electron–hole pairs are strongly localized in a way that the Bohr radius becomes comparable with the size of a unit cell in the crystal lattice. The PL response of CrI_3_ appears therefore, characteristically for molecular-like excitation, in form of broad emission bands originating from the coupling between the electronic states with vibrational motion of the crystal lattice. The observation of the circular polarization degree of the emitted light in external magnetic fields is consistent with the birefringence effects, as illustrated in Figure 22 for monolayer CrI_3_. Such finding is indicative of an efficient coupling between the band carriers with the localized magnetic moments of the Cr^3+^ ions.

### Challenges and Perspectives

Magneto-optical spectroscopy techniques have made significant contributions in revealing and investigating emergent 2D magnetic phases in both strongly correlated TMD heterostructures and natural 2D magnetic atomic crystals.

One the one hand, while TMD heterostructures have proven a rich test bed to optically explore emergent correlated phenomena in 2D systems, so far the material choices have been quite limited. This platform is ripe for many discoveries of unexplored phase diagrams of interacting electrons in tailored lattices. The robust excitons in TMDs present a large sensitivity to their surrounding magnetic and dielectric environment, which, together with their spin-selective coupling to photons, can help to probe and sense a variety of emergent magnetic phases either within the TMD themselves or in other nearby 2D vdW materials *via* optical spectroscopy. The ease of the “pick and place” stacking technique characteristic of 2D crystals offers further goals to engineer vdW heterostructures with enhanced sensing capabilities. For example, although vdW heterostructures consisting of a monolayer TMD semiconductor and a bilayer/trilayer ferromagnet have allowed to monitor the flip in the magnetization of an individual layer in the vdW ferromagnet, these heterostructures have not yet allowed to disentangle the layer responsible for the spin flip. Alternatively, a vdW heterostructure in which the bilayer/trilayer ferromagnet is encapsulated by TMDs monolayers with different energy band gaps would in principle allow to track down the individual layers responsible for the magnetization flips, providing further insight into the layered ferromagnetism of vdW crystals. Moreover, two of the current limitations of 2D TMD excitons as sensors of magnetic phenomena are their response to applied magnetic fields (*i.e.*, the exciton *g*-factors) and the energy line widths of the exciton resonances. TMD excitons present typical *g*-factors of |*g*|∼ 4^168^ and transform-limited line widths down to 2–4 meV in the highest quality samples.^240^ Other excitonic species in TMDs which also exhibit spin-dependent optical selection rules, but present larger *g*-factors and narrower optical line widths, might naturally provide better sensitivity. For example, interlayer excitons localized in the moiré potential created by a TMD heterobilayer feature spin- and valley-dependent optical transitions with line widths below 100 μeV and *g*-factors as large as |*g*|∼ 16.^207,241−245^ These localized interlayer excitons and trions might prove useful to keep exploring the emerging field of magnetic phenomena in 2D vdW crystals.

On the other hand, 2D magnetic atomic crystals have shown a wealth of intriguing phenomena that stem out of the intimate interactions among the lattice, spin, and charge degrees of freedom, yet much effort so far has been focused on classical spin systems. Even though examples surveyed in this section focus on the archetype 2D magnet CrI_3_, it already presents interesting phenomena including magnetism-induced structural phase transitions and vice versa (spin–lattice coupling), time-reversal symmetry broken static magnetism-coupled phonon modes (spin-phonon coupling), and magnetism-enhanced exciton–polarons (spin-charge-lattice coupling). Looking at the much broader class of 2D magnets that have been and are to be discovered, ranging from Ising, XY, Heisenberg, and Kitaev-types to spin liquids, one can exploit 2D magnets of different kinds to explore interactions among multiple degrees of freedoms in the 2D limit that cannot be accessed previously in quasi-2D or 3D magnetic systems. One would also be driven to quest how the enhanced thermal and quantum fluctuations and the promoted instabilities, inherent to 2D, would impact the magnetic ground states, spin wave excitations, and the coupling of spin to other degrees of freedoms. Moreover, one would indeed be curious about how moiré superlattices would modulate the 2D magnetism in twisted 2D magnetic hetero- and homostructures. At the same time, the rich physics in 2D magnets naturally calls for sophisticated experimental tools to reveal, understand, and control them. Referring back to the research of quasi-2D and 3D magnets, a vast variety of optical, X-ray, neutron, and transport techniques have been exploited to uncover the nature of their magnetism. Due to the limitation from the sample size of 2D magnets, X-ray, neutron, and bulk transport probes become no longer feasible here, whereas the optical spectroscopy and nanotransport techniques remain active in the field. One may go well beyond the linear magneto-optical spectroscopy discussed here to apply nonlinear optical techniques such as multidimensional coherent spectroscopy that are suitable in capturing the spin, charge, and lattice degrees of freedom and disentangling their coupling in 2D magnets.

## Magnetic Imaging

Magnetic imaging techniques play an important role in studying vdW magnetic materials. With optical, electron beam, and nanoscale physical probes, these techniques offer spatially resolved information on sample magnetization. The microscopic magnetic properties can be unambiguously determined from these measurements upon systematically varying conditions such as external magnetic field or temperature. In this section, we review magnetic imaging techniques that have been used to explore the vdW magnetic materials in the past few years. We briefly introduce the basic physics and applications of these techniques and discuss their strengths and limitations. Indeed, a comprehensive study of vdW magnets requires quantitative measurement at nanoscale and even atomic scale for both static and dynamic magnetization (up to THz). However, no single magnetic imaging technique can satisfy all these requirements. Multiple techniques are needed to obtain a complete understanding of materials.

### Optical Detection Methods

Optical detection techniques using visible and UV light are most widely used in the primary characterization of vdW magnetic materials because they are easy to implement in ordinary laboratories and have nearly no limitation on work temperature and locally applied magnetic field. These methods are based on magnetization dependent response to light, for example, MOKE and MCD. MOKE is one of the magneto-optics effects, which is based on the effect that the polarization of reflected light changes as a function of sample magnetization. The case of transmitted light is called the Faraday effect, which is seldom used because it requires a transparent sample/substrate. MCD refers to the difference in absorption of left and right circularly polarized light by a material with a magnetization direction parallel to the light propagation direction. Actually, both MOKE and MCD originate from spin-dependent electronic states of the magnetic material, which results in an anisotropic optical permittivity. Both MOKE and MCD can be phenomenologically understood as a manifestation of imaginary and real parts of magnetization-dependent optical permittivity tensor.

**Figure 23:**
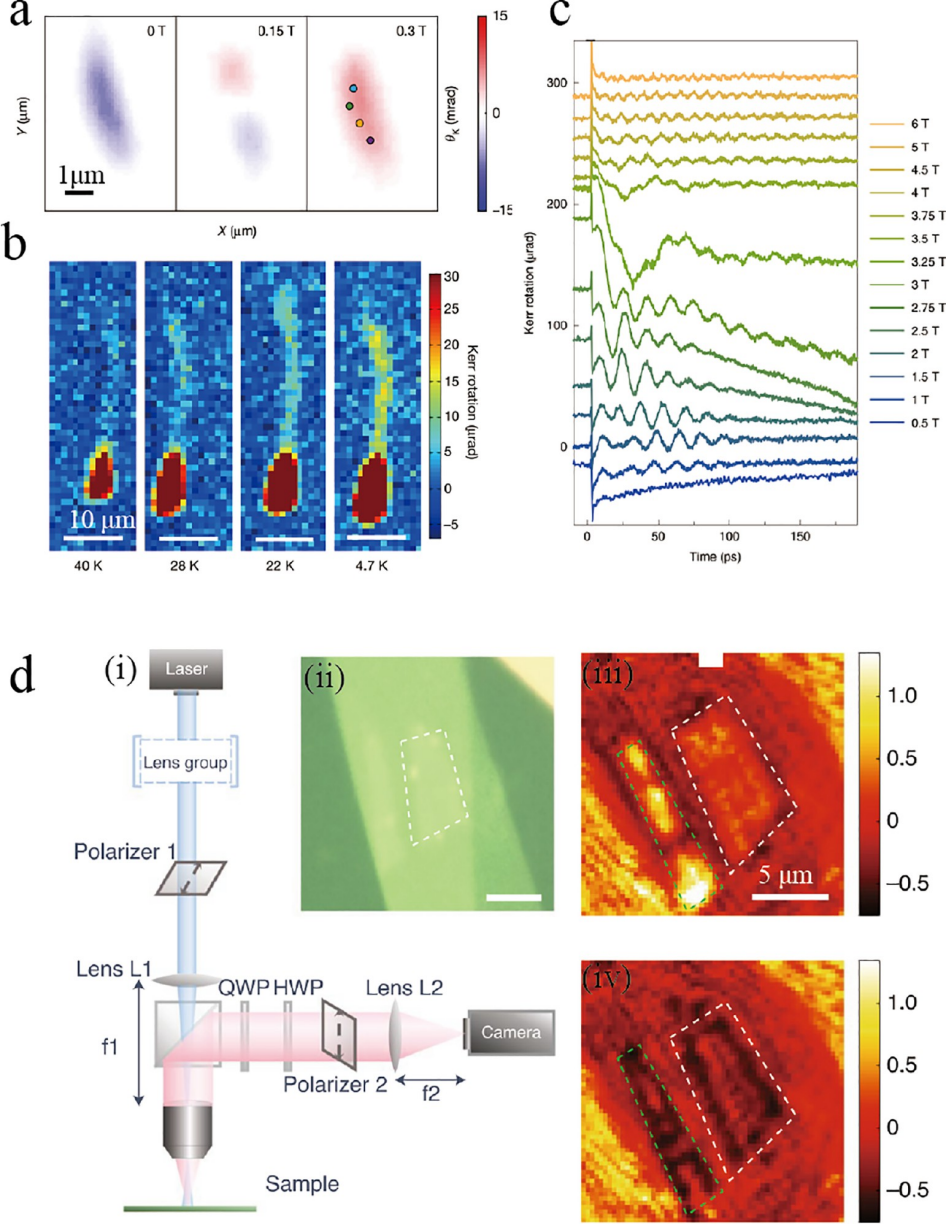
(a) MOKE maps of a CrI_3_ monolayer at external magnetic fields of 0, 0.15, and 0.3 T. Adapted with permission from ref (5). Copyright 2017 Springer Nature. (b) Kerr rotation signal for Cr_2_Ge_2_Te_6_ bilayer flake under 0.075 T as the temperature decreases from 40 to 4.7 K. The average background signal has been subtracted and the signals are truncated at 30 μrad. Adapted with permission from ref (6). Copyright 2017 Springer Nature. (c) Pump-induced Kerr rotation as a function of pump–probe delay time in bilayer CrI_3_ under different in-plane magnetic fields. Adapted with permission from ref (28). Copyright 2020 Springer Nature. (d) (iii) Illustration of the wide-field MCD experimental setup. Blue and red beams represent illumination light from the laser and scattered light from the sample with different effective numeric apertures. HWP: Half-wave plate. QWP: Quarter-wave plate. (ii–iv) Optical microscopy image (ii) and polarization-enhanced MCD image (iii, iv) of a monolayer CrBr_3_ (white dashed box). The MCD image shows giant optical contrast of ±60% for the positive (iii) and negative (iv) remnant magnetization. Adapted with permission from ref (246). Copyright 2020 Springer Nature.

With laser scanning MOKE, Huang *et al*. measured the magnetization of CrI_3_ and observed FM order in monolayer CrI_3_ and AF order in bilayer,^5^ and Gong *et al*. showed that Cr_2_Ge_2_Te_6_ remains a ferromagnet down to bilayers (Figure 23).^6^ The Curie temperatures of monolayer CrI_3_ (45 K) and Cr_2_Ge_2_Te_6_ (30 K) are lower than that of their bulk material, which are 60 and 70 K, respectively, indicating an enhanced spin fluctuation in intrinsic 2D systems. Later, electrical control of the magnetic order in atomically thin CrI_3_ has been demonstrated using MCD.^8,9,11^ A certain challenge for both techniques is that the MOKE and MCD signal level from vdW magnets only results in a relative change of the total detection signal in the order of 10^–3^ to 10^–6^. SiO_2_/Si substrates also contribute a disturbing background. The signal-to-noise ratio can be improved by using lock-in detection in laser scanning imaging setups.^5,8,9,11^ The laser scanning setup can be easily adapted to a femtosecond pump–probe scheme. This enables the observation of high-frequency magnetization dynamics, such as spin waves.^28^ The recent development of single photon level sensitivity electron-multiplying CCD cameras allows to reduce to laser power in the wide-field illumination MCD so that laser heating effects can be suppressed. Jin *et al*. imaged spin fluctuation in monolayer CrBr_3_ using a a wide-field MCD with time resolution of about 10 ms.^246^ They observed the usual strong critical slow down of spin fluctuation and demonstrated magnetic states switching using electrostatic gating .

Besides MOKE and MCD, SHG, Raman spectroscopy, and polarization resolved PL have also been used to probe the magnetic orders of chromium trihalides.^24,203,247^ All these techniques have been proved to be powerful tools to probe vdW magnets down to the monolayer with diffraction-limited spatial resolution.

**Figure 24:**
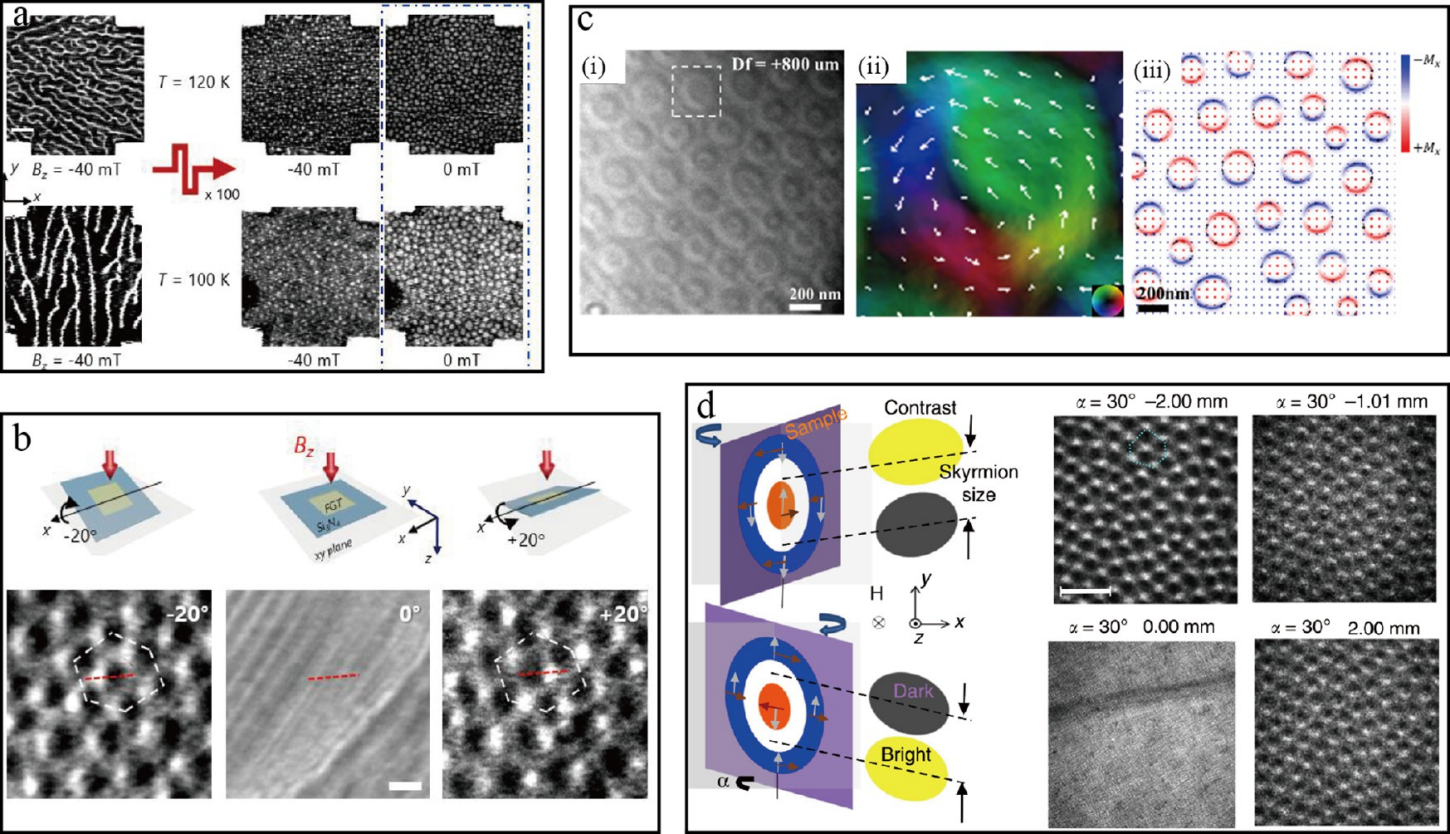
(a) STXM images show the initial labyrinth domain states stabilized at 120 and 100 K transformed to the magnetic skyrmion lattices induced by bipolar pulse bursts. The other two images at *B*_*z*_ = 0 mT were acquired after removing the external fields. (b) Lorentz TEM images of magnetic skyrmion lattices taken at the sample tilting angle of −20° (left), 0° (middle) and 20° (right) with respect to *x*-axis as illustrated in the upper panels at *B*_*z*_ = −40 mT and 160 K, respectively. Panels (a) and (b) are adapted with permission from ref (26). Copyright 2021 American Physical Society. (c) (i) Overfocused Lorentz-TEM images of the skyrmion bubbles taken at 93 K and in zero-field. (ii) An enlarged in-plane magnetization distribution map obtained by transport of intensity equation analysis for a selected skyrmion bubble indicated by the white dotted box in (i). (iii) Simulation of skyrmion lattices at an out-of-plane field of 60 mT. Adapted with permission from ref (25). Copyright 2020 American Chemical Society. (d) Schematic diagram of a Néel-type skyrmion on a tilt sample for Lorentz TEM imaging (left). The orange and blue circles are for positive and negative magnetizations along *z* direction, respectively. Brown arrows indicate the in-plane magnetization component, while gray arrows indicate the Lorentz force. Lorentz TEM images (right) observation of skyrmion lattice from under focus to over focus on WTe_2_/40L Fe_3_GeTe_2_ samples at 180 K with a field of 51 mT. Adapted with permission under a Creative Commons CC BY license from ref (27). Copyright 2020 Springer Nature.

A much higher spatial resolution can be achieved by using XMCD, which is based on measuring the asymmetry in X-ray absorption spectrum (XAS) upon exciting the atomic core levels with the left and right circular polarized X-rays. XMCD can be realized with many detection methods, for example, photoemission electron microscopy (PEEM) and scanning transmission X-ray microscopy (STXM) (Figure 24). Li *et al*. imaged the magnetic structure in Fe_3_GeTe_2_ flakes with PEEM and observed unconventional out-of-plane strip domains down to 14 nm-thick flakes. They found that patterned flakes show a transition to an in-plane vortex phase above the Curie temperature of the patterned flakes (230 K), which persists even at room temperature. Park *et al*. used STXM to observe the formation of Néel-type chiral magnetic skyrmions in thick Fe_3_GeTe_2_ flakes.^26^ They demonstrated that bipolar pulse injection can induce a transition from labyrinth random domain states into Néel-type magnetic skyrmion lattices. Further on, they imaged the current-driven motion of skyrmions. We note that the applications of magneto-optical sum rules allows for the separation and the determination of both spin and orbital magnetic moments of each constituent element in multicomponent systems . The limitation of XMCD is that it requires X-ray synchrotron radiation as a light source and samples should be prepared on special thin substrates (e.g.,100 nm-thick Si_3_N_4_ membrane substrate in ref (26)).

### Electron Microscopy

Electron microscopy has high spatial resolution because the wavelength of an electron (0.037 Å for 100 keV,0.009 Å for 1 MeV) can be much smaller than atomic distances. Scanning electron microscopy (SEM) with polarization analysis can image the magnetic microstructure at nanometer scale by using the spin polarized secondary electrons emitted from the surface of a magnetic material when it is hit by a primary electron beam. Meijer *et al*. used this technique to observe Néel spin spirals at the surface of bulk Fe_3_GeTe_2_ and determined its periodicity to be 300 nm.^248^ The disadvantage of this technique is that it can only be applied to conducting magnets with clean surfaces and requires ultrahigh vacuum conditions to avoid degradation of electron polarization. The integration time of this technique is relatively long due to the low intensity and low efficiency polarization detectors.

LTEM generates magnetic images using the deflection of high-energy (100–1000 keV) electrons transmitted through the sample caused by the Lorentz force on its trajectory. The electron deflection forms an image contrast under certain experimental configurations, such as the conventional Fresnel and Foucault mode, electron holography, and the differential phase contrast mode. It has been shown that only the magnetization curl component parallel to the electron beam direction contributes to the magnetic contrast. Therefore, Néel-type domain walls or skyrmions can only be visualized by tilting the sample because of the lack of out-of-plane magnetization curl. This also makes Lorentz TEM a powerful tool to distinguish the Néel-type and Bloch-type magnetic textures. With this technique, three groups indecently reported topological magnetic textures observed in thick Fe_3_GeTe_2_ flakes.^25,26,249^ Park *et al*.^26^ determined the chirality of magnetic skyrmions observed via the STXM to be of Néel type and showed its origin to be the Dzyaloshinskii-Moriya interactions (DMI)^250,251^ induced at the oxidized Fe_3_GeTe_2_ interface. Wang *et al*.^249^ also observed Néel-type magnetic skyrmions but argued DMI in the Te/Fe_3_Ge/Te slabs as a source of spin chirality. Ding *et al*. showed the skyrmion bubbles form vortex-like domains with Bloch-type chirality which might be the result of competition between the perpendicular magnetic anisotropy and magnetic dipole–dipole interaction. We note that the mechanism that forms Skyrmions in thick Fe_3_GeTe_2_ flakes is still under debate.^252^ Wu *et al*. found that the DMI and topological spin textures can be induced by spin orbit coupling proximity at the Fe_3_GeTe_2_/WTe_2_ interface by transport experiments. They successfully imaged Néel-type magnetic skyrmions in thick Fe_3_GeTe_2_ and few-layer WTe_2_ heterostructures with Lorentz-TEM.^27^

Lorentz TEM suffers from a number of drawback. The electrons scattered by nuclei and the core electron of the sample generate a disturbing background, limiting the maximum sample thickness to be ∼100 nm. In addition, the ability to probe atomically thin vdW magnets has not been demonstrated yet. It is also challenging to apply high external magnetic fields. Both the stray magnetic field and the magnetic induction of the sample contribute to the electron deflection. In some cases, these contributions may cancel out each other and result in no net deflection. This also makes the interpretation of the magnetic image challenging.

### Scanning Probe Microscopy

**Figure 25:**
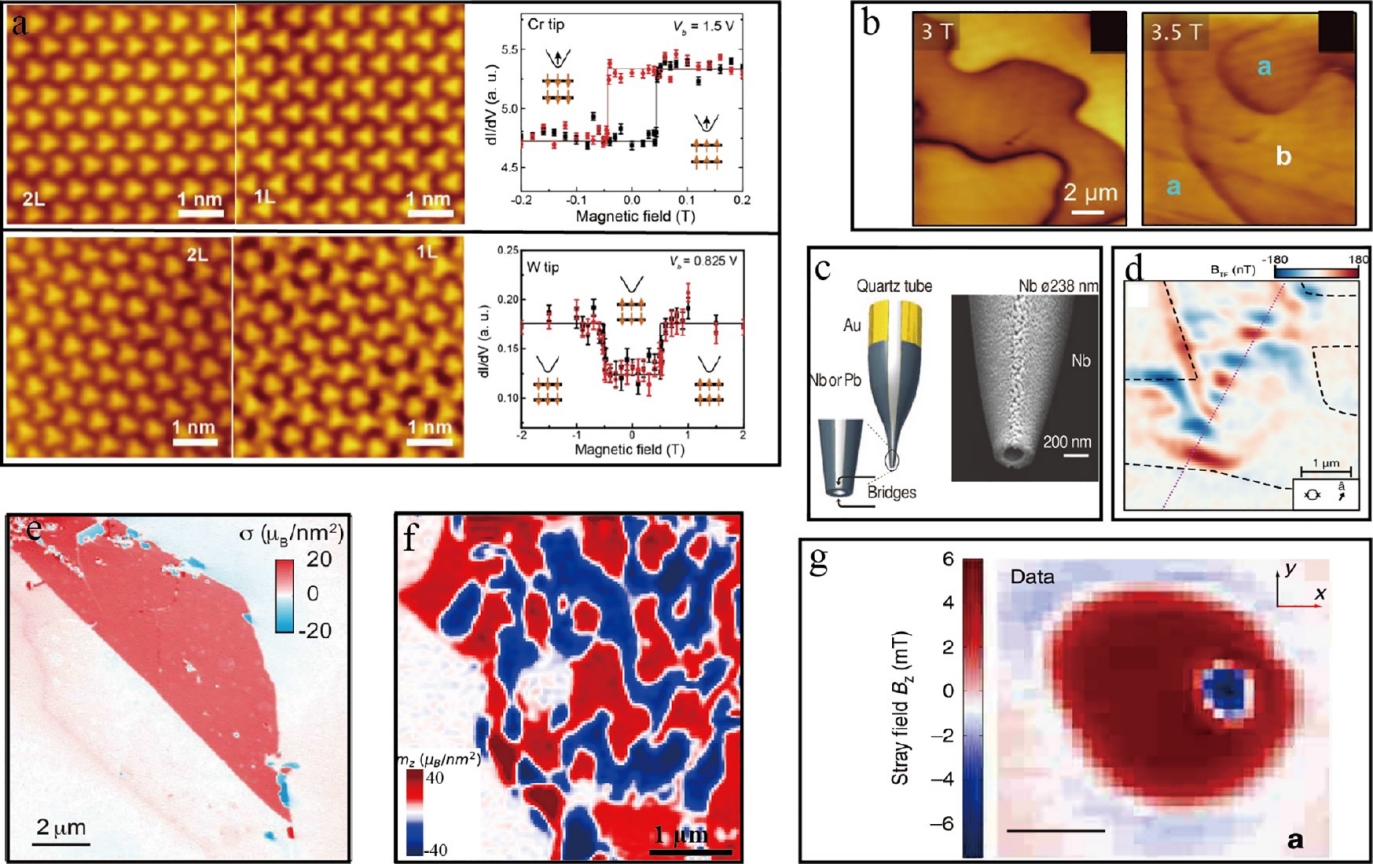
(a) STM images of a CrBr_3_ film with adjacent monolayer (1L) and bilayer (2L) regions and spin-polarized tunneling signals on the bilayer regions as a function of magnetic field. Adapted with permission from ref (20). Copyright 2019 AAAS. (b) Antiferromagnetic domain patterns in MnBi_2_Te_4_ measured with MFM. Adapted with permission from ref (253). Copyright 2020 American Chemical Society. (c) Schematic illustration of the pulled quartz tube with two Nb or Pb superconducting leads connected to Au electrodes and image of a Nb SQUID-on-tip device. Inset: Magnified view showing the superconducting loop on the apex of the tip. The bridges that reside in the gap regions between the leads form the two weak links of the SQUID. Adapted with permission from ref (254). Copyright 2013 Nature Springer. (d) Gradient magnetometry signal associated with the fully polarized twisted bilayer graphene device. Adapted with permission from ref (255). Copyright 2021 AAAS. (e–g) Magnetic images obtained with scanning NV magnetometry. (e) Magnetization image of bilayer/trilayer CrI_3_ adapted with permissions from ref (21). Copyright 2019 AAAS. (f) The magnetic domains in bilayer CrBr_3_ adapted with permission under a Creative Commons CC BY license from ref (256). Copyright 2021 Springer Nature. (g) Stray field map of a magnetic skyrmion in the CoFeB system adapted with permission under a Creative Commons CC BY license from ref (257). Copyright 2018 Springer Nature.

Scanning probe microscopy (SPM) is a family of surface analysis tools that form high-spatial-resolution images by scanning a physical probe over the sample. Spin-polarized scanning tunnelling microscopy (SP-STM) uses a magnetized tip, which results in a spin-dependent tunnelling probability. This technique allows both atomic scale imaging of the crystal structure and *in situ* determination of the magnetic order, which is crucial to investigate the stacking order dependent magnetism in chromium trihalides.^228^ With SP-STM, Chen *et al.* imaged the atomic structure in CrBr_3_ bilayer and found that the two layers can be aligned either parallel or antiparallel, and exhibit AF and FM order, respectively (Figure 25).^20^ In this work, they grow the CrBr_3_ film on freshly cleaved highly oriented pyrolytic graphite substrates by MBE. We note that the SP-STM usually works with a tip-to-sample distance of 1–2 nm or smaller and requires electrically conducting sample/substrate, *i.e.*, it would be a challenge to probe vdW magnetic insulator with SP-STM.

Magnetic force microscopy (MFM) is a variant of atomic force microscopy (AFM) for studying surface magnetic properties. It is based on the noncontact mode AFM while using a magnetized tip. The magnetic contrast is obtained by subtraction of images obtained in a dual-pass scan scheme: The surface topography is recorded by measuring the repulsive forces, and then in the second scan the repulsive forces are recorded with the tip lifted up by a constant distance above the previously measured surface profile. MFM has relative high spatial resolution determined by the tip geometry and lifted height (∼100 nm). Fei *et al*. directly imaged magnetic domains in a thick Fe_3_GeTe_2_ flake with MFM and recorded their evolution upon sweeping the magnetic field.^77^ These results can explain the sudden jump of MOKE signals in hysteresis measurements. Sass *et al*. showed that MFM can be used to directly image antiferromagnetic domain walls in the MnBi_2_Te_4_ family and the Dirac semimetal EuMnBi_2_.^253^ We note that MFM has also been used in other works and successfully revealed the domain structures in thick vdW magnets.^81,258^ However, MFM is an invasive probe because it relies on the interaction between the magnetic tip and the sample. The tip magnetization should be reduced to avoid disturbing the magnetic structure, at the expense of sensitivity. The electrostatic interaction between the tip and sample should also be minimized in magnetic imaging.^253^

In principle, it is possible to obtain quantitative magnetic field information in MFM measurement. But this requires a sophisticated measurement process and system calibration. Scanning probe magnetometry with nanoscale magnetometers is an elegant solution to directly measure the magnetic field above the sample surface. Among all the traditional magnetometers, the superconducting quantum interference device (SQUID) offers the highest sensitivity. The recent progress in nano-SQUID makes it possible to achieve high spatial resolution images in a scanning probe geometry. The state-of-the-art nano-SQUID can achieve device diameters down to about 50 nm, while still offering a magnetic field sensitivity of sub-100 nT/ .^254^ This technique has been used to observe the orbital FM order in twisted bilayer graphene^255^ and equilibrium currents of individual quantum Hall edge states in graphene monolayers.^259^ The scanning nano-SQUID can work at sub-Kelvin temperatures and in high magnetic fields of over 2.5 T. However, it is challenging to use nano-SQUID at high temperature (>10K) limited by the low-temperature superconducting material of the probe.

The nitrogen vacancy (NV) center in diamond is a promising candidate to realized nanoscale magnetometry.^260^ The spin state of NV center can be easily initialized and readout with a green laser and coherently manipulated with microwave fields. Interestingly, the NV spin state has long relaxation and coherence time, and high fidelity single spin control can be realized at temperatures from cryogenic to room temperature. It has been demonstrated as a high sensitivity magnetometer for magnetic field with frequency range from DC to several GHz at a broad temperature range. Devices are operated in a scanning probe geometry using a diamond probe with a single NV center, which can simultaneously obtain the sample surface profile and magnetic field information in a single scan. Thiel *et al*. and Sun *et al.* independently studied few-layer CrI_3_ and CrBr_3_ with cryogenic scanning NV magnetometry.^21,256^ In both works, the stray magnetic field is mapped by measuring the frequency shift of NV electron spin resonance *via* the so-called optically detected magnetic resonance, and the quantitative magnetization images are obtained by reverse-propagation protocols. The spatial resolution achieved in these works is about 50–80 nm, limited by the distance between the NV center and the sample surface. Thiel *et al*. determined the magnetization of CrI_3_ to be about 16 μ_B_/nm^2^ for odd number of layers while vanishing for even number of layers. These results are consistent with layer-number-dependent magnetism reported by Huang *et al*.^5^ Moreover, by studying a sample punctured unintentionally by a diamond tip, it was found that at structural modifications can induce switching between FM and AF interlayer ordering. Latter, Sun *et al*. determined the magnetization of a CrBr_3_ bilayer to be about 26 μ_B_/nm^2^ and imaged the magnetic domain structure formed in a thermally demagnetized sample. They studied the domain evolution upon varying the external magnetic field and directly imaged the domain wall pinning at defect sites. The DC magnetic sensitivity achieved is about 300 nT/ . In the room-temperature measurement, Fabre *et al*. characterized the vdW magnet CrTe_2_ thin flake and determined the magnetization to be ∼25 kA/m for a 20 nm-thick flake.^261^ These works highlighted scanning NV magnetometry as a powerful tool to quantitatively study static properties vdW magnets. A recent work by Vool *et al*.^262,263^ that studies the electron transport in WTe_2_ using echo magnetometry shows that it is feasible to study the dynamic magnetization properties with scanning NV magnetometry.

Although scanning nano-SQUID and scanning NV magnetometry can provide quantitative information on the magnetism, it takes a long time to obtain a whole image. In the cases where the spatial resolution is not that crucial, NV microscopy based on ensemble NV centers implanted in a plane closed to the diamond surface can be used to reduce the measurement time.^264^ A common challenge for magnetic imaging techniques that probe magnetic field is the magnetization reconstruction problem. Generally, the solution of magnetization reconstruction does not result in a single solution. Dovzhenko *et al*. discussed this problem for scanning NV magnetometry and showed that arbitrariness of the solution can be attributed to gauge-like degree of freedom as in electromagnetism.^257^ They determined the magnetization distribution of magnetic skyrmions in a CoFeB system by fixing the gauge. In ref (21) and ref (256), the magnetization reconstruction is based on the out-of-plane spin assumption supported by previous studies. However, solutions for the general case are unexplored.

## Magnetic and Electrical Transport Characterization

The discovery of intrinsic long-range magnetic order in atomic monolayer magnets has triggered significant interest in the fundamental 2D magnetism and spin-related applications. In order to understand the magnetic behavior in 2D thin samples and in nanostructures, it is necessary to probe the nature of magnetism in crystals and its influence on electrical transport properties. Here we survey the current status of magnetocaloric effect in the vicinity of magnetic transition temperature in 2D vdW magnetic crystals. We also present a review of electrical transport properties of the itinerant and exfoliable ferromagnet Fe_*n*_GeTe_2_.

### Magnetocaloric Effect

The magnetocaloric effect discerned *via* adiabatic temperature change or isothermal magnetic entropy change Δ*S*_*M*_ is an important property of ferromagnets that can be used for magnetic refrigeration, which is a promising and environmentally friendly energy conversion technology.^265−268^ The magnetic entropy change Δ*S*_*M*_ is also correlated with the critical behavior of the phase transition *via* magnetization isotherms *M*(*H*) and specific heat *C*_*p*_(*T*). The magnetic entropy change Δ*S*_*M*_(*T*, *H*) induced by the external field is 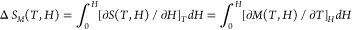
with the Maxwell’s relation [*∂S*(*T*,*H*)/*∂H*]_*T*_ = [*∂M*(*T*,*H*)/*∂T*]_*H*_.^269,270^ In the case of magnetization measured at small discrete magnetic field and temperature intervals, Δ*S*_*M*_(*T*, *H*) could be practically approximated as 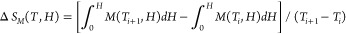. The parameters of |Δ*S*_*M*_(*T*, *H*)| curves follow a series of power laws dependent on the field as , δ_*fwhm*_ ∝ *H*^*b*^, and *RCP* ∝ *H*^*c*^, where  is the maximum of the |Δ*S*_*M*_(*T*, *H*)|, δ_*fwhm*_ is the full width at half-maximum, and  is relative cooling power.^271,272^ The magnetic entropy change parameters directly fit critical exponents; this can avoid the multistep nonlinear fitting induced deviation in the modified Arrott plot and Kouvel–Fisher plot (see Magnetic Critical Behavior section for details). The exponents *n*, *b*, and *c* are *n* = 1 + (β – 1)/(β + γ), *b* = 1/Δ, and *c* = 1 + 1/δ, respectively, where β, γ, δ, and Δ are critical exponents; the reference temperature of the peak entropy change should scale with 1/Δ.^273^*T*_*c*_ can be obtained from the magnetic specific heat change Δ*C*_*p*_(*T*, *H*) = *C*_*p*_(*T*, *H*) – *C*_*p*_(*T*, 0) = *T∂*Δ*S*_*M*_(*T*, *H*)/*∂T*.^274^ With the decrease in temperature, Δ*C*_*p*_ changes from positive in the paramagnetic to negative in FM phase. At the critical point *T*_*c*_, all Δ*C*_*p*_(*T*) curves cross over the zero point. The entropy  and the Δ*S*_*M*_ induced by the external field should be Δ*S*_*M*_(*T*, *H*) = *S*_*M*_(*T*, *H*) – *S*_*M*_(*T*, 0). The adiabatic temperature change Δ*T*_*ad*_ caused by the field change can be indirectly determined, Δ*T*_*ad*_(*T*, *H*) = *T*(*S*, *H*) – *T*(*S*, 0), where *T*(*S*, *H*) and *T*(*S*, 0) are the temperatures in the field *H* ≠ 0 and *H* = 0, respectively, at constant total entropy *S*.

According to the principle of universality, Δ*S*_*M*_(*T*, *H*) can be scaled into a universal curve independent of the external field.^272^ It is constructed by normalizing all the −Δ*S*_*M*_ curves against the respective maximum , namely,  by rescaling the temperature θ defined as θ_–_ = (*T*_*c*_ – *T*)/(*T*_*r*1_ – *T*_*c*_), *T* < *T*_*c*_and θ_+_ = (*T* – *T*_*c*_)/(*T*_*r*2_ – *T*_*c*_), *T* > *T*_*c*_, where *T*_*r*1_ and *T*_*r*2_ are the temperatures of the two reference points below and above *T*_*c*_, respectively. Here, *T*_*r*1_ and *T*_*r*2_ are defined as . A good scaling and convergence of Δ*S*_*M*_(*T*, *H*) curves indicates that the magnetic phase transition is of a second-order type.^275^ Then, the Δ*S*_*M*_(*T*, *H*) curves should follow the scaling equation of state *H*/*M*^δ^ = *f*(ε/*M*^1/β^), where the Δ*S*_*M*_(*T*, *H*) can be rewritten in the form of Δ*S*_*M*_(*T*, *H*) = *H*^(1−α)/Δ^*g*(ε/*H*^1/Δ^), where critical exponents α and Δ can be obtained by Rushbrooke’s law α = 2–2β – γ and Δ = *βδ*.

### Magnetism in Bulk 2D vdW Magnets

Ferromagnetic order in ultrathin crystals was reported in a monolayer CrI_3_ and in a few-layer Cr_2_Ge_2_Te_6_.^5,6^ These discoveries were soon followed by many others including insulating CrCl_3_, CrBr_3_, VI_3_, MnPS_3_, FePS_3_ and conducting monolayers such as Fe_3_GeTe_2_ and CrTe_2_ exhibiting magnetic order close to or at the room temperature.^12,32,119,120,212,234,276−278^ Probing magnetic order in ultrathin crystals is beyond the reach of conventional magnetometry. Thus, several, mostly optical, methods have been used to probe the magnetic state in mono- and few-layer crystals such as MOKE, Raman, reflection MCD, polarization-resolved PL, and NV-center magnetometry (see Magnetic Imaging section). Here we summarize an extensive magnetization measurements used to investigate the critical behavior and magnetocaloric effect in 2D vdW crystals on the example of Cr_2_Ge_2_Te_6_. Estimate of universality class to which the material belongs gives an important starting point for the understanding of magnetic state in bulk and in nanofabricated crystals.

**Figure 26:**
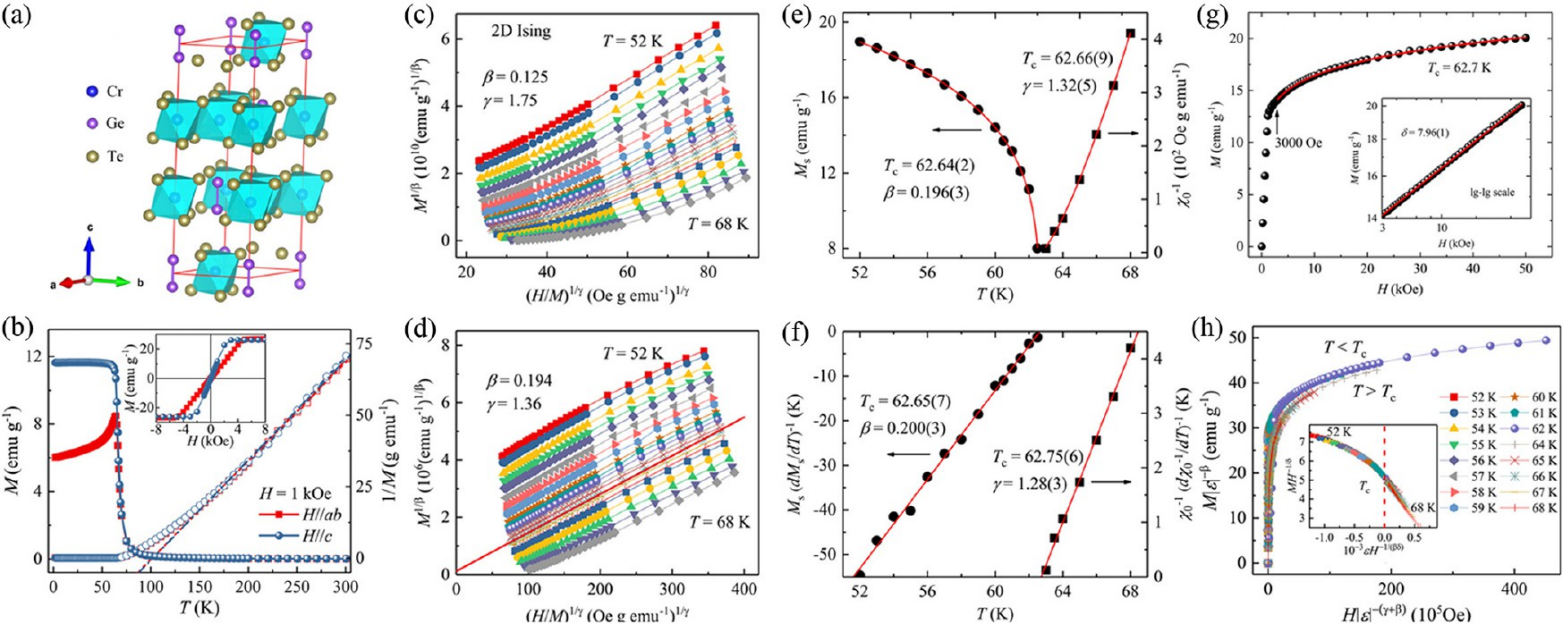
(a) Crystal structure of Cr_2_Ge_2_Te_6_. (b) Temperature dependence of magnetization for Cr_2_Ge_2_Te_6_ measured in *H* = 1 kOe. Inset: Field dependence of magnetization at *T* = 2 K. (c) 2D Ising model plot of isotherms around *T*_*c*_. (d) Modified Arrott plot of *M*^1/β^*versus* (*H*/*M*)^1/γ^ with β = 0.194 and γ = 1.36. The straight line is the linear fit of isotherm at *T* = 62.5 K. (e) Temperature dependence of the spontaneous magnetization *M*_*s*_ (left) and the inverse initial susceptibility  (right) with solid fitting curves. (f) Kouvel–Fisher plots of  (left) and  (right) with solid fitting curves. (g) Isotherm *MversusH* plot collected at *T*_*c*_ = 62.7 K. Inset: The same plot in log–log scale with a solid fitting curve. (h) Scaling plots of renormalized magnetization *mversus* renormalized field *h* below and above *T*_*c*_ for Cr_2_Ge_2_Te_6_. Inset: The rescaling of the *M*(*H*) curves by *MH*^–1/δ^*versus* ε*H*^–1/(*βδ*)^. All panels adapted with permission from ref (279). Copyright 2017 American Physical Society.

Cr_2_Ge_2_Te_6_ crystallizes in a quasi-2D layered structure (Figure 26a), in which the Cr ions are located at the centers of slightly distorted octahedra of Te atoms.^279,280^ When compared to Cr_2_Si_2_Te_6_, Cr_2_Ge_2_Te_6_ features smaller vdW gap, larger cleavage energy and larger in-plane nearest-neighbor Cr–Cr distance, which enhances the *T*_*c*_ from 32 K for Cr_2_Si_2_Te_6_ to 61 K for Cr_2_Ge_2_Te_6_ (Figure 26b). The isothermal data with 2D-Ising model (β = 0.125 and γ = 1.75) (Figure 26c) show a set of relatively parallel straight lines, in contrast to different 3D models (not shown here). Figure 26d shows the final modified Arrott plot generated by using β = 0.194 and γ = 1.36, rapidly converged by a rigorous iterative method and with the adoption of 2D Ising model as an initial model.^269^ The finally obtained *M*_*s*_(*T*) and  are plotted as a function of *T* in Figure 26e. The power law fit gives β = 0.196(3), *T*_*c*_ = 62.64(2) K for *T* < *T*_*c*_ and γ = 1.32(5), *T*_*c*_ = 62.66(9) K for *T* > *T*_*c*_ (Figure 26e), very close to the values β = 0.200(3) with *T*_*c*_ = 62.65(7) K and γ = 1.28(3) with *T*_*c*_ = 62.75(6) K generated from the Kouvel–Fisher plot (Figure 26f). The critical exponent δ = 7.96(1) determined from the critical isotherm analysis at *T*_*c*_ = 62.7 K (Figure 26g), matches reasonably well with the calculated values δ = 7.73(15)/7.40(5) by using the Widom relation δ = 1 + γ/β with β and γ obtained from the modified Arrott/Kouvel–Fisher plot. The scaled *mversush* well collapse into two separate branches, one below *T*_*c*_ and another above *T*_*c*_ (Figure 26h), respectively, as well as the *MH*^–1/δ^*versusεH*^–1/(*βδ*)^ which collapse into a single curve (inset in Figure 26h). This clearly indicates that the interactions get properly renormalized in a critical regime following the equation of state scaling.

**Figure 27:**
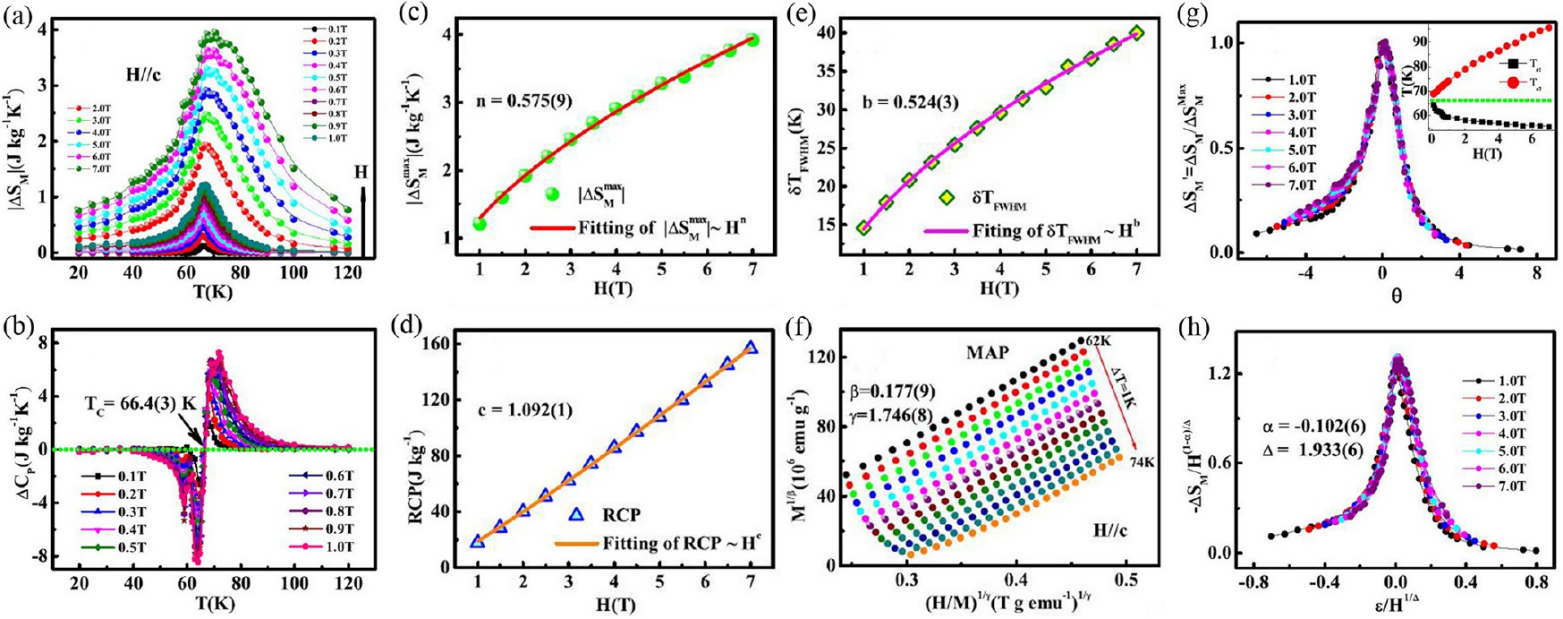
Temperature dependence of (a) |Δ*S*_*M*_| and (b) Δ*C*_*p*_ in different fields with *H* ∥ *c*; Field dependence of parameters from |Δ*S*_*M*_(*T*)| with the fitted curves: (c) , (d) *δT*_*fwhm*_, and (e) *RCPversusH* with the fitted curves; (f) Modified Arrott plot based on the obtained critical exponents. Scaling of the |Δ*S*_*M*_(*T*, *H*)| curves: (g) normalized Δ*S*_*M*_(*T*, *H*) as a function of θ (inset gives *T*_*r*1_ and *T*_*r*2_ as a function of *H*); (h) – Δ*S*_*M*_/*H*^(1−α)/Δ^*versus* ε/*H*^1/Δ^. All panels adapted with permission from ref (280). Copyright 1998 American Physical Society.

Considering the intrinsic correlation between the magnetic entropy change (|Δ*S*_*M*_|) and critical behavior near the magnetic phase transition, the critical exponents β, γ, δ, and *T*_*c*_ can also be deduced from the magnetic-field-dependent |Δ*S*_*M*_(*T*, *H*)| without the use of any initial models. The |Δ*S*_*M*_(*T*, *H*)| curves in different *H* are depicted in Figure 27(a). The precise value of *T*_*c*_ = 66.7 K can be determined by the zero point of magnetic specific heat change Δ*C*_*p*_ (Figure 27b). The field dependence of magnetocaloric effect parameters , δ_*fwhm*_, and *RCP* are plotted in Figure 27c–e, where the power-law fitting gives *n* = 0,575(9), *b* = 0.524(3), and *c* = 1.902(1), respectively. Then the critical exponents can be calculated as β = 0.177(9), γ = 1.746(8), δ = 10.869(5), and Δ = 1.907(3), which avoids the multistep nonlinear fitting described previously. The generated modified Arrott plot illustrates the reliability of the obtained critical exponents, in which the *M*^1/β^*versus* (*H*/*M*)^1/γ^ curves show a series of lines parallel to each other (Figure 27f). Good collapse and overlap of rescaled curves around *T*_*c*_ further confirms the reliability and validity of critical exponents obtained by the magnetic entropy change method (Figure 27g,h). Critical exponents calculated by different methods should agree with each other and show self-consistency for a certain type of phase transition because the critical behavior is independent of the microscopic details.

It is also important to understand the nature as well as the range of interactions in Cr_2_Ge_2_Te_6_. In renormalization group theory analysis the interaction decays with distance *r* as *J*(*r*) ≈ *r*^–(*d*+σ)^, where σ is the range of interaction.^281^ According to this model, the range of spin interaction is long or short depending on the σ < 2 or σ > 2; and the mean-field model is satisfied when σ ≤ 3/2. The susceptibility exponent γ calculated from renormalization group approach is
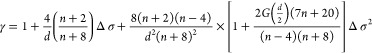
where  and .^282^ To find out the range of interaction σ as well as the dimensionality of both lattice *d* and spin *n* in Cr_2_Ge_2_Te_6_, the parameter σ in above equation is adjusted for different set of {*d*:*n*} so that it yields a value for γ close to that observed experimentally.^282^ Here {*d*:*n*} = {2:1} and σ ≈ 1.5–1.6 implies that the spin interaction in Cr_2_Ge_2_Te_6_ is of a 2D Ising type coupled with a long-range interaction. This long-range interaction might be associated with a non-negligible interlayer and strong spin–lattice coupling.^283−286^

Critical exponents and magnetocaloric effect parameters obtained by different methods for related 2D vdW magnets are summarized in Table 7. In a comprehensive study of critical exponents for 2D magnets Taroni *et al*. concluded that the critical exponent β for a 2D magnet should be within a window ∼0.1 ≤ β ≤ 0.25.^316^ The critical exponents of Cr_2_Si_2_Te_6_ are close to those of Cr_2_Ge_2_Te_6_, indicating 2D Ising-like behavior coupled with a long-range interaction (Table 7). In contrast to Cr_2_(Si/Ge)_2_Te_6_, the critical exponents of bulk CrI_3_, Cr_1–*x*_Te, FeCr_2_Te_4_ and Fe_*n*_GeTe_2_ crystals exhibit 3D critical scaling since those compounds possess smaller vdW gaps, giving rise to much stronger interlayer coupling (Table 7). In CrX_3_ (X = Cl, Br, and I), the Cr–Cr distances increase with increasing halogen size, the direct exchange should weaken from Cl to Br to I. Therefore, the superexchange *via* Cr-X-Cr is expected to be FM and plays a more important role in magnetic interaction.^235^ Moving form Cl to Br to I, more covalent Cr–X bonds strengthen superexchange interactions and raise ordering temperatures. They also increase spin–orbit coupling, which may account for the large magnetic anisotropy.^235^ CrCl_3_ and VI_3_ are situated close to a 2D to 3D critical point (β ∼ 0.25). A similar critical phase transition crossover from 2D to 3D is also found in NiPS_3_ and MnPS_3_.^317,318^ Furthermore, a tunable *T*_*c*_ of 150–220 K for Fe_3−δ_GeTe_2_ strongly depends on the Fe deficiency, suggesting the role of Fe occupancy in the magnetic exchange. However, the obtained critical exponents for this family show a robust 3D Heisenberg-type spins coupled with a long-range interaction; the range of interaction σ ranges from 1.6 to 1.89 with decrease in Fe content (Table 7). It is interesting that the Fe vacancy has small effect on the universality class of the critical behavior. Fe_5_GeTe_2_ also exhibits 3D Heisenberg-type magnetic exchange with long-range interaction decaying as *J*(*r*) ≈ *r*^–4.916^ (Table 7). In Mn_3_Si_2_Te_6_, a 3D analog of Cr_2_Si_2_Te_6_, some Mn atoms reside in the vdW gap, similar to intercalated TMDs.^319,320^ Such structure promises the robust interlayer coupling as well as a 3D long-range magnetic interaction.^315^ Further investigation on the size-dependent properties is of interest. In addition, we can take the dimensional crossover in the critical exponents from the values expected in 3D to those in 2D (see Table 4) to mark the transition from a 3D behavior to a truly 2D character, as for examples for CrI_3_ and Fe_3_GeTe_2_.^321,322^

**Table 7:** Comparison of magnetocaloric effect parameters and critical exponents of indicated vdW magnetic materials^a^

materials	field	ref	technique	–Δ*S*_*M*_^max^	RCP	Δ*T*_*ad*_	α	β	γ	δ	*T*_*c*_	*J*(*r*)
Cr_2_Si_2_Te_6_	*H* ∥ *ab*	(287)	MH	4.9								
	*H* ∥ *c*		MH	5.05	114							
		(288)	MAP					0.170(8)	1.532(1)	10.01(5)^*cal*^	30.95(20)	*r*^–3.630^
			KFP					0.175(9)	1.562(9)	9.93(6)^*cal*^	30.97(16)	
			CI							9.917(8)	31	
		(287)	MAP					0.169(4)	1.33(8)	8.9(3)^*cal*^	32.0(4)	
			KFP					0.178(9)	1.32(4)	8.4(2)^*cal*^	32.2(4)	
			CI							9.28(3)	32	
												
Cr_2_Ge_2_Te_6_	*H* ∥ *ab*	(287)	MH	2.6								
	*H* ∥ *c*		MH	2.64	87							
		(289)	MH	3.21	94.3							
		(279)	MAP					0.196(3)	1.32(5)	7.73(15)^*cal*^	62.65(7)	*r*^–3.52^
			KFP					0.200(3)	1.28(3)	7.40(5)^*cal*^	62.70(12)	
			CI							7.96(1)	62.7	
		(280)	MEC					0.177(9)	1.746(8)	10.869(5)^*cal*^	66.4(3)	*r*^–3.592^
		(290)	MAP					0.242(6)	0.985(3)	5.070(6)^*cal*^	67.93(7)	
			KFP					0.240(6)	1.000(5)	5.167(6)^*cal*^	67.90(8)	
			CI							5.032(5)	67.9	
		(291)	AC					0.35	1.43	5.24	62.84	
												
CrI_3_	*H* ∥ *ab*	(292)	MH	2.68								
	*H* ∥ *c*		MH	4.24	122.6							
			SH	5.65		2.34						
		(293)	MAP					0.284(3)	1.146(11)	5.04(1)^*cal*^	60.42(8)	*r*^–4.69^
			KFP					0.260(4)	1.136(6)	5.37(4)^*cal*^	60.24(26)	
			CI							5.32(2)	60	
		(294)	MAP					0.325(6)	0.825(3)	3.538(6)^*cal*^	64.02(7)	
			KFP					0.323(6)	0.835(5)	3.585(6)^*cal*^	63.99(9)	
			CI							3.569(4)	64	
			SH				0.11					
												
CrBr_3_	*H* ∥ *c*	(295)	MH	7.2	191.5							
			SH	6.91		2.37						
												
CrCl_3_	*H* ∥ *ab*	(296)	MH	14.6	340.3							
	*H* ∥ *c*		MH	13.8	317.3							
	*H* ∥ *ab*	(297)	MH	19.8								
	*H* ∥ *c*		MH	19.5								
			SH			6.8						
	*H* ∥ *ab*	(296)	MAP					0.26(1)	0.86(1)	4.31(9)^*cal*^	19.11(10)	
			KFP					0.28(1)	0.89(1)	4.18(8)^*cal*^	19.18(37)	
			CI							4.6(1)	19	
												
VI_3_	*H* ∥ *ab*	(298)	MH	2.27								
	*H* ∥ *c*		MH	2.64								
			SH	2.80		0.96						
	*H* ∥ *ab*	(299)	MH	2								
	*H* ∥ *c*		MH	3								
		(298)	MAP					0.244(5)	1.03(1)	5.21(4)^*cal*^	50.04(10)	
			CI							5.24(2)	50	
		(299)	MEC					0.204(8)	1.65(7)	9.09(1)^*cal*^		
			MAP					0.155(7)	1.04(2)	7.70(9)^*cal*^	45.31(13)	
			KFP					0.146(1)	1.07(7)	8.32(8)^*cal*^	45.32(12)	
			CI							7.78(8)	45	
			AC					0.12(3)	0.92(4)	8.30(4)		
												
Fe_2.64_GeTe_2_	*H* ∥ *ab*	(300)	MH	1.26								
	*H* ∥ *c*		MH	1.44	113.3							
			SH	1.20		0.66						
		(301)	MAP					0.374(1)	1.273(8)	4.404(12)^*cal*^	151.27(1)	*r*^–4.89^
			KFP					0.372(4)	1.265(15)	4.401(6)^*cal*^	151.25(5)	
			CI							4.50(1)	151	
												
Fe_2.72_GeTe_2_	*H* ∥ *c*	(302)	MEC					0.361(3)	1.736(7)	5.806(8)	157.2(2)	
	*H* ∥ *ab*		MEC					0.714(3)	1.243(7)	2.741(1)	158.5(2)	
												
Fe_2.85_GeTe_2_	*H* ∥ *c*	(303)	MAP					0.361(2)	1.225(2)	4.39(1)^*cal*^	193.68(26)	*r*^–4.8^
			KFP					0.363(4)	1.228(4)	4.38(3)^*cal*^	193.77(11)	
			CI							4.41(3)	194	
												
Fe_3_GeTe_2_	*H* ∥ *c*	(304)	MAP					0.327(3)	1.079(5)	4.30(5)^*cal*^	215.13(8)	*r*^–4.6^
			KFP					0.322(4)	1.063(8)	4.30(7)^*cal*^	215.15(21)	
			CI							4.261(9)	215	
												
Fe_5_GeTe_2_	*H* ∥ *c*	(305)	MAP					0.351(1)	1.413(5)	5.02(6)^*cal*^	273.82(10)	*r*^–4.916^
			KFP					0.346(4)	1.364(9)	4.94(0)^*cal*^	273.86(19)	
			CI							5.02(1)	274	
												
t-Cr_5_Te_8_	*H* ∥ *ab*	(306)	MH	1.39								
	*H* ∥ *c*		MH	1.73	131.2							
		(307)	MAP					0.314(7)	1.83(2)	6.83(7)^*cal*^	230.76(9)	*r*^–4.626^
			KFP					0.315(7)	1.81(2)	6.75(6)^*cal*^	230.65(26)	
			CI							6.35(4)	230	
		(308)	MAP					0.362	1.399	4.86^*cal*^	220.1	*r*^–4.949^
			CI						4.83	220		
												
m-Cr_5_Te_8_	*H* ∥ *c*	(309)	MAP					0.327(4)	1.26(1)	4.9(1)^*cal*^	221.82(44)	*r*^–4.94^
			KFP					0.321(7)	1.27(2)	4.9(2)^*cal*^	221.7(7)	
			CI							4.86(4)	222	
			MH	2.38	143							
												
Cr_4_Te_5_	*H* ∥ *c*	(310)	MH	2.42								
	*H* ∥ *ab*		MH	2.58								
			MAP					0.388(4)	1.290(8)	4.32(3)^*cal*^	318.88(24)	*r*^–4.85^
			KFP					0.387(9)	1.288(5)	4.32(2)^*cal*^	318.74(26)	
			CI							3.93(8)	318.7	
												
FeCr_2_Te_4_	*H* ∥ *c*	(311)	MH	1.92								
			MAP					0.33(2)	1.20(1)	4.6(2)^*cal*^	122.8(4)	*r*^–4.88^
			KFP					0.30(1)	1.22(1)	5.1(1)^*cal*^	122.6(7)	
			CI							4.83(6)	123	
												
Mn_3_Si_2_Te_6_	*H* ∥ *c*	(312)	SH	2.94		1.14						
	*H* ∥ *ab*		MH	2.53								
		(312)	MAP					0.41(1)	1.25(1)	4.05(5)^*cal*^	74.23(4)	
			KFP					0.41(1)	1.21(2)	3.95(2)^*cal*^	74.27(15)	
			CI							4.29(5)	74	
												
Cr_0.33_NbS_2_	*H* ∥ *c*	(313)	MAP					0.370(4)	1.380(2)	4.729(7)^*cal*^	126.3(7)	*r*^–4.9^
			CI							4.853(6)	126	
		(314)	KFP					0.346(40)	1.344(2)	4.88(44)^*cal*^	130.78(8)	
												
Fe_0.26_TaS_2_	*H* ∥ *c*	(315)	MAP					0.460(4)	1.216(11)	3.64(3)^*cal*^	100.67(3)	*r*^–4.71^
			KFP					0.459(6)	1.205(11)	3.63(4)^*cal*^	100.69(5)	
			CI							3.69(1)	100.7	

a The MEC, SH, MAP, KFP, and CI represent the magnetic entropy change, specific heat, modified Arrott plot, Kouvel–Fisher plot, and critical isotherm, respectively.

### Metallic vdW Ferromagnets

Here we discuss metallic, itinerant layered vdW ferromagnets^87,88,323^ which can be exfoliated and incorporated into heterostructures^27,109,115,324,325^ while presenting relatively high Curie temperatures.^87,88,323^ Such systems are also tunable *via* an external electric field^12^ or through microstructuring.^326^

**Figure 28:**
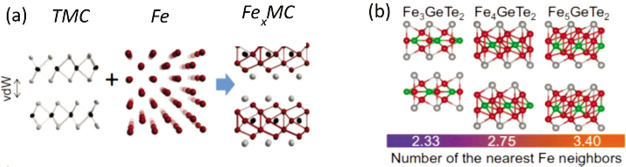
(a) Schematics illustrating the combination of two structural motifs, *i.e.*, that of transition-metal dichalcogenides *TMC*_2_ (TM, transition metal; C, chalcogen) together with that of body-centered cubic iron to form Fe-rich vdW coupled ferromagnets of composition Fe_*x*_GeC_2_. (b) Three stable structures within the Fe_*n*_GeTe_2_ series with the values *n* = 3, 4, and 5 which were previously identified through *ab initio* calculations. Fe–Fe dumbbells are the common structural units that form multiple-layer Fe-rich slabs stacked through vdW-like coupling. As *n* increases, the number of nearest Fe neighbors per Fe atom gradually increases, which is expected to enhance the pair exchange interaction and, thus, *T*_*c*_. All panels are adapted with permission under a Creative Commons CC BY-NC 4.0 license from ref (88). Copyright 2020 AAAS.

So far, the most well studied compound within this series is Fe_3−δ_GeTe_2_ which displays comparatively high *T*_*c*_, relative to the magnetic ordering temperature of other 2D magnetic systems, *i.e.*, ranging from 150 to 220 K depending on the Fe occupancy.^99,323,328,329^ Fe_3_GeTe_2_ can be understood as containing Fe_3_Ge slabs separated by vdW-like bonded Te layers (Figure 28). An alternative way to understand its structure, as discussed in ref.^88^ is to imagine a scaffold with a lattice akin to that of the transition-metal dichalcogenides (*TMC*_2_) but with the Fe atoms stuffed within it. Its structure and the valence states of the constituent atoms can be written as (Te^2–^) ()[() (Ge^4–^)]() (Te^2–^) per formula unit which leads to two inequivalent Fe sites,  and , within the Fe_3_Ge slab.^12,329^ Partially filled Fe-d orbitals dominate the band structure around the Fermi level and give rise to itinerant ferromagnetism in bulk Fe_3_GeTe_2_.^6^ Adjacent monolayers are separated by a 2.95 Å vdW gap in bulk crystal. As a result of the reduced crystal symmetry inherent to the layered structure, bulk Fe_3_GeTe_2_ exhibits a strong magneto-crystalline anisotropy.^330^ Such anisotropy would help to stabilize long-range FM order in monolayer of this compound.

**Figure 29:**
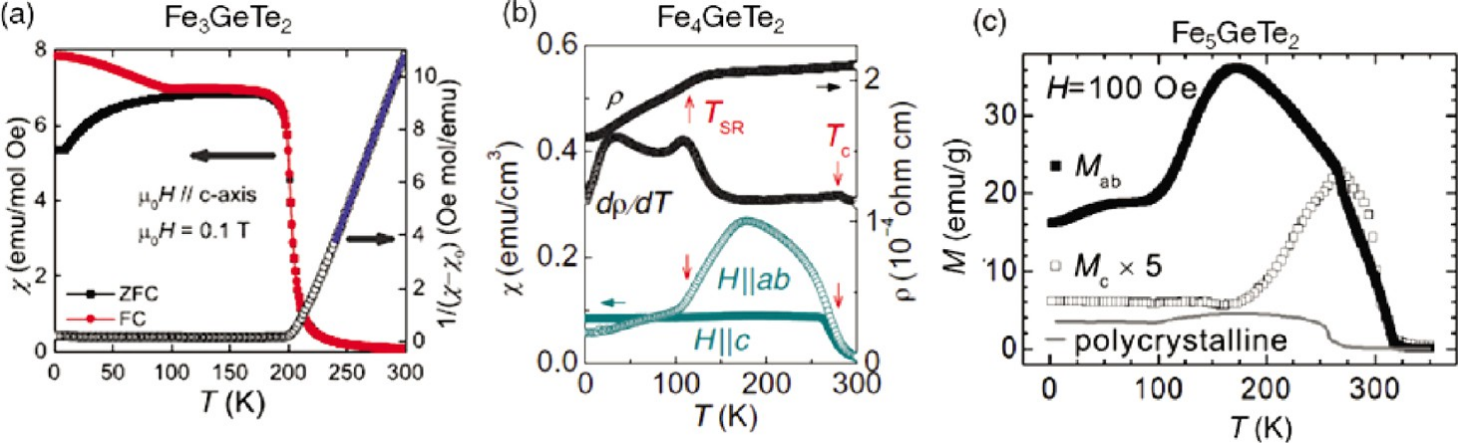
(a) Magnetic susceptibility χ as a function of the temperature *T* for a Fe_3_GeTe_2_ single-crystal measured under zero field-cooled (black markers) and field cooled (red markers) conditions. Open markers correspond to the inverse of χ where the blue line is a linear fit. The Curie temperature exceeds 200 K. Adapted with permission from ref (327). Copyright 2017 American Physical Society. (b) χ as a function of *T* for a Fe_4_GeTe_2_ single-crystal and for fields along the *c*-axis (solid green makers) and the *ab*-plane (open green markers). Solid black markers depict both the resistivity and its derivative indicating a *T*_*c*_ of ∼270 K with another spin-reorientation transition above 100 K. Adapted with permission under a Creative Commons CC BY-NC 4.0 license from ref (88). Copyright 2020 AAAS. (c) Magnetization as a function of the temperature for Fe_5_GeTe_2_ for fields along the *c*-axis (open markers) and the *ab*-plane (solid markers) and also polycrystalline material (gray line). It displays a *T*_*c*_ in excess of 280 K. Adapted with permission from ref (87). Copyright 2019 American Chemical Society.

One aspect of Fe_3_GeTe_2_ is that its Curie–Weiss susceptibility in the paramagnetic state yields effective moments ranging from 3.9 to 4.9 μ_B_/Fe for Fe fractions *x* ranging from 2.69 to 2.97, in contrast to neutron scattering that finds values ranging from 1.4 to 2.18 μ_B_/Fe for *x* = 2.71 and 2.9, respectively.^323^ Magnetization measurements on the other hand, yield a saturation of moment of ∼ (1.2 ± 1) μ_B_/Fe (Figure 29).^323,327^ According to neutron scattering and magnetization measurements the moments point along the *c*-axis forming a collinear arrangement.^323,327^ The Rhodes–Wohlfarth ratio (RWR), defined as *P*_*c*_/*P*_*s*_ with *P*_*c*_ obtained from the effective moment  where *P*_*s*_ is the saturation moment obtained in the ordered state^331,332^ is expected to be 1 for a localized system and larger in an itinerant system. Observed large values of RWR ∼ 3.3 in crystals with Fe vacancies and ∼3.8 reported in CVT-grown single crystals without Fe vacancy indicate a possible Kondo coupling, weak itinerant character and/or strong spin fluctuations in the ground state.^301,322,333^

This collinear spin arrangement leads to stripe-like magnetic domains according to *in situ* LTEM.^25,27,334^ Interestingly, application of a magnetic field along the *c*-axis, induces the formation of magnetic bubbles or magnetic skyrmions as the domains having spins opposed to the field are suppressed by it.^25,334^ Skyrmions could result from the DMI since the inequivalent Fe sites form a lattice that lacks inversion symmetry.^252^ The size of these domains are susceptible to manipulation *via* electrical current pulses which seemingly can also induce skyrmion bubbles^334^ These observations suggest that this compound has a sizable potential for spintronic applications.

Yet another interesting aspect of Fe_3_GeTe_2_ is its very large anomalous Hall response claimed to result from the existence of a nodal line and its associated Berry curvature texture. This leads to very large anomalous Hall coefficients and anomalous Hall angles; the anomalous Hall coefficients is somewhat decreased in crystals Fe_3−δ_GeTe_2_ crystals with Fe vacancies and smaller *T*_*c*_.^335,336^ Therefore, Fe_3_GeTe_2_ would correspond to a rare example of a FM topological nodal line semimetallic system for which electronic correlations are claimed to be relevant.^336,337^

**Figure 30:**
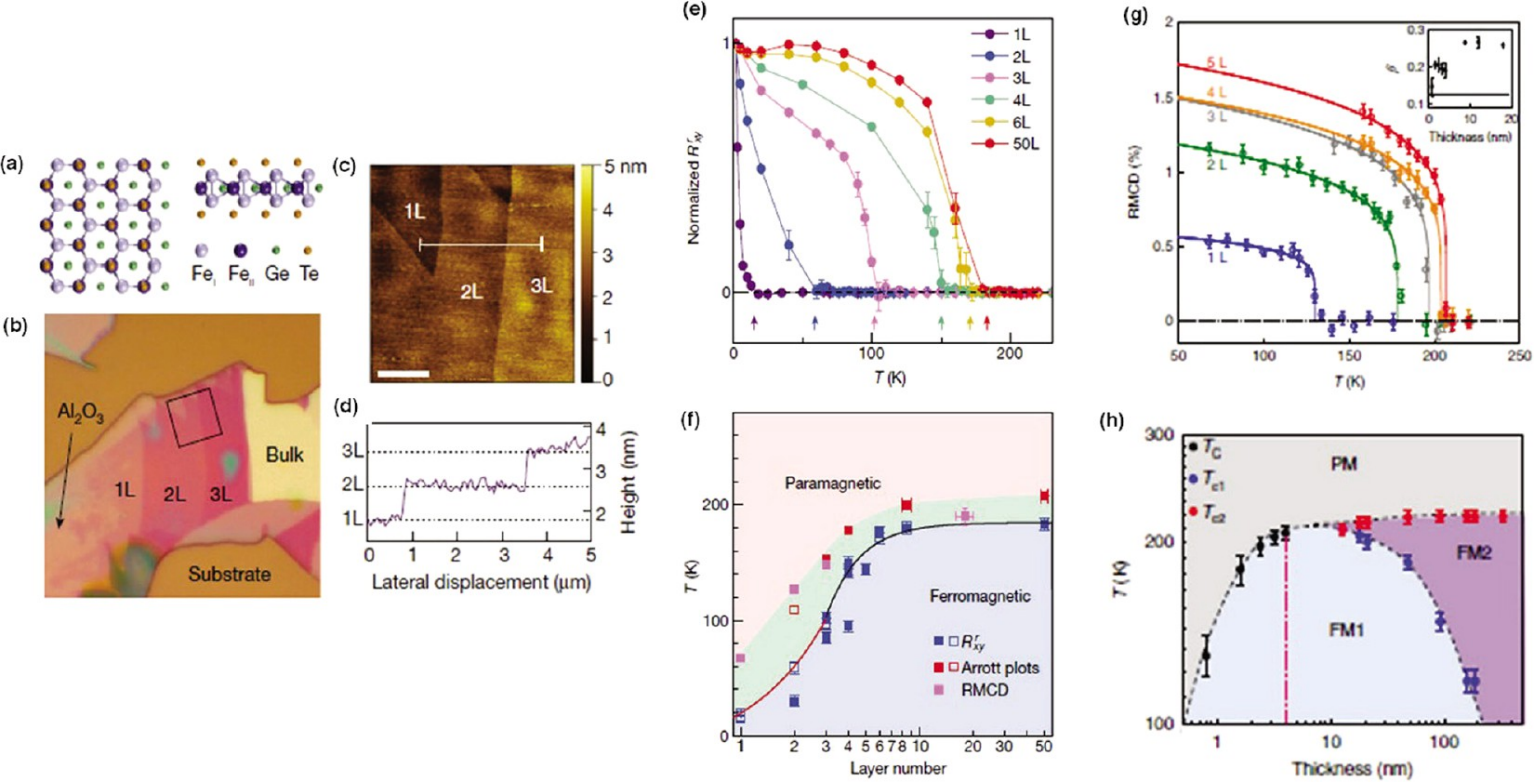
(a) Atomic structure of monolayer Fe_3_GeTe_2_. The left panel shows the view along [001]; the right panel shows the view along [010]. Bulk Fe_3_GeTe_2_ is a layered crystal with an interlayer vdW gap of 2.95 Å. Fe_I_ and Fe_II_ represent the two inequivalent Fe sites in the +3 and +2 oxidation states, respectively. (b) Optical image of typical few-layer flakes exfoliated on an Al_2_O_3_ thin film. (c) Atomic force microscopy image of the area marked by the square in (b). Mono- and few-layer flakes are clearly visible. Scale bar, 2 μm. (d) Cross-sectional profile of the Fe_3_GeTe_2_ flakes along the white line in (c). The steps are 0.8 nm in height, or consistent with the thickness (0.8 nm) of monolayer (1L) Fe_3_GeTe_2_. (e) Normalized remanent anomalous Hall resistance  as a function of temperature obtained from Fe_3_GeTe_2_ thin-flake samples with varying numbers of layers. Arrows mark the FM transition temperature *T*_*c*_. (f) Phase diagram of Fe_3_GeTe_2_ as layer number and temperature are varied. *T*_*c*_ values are determined from anomalous Hall effect, Arrott plots and RMCD are displayed in blue, red and magenta, respectively. (g) Remanent RMCD signal as a function of temperature for a sequence of selected few-layer flakes (1 L, monolayer; 2 L, bilayer; 3 L, trilayer; 4 L, four layers; 5 L, five layer). The solid lines are least-squares criticality fits of the form α(1 – *T*/*T*_c_)^β^. Inset: derived values of the exponent β plotted as a function of thickness. (h) Thickness-temperature phase diagram. PM denotes the region in which the flake is paramagnetic, FM1 that in which it is FM with a single domain and FM2 that in which the flake exhibits labyrinthine or stripe domains. The transition temperatures, *T*_*c*_, *T*_*c*1_, and *T*_*c*2_, are based on the temperature-dependent RMCD or anomalous Hall effect measurements for each flake thickness. The red dashed line denotes the critical thickness at which a dimensional crossover occurs. All panels are adapted with permission from ref (12). Copyright 2018 Springer Nature.

Perhaps more important is the fact that it was demonstrated that Fe_3_GeTe_2_ is exfoliable while still exhibiting robust ferromagnetism with a strong perpendicular anisotropy when thinned down to the monolayer limit.^77^ A study focused on layer-dependent properties reveals a crossover from 3D to 2D Ising ferromagnetism for thicknesses below 4 nm (five layers), which is accompanied by a fast drop of *T*_*c*_ from 207 to 130 K in the monolayer limit (Figure 30).^77^ For flakes thicker than ∼15 nm, a distinct magnetic behavior is emerges within an intermediate temperature range, due to formation of labyrinthine domain patterns.^77^ The persistence of itinerant ferromagnetism down to the monolayer limit is confirmed by ref.^12^ But upon exfoliation *T*_*c*_ is confirmed to be suppressed relative to the bulk *T*_*c*_ of 205 K in pristine bulk Fe_3_GeTe_2_.^12^ Utilization of solid ionic gating, however, raises *T*_*c*_ to room temperature, which is considerably higher than the bulk *T*_*c*_. The gate-tunable room-temperature ferromagnetism in 2D Fe_3_GeTe_2_ is important for potential voltage-controlled magnetoelectronics based on atomically thin vdW crystals.^8,10,14,338^ This conclusion is particularly pertinent if one considers other members of this series, like Fe_4_GeTe_2_ and Fe_5_GeTe_2_ which can display *T*_*c*_s as high as 280 and 310 K,^88,339^ respectively depending on the Fe content.

### Challenges and Perspectives

Persistence of magnetic order in nanofabricated monolayer and few-layer materials stems from magnetic anisotropy since rotational symmetry can not be spontaneously broken at finite temperature in an isotropic 2D system with short-range interactions, as formulated in the Mermin-Wagner theorem.^40^ Single-ion anisotropy and anisotropic exchange both could contribute to a spin wave gap which suppresses fluctuations and enables magnetic order in 2D limit.^340^ A promising direction for high-*T*_*c*_ 2D vdW insulating magnet design is a computational search with the spin wave gap in the magnon dispersion as a descriptor of magnetic state in 2D;^341^ the gap arises in the 2D vdW crystals due to magnetic anisotropy.^235^ In insulating magnetic hexagonal and honeycomb lattices high Curie temperatures are facilitated by the exchange anisotropy whereas the effects of single ion are similar except for *S* = 1/2 where an energy shift could be expected.^342^ Critical scaling behavior is a good and relatively rapid probe of the nature of the magnetic interaction and underlying anisotropy.

Whereas no predictive theory for metallic high-*T*_*c*_ 2D vdW magnets exists at the moment, it should be noted that in 2D metallic magnets with strong XY anisotropy, a quantum critical point is predicted by varying order/disorder in the lattice.^343^ Future materials in this class are likely to host a high proportion of magnetic atoms separated by a vdW gap and the theory should account for possibly complex magnetic sublattices. We note that spin density waves in metallic 2D vdW magnets might induce a periodic charge modulation, particularly at Fermi surfaces prone to nesting behavior.^18,344,345^ As seen on the example of Fe_5_GeTe_2_, critical behavior and scaling analysis should yield exponents consistent with 3D magnetic behavior for small vdW gaps (Table 7).

2D disordered Ising vdW magnets are also subjected to universal scaling behavior in the critical region of disorder-induced phase transition within the random field Ising model; the phase transition separates regions of smooth and step-like *M*(*H*).^346^ In the step-like *M*(*H*) region majority of spins flip in a single system-spanning avalanche. The scaling behavior, critical exponents and correlation length could give some insight into avalanche size distribution. The scaling also persists in variable thicknesses^347^ and it could be relevant in disordered 2D vdW magnetic crystals.

## Probing the Magnetic Properties of Layered Materials *via* Elementary μ-Particles

### Muon Spin Rotation (μSR)

The acronym μSR stands for muon spin rotation, or relaxation, or resonance, depending respectively on whether the muon spin motion is predominantly a rotation (more precisely a precession around a magnetic field), or a relaxation toward an equilibrium direction, or a more complex dynamics dictated by the addition of short radio frequency pulses.

### Production of Spin Polarized Muon Beams and Parity Violating Decay

The muon is an elementary particle similar to the electron or positron, with a unitary positive or negative electric charge (±1) and a spin of 1/2.^351^ The muon is a particle belonging to the family of the leptons with an average lifetime of τ_μ_ = 2.2 μs. The muon mass is about one-ninth of the proton mass or, alternatively, about 200 times the electron mass.^351^ Note that for experiments in condensed matter physics mainly the positive muon is used. Beams of positive muons are artificially produced using proton accelerators. High energy proton beams are fired onto a target (usually graphite) to produce pions *via* the following process:
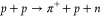
5where *p* denotes the proton and *n* is a neutron. The pions π^+^ decay with a lifetime of τ_π_ = 26 ns into muons:
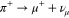
6where ν_μ_ is a muon neutrino. Lets consider pions which are produced at rest in the laboratory frame. According to the momentum conservation law, the muon μ^+^ and the neutrino ν_μ_ must have equal and opposite momentum. Since the pion π^+^ has zero spin the muon spin must be opposite to the neutrino spin. An interesting property of the neutrino is that its spin is aligned antiparallel to its momentum (it has negative helicity). This implies that the muon also has negative helicity. Thus, by selecting pions which stop in the target a beam of 100% spin-polarized muons is produced. This is the method most commonly used for producing muon beams for condensed matter physics research.^352^ Note that the muons produced by the above-mentioned way are called surface muons,^353^ and have a well-defined kinetic energy of 4.1 Mev and a corresponding momentum of 29.8 MeV/c.

When a beam of the spin polarized muons is implanted into a specimen of interest,^354^ the muons thermalize in the sample within typically 1 ns. Muons with positive charge stop at interstitial positions away from positively charged ions. In the presence of a magnetic field, the spin of the implanted muon precesses around the direction of the local magnetic field *B* with the Larmor frequency:
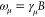
7where γ_μ_ = 2π·135.5 MHz/*T* is the gyromagnetic ratio of the muon. An implanted μ^+^ in the sample will decay after a mean lifetime of τ_μ_ = 2.2 μs. The muon decay is a three body process:
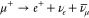
8where *e*^+^ denotes the positron, ν_*e*_ the electron neutrino, and  the muon antineutrino. The kinetic energy of the emitted positron may vary continuously between zero and *E*_max_ = 52.3 MeV. Because of the parity violating decay of the muon (the decay involves the weak interaction) the decay positrons are emitted preferentially along the direction of muon spin. By measuring the anisotropy of the decay positrons from a bunch of muons, the statistical average direction of the spin polarization of the muon ensemble is determined. This in turn reflects the spatial and temporal distribution of magnetic fields at the muon site.

The μSR technique is made possible by the properties of the pion and the muon: (i) Due to parity violation in the decay of pions, surface muons are 100% spin polarized, (ii) the positron is preferentially emitted along the direction of the muon spin at decay time, and (iii) the muon has a magnetic moment and its spin precesses around a magnetic field with the Larmor frequency.

Due to the large magnetic moment of the muon, there is high sensitivity to extremely small magnetic moments (down to 10^–3^–10^–4^ μ_B_) and the broad time window of 10^–4^ s to 10^–11^ s makes μSR a powerful tool to investigate magnetism in solid state physics.^355−357^ Moreover, the μSR technique has a time window for the study of magnetic fluctuations in materials, which is complementary to other experimental techniques such as neutron scattering, NMR or magnetic susceptibility. In addition to magnetism, this technique allows to study interesting problems related to superconductivity,^358−370^ chemical kinetics, diffusion, molecular dynamics, and semiconductor physics. One can also probe magnetic and superconducting properties at the surface of a superconductor using ultralow energy muons.^361,371−373^ In this chapter a brief introduction to the μSR technique and its applications to the study of magnetic materials are presented. A detailed description of the μSR technique can be found in textbooks^374,375^ or in review articles.^351,355−357,376−379^

### Principle of a μSR Experiment

**Figure 31:**
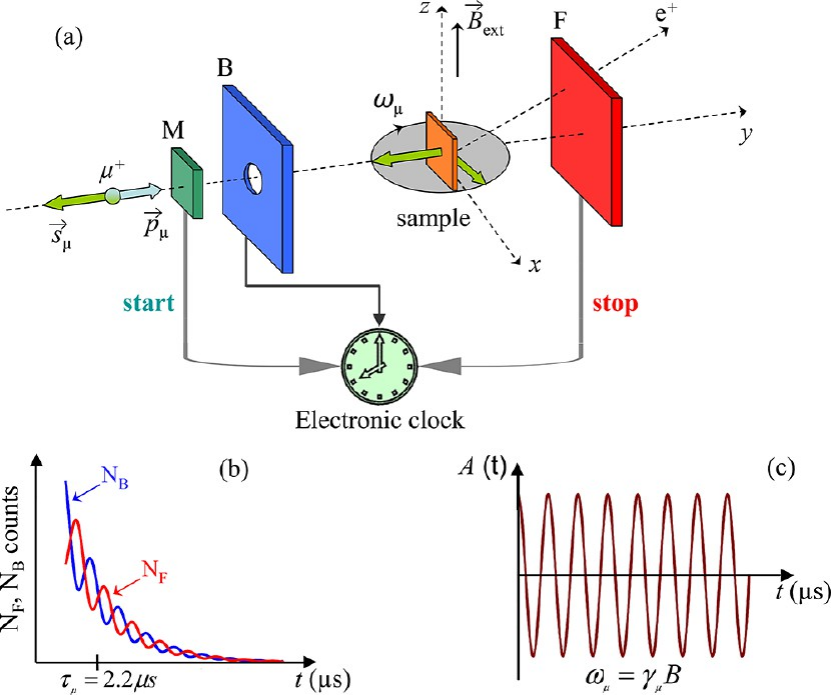
Principle of a μSR experiment. (a) Overview of the experimental setup. Spin polarized muons with spin *S*_μ_ antiparallel to the momentum *p*_μ_ are implanted in the sample placed between the forward (F) and the backward (B) positron detectors. A clock is started at the time the muon goes through the muon detector (M) and is stopped as soon as the decay positron is detected in the detectors F or B. Adapted with permission from ref (348). Copyright Swiss Physical Society. (b) The number of detected positrons *N*_*F*_ and *N*_*B*_ as a function of time for the forward and backward detector, respectively. Reproduced with permissions from ref (349). Copyright University of Zurich. (c) The so-called asymmetry (or μSR) signal is obtained by essentially building the difference between *N*_*F*_ and *N*_*B*_ (eq 2). All panels are adapted with permission under a Creative Common CC BY license from ref (350). Copyright 2019 MDPI.

The μSR method is based on the observation of the time evolution of the spin polarization *P⃗* (*t*) of the muon ensemble. A schematic layout of a μSR experiment is shown in Figure 31a. In a μSR experiments an intense beam (*p*_μ_ = 29 MeV/c) of 100% spin-polarized muons is stopped in the sample (see Figure 31a). Currently available instruments allow essentially a background free μSR measurement at ambient conditions.^377^ The positively charged muons thermalize in the sample at interstitial lattice sites, where they act as magnetic microprobes. In a magnetic material the muons spin precesses in the local field *B*_μ_ at the muon site with the Larmor frequency ν_μ_ = γ_μ_/(2π)*B*_μ_ (muon gyromagnetic ratio γ_μ_/(2π) = 135.5 MHz T^–1^). The muons μ^+^ implanted into the sample will decay after a mean lifetime of τ_μ_ = 2.2 μs, emitting a fast positron *e*^+^ preferentially along their spin direction. Various detectors placed around the sample track the incoming μ^+^ and the outgoing *e*^+^ (see Figure 31a). When the muon detector records the arrival of a μ in the specimen, the electronic clock starts. The clock is stopped when the decay positron *e*^+^ is registered in one of the *e*^+^ detectors, and the measured time interval is stored in a histogramming memory. In this way a positron-count *versus* time histogram is formed (Figure 31b). A muon decay event requires that within a certain time interval after a μ^+^ has stopped in the sample a *e*^+^ is detected. This time interval extends usually over several muon lifetimes (*e.g.*, 10 μs). After a number of muons has stopped in the sample, one obtains a histogram for the forward (*N*_*e*^+^F_) and the backward *N*_*e*^+^B_ detectors as shown in Figure 31b, which in the ideal case has the following form:
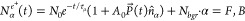
9Here, the exponential factor accounts for the radioactive muon decay. *P⃗* (*t*) is the muon-spin polarization function with the unit vector  (α = *F*,*B*) with respect to the incoming muon spin polarization. *N*_0_ is number of positrons at the initial time *t* = 0. *N*_bgr_ is a background contribution due to uncorrelated starts and stops. *A*_0_ is the initial asymmetry, depending on different experimental factors, such as the detector solid angle, efficiency, absorption, and scattering of positrons in the material. Typical values of *A*_0_ are between 0.2 and 0.3.

Since the positrons are emitted predominantly in the direction of the muon spin which precesses with ω_μ_, the forward and backward detectors will detect a signal oscillating with the same frequency. In order to remove the exponential decay due to the finite lifetime of the muon, the so-called asymmetry signal *A*(*t*) is calculated (see Figure 31c):
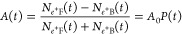
10where, *N*_*e*^+^F_(*t*) and *N*_*e*^+^B_(*t*) are the number of positrons detected in the forward and backward detectors, respectively. The quantities *A*(*t*) and *P*(*t*) depend sensitively on the spatial distribution and dynamical fluctuations of the magnetic environment of the muons. Hence, these functions allow to study interesting physics of the investigated system.

**Figure 32:**
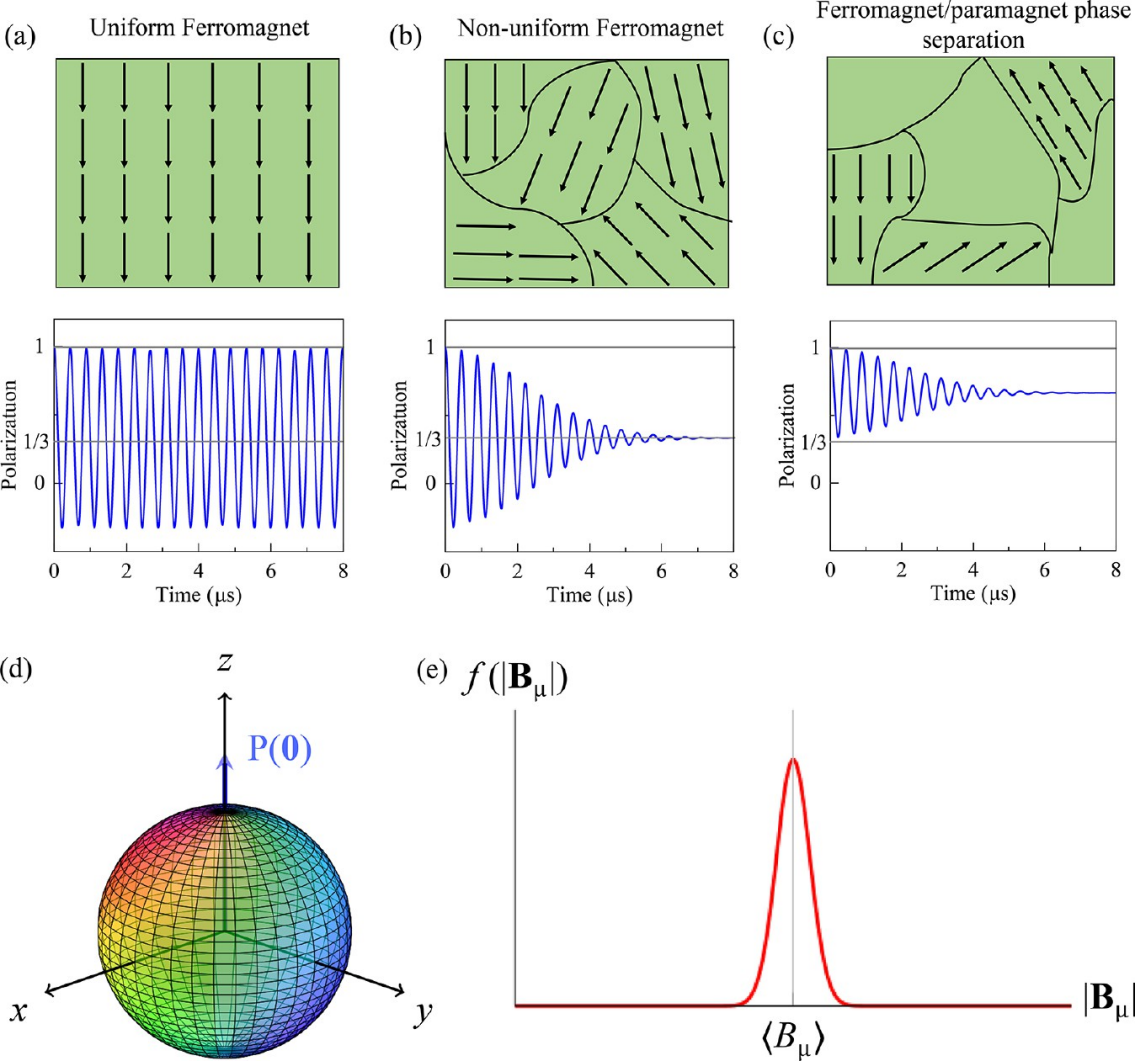
(a-c) Schematic illustration of the magnetically homogeneous (*i.e.*, full volume magnetic) (d), inhomogeneous (full volume magnetic, but with domains) and phase separated (*i.e.*, part of the volume magnetic and part paramagnetic) polycrystalline samples and the corresponding μSR spectra. The 1/3 nonoscillating μSR signal fraction originates from the spatial averaging in powder samples where 1/3 of the magnetic field components are parallel to the muon spin and do not cause muon spin precession. (d, e) Isotropic Gaussian field distribution for polycrystalline sample. Panels (a–c) adapted with permission under a Creative Common CC BY license from ref (350). Copyright 2019 MDPI.

In μSR experiments two different magnetic field configurations are used: (i) Transverse field (TF) μSR involves the application of an external field perpendicular to the initial direction of the muon spin polarization. The muon spin precesses around the transverse field, with a frequency that is proportional to the size of the field at the muon site in the material. (ii) In the longitudinal field (LF) configuration the magnetic field is applied parallel to the initial direction of the muon spin polarization. The time evolution of the muon spin polarization along its initial direction is measured in this configuration. Measurements are often carried out in the absence of external magnetic field, a configuration called zero-field (ZF) μSR. In this configuration the frequency of an obtained μSR signal is proportional to the internal magnetic field, from which the size of the ordered moment and thus the magnetic order parameter is calculated. The capability of studying materials in zero external field is a big advantage over other magnetic resonance techniques.

### Applications of μSR in Magnetism

**Figure 33:**
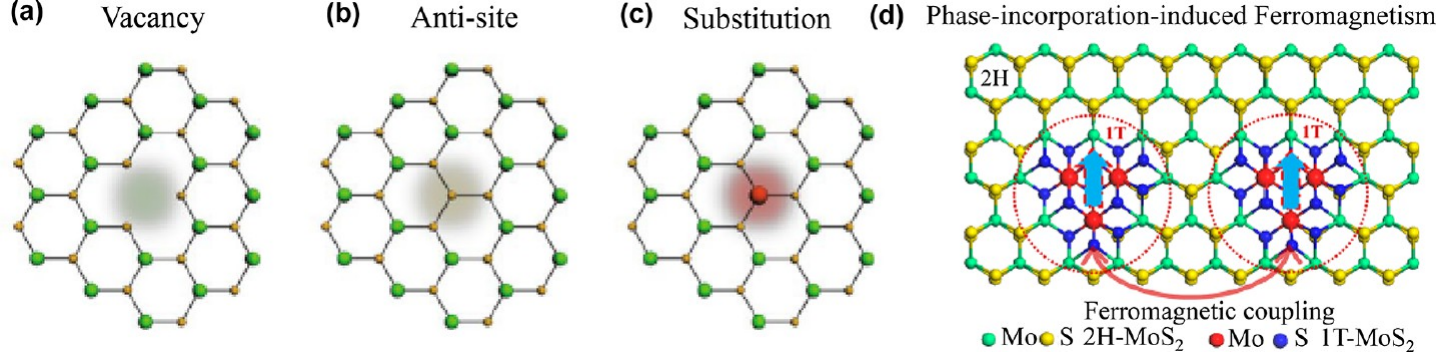
Types of intrinsic disorder in TMDs: (a) vacancy, (b) antisite, (c) substitution. Panels (a–c) adapted with permission from ref (380). Copyright 2019 Springer Nature. (d) Schematic diagram of the phase incorporation strategy to achieve ferromagnetism of 2H-MoS_2_ nanosheets. Adapted with permission from ref (381). Copyright 2015 American Chemical Society.

μSR has been widely applied to magnetic materials due to the high sensitivity of the muon to small fields and its capability to probe both static and dynamic local field distributions. ZF μSR is used to investigate microscopic magnetic properties of solids. If the local magnetic field *B⃗* (*r⃗*) at the muon site is pointing under an angle θ with respect to the initial muon spin polarization, the decay positron asymmetry is given by^351^
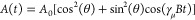
11where *A*_0_ is the maximal value of the asymmetry. Further assuming that the random fields are isotropic and each component can be represented by a Gaussian distribution of width Δ/γ, then a statistical average of this distribution yields
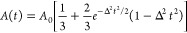
12This function was obtained in a general stochastic treatment of Kubo and Toyabe.^378^ The form of the distribution of internal magnetic fields influences the form of the μSR signal.^351,355,356^ Thus, by analyzing the observed muon-spin time evolution, the magnetic field distribution inside the sample can be obtained. For clarity, the time traces in Figure 33a-c shows the expected time evolution of the muon spin polarization for three different cases of magnetically ordered polycrystalline sample: fully magnetic and magnetically homogeneous (a), full volume magnetic and inhomogeneous (b) and phase separation between magnetic and paramagnetic regions (c). The muons stopping in the homogeneous sample will sense the same magnetic field and their spin will precess around the internal field and the μSR signal is characterized by maximum amplitude and zero depolarization (Figure 33a). If there is an inhomogeneous static internal field in the sample, different muons will precess at slightly different frequencies. This leads to a progressive dephasing of the μSR signal, and the oscillations in the μSR time spectra will be damped (see Figure 33b). In some cases the signal is strongly damped, so that the oscillation will not be observed, and the resulting muon spin polarization will be averaged out to zero. Then, at a magnetic phase transition, if no wiggles are observed in the μSR signal, one expects a drop in the effective initial asymmetry from *A*_0_ in the paramagnetic state to *A*_0_ = 1/3 in the ordered state.^355^ However, this effect could also be due to fluctuations of the internal field. μSR is capable to distinguish between these two possibilities by performing a LF-μSR experiment. In a longitudinal field inhomogeneous line broadening and fluctuations lead to different μSR time spectra. Since muons stop uniformly throughout a sample, and the amplitudes of the μSR signals arising from the different regions of the sample are proportional to the volume of the sample occupied by a particular phase, the presence of paramagnetic regions will result in the reduction of the signal amplitude as shown in Figure 33c. This schematics is a simple illustration of the fact that μSR is capable to provide quantitative information on coexisting and competing phases in a material. We note that in a single crystal the amplitude of the oscillatory component depends not only on the ordered volume fraction but also on the angle between magnetic field and muon spin polarization. Thus, the amplitude indicates the direction of the internal field.

### Magnetism in Semiconducting vdW Materials

TMD systems have honeycomb layers of 2D sheets with the common formula MX_2_, where M is a transition metal (M = Ti, Zr, Hf, V, Nb, Ta, Mo, W or Re) and X is a chalcogen (X = S, Se, or Te). These compounds crystallize in different structural phases resulting from different stacking of the individual MX_2_ layers, with vdW bonding between them. The 2H forms of these compounds are semiconducting and can be mechanically exfoliated to a monolayer. In bulk form, 2H-MoTe_2_ has an indirect band gap of 0.88 eV. The properties of the TMDs, especially in the monolayer form, have triggered a great experience in device applications such as magnetoresistance and spintronics, high on/off ratio transistors, optoelectronics, valley-optoelectronics, superconductors and hydrogen storage. Many of these interesting properties arise due to the strong spin–orbit interaction present in these materials from the heavy metal ion. Until recently, the family of TMDs has been missing one crucial member: a magnetic semiconductor. The situation has changed over the past few years with the discovery of layered semiconducting magnetic crystals, like for example CrI_3_^5,62,227,383^ and VI_3_.^276,277,384^ Unconventional magnetism in the semiconducting Mo-based TMD systems 2H-MoTe_2_ and 2H-MoSe_2_^382^ was also recently discovered. These observations suggest an importance of magnetic interactions in electronic structures of TMDs, and extend general commonalities of various unconventional superconductors to this important family of 2D conductor. This finding helps to study the interplay of 2D physics, semiconducting properties and magnetism in TMDs. It also provides a material platform to obtain tunable magnetic semiconductors, forming the basis for spintronics. To date, the origin and the nature of this magnetic order remains an unsolved issue. Thus, systematic magnetic, electronic and structural studies in the bulk and in thin films of semiconducting TMDs are essential. Here, we review recent experimental progress on magnetism of semiconducting TMDs with the emphasis on the results from the local-magnetic probe such as muon-spin rotation.

### Magnetism and Intrinsic Defects in Mo-Based TMDs

While there are many studies focused on the spin–orbit coupling and the interesting consequences for electrical and optical properties in semiconducting TMDs, there are very limited, and mostly theoretical, studies on the intrinsic magnetism. Specifically, theoretical works show that the pristine lattice of 2H Mo- and W-based TMDs are nonmagnetic, because the Mo^4+^ ions are in a trigonal prismatic local coordination in which the two 4d electrons are spin-antiparallel and the net magnetic moment is zero. If the Mo^4+^ 4d electron configuration could be tuned to have nonzero magnetic moment, the 2H-, Mo-, and W-based TMDs could display magnetic properties. This can be achieved either by the presence of various intrinsic crystalline imperfections/disorder (Figure 33a–c) or using variety of external methods, including atomic doping, and phase incorporation (Figure 33d).

#### Intrinsic Defects

The types of defect observed in TMDs depends on the fabrication process.^380^ The most common experimental techniques used to produce large chunks of TMDs are mechanical exfoliation, CVD, and physical vapor deposition. Defects usually play an important role in tailoring electronic, optical and magnetic properties.^91,380,386−388^ It was found from first-principles calculations that (1) MoSe_2_, MoTe_2_, and WS_2_ exhibit surprising confinement-induced indirect–direct-gap crossover and (2) certain defects induce magnetism in TMDs. In particular, the Mo vacancy defect and the antisite defects in these materials can induce spin-polarization and long-range magnetic coupling. Certain TMDs was even shown to exhibit an exceptionally large magnetic moment due to these defects. The linear atomic doping^386^ of TMDs was also shown to give rise to nonlocalized defect states (similar to line vacancy defects) and to a long-range magnetism. For instance, F and Fe atoms linearly doped MoS_2_ was predicted to be FM semimetals, while Mn or Co atoms doped MoS_2_ are FM semiconductors.^386^ First-principles calculations also predicted that macroscopic ferromagnetism of MoS_2_ nanosheets could be introduced by biaxial strain.^381^ Applied strain was also shown to induce a transition from FM semiconductor to a half-metallic state in Mn or Co linear-doped MoS_2_.^386^

#### Atomic Doping

Different types of atoms have been used to initiate magnetism. On the basis of previous studies, transitional-metal (TM) atom doping can effectively induce magnetism into MoS_2_. For example, magnetism is observed for Mn,^389−397^ Fe,^390,392−395^ Co,^390,392−395,398^ Cr,^390,393^ Zn,^392,393^ Cd,^393^ and Hg^393^ doping, and the magnetic moment of the 3d TM-doped MoS_2_ increases with the d-band filling of the TM dopants.^390^ Additional, spin polarization was found in MoS_2_ with S atoms replaced by incomplete d-band atoms, such as Fe and V,^396^ and Group VA and III elements, such as N, P, As, B, Al, and Ga.^395^ Moreover, adsorption of various atoms, such as H, B, C, N, and F, is also effective to turn MoS_2_ from nonmagnetic to magnetism.^397^ H-absorbed WS_2_, MoSe_2_, and MoTe_2_ monolayers and F-adsorbed WS_2_ and MoSe_2_ monolayers show long-range AFM coupling between local moments even when their distance is as long as ∼12 Å. It is worth noting that no magnetism is observed in V-doped MoS_2_ based on ref (393), but according to refs (390) and (395), V doping induces more than 1 μ_B_ magnetic moments into monolayer MoS_2_. And based on the study of Lee *et al*.,^392^ the nonmagnetic element Cu doping brings strong magnetism into the doped MoS_2_. Calculations indicate that V and Mn are promising candidates for engineering and manipulating the magnetism of the 2D TMDs.

#### Phase Incorporation

It was anticipated^381^ that introducing the 1T phase into the 2H nanosheets could be an effective strategy for combining the semiconducting and magnetic features of TMDs. Within this framework, 1T-MoS_2_ phase was introduced into the matrix of 2H-MoS_2_ nanosheet and robust FM response with a magnetic moment of 0.25 μ_B_/Mo at room temperature was observed.^381^ Since the crystal structures and the Mo atomic positions are identical in both 1T-MoS_2_ and 2H-MoS_2_, such an incorporation does not change the distributions of Mo ions in 2H-MoS_2_ and does not hamper its practical applications. It was further revealed that the interaction between the Mo 4d states of the 1T-MoS_2_ dopant and a bandgap energy state induced by sulfur vacancy is the origin of the ferromagnetism of the phase-incorporated MoS_2_ nanosheets. Such a phase-incorporation strategy can be also used for the bulk materials.

**Figure 34:**
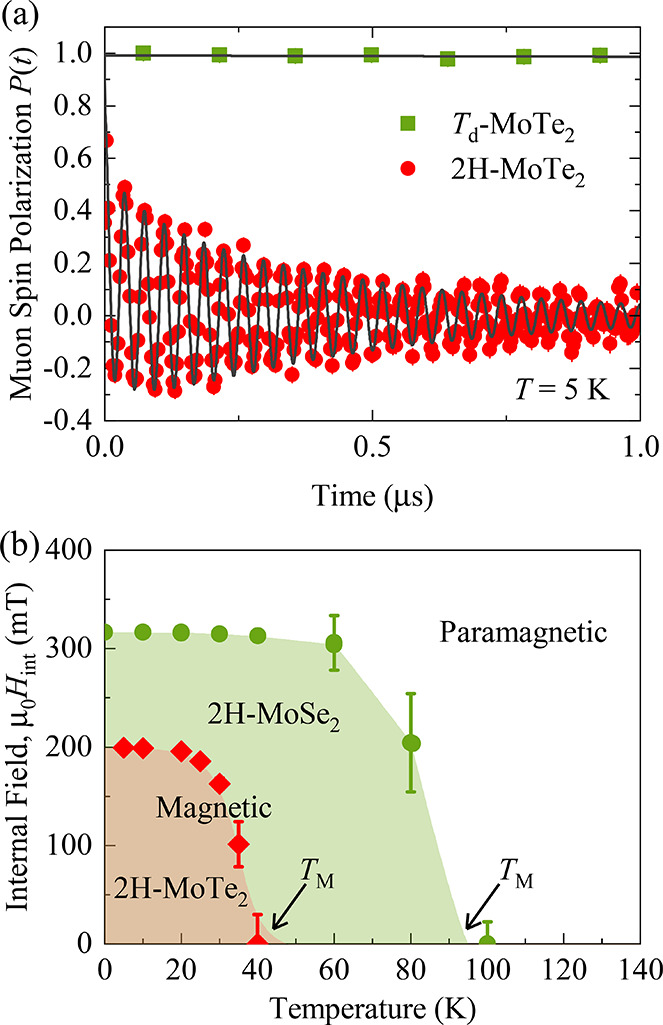
(a) ZF μSR time spectra for the single crystal samples of *T*_d_-MoTe_2_ and 2H-MoTe_2_ recorded at *T* = 5 K. Adapted with permission under a Creative Common CC BY license from ref (350). Copyright 2019 MDPI. (b) Temperature dependence of the internal field *H*_*int*_ of 2H-MoTe_2_, 2H-MoSe_2_ as a function of temperature. Adapted with permission under a Creative Common CC BY-NC 4.0 license from refs (382). Copyright 2019 AAAS.

In the following, we will discuss the clearest evidence of involvement of magnetism in semiconducting TMDs 2H-MoTe_2_ and 2H-MoSe_2_ using local probe such as μSR. The muons in 2H-MoTe_2_ has one stable stopping site inside the Mo-layer and a metastable site in the vdW gap.^399^ It was observed that at low temperature, the spins of the implanted muons precess at an oscillating frequency (Figure 34) which correspond to an internal field μ_0_H_*int*_ = 200(2) mT at stable site inside the Mo-layer. There is a smooth increase of μ_0_H_*int*_ below *T*_M_ ≃ 40 and 100 K for 2H-MoTe_2_ and 2H-MoSe_2_, respectively (Figure 34b). The observation of a spontaneous muon spin precession is a clear signature of the involvement of magnetism below *T*_M_ ≃ 40 and 100 K in the bulk of MoTe_2_ and MoSe_2_, respectively.^382^ Moreover, we find that the bulk magnetic response observed with μSR in 2H-MoTe_2_ are robust and do not change in the near surface region of the crystals.^399^ This demonstrates potential applicability of 2H-MoTe_2_ for magnetic thin film heterostructures. We also find that the magnetic response is efficiently tuned by hydrostatic pressure.^382^

**Figure 35:**
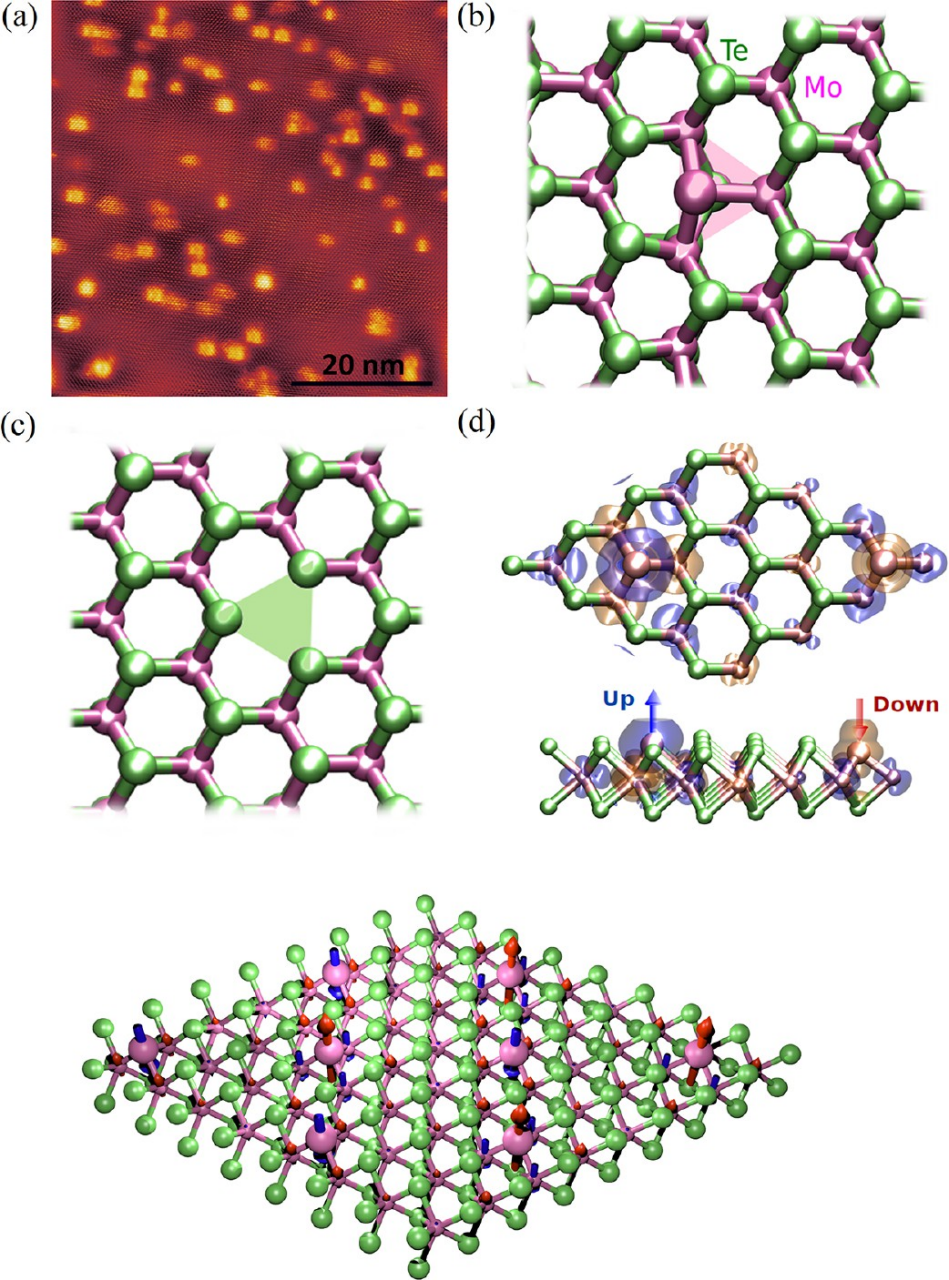
(a) Large-scale atomic-resolution STM topography (20 nm) of the 2H-MoTe_2_ surface. The image reveals an approximately uniform density of two types of defects over the entire surface. (b) DFT+*U*-optimized geometry for Mo_*sub*_ defect. (c) DFT+*U*-optimized geometry of the Mo vacancy Mo_*vac*_. (d) Magnetization density (0.001 electrons/bohr^3^) on the top surface of bulk 2H-MoTe_2_ in AF configuration. Spin-up and spin-down states are shown in faint blue and orange isosurfaces, respectively. Note that spins also couple antiferromagnetically at the local level between the Mo impurity and the nearest Mo atoms. All panels are adapted with permission under a Creative Common CC BY-NC 4.0 license from ref (382). Copyright 2019 AAAS.

**Figure 36:**
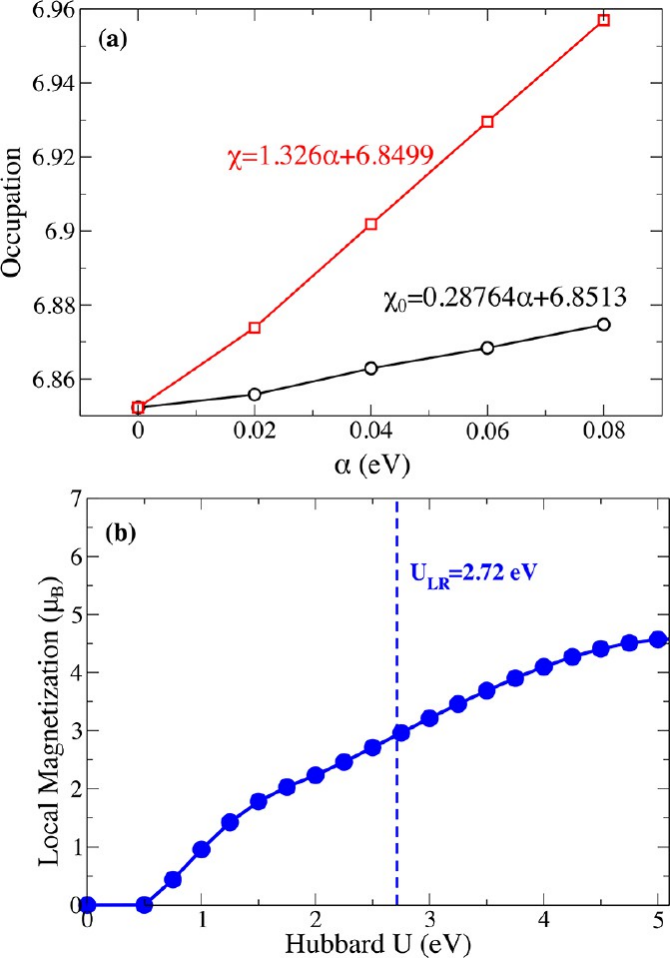
Calculated magnetization of the antisite defect. (a) Occupation number *versus* rigid potential shifts α for antisite defects for the bare, noninteracting potential χ_0_ and the interacting potential χ. From the angular coefficients of both curves we can extract the optimum *U*_*LR*_ for our system, *U*_*LR*_ =  – χ^–1^.^385^ (b) Variation of the local magnetization at the defect antisite *versus U*. At *U* = 0, no magnetic moments are observed as the defect shows a symmetric configuration at the Mo–Mo bonds. At *U* > 0.5 eV, this symmetry is broken and the defect develops an appreciable magnetic moment that increases with *U* as a result of the increased localization of the bands. All panels are adapted with permission under a Creative Common CC BY-NC license from ref (382). Copyright 2019 AAAS.

These results came as a surprise since the previous theoretical work^400^ and simple chemical bonding considerations indicate that the Mo atoms in these samples are in a nonmagnetic 4d^2^ configuration. The origin of this magnetism remains unclear. We note that in the same material intrinsic magnetic Mo antisite defects^382^ were observed by combination of the high-resolution STM^401^ and Hubbard corrected DFT+*U* calculations^387^ (Figure 35a–d). Namely, STM measurements demonstrate the presence of intrinsic dilute self-organized defects. Note that two major defects, i.e., metal-vacancies (Figure 35c) and chalcogen-antisites (Figure 35b) (where a molybdenum atom substitutes the Tellurium/Selenium atom) were found in these materials. In general, TMD crystals do not share the near-perfection of graphene. This is not surprising given that the formation energy for defects in TMDs is much lower than for similar defects in graphene (for example, 7–8 eV for a graphene vacancy versus 4.8 eV for a metal antisite defects in MoTe_2_). The defect concentration is small (∼0.5–1 *%*), but defects are found to have a large electronic impact. Moreover, DFT indicates that at finite values of *U*, a magnetic moment in the range of 0.9 to 2.8 μ_B_ is observed per chalcogen-antisite defect Mo_sub_. But, the metal-vacancy defects Mo_vac_ do not introduce a significant local moment. The Hubbard *U* value used in our simulations is in the range of 0.5 to 4.0 eV to account for strong on-site interactions at the defect. We calculated the local magnetization of the antisite defect as a function of *U*. The strong *U* dependence of moment is found as shown in Figure 36b. We have also explicitly calculated the magnitude of the Hubbard *U* using linear response theory as included in Figure 36a. The magnitude obtained is of *U*_*LR*_ = 2.72 eV for the antisite defect (this value is marked as the dashed line in Figure 36b), which is within the limit of initial range calculated the magnetic properties of the defects. The calculation suggests that the Mo_sub_ defects are coupled antiferromagnetically to the nearest-neighbor Mo atoms, as shown in Figure 35d. The magnetic moments at the nearest-neighbor Mo atoms can reach 0.10 to 0.40 μ_B_/atom, with smaller contributions for second and third neighbors (0.02 to 0.08 μ_B_/atom).

The Te atoms show negligible spin polarization. Although, DFT shows the magnetic defects in these systems, it is difficult to understand how the low-density of the chalcogen-antisite defects can give rise to homogeneous internal magnetic fields, observed in 2H-MoTe_2_ and 2H-MoSe_2_. This may be possible if these defects have electronic coupling to the semiconductor valence electrons. The presence of such spin-polarized itinerant electrons would imply that these materials are dilute magnetic semiconductors. This idea may be partly supported by the recent report on the observation of hidden spin-polarized states in the bulk MoTe_2_.^388^

Another possibility is that muons are trapped near the magnetic defect, senses the large dipolar field created by the defect and produces the coherent oscillations in the μSR signal. Although the exact link between μSR and STM/DFT results^382^ in 2H-MoTe_2_ and 2H-MoSe_2_ is not yet clear, both results together constitute a strong evidence concerning the relevance of magnetic order in the TMDs physics. Our observations also add to the growing evidence that defects in TMDs are important to understand their physical properties. Recently, there have been several reports on magnetism in Mo- and W-based TMDs from bulk magnetization measurements. Namely, the formation of ferromagnetism was reported for V-doped WS_2_^402^ and WSe_2_^93,403^ monolayers with a small amount (∼0.5–4%) of V-content and the materials was classified as a dilute-magnetic semiconductor. Room-temperature hysteresis has been observed for small amounts of vanadium deposited on 2H-MoTe_2_. Defect induced, layer-modulated magnetism was recently reported for ultrathin metallic system PtSe_2_.^91^ Ferromagnetism has also been observed in metallic monolayer 1T-VSe_2_,^18^ which is different relative to semiconducting systems, *i.e.*, 2H-MoTe_2_, 2H-MoSe_2_ and 2H-WSe_2_, 2H-VSe_2_.^404^ Thus, they raise opportunities to obtain tunable magnetic semiconductors, forming the basis for spintronics. Theoretical calculations also predict that apart from defects, hydrogen and transition metal dopants are able to induce spin polarization in MoTe_2_ in the monolayer limit. Since a muon can be considered a light hydrogen isotope, muon induced/enhanced^399^ magnetism has also been discussed. However, magnetic hysteresis is clearly seen in the macroscopic magnetization data of MoTe_2_ and MoSe_2_,^382^ which can be considered as additional, besides μSR, independent piece of evidence for the involvement of magnetism in TMDs.

### Magnetic Phases in vdW Magnet CrI_3_

Besides Mo/W-based TMDs, very interesting magnetic semiconducting TMD system is CrI_3_. Although the experimental investigations of bulk CrI_3_ date back to the 1960s, the temperature-dependent magnetic and structural properties have only recently been reported.^5,62,227^ The presence of a heavy halide atom in CrI_3_ result in marked anisotropy constants and a clear deviations from the paramagnetic regime at relatively high temperatures. Standard bulk magnetization measurements provide clear signatures of long-range magnetic order and a consequent phase-transition at a nonzero temperature. However, they also fail to capture many fine details hidden at microscopic level. Such details are instrumental to distinguish macroscopic ground states with competing magnetic phases. It has recently been found that CrI_3_ exhibit both AF and FM orders in thin layers driven by hydrostatic pressure.^22^ These phases occurred at the same critical temperature with a spatial separation of few hundreds of nm and consequently no prelude of themally activated spin ordering. If competition occurs between phases it is largely unknown but these observations establish a much more intricate scenario than originally pictured for CrI_3_ with many hidden subtleties that have important implications in the ordering of the magnetic domains in the system. We recently reported results of high-resolution μSR spectroscopy, complemented by SQUID magnetometry and large scale micromagnetic simulations, to systematically study the thermal evolution of magnetic states in CrI_3_. This suite of techniques was essential to identify, characterize and understand distinct macroscopic ground states with any competing magnetic phases.

**Figure 37:**
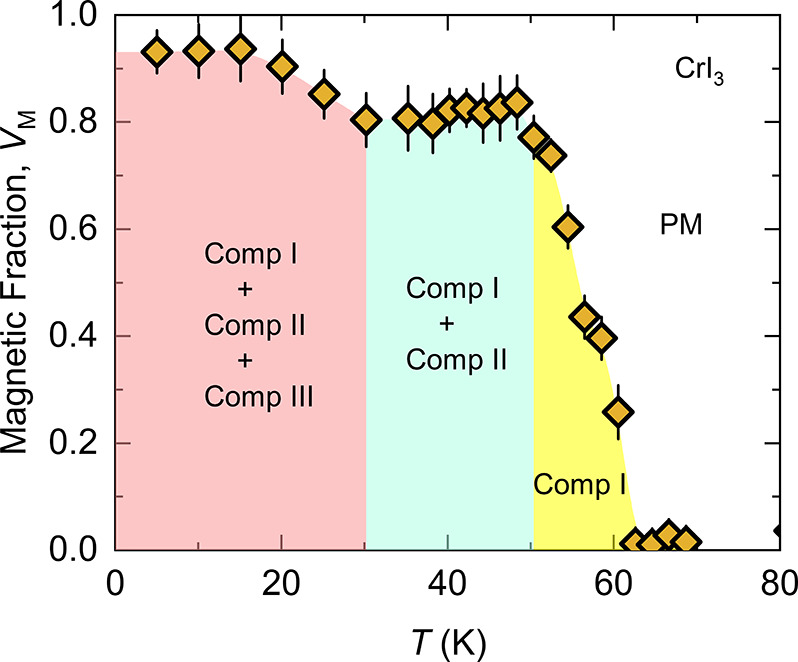
Temperature dependence of the magnetic volume fraction for CrI_3_. Adapted with permission under a Creative Commons CC BY license from ref (383). Copyright 2021 Springer Nature.

The temperature dependence of the total magnetic fraction *V*_m_, determined from μSR experiments,^383^ is shown in Figure 37. The magnetic fraction *V*_m_ does not acquire full volume right below *T*_C1_ ≃ 62 K. Instead, it gradually increases below *T*_C1_ and reaches ≃80% at *T*_C2_ ≃ 50 K. Additional increase of *V*_m_ takes place below *T*_C3_ ≃ 25 K, at which the third strongly damped component appears and reaches nearly ≃100%. The volume wise evolution of magnetic order across *T*_C1_, *T*_C2_ and *T*_C3_ in CrI_3_ strongly suggests the presence of distinct magnetic states in the separate volumes of the sample. The experimental results are supported by macroscale micromagnetic simulations, which revealed three main phases emerged in CrI_3_ as a function of the temperature: disordered-I, ordered, and disordered-II. The main driving force for the formation of these three phases is the coexistence of different crystal structures (monoclinic and rhombohedral) on bulk CrI_3_.^383^

### Magnetic Transitions in vdW Magnet VI_3_

Ferromagnetism below *T*_*C*_ ≃ 50 K was also recently discovered in bulk single crystals of VI_3_.^276,405^ Similar to other transition-metal trihalides, such as CrI_3_, VI_3_ consists of stacked layers in which edgesharing VI_6_ octahedra form a honeycomb lattice. This system VI_3_ was shown to undergo a structural transition at *T*_*s*_ ≃ 78 K, followed by two subsequent FM transitions at *T*_*C*_ ≃ 50 K and  36 K upon cooling.^405^ Namely, using NMR, two magnetically ordered V sites were identified below , whereas only one magnetically ordered V site was observed for .^405^ We studied the magnetism and its temperature dependence in VI_3_ using the μSR technique.

**Figure 38:**
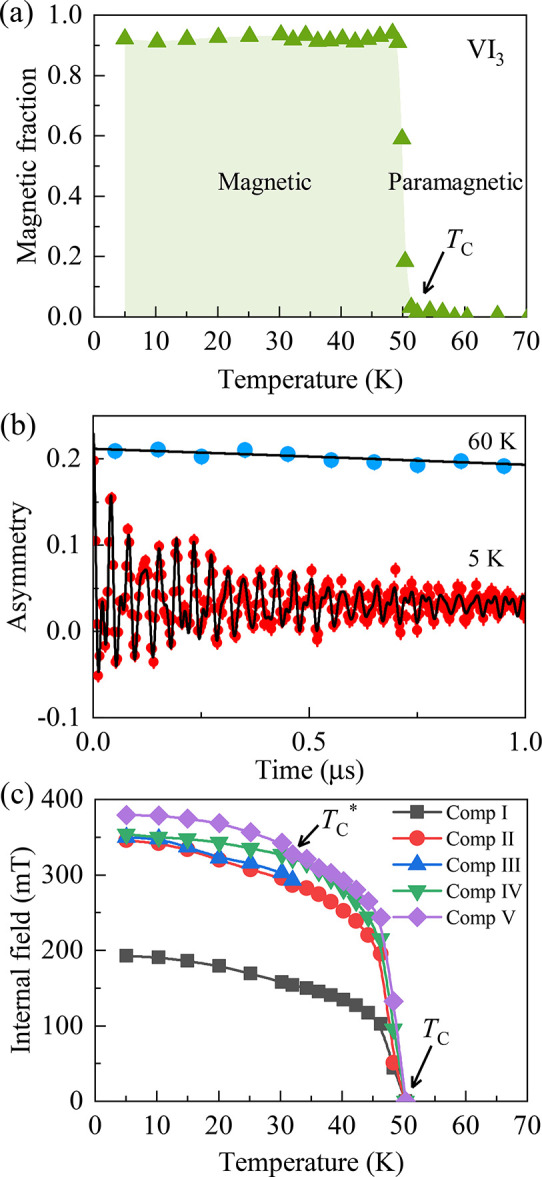
(a) Temperature dependence of the magnetic volume fraction for VI_3_, determined from weak transverse field μSR experiments. (b) Zero-field μSR spectra, recorded at *T* = 5 and 60 K. (c) The temperature dependence of the internal fields *H*_*int*_ for VI_3_. Original figure, no permissions needed.

The temperature-dependent magnetic fraction, shown in Figure 38a, shows a sharp transition from the paramagnetic to the magnetic state with the coexistence of magnetic and paramagnetic regions in the temperature interval 50–52 K, *i.e.*, only very close to the transition. In order to study the detailed temperature evolution of the magnetic order parameter in VI_3_, zero-field μSR measurements were carried out. The ZF-μSR spectra, recorded at temperatures above and below *T*_*C*_ (*T* = 60 and 5 K), is shown in Figure 38b. At *T* = 60 K, the entire sample is in the paramagnetic state as evidenced by the weak μSR depolarization and its Gaussian functional form arising from the interaction between the muon spin and randomly oriented nuclear magnetic moments. At 5 K, five distinct precession frequencies appear in the μSR spectra, which is most likely due to the presence of magnetically inequivalent muon stopping sites, especially in the condition of two magnetically ordered V sites. The temperature dependencies of the internal fields for all the components are shown in Figure 38c. We find that above  32 K, component III disappears as well as components IV and V nearly merge. The decrease of number of components above  could be related to the fact that above there is only one magnetically ordered V site, as shown by NMR measurements. These data along with the previous NMR measurements^405^ point toward a complex temperature evolution of magnetic structure in VI_3_ and call for a detailed understanding of magnetic structures in this compound.

## Spintronics: From Fundamentals to Devices

### General Introduction and Background

The ability to carry electron spin over long distances due to long relaxation times made graphene an excellent candidate for spin electronics giving rise to the emergence of graphene spintronics.^406,407^ Initially graphene, hexagonal boron nitride (hBN) and other vdW materials and heterostructures^408^ were explored as a spin transport channel in lateral geometry setups^409−412^ and/or as a tunnel barrier or part of vertical MTJs^413−416^ partially encouraged by theoretical predictions that they can serve as efficient spin filters.^417,418^ Since 2D materials were believed to be nonmagnetic, significant attention has been paid to induce magnetic moments and exchange splitting within graphene, for instance, *via* introducing defects (*e.g.*, vacancies, adatoms,^419,420^ graphene nanomeshes^421,422^), or alternatively *via* proximity with magnetic insulators^423−425^ or with magnetic metals across a spacer made of 2D hBN.^426,427^

As already mentioned, the skepticism about the absence of standalone 2D materials with intrinsic magnetism was based on the Mermin–Wagner theorem^40^ stating that at any finite temperature no long-range magnetic order is possible due to massive gapless excitations of spin waves (magnons) that destroy it. The theorem was formulated for the case of isotropic Heisenberg model with finite-range interactions, leaving the opportunity to stabilize magnetic order in two dimensions. Indeed, it can be shown that the presence of uniaxial anisotropy (*e.g.*, magnetocrystalline one caused by spin–orbit interaction) gives rise to a magnon excitation gap that quenches their impact on magnetic order lifting Mermin–Wagner restriction and thus resulting in finite Curie temperature (*T*_*C*_).^213^

As the system evolves from 2D toward 3D, the magnon spectrum behavior softens with no anisotropy needed to preserve long-range magnetic order at finite temperature.^213^ In this case, the Curie temperature for systems comprising *n* monolayers, *T*_*C*_(*n*), should evolve according to finite-size scaling formula representing its relative shift from its bulk value  as , where *C* represents characteristic thickness for a given system and λ is the inverse of the bulk correlation length exponent ν reflecting the appropriate universality class.^428−432^ This formula is valid in case of thick layers and alternative expression suitable for larger thickness range was suggested with the relative shift in respect to *T*_*C*_(*n*) of the form  used, for instance, for studies of thin transition metal^433,434^ or ferroelectric films.^435^ The problem is that λ′ disagrees with λ and does not have a physical meaning, but such disagreement seems to be resolved by taking into account spin–spin interactions with its range parameter *N*_0_.^432^

The scaling formula can then be written for two distinct cases. *T*_*C*_ scales according to the power law in the case of thin films with *n* > *N*_0_ and follows the relationship  and, in the case of ultrathin films when *n* < *N*_0_, the scaling becomes linear instead, yielding .^432^ Together with other critical exponents, including those governing temperature dependence behavior of magnetization (β), the scaling behavior analysis provide powerful insights for identifying phase transitions from 3D to 2D magnetism as demonstrated for thin transition metals according to universality hypothesis^436^ and can be used to identify and describe 2D magnetism in vdW heterostructures.^62^

Indeed, *T*_*C*_ behavior as a function of thickness of metallic exfoliated Fe_3_GeTe_2_ (FGT) nanoflakes was shown to follow the aforementioned power law with estimated λ and *N*_0_ values of the order 1.7 and 5 ML, respectively, corresponding to the Heisenberg ferromagnetism.^123^ Similar power law behavior was reported by Deng *et al*.,^12^ with smaller *N*_0_ ∼ 3 ML and larger estimated value of λ = 2.3 ± 0.8, more consistent with mean field behavior but also not excluding 3D Heisenberg magnetism. It is not surprising that it was harder to conclude on the type of magnetism since *N*_0_ indicating a boundary between 3D and 2D magnetism was smaller. It is interesting, however, that they were able to confirm the aforementioned linear scaling for thicknesses smaller than *N*_0_ pointing that they were able to reach ultrathin limit.^12^ A clear crossover from the bulk to 2D ferromagnetism in this vdW system of thickness less than 5 ML reaching 2D Ising model behavior for a FGT monolayer was demonstrated by Fei *et al.*(77) from the analysis of temperature dependence critical exponent β.

FGT has actually emerged as a higher *T*_*C*_ alternative to a previously reported experiment with demonstration of 2D magnetism in Cr_2_Ge_2_Te_6_ (CGT) vdW semiconductors down to the bilayer limit with fine control of transition temperature with low applied magnetic fields.^6^ Another breakthrough experiment demonstrated intrinsic 2D magnetism down to the monolayer limit in insulating exfoliated CrI_3_.^5^ Interestingly, these vdW materials showed layer-dependent magnetism due to behavior alternating between FM and AF states as number of layer increases. Some TMDs, *e.g.*, 1T-VSe_2_^18^ and MnSe_2_,^17^ have been also reported being magnetic in some of their crystallographic phases or as a result of doping.^437^ Ising-type magnetic ordering has also been demonstrated in phosphorus-based insulating antiferromagnets, *e.g.*, in FePS_3_.^32^

Actually, these studies triggered enormous interest in 2D vdW structures since they can be representing part of more general classes or families. For instance, CrI_3_ belongs to the family of chromium trihalides CrX_3_ with X = I, Br or Cl; CGT is part of Cr_2_X_2_Te_6_ with X = Si,Ge; TMD family can be written as MX_2_ with M being a transition metal and X = S,Se,Te; and phosphorus AF insulator 2D family formula is MPX_3_.

Such a vast number of atomically thin vdW magnets shows a wide variety of conduction and magnetic properties, ranging from FM semiconductors or metals to AF insulators. Due to their 2D character, they are much more sensitive to external stimuli allowing efficient control of their transport and magnetic properties. They can be naturally stacked together or with a wide range of vdW 2D or other 3D materials, forming heterostructures with properties induced *via* magnetic or spin–orbit proximity effects. Proximity can efficiently boost *T*_*C*_, alter interfacial spin polarization or introduce Rashba or Dzyaloshinskii–Moriya interactions. This implicates further exploration on spin–orbitronic and spin-caloritronic phenomena in nanostructures comprising vdW magnetic materials.

### Voltage Control of Magnetism in 2D Magnets

The control of magnetism in a material with an electric field is raising a wide interest because the absence of heating by currents favors energy efficient writing in magnetic-based nonvolatile memories. Layered magnets are very promising, since the atomically thick materials have potential to be more sensitive to electric field than common thin films, with the possibility to obtain almost ideal interfaces when stacking them with other vdW materials. The electrical control of magnetism in a 2D magnet can occur *via* different mechanisms, such as linear magnetoelectric coupling or electrostatic doping.

**Figure 39:**
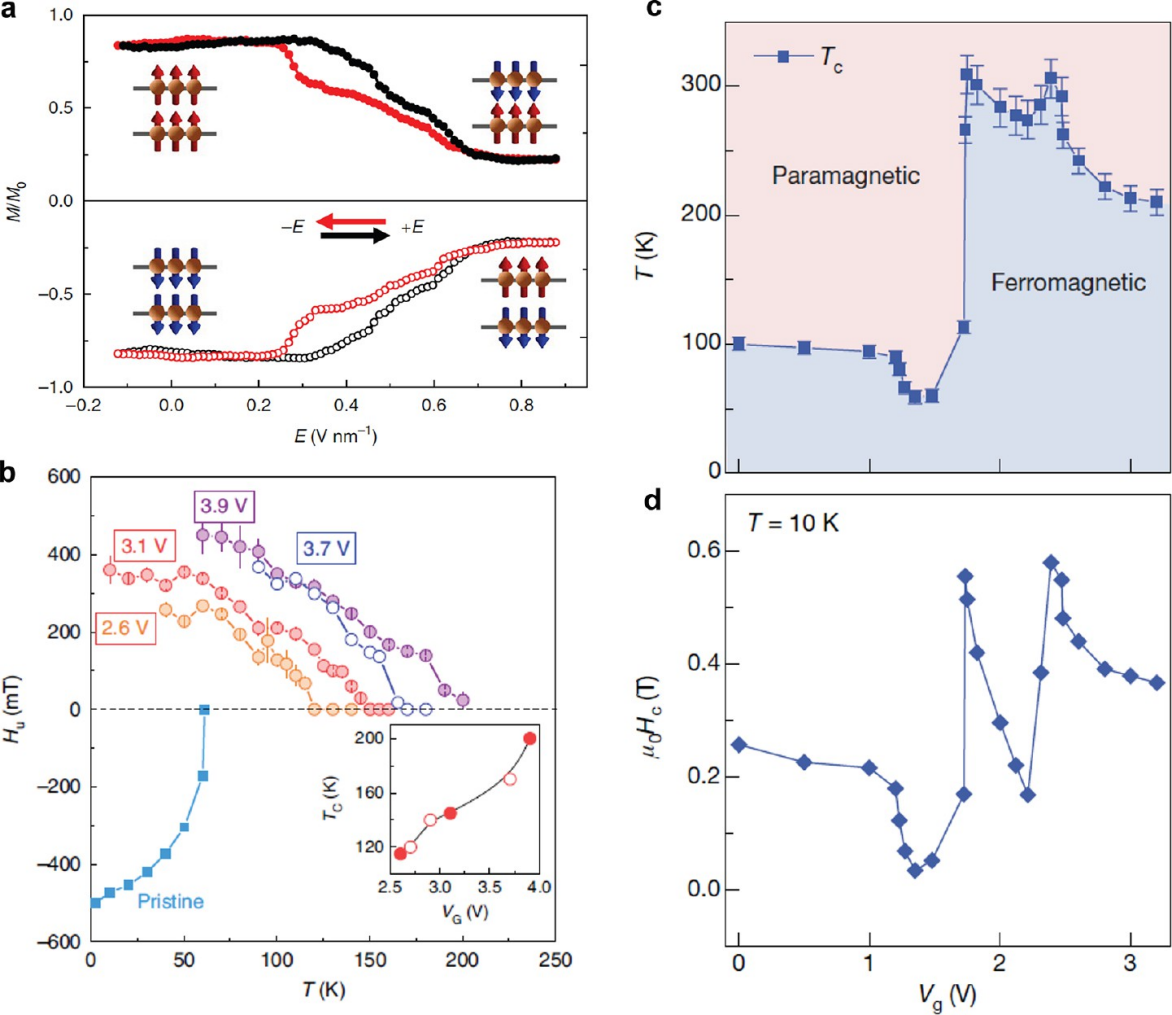
Voltage control of the magnetic properties of CrI_3_, CGT and FGT. (a) Top: Normalized magnetization measured by MCD as a function of the applied electric field (trace and retrace) at 4 K and fixed magnetic field (+0.44 T for top panel and −0.44 T for bottom panel), showing the electrical switching of the magnetic order in bilayer CrI_3_. The insets represent the corresponding magnetic states.^8^ Adapted with permission from ref (8). Copyright 2018 Springer Nature. (b) Uniaxial magnetic anisotropy field  of multilayer CGT as a function of temperature at different gate voltages and in the pristine case. Inset: The dependence of *T*_*C*_ on gate voltage.^86^ Adapted with permission from ref (86). Copyright 2020 Springer Nature. (c) *T*_*C*_ of a trilayer FGT as a function of gate voltage.^12^ (d) *H*_*C*_ of a trilayer FGT as a function of gate voltage at 10 K.^12^ Panels (c) and (d) are adapted with permission from ref (12). Copyright 2018 Springer Nature.

The former mechanism requires the material to break simultaneously time-reversal symmetry and inversion symmetry, a condition fulfilled by bilayer CrI_3_ in the AF ground state, but not by the FM phase or by the monolayer CrI_3_, in which inversion symmetry is present. Jiang *et al*.^8^ measured the magnetoelectric response with MCD and using a dual gate structure to apply an electric field in order to take out the effect of doping. Interestingly, the magnetoelectric coupling was maximum around the spin-flip transition that occurs at ∼0.5 T. This allowed the authors to switch electrically bilayer CrI_3_ between the AF and FM states at a constant magnetic field (close to the spin-flip transition, see Figure 39a).

The control of magnetism is also possible *via* electrostatic doping in 2D magnets. The advantage is that this mechanism does not require the specific symmetry of the linear magnetoelectric coupling and, besides bilayer CrI_3_,^9,11^ is also possible in monolayer CrI_3_^11^ and in CGT.^10,86^ In the case of monolayer CrI_3_,^11^ saturation magnetization (*M*_*S*_), coercive field (*H*_*C*_), and *T*_*C*_ increase (decrease) with hole (electron) doping. In bilayer CrI_3_, electron doping (∼2.5 × 10 ^13^ cm^–2^) reduces the spin flip transition almost to zero magnetic field.^11^ Although this should enable electrical switching of magnetization at zero field, a magnetic field close to the spin-flip transition still needs to be applied for fully reversible switches.^9,11^ Electrostatic doping using ionic liquid gating has also been reported in multilayer CGT.^10,86^ Wang *et al*.^10^ showed using MOKE measurements that saturation field (*H*_*S*_) decreases and *M*_*S*_ increases as a function of doping levels (both electron and hole), while *H*_*C*_ and *T*_*C*_ are insensitive to doping. This behavior is tentatively attributed to a moment rebalance of the spin-polarized band structure while tuning its Fermi level. In contrast, Verzhbitskiy *et al*.^86^ report an enhancement of *T*_*C*_ from ∼61 K to up to 200 K when an electron doping of ∼4 × 10^14^ cm^–2^ is applied, using magnetoresistance measurements. Interestingly, there is also a dramatic change in the magnetic anisotropy, which changes from perpendicular to in-plane (see Figure 39b). In this case, the effect is attributed to a double-exchange mechanism that is mediated by free carriers, which dominates over the superexchange mechanism of the original insulating state.

A different effect, reported in multilayer CrI_3_, is memristive switching at certain applied voltage, where the two resistive states are coupled to the magnetic phases.^438^ The mechanism here is thermally induced when current flows across CrI_3_.

Voltage control of magnetism has also been reported in FGT which, unlike the previous 2D magnets mentioned in this subsection, is metallic. Deng *et al*.^12^ use ionic gating to bring *T*_*C*_ from ∼100 K up to ∼300 K in trilayer FGT (see Figure 39c), an important result since no pristine 2D magnet is FM at room temperature. *H*_*C*_ roughly follows the variations in *T*_*C*_, as shown in Figure 39d. The large electron doping induced by the ionic gate (∼10^14^ cm^–2^ per layer) causes a substantial shift of the electronic bands of FGT. The large variation in the DOS at the Fermi level leads to appreciable modulation in the ferromagnetism, in agreement with the Stoner model for itinerant electrons.^12,439^ Finally, metallic ferromagnet Fe_5_GeTe_2_ has been electron doped with protonic gating, which can induce a transition to an AF phase at 2 K.^440^ Recently, room temperature ferromagnetism has been observed in MBE grown Fe_5_GeTe_2_ 2D films.^339^

### Manipulation of the Magnetization of 2D Magnets by Current-Induced Spin–Orbit Torque

**Figure 40:**
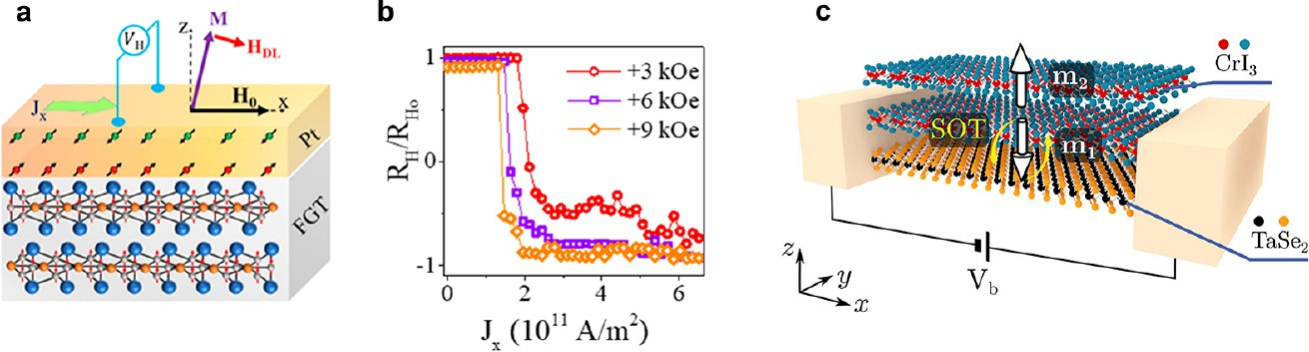
Current-induced magnetization switching of a FGT 2D magnet. (a) Schematic illustration of the current-induced switching of a FGT nanoflake by the spin current generated by SHE in the Pt layer deposited on FGT and injected into FGT to produce a SOT.^442^*J*_*x*_ is the current in Pt generating a downward spin current by SHE, *H*_0_ is the in-plane field tilting the magnetization *M* from its out-of-plane orientation at zero field, *H*_*DL*_ is the damping-like (DL) component of the effective field expressing the action of the spin transfer torque. (b) Current-induced switching detected by the change of sign of the transverse voltage *V*_*H*_ in panel (a) (*R*_*H*_ ∼ *V*_*H*_/*J*_*x*_).^442^ Panels (a) and (b) are adapted with permission from ref (442). Copyright 2019 American Chemical Society. (c) Schematic view of the CrI_3_/TaSe_2_ vdW heterostructure consisting of an insulating AF bilayer of CrI_3_ and a nonmagnetic metallic monolayer TMD TaSe_2_. The SOT on the magnetization of CrI_3_ is due to the charge-to-spin Edelstein conversion of the current flowing along the CrI_3_/TaSe_2_ interface. The resulting switching of **m**_1_ is indicated by arrows.^444^ Adapted with permission from ref (444). Copyright 2020 American Chemical Society.

The magnetization of 2D magnets can be manipulated by spin–orbit torques (SOTs) induced by spin-transfer from spin currents generated from charge currents by spin–orbit couplings (SOCs). The conversion of charge current into spin current can be obtained by spin Hall effect (SHE) in 3D materials of large SOC (*e.g.*, heavy metals such as Pt) or by the Edelstein effect in 2D electron gas (2DEG) such as Rashba interface states or surface states of topological insulators.^441^ In the FGT/Pt bilayer of Figure 40a, a horizontal charge current in Pt is converted by SHE into a vertical spin current which is injected into FGT and generates the SOT on the FGT magnetization.^442^ The switching of the out-of-plane magnetization of FGT flake is detected by the transverse voltage induced by the anomalous Hall effect (AHE) in FGT, as shown in Figure 40b.^442^ This switching is observed only if an in-plane magnetic field (*H*_0_ in Figure 40a, 3, 6, or 9 kOe in Figure 40b) is also applied in the current direction, which is the usual symmetry breaking condition required in the experiments of magnetic switching in layers of 3D FM materials with out-of-plane magnetization.^441^ A similar experiment has been reported by Wang *et al*.^443^

Interestingly, similar experiments of switching by current-induced SOT have been performed with nonconducting 2D magnets, for example on bilayers of CGT and Pt or Ta.^445−447^ The SOT is detected by the Hall voltage ascribed to the introduction of spin polarization and AHE in Pt (Ta) by proximity with CGT. An alternative explanation is by the spin Hall magnetoresistance (SMR) associated with SOT, that is the dependence of the resistance of Pt (Ta) on the relative directions of the current and magnetization of CGT.^445,447^ SMR effects have been clearly observed in bilayers of the insulating 2D magnet Co-doped MoS_2_ and Ta.^448^

As mentioned above, the spin currents inducing SOT can be generated not only by the SHE of a 3D material as Pt but also from SOC effects in 2DEGs at interfaces or surfaces. An interesting example is given by the theory by Dolui *et al*.^444^ of the SOT acting on CrI_3_ in the bilayer CrI_3_/monolayer TaSe_2_ heterostructure shown in Figure 40c. A CrI_3_ bilayer is an antiferromagnet with opposite magnetizations of the two layers. The proximity of the bottom CrI_3_ layer with TaSe_2_ introduces interface states of large SOC and the current flowing in these states is converted by the Edelstein effect^441^ into a spin accumulation that is injected into the bottom CrI_3_ later and generates a SOT on its magnetization **m**_1_. An interesting result is that the SOT generated by current pulses in TaSe_2_ can reverse **m**_1_ and convert the CrI_3_ bilayer from antiferromagnet to ferromagnet with parallel magnetizations of the two layers. According to Dolui *et al*.,^444^ the transition should induce a change of resistance of 240% for a tunnel junction composed of a bilayer-CrI_3_/monolayer-TaSe_2_ between graphite/hBN electrodes.

Another example of interfacial SOT with 2D magnets is given by the SOT results on bilayers of the 3D ferromagnet NiFe and the 2D AF insulator NiPS_3_.^449^ As the large SOTs acting on NiFe include components of the different “damping-like” and “field-like” symmetries, they can be ascribed to interfacial SOT. The SOT increases below the Néel temperature of NiPS_3_ (170 K), pointing out a possible relation with magnetic ordering.

### Proximity Effects

**Figure 41:**
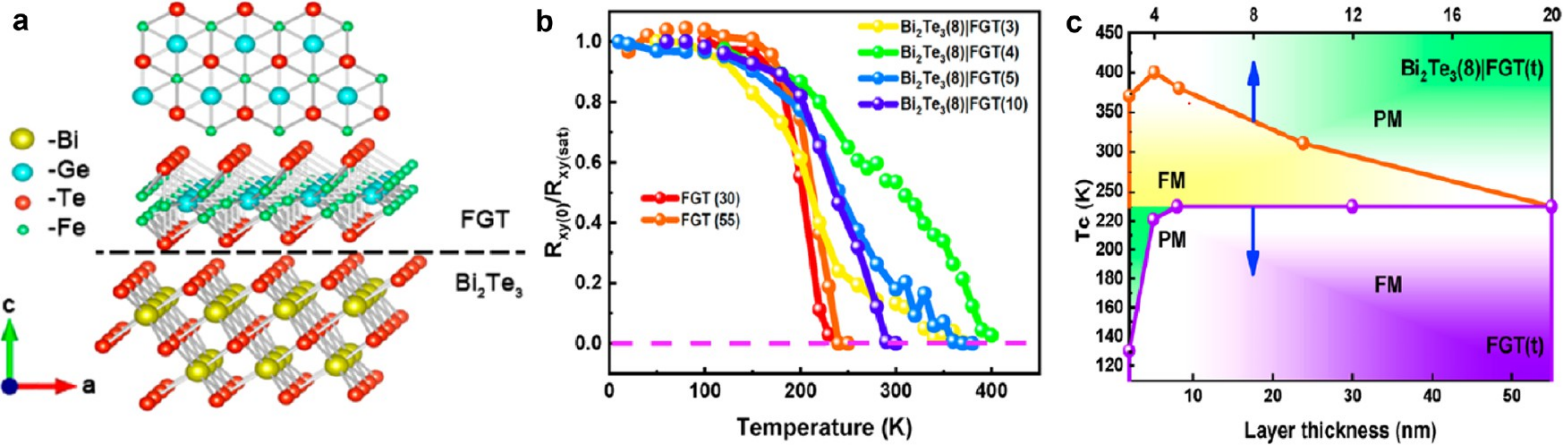
Structure and magnetic properties of Bi_2_Te_3_/FGT heterostructures. (a) Structure of Bi_2_Te_3_/FGT. (b) Anomalous Hall resistance *R*_*xy*_ as a function of temperature for FGT and Bi_2_Te_3_/FGT heterostructures. (c) Magnetic phase diagram of pure FGT and Bi_2_Te_3_/FGT heterostructures versus FGT layer thickness and temperature (FM for ferromagnetic, PM for paramagnetic). All panels are adapted with permission from ref (90). Copyright 2020 American Chemical Society.

Atomically thin materials are expected to be very sensitive not only to electric fields but also to proximity effects. We first consider the proximity effects between 2D magnets and 3D materials and their potential to change the properties of the 2D magnets. A highly interesting example is the large increase of *T*_*C*_ of the 2D magnet FGT when it is grown on the topological insulator (TI) Bi_2_Te_3_.^90^ The bilayer Bi_2_Te_3_/FGT is grown by MBE and its structure is displayed in Figure 41a. The magnetic properties are characterized by the AHE associated with the ferromagnetism of FGT. The plot of the AHE resistance as a function of temperature shown in Figure 41b demonstrates that *T*_*C*_ of FGT is enhanced from about 230 K for pure FGT to 400 K in a Bi_2_Te_3_ (8 nm)/FGT(4 nm) heterostructure. *T*_*C*_ decreases at increasing thickness of FGT but is still at about 300 K for Bi_2_Te_3_(8 nm)/FGT(10 nm).

A precise determination of *T*_*C*_ in Bi_2_Te_3_(8 nm)/FGT(4 nm) heterostructures can be achieved by the classical Arrott plot and leads to the magnetic phase diagram of Bi_2_Te_3_/FGT *versus* layer thickness and temperature in Figure 41c. The decrease of *T*_*C*_ with increasing FGT thickness is in favor of the general idea of interfacial effect. However, the role of the large SOC and spin-momentum locking in the surface states of TI is not well understood yet. It was suggested that the large SOC at the interface with Bi_2_Te_3_ could enhance the intralayer interactions in FGT.^90^ Anyhow, as it is extremely challenging to achieve room temperature 2D ferromagnets, the type of result described in the preceding lines is very promising for the future of the 2D magnets.

Proximity effects can also be used to introduce additional properties in vdW heterostructures involving 2D magnets. An example is the generation of magnetic skyrmions in FGT by the DMI introduced by an interface between FGT and the TMD WTe_2_.^27^ Another example is the introduction of magnetic proximity effect in graphene in combination with a 2D magnet. Karpiak *et al*.^450^ used ferromagnetic CGT on top of graphene and obtained an exchange field of few tens of mT using Hanle precession during spin transport, smaller than the one obtained by Tang et al.^451^ using CrBr_3_ and Zeeman spin Hall measurements (few T). Ghiasi *et al*.^452^ proximitized graphene with AF CrSBr. With this combination they obtained a conductivity spin polarization of 14%, corresponding to a much larger exchange field in graphene (∼170 T). These proximity-induced exchange fields are comparable with those obtained using magnetic insulators such as yttrium iron garnet (YIG)^425,453,454^ or bismuth ferrite (BFO)^455^ even though lower than theoretical predictions.^456−459^ This is an important addition to graphene functionalities for spintronics,^406,407^ besides long-distance spin transport^460^ and spin–orbit proximity effects.^461^ Beyond graphene, we refer to the examples of the proximity of CrI_3_ in inducing a zero-field Zeeman splitting in the valley states of WSe_2_.^206,462^

The properties of 3D materials can also be changed by proximity with a 2D material. We referred previously to the AHE or SMR effects induced in a Pt layer by proximity with the 2D magnet CGT and their use to detect the magnetic state of CGT.^445−447^ A proximity magnetoresistance in Pt has also been reported in combination with CrI_3_.^463^ In a similar way, the interfacial hybridization of magnetic Ni with graphene changes the effective spin polarization of its density of states, what has been used to obtain very large tunnel magnetoresistances.^464^ Similarly, proximity effects in Co/WSe_2_ bilayers change the sign and magnitude of the effective spin polarization of Co.^465^ The interfacial hybridization between Co and graphene orbitals can also explain strong enhancement of perpendicular magnetic anisotropy in Co films.^466^

### Spin Seebeck Effect and Magnon Transport

**Figure 42:**
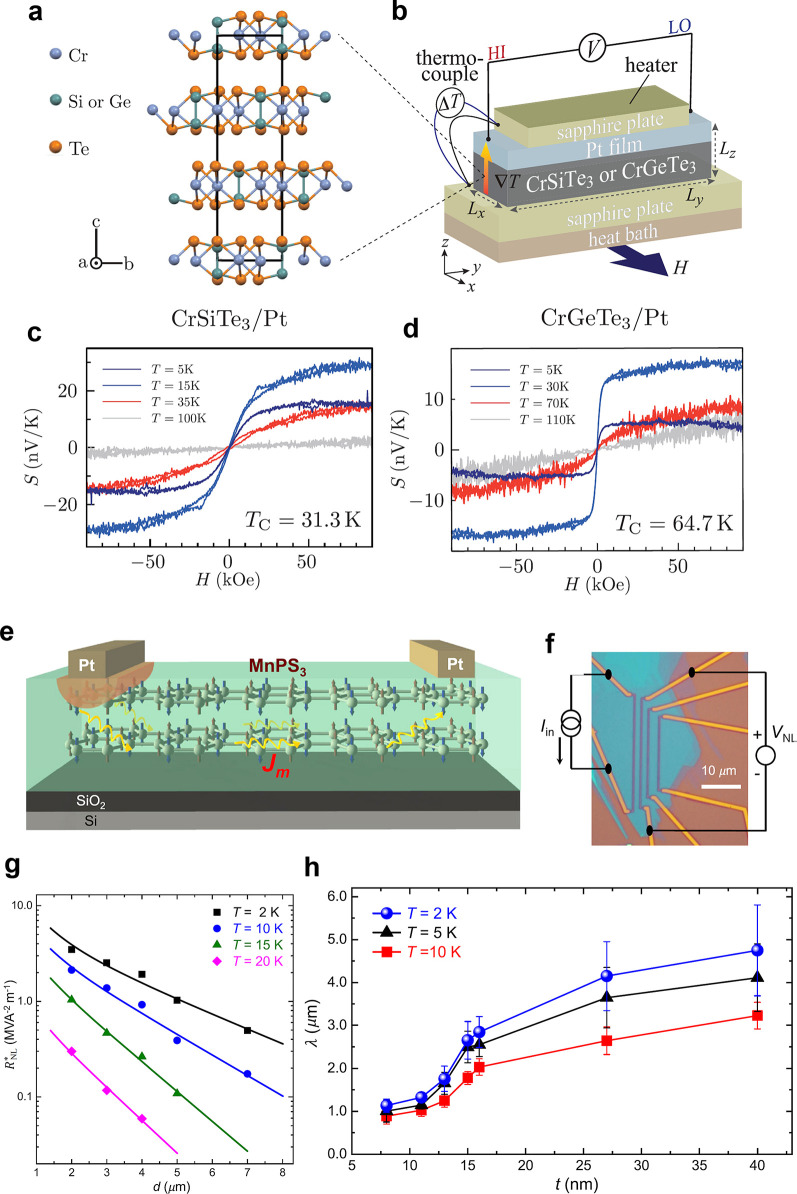
Spin Seebeck effect and magnon transport with 2D magnets. (a) Crystal structure of CGT and CST.^467^ (b) Schematic of the longitudinal SSE measurements in CST/Pt or CGT/Pt bilayers. *H* denotes the external magnetic field and Δ*T* (∇*T*) the temperature difference (gradient).^467^ (c, d) Normalized SSE voltage *S* = (*V*/∇*T*) (*L*_*z*_/*L*_*y*_) as a function of *H* in the (c) CST/Pt and (d) CGT/Pt bilayers at selected temperatures.^467^ Panels (a–d) are adapted with permission from ref (467). Copyright 2019 American Physical Society. (e) Schematic of the magnon generation, transport, and detection in MnPS_3_.^468^ (f) Optical image of the device with the MnPS_3_ flake and Pt electrodes, including the measurement configuration of the nonlocal SSE.^468^ (g) Normalized nonlocal signal  as a function of distance (*d*) for selected temperatures in a 16 nm-thick MnPS_3_ flake. The solid lines represent the best-fitting results based on a diffusion equation.^468^ (h) Magnon diffusion length as a function of MnPS_3_ thickness (*t*) for selected temperatures.^468^ Panels (e–h) are adapted with permission under a Creative Commons CC BY 4.0 license from ref (468). Copyright 2019 American Physical Society.

The spin Seebeck effect (SSE) is the conversion of a temperature gradient into a voltage, mediated by spin currents, occurring in simple magnetic material/paramagnetic metal bilayers,^469^ with a potential to be used as thermoelectric conversion elements.^470^ In a structure similar to that of Figure 42b, a temperature gradient in the magnetic material along *z* creates a vertical magnon current that leads to magnon imbalance at the top interface. The angular momentum of the magnons (along *x*, the magnetization direction determined by the applied magnetic field *H*) is transferred *via* exchange coupling to the conduction electrons of the paramagnetic metal, generating a spin accumulation. This spin accumulation diffuses into the paramagnetic metal as a spin current along *z*, which is converted into a charge current along *y via* the inverse SHE.

The SSE has been recently observed in 2D magnets. Ito *et al*.^467^ report SSE in FM insulators CGT and CST (CrSiTe_3_) covered by Pt (Figure 42a,b). In contrast to prototypical YIG, the SSE response persists above the critical temperatures in both 2D magnets (Figure 42c,d), which is attributed to exchange-dominated interlayer transport of in-plane paramagnetic moments reinforced by short-range FM correlations and strong Zeeman effects.

Furthermore, the SSE has been used to inject magnon currents in order to study magnon transport. By using a lateral structure, the magnon transport have been quantified in 3D insulating magnets such as ferrimagnetic YIG^471,472^ or AF α-Fe_2_O_3_.^473^ Xing *et al*.^468^ used such a nonlocal SSE to demonstrate magnon transport in 2D antiferromagnet MnPS_3_ (Figure 42e,f). Whereas the current injected in a Pt wire induces the temperature gradient to generate the magnon accumulation *via* the SSE, a second Pt wire detects the diffusing magnons as a voltage *via* the inverse SHE. The decay of this voltage with the distance (*d*) between Pt wires (Figure 42g) is used to extract the magnon diffusion length of MnPS_3_ (Figure 42h), which is of the order of the best 3D magnets^471−473^ and thus promising for magnonics. With the same approach, magnon transport induced by SSE has been reported in 2D FM insulator CrBr_3_ by Liu *et al.*(474)

### Chiral Magnetic Structures: Skyrmions

**Figure 43:**
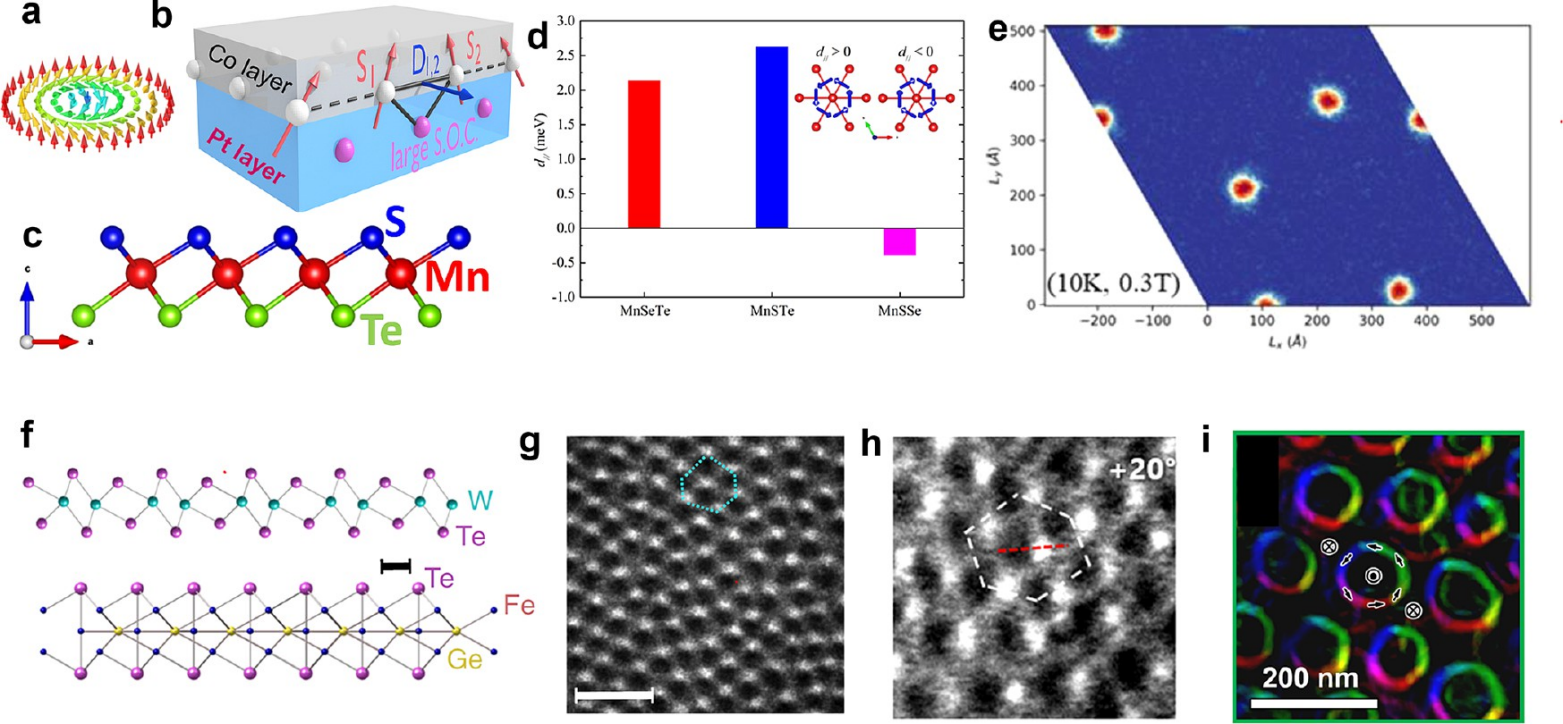
Skyrmions in 2D magnets. (a) Spin structure of a Néel skyrmion. (b) Pt/Co interface in which the absence of inversion symmetry generates DMI, *H*_*DMI*_ = −(**S**_1_ × **S**_2_)·**D**_12_. (c) Side view of the crystal structure of the Janus TMD MnSTe in which the absence of inversion symmetry generates DMI.^480^ (d) DMI strength calculated for the Janus TMD MnSeTe, MnSTe, and MnSSe.^480^ (e) Skyrmions in MnSeTe (*T* = 10 K in applied field of 0.3 T) from DFT calculation of DMI and Monte Carlo simulations.^480^ Panels (c–e) are adapted with permission from ref (480). Copyright 2020 American Physical Society. (f) Side view of the crystal structure of WTe_2_ on FGT.^27^ (g) LTEM image of Néel skyrmion lattice at 180 K under 510 Oe in the sample 2L WTe_2_/40L FGT (L = layer) at tilt angle 30° and under focus. Scale bar: 500 nm.^27^ Panels (f) and (g) are adapted with permission under a Creative Commons CC BY license from ref (27). Copyright 2020 Springer Nature. (h) TEM image of Néel skyrmion lattice in an oxidized FGT flake (about 50 μm thick) at 160 K, tilt angle 20° and over focus.^26^ Adapted with permission from ref (26). Copyright 2021 American Physical Society. (i) Magnetization maps derived from analysis of LTEM image for Bloch bubbles in a CGT flake at 17 K in a field of 11.7 mT.^481^ Adapted with permission from ref (481). Copyright 2019 American Chemical Society.

The past decade has seen a substantial development of the research on chiral spin structures such as magnetic skyrmions or chiral domain walls. As represented in Figure 43a, skyrmions are small local whirls of the magnetization with a topology induced by chiral interactions between spins.^475,476^ These topological spin textures behave as nanoparticles that can be manipulated by electrical currents, which makes them suitable for important applications in information technologies. The skyrmions, in most cases, are induced by DMI.^250,251^ The DMI between spins **S**_1_ and **S**_2_ is of the form:
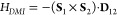
13where **D**_12_ indicates the Dzyaloshinskii–Moriya vector. The DMIs are induced by SOC in systems without inversion symmetry, either in noncentrosymmetric lattices or when the inversion symmetry is broken by the presence of an interface. In recent years, most studies on chiral domain walls and skyrmions have been performed in systems with interface-induced DMI,^477,478^ as illustrated, for instance, in Figure 43b by a Pt/Co bilayer in which Pt brings its large SOC, while the inversion symmetry is broken by the presence of an interface. Large DMI can also be obtained in Co layers by their interface with graphene.^479^

For 2D magnets, the simplest situation for chiral magnetism is a structure with inherent inversion asymmetry and intrinsic DMI. The opportunity of this situation is given by the so-called Janus TMDs, which can be synthesized by controlling the reaction conditions.^482−484^ An example of Janus crystal structure is that of MnSTe shown in Figure 43c. First principles calculations based on density functional theory (DFT) have shown that single layers of the Janus TMD MnSeTe, MnSTe, and MnSSe are FM with Curie temperatures between 140 and 190 K, out of plane magnetizations and the DMI energies presented in Figure 43d.^480^ These DMIs are comparable to those generated by Pt/Co interface (Figure 43b) and other interfaces, which are routinely used for the generation of skyrmions.^480,485^ Monte Carlo simulations using the calculated exchange and DMI parameters find that the FM ground state of MnSeTe and MnSTe at zero field develops skyrmions by applying a magnetic field,^480^ see Figure 43e. The possibility of inducing skyrmions in Janus chromium chalcogenides,^486^ trihalides^487^ and in 2D multiferroics^488^ has been also recently reported.

In the general situation of 2D magnets with centrosymmetric structures, DMIs can be introduced by breaking their inversion symmetry by interfaces between different 2D materials. An example of interface induced DMI is the bilayer of Figure 43f in which the TMD WTe_2_ is deposited on a FGT layer. The LTEM images at tilt angle in Figure 43g, with black and with half-moons, are typical of Néel skyrmions, which have been ascribed to DMIs at the WTe_2_/FGT interface.^27^ However, DMIs at the single WTe_2_/FGT top interface are not expected to generate a skyrmionic texture extending to the bottom of the stack of 30 FGT monolayers. The authors have suggested a skyrmionic texture extending to only a certain depth, but its exact profile has not been determined.^27^ Néel skyrmions have been also clearly identified in LTEM images of similar FGT layers,^26^ as shown in Figure 43h. These skyrmions are ascribed to DMIs at the interface between top and bottom oxidized FGT layers and the nonoxidized central FGT. This interpretation is consistent with the absence of Néel skyrmions replaced by magnetic bubbles of Bloch type in FGT samples without oxidation.^25,26^ The pending question for the Néel skyrmions in the oxidized samples is again the exact profile of the skyrmions as a function of the depth in the 50-μm-thick FGT. Nevertheless, the promising result with the skyrmions in FGT of Figure 43h is that they can be moved by current pulses as classical Néel skyrmions in 3D materials.^26^

In the absence of DMI, 2D magnets with out-of-plane magnetization generally present spin textures of the magnetic bubble type, bubbles of reversed magnetization surrounded by a Bloch domain wall, as shown in Figure 43i for CGT^481^ and already mentioned above for nonoxidized FGT.^26^ The possibility of inducing skyrmions from mechanisms other than DMI such as dipole–dipole interactions in FGT on Co/Pd superlattices has also been reported.^489^ In addition, the skyrmion formation in 2D magnets using Moiré patterns in vdW heterostructures was also proposed.^490^

### Spintronic Devices

2D magnets can be integrated into more complex structures to create spintronic devices that could present interesting advantages for rapidly emerging technologies. An example of such advantages is the possibility of obtaining almost ideal interfaces when stacking them with other vdW materials. The voltage control of magnetism present in atomically thin materials allows, in some cases, for extra functionalities beyond the classical spintronic devices.

**Figure 44:**
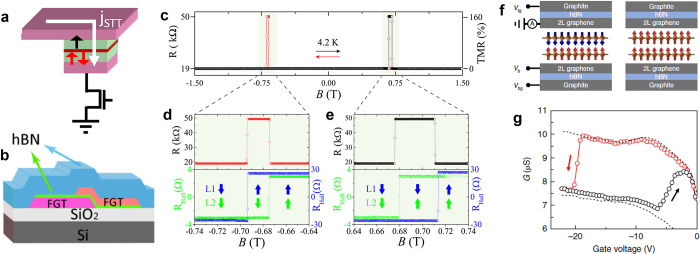
Toward MRAM with 2D magnets. (a) Schematic of STT-MRAM with 3D materials:^491^ information coded by the relative orientations of the magnetization of the two magnetic layers (green) separated by an insulating MgO layer (red), writing by current-induced STT and reading by TMR. Adapted with permission from ref (491). Copyright 2017 American Physical Society. (b) Schematic of the TMR device based on a FGT/hBN/FGT vertical stack used for the results shown in panels (c–e).^109^ (c) TMR measurement in a FGT/hBN/FGT vertical stack at 4.2 K. The swapped magnetic field is out of plane.^109^ (d, e) Magnified regions of the TMR measurement around the field range of antiparallel configuration (upper panels) and variation of the AHE resistance of the top (blue) and bottom (green) FGT electrodes in the same field range. Blue and green arrows indicate the successive orientations of the magnetizations.^109^ Panels (b–e) are adapted with permission from ref (109). Copyright 2018 American Chemical Society. (f) Schematic of the device combining a gate-controlled spin-flip transition in bilayer CrI_3_ and spin filtering in the tunnel junction. Arrows indicate the magnetic orientation of each layer.^492^ (g) Tunnel conductance of the device illustrated in panel **f** as a function of gate voltage (sweeping back and forth) under a constant magnetic field (0.76 T). The measured tunnel conductance changes when the magnetic order of bilayer CrI_3_ is switched by the gate.^492^ Panels (f) and (g) are adapted with permission from ref (492). Copyright 2019 Springer Nature.

The prototypical spintronic device is the spin valve, which consists of two different magnetic conducting layers sandwiching a nonmagnetic layer. The latter can be either a conductor or an insulator, giving rise to giant (GMR)^101,102^ or tunneling magnetoresistance (TMR),^493,494^ respectively, caused by the spin-dependent transport across the device. Since the 90s, the GMR has boosted the technologies of information storage, for example in the read heads of the hard disk drives.^495^ The TMR of MTJs is exploited today in nonvolatile magnetic random access memories (MRAM) (Figure 44a), and recent advances have been achieved in STT-MRAMs using magnetic layers with out-of-plane magnetization and spin-transfer torque (STT) for electrical switching.^491,496^ The advantages of the STT-MRAMs are nonvolatility, superior scaling properties, speed, and low energy consumption. They are presently commercialized as a replacement for SRAMs and eFlash in embedded cache memories.

MRAM technology could benefit from 2D magnets that present large magnetic anisotropy in atomically thick layers and can also be integrated with vdW heterostructures for a great variety of devices. An MTJ using 2D magnets was achieved by stacking two flakes of FM layered dichalcogenide Fe_0.25_TaS_2_, where native Ta_2_O_5_ oxide layer works as the spacing layer, reaching above 6% TMR ratios.^497^ Similar devices, replacing one of the magnetic layers with Cr_0.33_TaS_2_, yield up to 15% TMR.^498^ Taking advantage of vdW stacking, Wang *et al*.^109^ used atomically thin hBN as an insulating layer between two FGT flakes (Figure 44b), where a TMR up to 160% is observed (Figure 44c–e). This allowed the authors to determine a spin polarization of 0.66 for the density of states in FGT.^109^ Theoretical calculations predict that TMR ratios could exceed thousands of percent in such heterostructures.^499^ By replacing hBN with graphite, the observation of three resistance states is reported and attributed to spin-momentum locking at the FGT/graphite interface caused by the strong SOC in FGT.^500^ Furthermore, recent theoretical studies pointed out toward realization of four resistance states in vdW multiferroic tunnel junctions comprising FGT layers separated by 2D ferroelectric In_2_Se_3_ barrier layers.^501^ TMR up to 3.1% has been reported when using semiconducting MoS_2_ as a spacer, acting as a conductor rather than a tunnel barrier.^115^ The prediction of FeCl_2_, FeBr_2_, and FeI_2_ as half metals suggests these materials could further improve the figures for TMR.^502^ Beyond the standard spin valves, devices where the two magnetic layers do not conduct but spin-polarize the electrons of the nonmagnetic spacer by strong proximity effects have been theoretically proposed by Cardoso *et al*.,^503^ who model a bilayer graphene sandwiched by two CrI_3_ monolayers. A band gap opens at the Dirac point of graphene in the antiparallel configuration, whereas in the parallel configuration, the graphene bilayer remains conducting.

Large magnetoresistances are also achievable in devices where the magnetic and nonmagnetic layers are swapped, and two nonmagnetic conductors sandwich a magnetic insulator that acts as a spin filter. Altering the magnetic state of such spacer *via* applied magnetic field could lead to the large difference in the spin-dependent tunneling giving rise to enormous TMR values (see Spin Filtering Effect section for details). Taking advantage of the voltage control of magnetism present in these atomically thick materials, Song *et al*.^504^ and Jiang *et al*.^492^ combine the spin filtering in a graphene/CrI_3_/graphene heterostructure for reading the magnetic order of CrI_3_ with the electrical switching (“writing”) of such magnetic order *via* spin-flip transition (see Figure 44f). This device shows nonvolatility and a large conductance change between the different magnetic orders (Figure 44g), which could be an alternative in MRAM applications. For another CrI_3_ heterostructure, we have already mentioned a change of conductance induced by a spin-flip transition of a CrI_3_ bilayer.^444^

STT-MRAMs are expected to be ultimately limited in speed because of the relatively large switching latency of STT and high currents required to reach sub-ns switching times, which can damage the MTJ tunnel barrier. One of the most solid alternatives is the SOT-MRAM in which the magnetic state of an MTJ is switched by the SOT induced by the spin current generated by the Edelstein effect^505^ and/or the SHE in a material with large SOC.^506−508^ They offer unmatched switching speed and endurance compared to STT-MRAM. We present below the perspective with 2D magnet-based SOT-RAMs.

**Figure 45:**
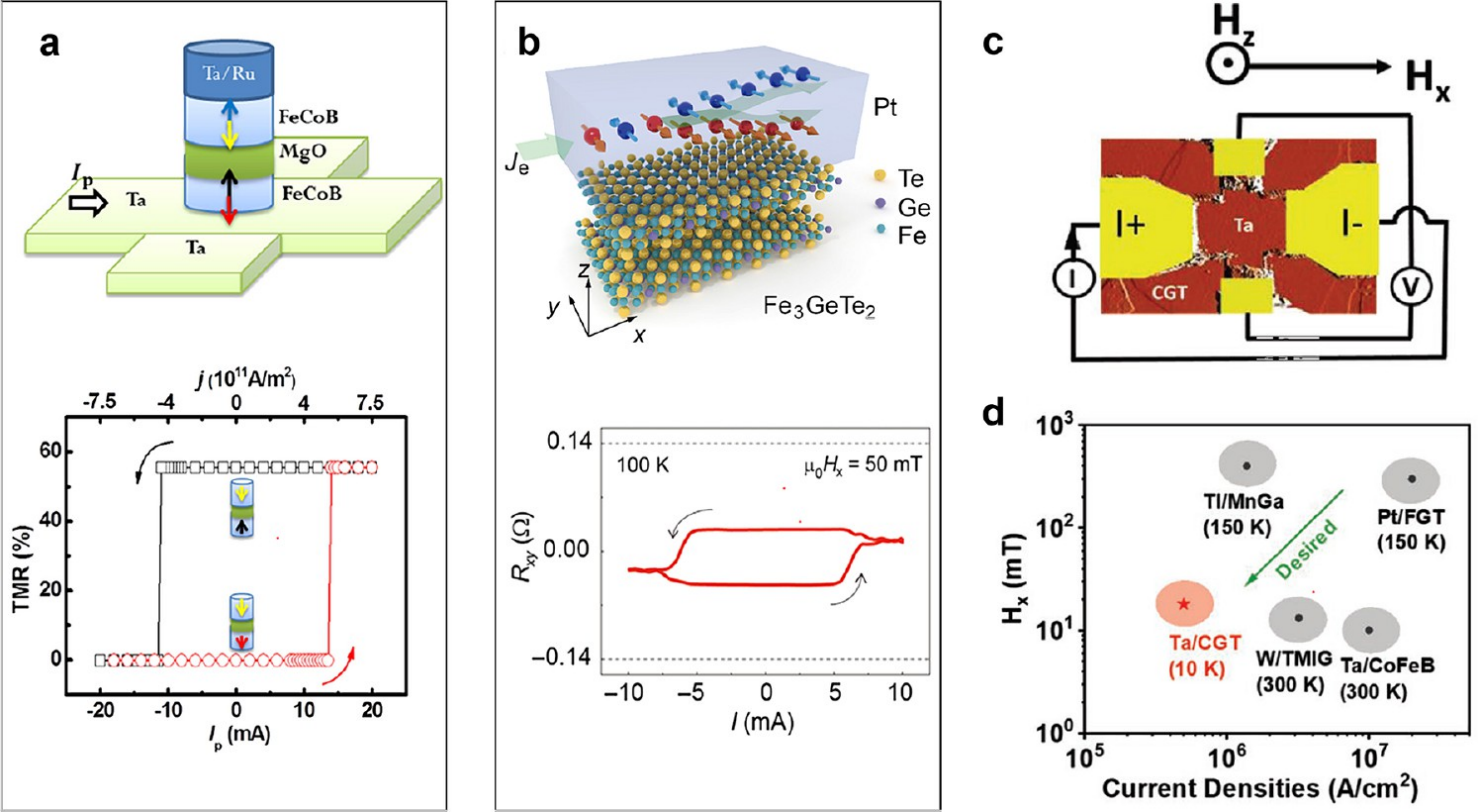
2D magnet-based SOT-MRAMs. (a) Top: Schematic of a SOT-MRAM based on 3D materials^507^ in which an electrical current in the heavy metal (Ta) of the bottom electrode generates by SHE the vertical spin current injected in the bottom FeCoB layer. This injection of spin current switches the magnetization of FeCoB by SOT (writing). The state of the memory is detected by the TMR of the FeCoB/MgO/FeCoB MTJ (reading). Bottom: Detection by TMR of the SOT-induced switching of the magnetization of the bottom FeCoB layer in the device of the schematic.^507^ Adapted with permission from ref (507). Copyright 2014 AIP Publishing. (b) Top: Schematic of a bilayer for SOT-MRAM in which the orientation of the out-of-plane magnetization of a FGT layer codes the information and is switched by the SOT generated by the SHE of the Pt layer.^443^ As shown in the bottom part of the figure, the switching is detected by the AHE resistance *R*_*xy*_ derived from the voltage between transverse contacts. Adapted with permission under a Creative Commons CC BY-NC 4.0 license from ref (443). Copyright 2019 AAAS. (c) Image of a heterostructure for SOT-MRAM in which the magnetic state of a CGT layer can be switched by the SHE of a Ta layer.^446^ (d) Comparison of the current densities and in-plane fields required for SOT switching in devices based on 3D magnetic layers (CoFeB, MnGa, thulium iron garnet (TmIG)) and 2D magnets (FGT, CGT). The best results so far are for Ta/CGT.^446^ Panels (c) and (d) are adapted with permission from ref (446). Copyright 2020 John Wiley and Sons.

In the schematic of SOT-MRAM with 3D materials in Figure 45a, a horizontal current flowing in the Ta layer generates *via* SHE a vertical spin current injected into the bottom FeCoB layer of the MTJ to switch its magnetization by SOT.^507^Figure 45a also shows an example of the TMR signal reflecting the current-induced back and forth switching of the FeCoB/MgO/FeCoB MTJ.^507^ One can see a similar switching of the magnetization of a FGT layer by SOT for the device in Figure 45b reported by Wang *et al*.^443^ Here the SHE in the top platinum layer generates the vertical spin current injected into the FGT to reverse its magnetization that is detected by AHE resistance of FGT. The figure displays an example of back and forth switching of the magnetization of FGT similar to those in Figure 45a for FeCoB (in both cases, the switching requires an applied magnetic field along the current direction). The schematic in Figure 45c represents another example of device harnessing the SHE of Ta to switch the magnetization of the 2D magnet CGT.^446^ Actually, the performance of such SOT devices can be characterized by their requirement of small current density and small applied fields. Figure 45d compares the current densities and in-plane fields required for SOT switching in devices based on 3D magnetic layers (CoFeB, MnGa, TMIG) or 2D magnets (FGT, CGT). The comparison is at the advantage of 2D magnets, in particular Ta/CGT. However, the obvious disadvantage of 2D magnets is the low temperature that is required. They would be very promising for applications if their *T*_*C*_ can be raised above room temperature, as it has been already achieved for FGT grown on Bi_2_Te_3_^90^ or with electrostatic doping.^12^

**Figure 46:**
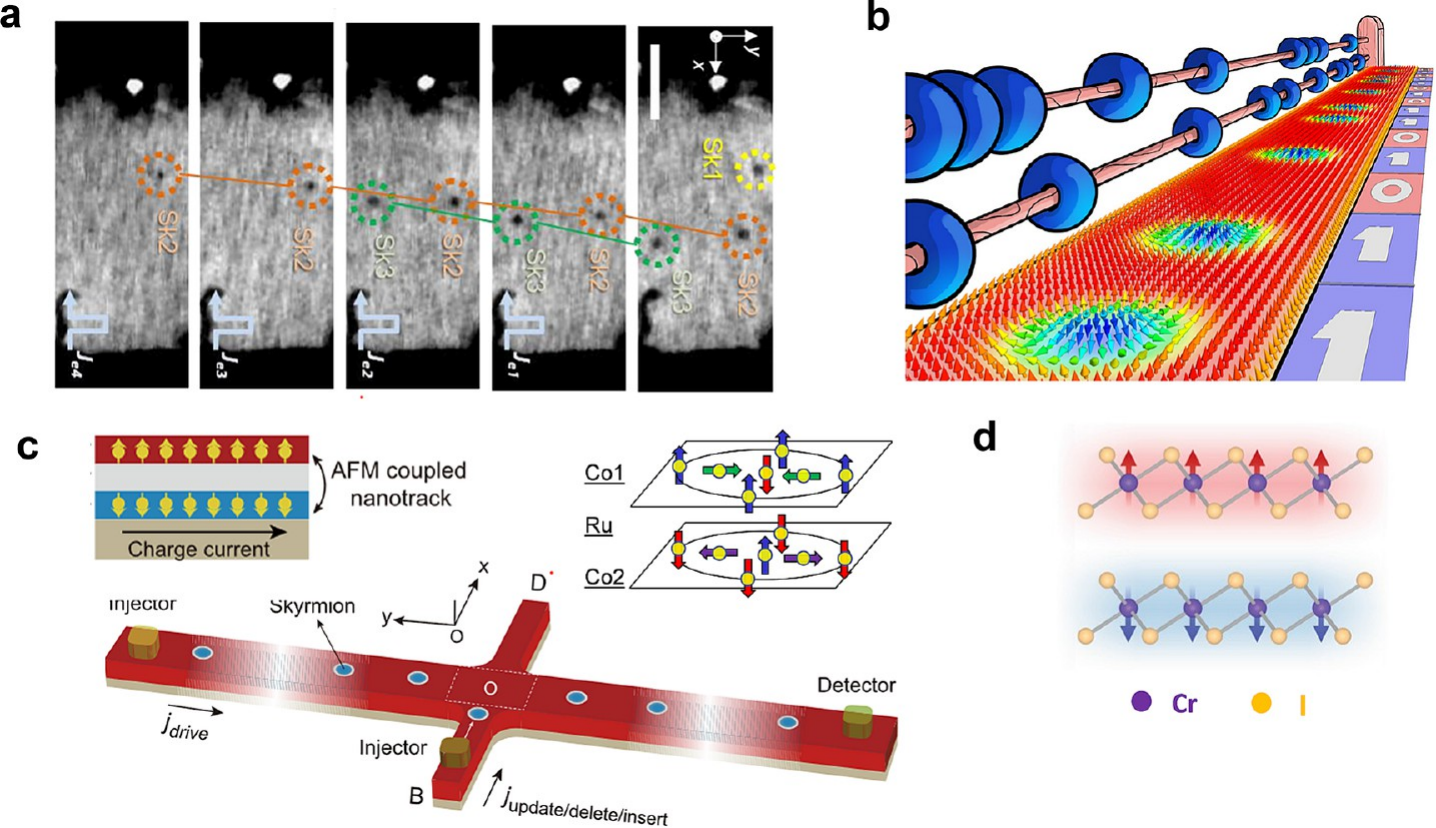
Devices based on current-induced motion of skyrmions in 2D magnets. (a) Skyrmion motion induced by current pulses in a FGT track.^26^ Each STXM image is acquired after injecting five unipolar current pulses of 50 ns. Two individual Néel skyrmions are outlined in colored circled for clarity. The diameter of the skyrmions is about 200 nm, their velocity is around 1 m/s for a current density of 1.4 × 10^11^ A/m^2^ and the width of the track is 50 μm.^26^ Adapted with permission from ref (26). Copyright 2021 American Physical Society. (b) Schematic of racetrack memory storing data by aligning skyrmions like beads on an abacus and displacing them by current-induced SOT from write head to read head.^476^ Adapted with permission from ref (476). Copyright 2018 AIP Publishing. (c) Proposal of skyrmion-based racetrack memory based on the SOT-induced motion of antiferromagnetically-coupled skyrmions in two layers coupled by AF interactions.^513^ The left inset is a schematic of an antiferromagnetic (AFM or AF)-coupled nanotrack and the right inset represents AF-coupled skyrmions in Co/Ru/Co trilayers.^512^ As the AF-coupled skyrmions have the same chirality but opposite polarities, their motion has the advantage of being along the current direction (no Skyrmion Hall effect^509,512,513^). Note that the racetrack memory of the schematic includes not only injector and detector but also an update/delete/insert. Adapted with permission from ref (513). Copyright 2018 IEEE. (d) AF-coupled CrI_3_ layers in a CrI_3_ bilayer.^9^ Adapted with permission from ref (9). Copyright 2018 Springer Nature.

Finally, recent results of skyrmions on 2D magnets may induce additional work on skyrmionic devices. The use of skyrmions for applications has already been put forward in several technologies, from devices for data storage to components for logic functions, neuromorphic or reservoir computing systems.^509^ The most prominent application of skyrmions is the racetrack device initially proposed for domain walls.^510^ Replacing domain walls with skyrmions has important advantages. A large number of recent papers have been devoted to several types of skyrmion-based racetrack memories based on the motion and manipulation of skyrmions in a track of magnetic material.^476,509^ Current-induced motions of skyrmions in 2D magnets have been already demonstrated, as in Figure 46a showing sequential images of the motion of skyrmions (with a diameter around 200 nm) induced by current pulses in a track of FGT.^26^ For 2D magnets, such results are an initial step toward the implementation of a skyrmion racetrack memory (Figure 46b) storing data by aligning objects like beads on an abacus and moving such a train of skyrmions from an injector of skyrmions to a detector to read the stored data.^476,509^ Up to now, the current-induced motion of skyrmions have been mainly studied in 3D layers of magnetic metals as Co or CoFeB in which the defects and roughness lead to pinning effects and finally to nonuniform velocities.^511^ The expected advantage of 2D magnets as the FGT of Figure 46a should be a lower density of defects and more uniform velocities. One can even see that the motion of skyrmions in FGT, Figure 46a, is almost uniform.

In addition, 2D magnets provide interesting opportunities beyond the usual scheme of skyrmion-based racetrack memory. A couple of recent works have been devoted to skyrmions in magnetic layers antiferromagnetically coupled by interlayer exchange interactions as for instance, Co layers separated by a layer of Ru, see right inset in Figure 46c.^512^ The resulting “AF skyrmions” have several advantages. The compensated magnetizations lead to a decrease of the dipolar fields and smaller interactions between skyrmions. In addition, for current-induced motion, there is a compensation of the transverse deflections of the skyrmions by the so-called skyrmion Hall effect and the motion is along the current direction (along the track). Figure 46c and right inset show schematics proposed for racetrack memory with AF skyrmions.^513^Figure 46d shows how this concept could be studied in a bilayer of CrI_3_ in which the magnetizations of the two layers are antiferromagnetically coupled.^9^

One of the most important current challenges in spintronics is the development of low-power components to reduce the continuous increase of energy consumption by information technologies. It is already starting with the massive production of low power STT-MRAMs for computers and smartphones. Devices based on 2D magnets can participate in the next generation with components harnessing commands by voltage or relativistic SOTs and concepts based on the topological properties of skyrmions for further reductions of energy consumption and faster speeds. We have shown that, in several types of these devices, the 2D magnets should have significant advantages over the 3D materials. However, the obvious bottleneck, as we have mentioned, is the excessively low ordering temperature of the 2D magnets. The challenge is increasing this temperature, and we have described some promising results on this problem. Putting the temperature issue aside, it turns out that 2D magnets, whether alone or integrated in vdW heterostructures, can improve the performance of several types of spintronic devices. It can be illustrated by the comparison between 3D and 2D SOT-MRAMs in Figure 45. In other domains of technology, 2D magnets provide a pathway to other device concepts. As scientists, we are happy to explore these intriguing roads in physics and technology.

## Magnetic-Topological Phases

Insulators are known to be nonconducting because of a finite energy gap that separates the conduction and valence bands. Over the years, the differences between insulators have been considered only quantitatively, as for example the difference in the band dispersion and in the energy gap size. Over the past decade, however, it has been demonstrated that insulators can actually be further classified into different classes according to the topology of their band structures. For instance, the usual ordering conduction and valence bands of an ordinary insulator can be inverted by strong spin–orbital coupling, leading to a topological insulator (TI).^517^ The inverted bulk band structure topologically gives rise to metallic surface states. Therefore, a topological insulator is characterized by gapless surface states inside the bulk energy gap. These surface states commonly exhibit a Dirac cone-type dispersion in which spin and momentum are locked-up and perpendicular to each other. Topological insulators have been observed in many materials (e.g., ref (517) and references therein), such as HgTe and Bi_2_Se_3_.

Recently, a great interest has been triggered by the discovery of topologically nontrivial states in materials that are not insulators, such as topological metals and magnetic topological metals, involving Weyl and Dirac Fermions.^518−520^ While the existence of massless Fermions was demonstrated in 1929 by Hermann Weyl, Weyl Fermions have remained elusive until very recently with their discovery in condensed matter systems. In solid-state band structures, Weyl Fermions exist as low-energy excitations of the WSM, in which bands disperse linearly in 3D momentum space through a node termed a Weyl point. The whole ground state (surface and bulk) of Weyl metals are exotic, identified by topological Fermi arcs on the surface and chiral magnetic effects in the bulk. Thus, the topological metals have expanded the repertoire of exotic topological states, making unforeseen physics readily accessible and constitute a fascinating recent topic of modern quantum matter research.

Magnetic topological phases of quantum matter are an emerging frontier in physics and material science.^514,516,517,521−527^ Magnetic Weyl/Dirac semimetals are topological materials expected to host Weyl Fermions as emergent electronic quasiparticles and to display fascinating interplay between the topological invariants and the magnetic order. Theoretically, it is expected that in a magnetic topological system there is a wealth of topological phases associated with broken time-reversal symmetry.^518,528^ In addition, topological magnets may show the occurrence of intriguing topological phase transitions upon approaching the magnetic transition temperature. Also, the typical energy scale for spin–orbit coupling is only 0.1 eV, which has hindered the search for wide band gap topological insulators useful for technical applications. By contrast, magnetic exchange splitting is easily of the order of 1 eV, so that large and robust band inversions may be easier to produce through magnetism. For all these reasons, the study of magnets with topological band structure has emerged as an exciting research path. In our quest to find suitable systems to investigate the magnetic topological semimetals, we have identified a few systems that will be discussed in the following.

The interplay of symmetry, relativistic effects and the magnetic structure, in magnetic materials, allows for the realization of a wide variety of topological phases through Berry curvature design. Weyl points and other topological electronic bands can be manipulated by various external perturbations like temperature, magnetic fields and pressure, which results in exotic local properties such as the chiral or gravitational anomaly and large topological Hall effects, concepts which were developed in other fields of physics such as high energy physics and astrophysics.

**Figure 47:**
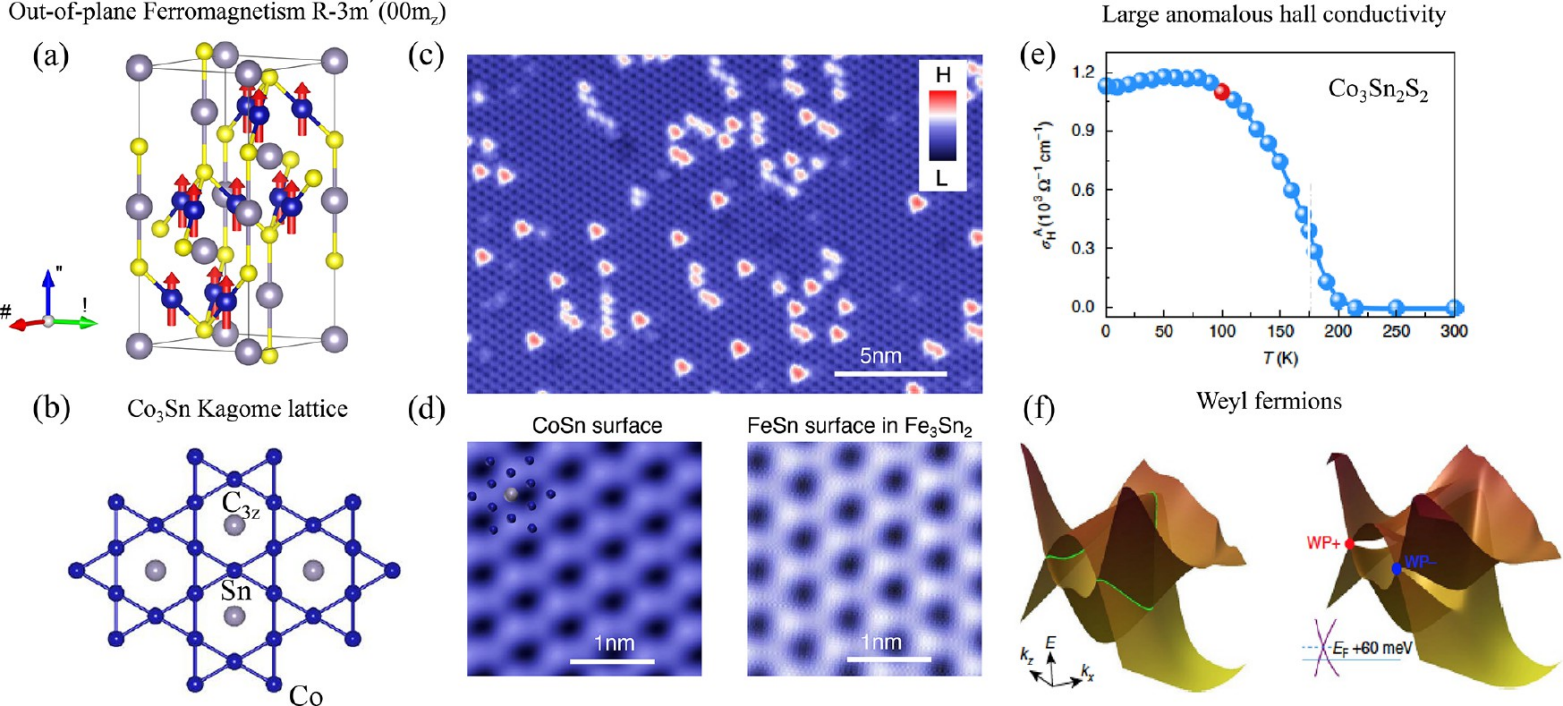
(a) Magnetic structure of Co_3_Sn_2_S_2_, showing a FM ground state with spins on Co atoms aligned along the *c*-axis. (b) Kagome lattice structure of the Co_3_Sn layer. (c) Topographic image of the CoSn surface. (d) A zoom-in image of the CoSn surface (left) that shows similar morphology with the FeSn surface (right) in Fe_3_Sn_2_. The inset illustrates the possible atomic assignment of the kagome lattice. Panels (a–d) are adapted with permission under a Creative Commons CC BY 4.0 license from ref (514). Copyright 2020 Springer Nature. (e) The temperature dependence of the intrinsic anomalous hall conductivity in Co_3_Sn_2_S_2_. (f) Left: linear band crossings form a nodal ring in the mirror plane. Right: Spin–orbit coupling breaks the nodal ring band structure into opened gaps and Weyl nodes. The Weyl nodes are located just 60 meV above the Fermi level, whereas the gapped nodal lines are distributed around the Fermi level. Panels (e) and (f) are adapted with permission from ref (515). Copyright 2018 Springer Nature.

One strategy to find FM Weyl semimetals is to look for materials that exhibit an anomalous Hall effect. The anomalous Hall effect can in part be attributed to scattering mechanisms, but there is also an intrinsic contribution arising from the Berry curvature of the band structure. Accordingly, being monopoles of Berry curvature, Weyl points (or topological nodal lines), as observed in magnetic topological semimetals, increase the anomalous Hall conductivity. Using this approach, several transition-metal-based kagome magnets were identified as ferro- or ferri-magnetic Weyl semimetal candidates, as they feature both large Berry curvature fields and unusual magnetic tunability.^514−516,525,529−532^ The kagome lattice is a 2D pattern of corner-sharing triangles. With this unusual symmetry and the associated geometrical frustration, the kagome lattice can host peculiar states including flat bands,^516^ Dirac Fermions^525,529^ and spin liquid phases.^530,533^ In the transition-metal based kagome family, the magnet Co_3_Sn_2_S_2_ (Figure 47a,d)^515,531^ is found to exhibit both the largest anomalous Hall effect (Figure 47e) and anomalous Hall angle.^515^ From the crystallographic point of view, Co_3_Sn_2_S_2_ has a layered structure with a CoSn kagome lattice (Figure 47b). Cleaving a sample at cryogenic temperatures often reveals Sn and S terminated surfaces as demonstrated by our recent STM study.^516^

**Figure 48:**
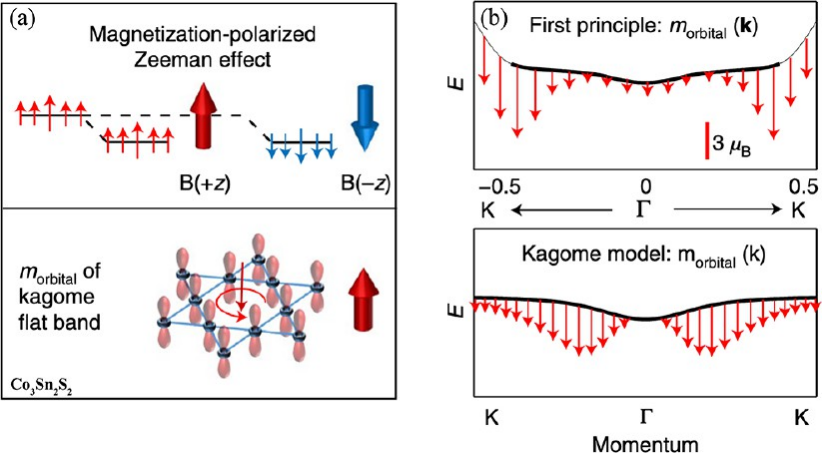
(a) Upper panel: Illustration of the magnetization-polarized Zeeman effect for Co_3_Sn_2_S_2_. Lower panel: illustration of the large negative orbital magnetism of the flat band in the kagome lattice. (b) Upper panel: Orbital magnetism for the flat band calculated from first principles. The magnetic moment (red arrows) is plotted along the flat band. The red bar marks the units of the magnetic moment value. Lower panel: Orbital magnetism from the magnetic kagome lattice model. The magnetic moment (red arrows, arbitrary unit) is plotted along the flat band. All panels adapted with permission from ref (516). Copyright 2019 Springer Nature.

In addition to these two dominant surfaces, we also rarely found CoSn surfaces (Figure 47c, left panel of (d)) which lies under the S surface. An enlarged view of this surface reveals a similar morphology similar to the FeSn surface (right panel of Figure 47d) in Fe_3_Sn_2_ at the atomic level, both of which are consistent with the transition metal based kagome lattice structure as seen in the STM images in Figure 47d. This material has a FM ground state (Curie temperature of *T*_*C*_ = 177 K) with a magnetization arising mainly from the cobalt moments. Density functional theory (DFT) calculations have predicted 6 pairs of Weyl points located only 60 meV above the Fermi level^515^ (Figure 47f). Theoretical calculations show Fermi arcs, the protected topological surface states characterizing Weyl semimetals, below the Fermi level on the (001) surface. Moreover, using scanning tunneling spectroscopy, a pronounced peak at the Fermi level was observed, which was identified as arising from the kinetically frustrated kagome flat band.^516^ High-field STM experiments evince that state exhibits an anomalous magnetization polarized many-body Zeeman shift, dominated by an orbital moment that is opposite to the field direction. Such unusual negative magnetism (Figure 48a,b) is induced by spin–orbit coupling quantum phase effects tied to nontrivial flat band systems.^516^

**Figure 49:**
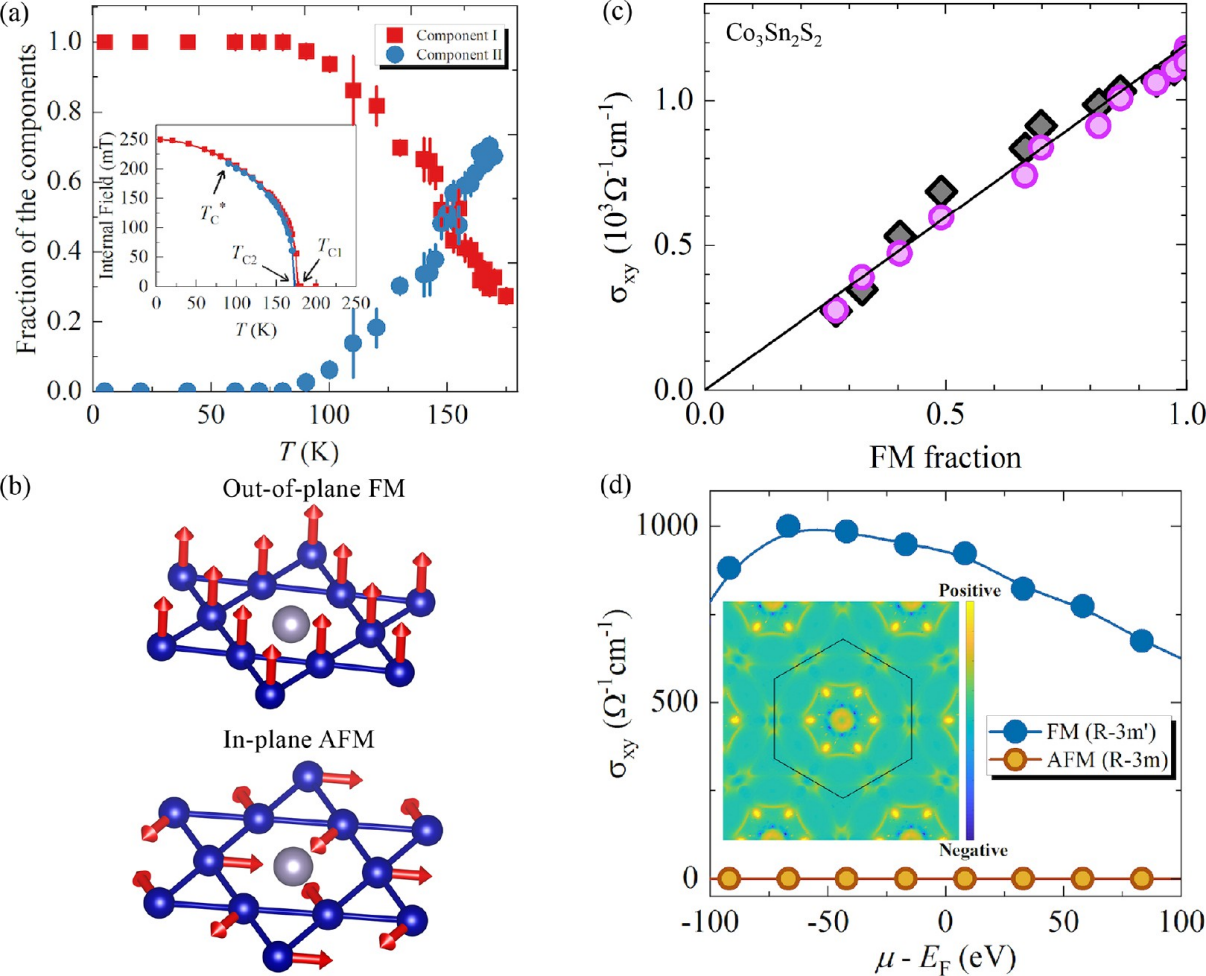
(a) The temperature dependence of the relative volume fractions of the two magnetically ordered regions. Arrows mark the critical temperatures *T*_*C*1_ and *T*_*C*2_ for FM and AF (or AFM) components, respectively as well as the transition temperature , below which only FM component is observed. (b) Spin structures of Co_3_Sn_2_S_2_, *i.e.*, the FM and the in-plane AF (or AFM) structures. (c) The correlation plot of anomalous hall conductivity versus FM fraction. (d) Calculated AHC for out-of-plane FM and in-plane AF structures. The inset shows the calculated Berry curvature distribution in the BZ at the FM phase. All panels are adapted with permission under a Creative Commons CC BY 4.0 license from ref (514). Copyright 2020 Springer Nature.

Despite the knowledge of the occurrence of ferromagnetism below *T*_*C*_ = 177 K^515^ with spins aligned along the *c*-axis (see Figure 47a) there was no report of its interplay with the topological band structure. We have carried out high-resolution ambient and high-pressure μSR^370,377,534^ and neutron diffraction, combined with first-principles calculations, muon stopping site calculations and group theoretical analysis, to systematically characterize the phase diagram of Co_3_Sn_2_S_2_.^514^ We found two magnetically ordered fractions in Co_3_Sn_2_S_2_ with different moment sizes: At low temperatures, the very homogeneous out-of-plane FM structure (top panel of Figure 49b) is dominant, and with increasing temperature, the fraction of the in-plane AF (bottom panel of Figure 49b) state grows and becomes the dominant component at 170 K. Both order parameters exhibits a monotonous decrease and clear separation with increasing temperature.

Figure 49a shows the temperature dependence of the relative volume fractions of the out-of-plane FM and in-plane AF ordered regions along with the total magnetic fraction. Arrows mark the critical temperatures *T*_*C*1_ and *T*_*C*2_ for FM and AF components, respectively as well as the transition temperature , below which only FM component is observed. It is clear that the volume of AF component develops at the cost of the FM one. The key finding of our μSR experiments is the observation of a phase separated ferromagnetically and antiferromagnetically ordered regions in the large temperature range in the Weyl semimetal Co_3_Sn_2_S_2_ (Figure 49a).

We further show^514^ that the competition of these magnetic phases is tunable through applying either an external magnetic field or hydrostatic pressure. I note that although the measured moment size of Co is only of the order of 0.1 μ_B_, using μSR, we were able to measure the whole temperature dependence of such a tiny moment as well as to determine the magnetic structure in Co_3_Sn_2_S_2_.^514^ High sensitivity of the μSR technique to extremely small moments is a tremendous advantage over other magnetic probes.

One of the most striking electronic effects in Co_3_Sn_2_S_2_ is a large intrinsic anomalous Hall conductivity and a giant anomalous Hall angle, due to the considerably enhanced Berry curvature arising from its topological band structure. Astonishingly, we found that the temperature dependence of the anomalous Hall conductivity very closely matches the volume fraction of the out-of-plane FM component, giving rise to an excellent linear correlation between these two quantities (Figure 49c).^514^ This is one of the rare examples of such a quantitative correlation between the magnetic volume fraction and the Berry curvature induced anomalous Hall conductivity (Figure 49c).

From first principles calculations,^514^ we concluded that the AHC is dominated by the *c*-axis FM structure (Figure 49d), providing an explanation for the reduction of the AHC when the ordered volume fraction of the out-of-plane FM state decreases. These results have strong impact since we establish Co_3_Sn_2_S_2_ as a material that hosts topological electronic states and frustrated magnetism. Our experiments suggest that the Co spins have both FM interactions along *c*-axis and AF interactions within the kagome plane, and there is a temperature dependent competition between these two ordering tendencies. The interplay between this intricate magnetism and the spin–orbit coupled band structure further induces nontrivial variations of its topological properties, which is characterized by a striking correlation between the giant anomalous Hall transport and the FM volume fraction. Our results demonstrate thermal tuning of Berry curvature effects mediated by changes in the frustrated magnetic structure. Our findings implicate control and manipulation of topological Fermions *via* thermodynamic and magnetic interactions. Moreover, the interplay between different magnetically ordered regions, each of which possesses distinct topological invariants, can possibly give rise to physical properties at the magnetic domain boundaries. Additionally, it enables the design of switchable magnetic materials with desired magneto-transport properties for potential technological applications.

**Figure 50:**
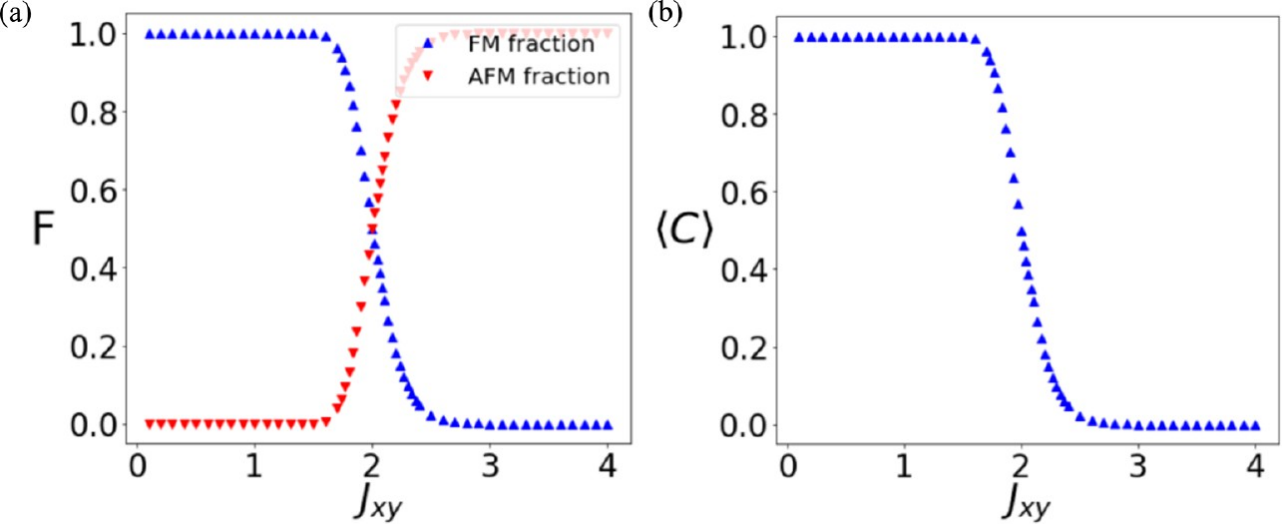
(a) Calculated fractions of the ferromagnetism *F* and antiferromagnetism (1 – *F*) in Co_3_Sn_2_S_2_ and (b) averaged Chern number as a function of the in-plane AF correlation *J*_*xy*_, taking into account fluctuations in the Hund’s coupling. All panels are adapted with permission under a Creative Commons CC BY 4.0 license from ref (535). Copyright 2020 American Physical Society.

Motivated by the scaling between AHC and the FM fraction in Co_3_Sn_2_S_2_, analytical model^535^ was built taking into account both localized electrons giving rise to a magnetic transition and conduction electrons producing topology of Bloch bands on the kagome lattice. Itinerant and localized electrons (the latter forming core spin-1/2’s on each atom) are coupled through a strong Hund’s FM mechanism. Hund’s coupling along *z*-direction, Mott physics and electron-mediated interactions between the half-filled orbitals was shown^535^ to reproduce the out-of-plane ferromagnetism and an AF transition with a 120° spin ordering in the *xy* plane, as we observed experimentally.^514^ Interestingly, it was shown that the (average) system’s magnetization in the *z* direction smoothly reduces to zero after the transition, producing the progressive canting of the spins, such that the statistically averaged Chern number follows the FM fraction (see Figure 50). This shows an excellent agreement with our experimentally obtained striking correlation between topological hall conductivity and the FM fraction.

According to DFT calculations^514^ the energies of the out-of-plane FM and in-plane AF configurations are similar. It seems that temperature or In-doping affects^536^ this intricate balance, and tip it in favor of the in-plane magnetic structure. The hydrostatic pressure also causes a suppression of both FM and AF states,^537^ but a pressure as high as 20 GPa is needed at which both orders are suppressed simultaneously. In Co_3_Sn_2–*x*_In_*x*_S_2_ however, only small amount of In is sufficient to push the system toward the AF or helical state. Indium substitution introduces holes to the system and at the same time increases the separation of the kagome layers, while hydrostatic pressure shrinks the lattice and no doping is expected. This suggests that lattice expansion and/or hole doping disfavors the out-of-plane FM state. It was also recently shown that the coupling of this material’s topological properties to its magnetic texture leads to a strongly exchange biased anomalous Hall effect, which was argued to be likely caused by the coexistence or competition of two magnetic phases.^538^ The interplay between the competing magnetic states and the spin–orbit coupled band structure further seem to induce nontrivial variations of the topological properties of Co_3_Sn_2–*x*_In_*x*_S_2_, which is evidenced by a nonmonotonous In-doping dependence of the anomalous Hall conductivity.^539^ The AHC was also shown to be selectively tuned from 0 to a very large value 1600 Ω^–1^ cm^–1^ in magnetic Heusler compounds *via* suitable manipulations of the symmetries and band structures of the materials.^540^ A large AHC was also predicted and recently observed in noncollinear antiferromagnets such as Mn_3_Ge or Mn_3_Sn, in which the Berry curvature originates from the noncollinear spin structure.^532,541,542^

Topological electronic response was also found for the rare earth-transition metal based kagome system TbMn_6_Sn_6_. Namely, Shubnikov–de Haas quantum oscillations with nontrivial Berry phases from relatively low fields (∼7 T), a large AHC (0.14 *e*^2^/h per Mn kagome layer), arising from Berry curvature fields, and quasi-linear (∝ *H*^1.1^) magnetoresistance (MR) likely resulting from linearly dispersive electrons. Moreover, TbMn_6_Sn_6_ was found to demonstrate a bulk-boundary correspondence between the Chern gap and the topological edge state, as well as Berry curvature field correspondence of Chern-gapped Dirac Fermions.^522,543^ Thus, it is identified as a promising topological magnetic system.^522,543^ Using μSR, neutron diffraction and magnetization we identified the low-temperature magnetic state in TbMn_6_Sn_6_, which seems to be responsible for the low-*T* topological transport properties.^544^ A number of nontrivial magnetic phases and a large topological Hall effect was also observed in another rare earth-transition metal based system YMn_6_Sn_6_.^527^ A nematic chirality mechanism, which comes from frustrated interplanar exchange interactions that trigger strong magnetic fluctuations, was discussed as an origin of the topological Hall effect.

A giant AHC was also found in KV_3_Sb_5_, an exfoliable, highly conductive semimetal with Dirac quasiparticles and a vanadium kagome net. Even without report of long-range magnetic order, the anomalous Hall conductivity reaches 15507 Ω^–1^*cm*^–1^. It was theoretically suggested that the kagome sublattice in KV_3_Sb_5_ is acting as tilted dynamic spin clusters, giving rise to an enhanced skew scattering effect, responsible for large AHE. Charge-sensitive probes have suggested exotic charge order^545^ in the kagome superconductor KV_3_Sb_5_, which can lead to giant anomalous Hall effect. Using the μSR technique we provided systematic evidence for the existence of time-reversal symmetry-breaking by charge order.^546^ We showed that the breaking of time-reversal symmetry is spontaneous and that the magnetic response can be enhanced by external magnetic field. The time-reversal symmetry breaking charge order is indicative of extended Coulomb interactions, which would lead to correlated superconductivity. In the superconducting state, we find superconductivity of multigap nature and with a dilute superfluid (low density of Cooper pairs), indicating that the superconductivity is indeed correlated and unconventional.

## Synthesis and Sample Preparation

In this section, we review the main methods of preparing atomically thin samples of vdW magnetic materials *via* exfoliation from larger bulk crystals (and the methods to grow the parent crystals), protecting sensitive samples from the environment, and bottom-up growth by vapor synthesis methods. Controlling sample quality and defects is also discussed.

### Exfoliation of 2D Samples

Due to their weak interlayer interactions, magnetic vdW crystals can be readily cleaved to produce 2D flakes with thicknesses on the nanometer scale (<10 nm).^547−549^ The primary method for isolating such thin flakes from bulk magnetic vdW materials is through mechanical exfoliation, similar to other vdW 2D materials. This method involves physically cleaving layers from single crystals with adhesive polymers (such as commercially available tape or polydimethylsiloxane (PDMS)).^548,550−552^ The process flow is simple: place a single crystal on the adhesive polymer, fold and unfold the adhesive polymer several times, then transfer the polymer with exfoliated flakes onto the desired substrate (often quartz, sapphire, or SiO_2_).^553−555^ This process can be done within an inert environment (such as a glovebox) without exposing flakes to solvents, heat, or atmosphere, which is ideal for air- and moisture-sensitive vdW magnets. Liquid phase exfoliation (LPE), in which various mechanistic methods (such as ion intercalation, ion exchange, and sonication) are used to break single crystals into a solution of single-layer sheets, is an alternative exfoliation process^554,556−560^ implemented for select 2D magnets.^561−564^ Though highly scalable, flakes obtained through LPE are typically lower quality compared to those produced by mechanical exfoliation.^559^

**Figure 51:**
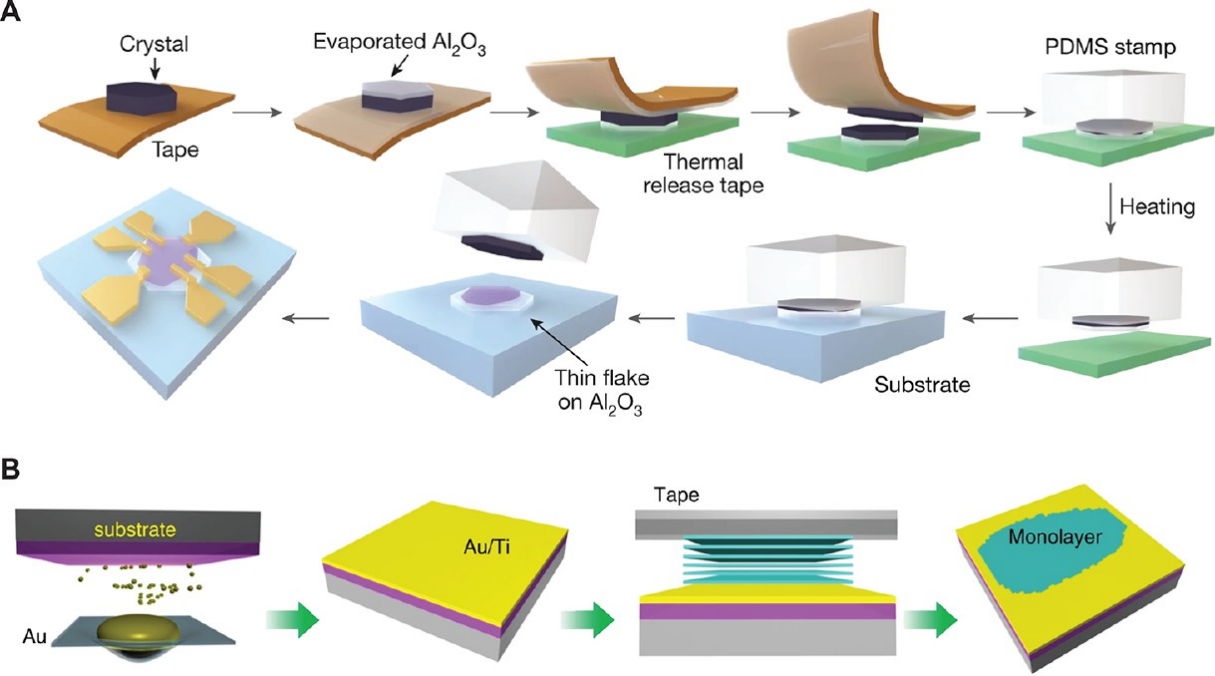
Mechanical exfoliation methods. (A) Schematic of the Al_2_O_3_ film-assisted mechanical exfoliation.^12^ The strong adhesion between the crystal and the Al_2_O_3_ film makes it possible to exfoliate layered crystals that are otherwise difficult to cleave from SiO_2_ surfaces using conventional methods.^12^ Adapted with permission from ref (12). Copyright 2018 Springer Nature. (B) Schematic of the Au-assisted exfoliation process. First, a thin layer of Au is deposited onto a substrate, then a freshly cleaved bulk crystal is placed on the Au layer. The Au is then removed with a KI/I_2_ aqueous solution etchant.^566^ Adapted with permission under a Creative Commons CC BY license from ref (566). Copyright 2020 Springer Nature.

Depending on the material, mechanical exfoliation with adhesive polymers can reliably yield flakes on the order of ∼10 μm × 10 μm in lateral dimensions,^555^ large enough for device fabrication and measurement. However, for especially difficult to exfoliate materials, film-assisted methods have been developed. These entail exfoliation with adhesive polymers followed by transfer to substrates with large adhesion compared to the interlayer binding energy of the crystal. For 2D magnets such as Fe_3_GeTe_2_ and MnBi_2_Te_4_, an Al_2_O_3_ assisted exfoliation was established (Figure 51A).^12,111^ It consists of covering a bulk surface with an Al_2_O_3_ thin film, exfoliating the stack onto thermal release tape, which is subsequently picked up and transferred onto a suitable substrate with PDMS. The increased yield of exfoliated flakes from the Al_2_O_3_ process is attributed to the increased contact area and affinity between the Al_2_O_3_ film and the target crystal.^12^ When exceptionally large 2D flakes are required, Au can be used as an exfoliation substrate to isolate millimeter-sized monolayers of a variety of 2D materials, including 2D magnets such as Fe_3_GeTe_2_ (Figure 51B).^565−569^ Though Au-assisted exfoliation is by far the most effective at isolating large monolayers, it necessarily requires a solvent to remove the Au layer before flakes can be utilized, which can damage especially sensitive 2D magnets. In contrast, the Al_2_O_3_ technique is solvent-free, allowing for feasible preparation of pristine sensitive flakes.

**Figure 52:**
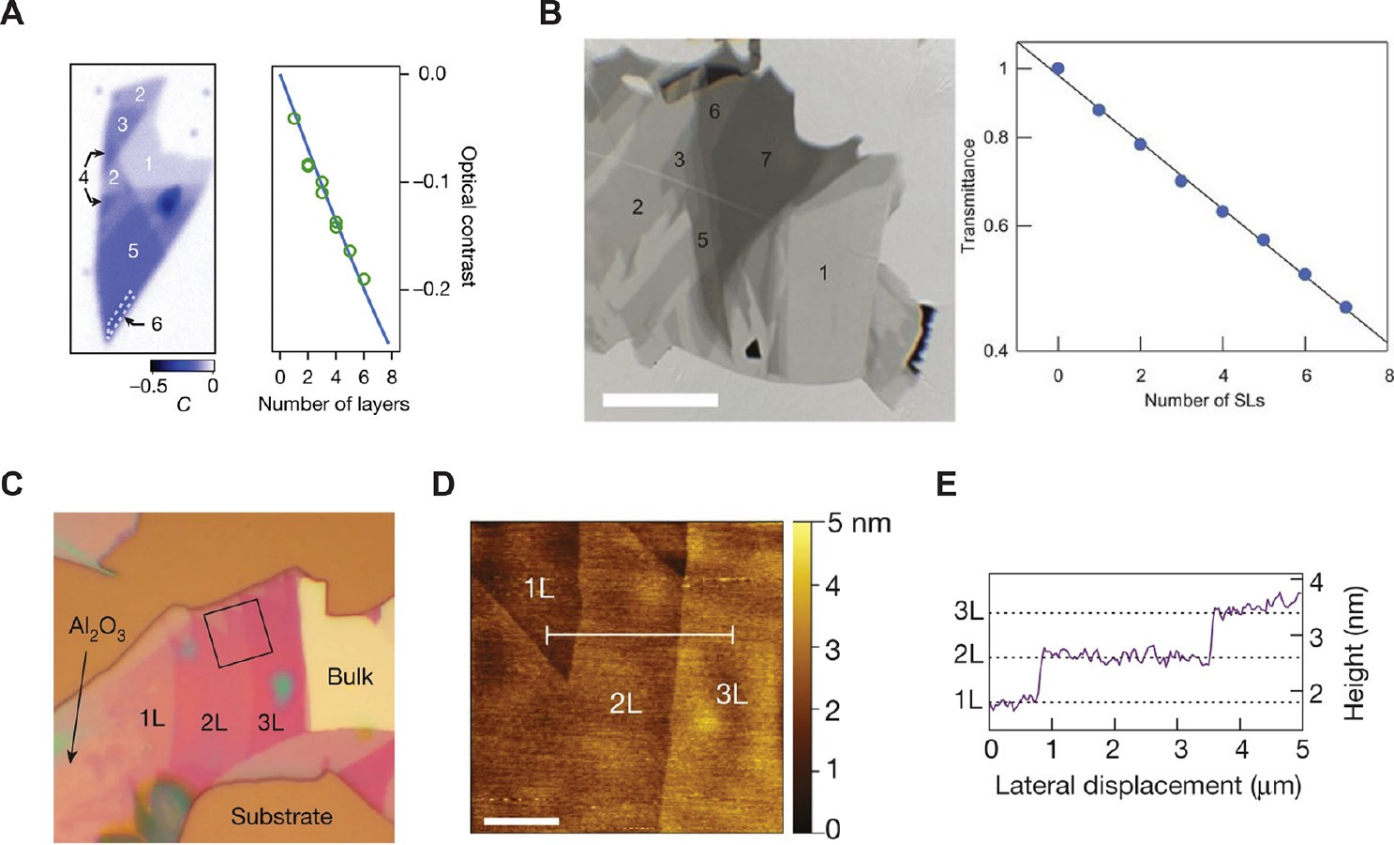
Identification of exfoliated layer numbers. (A) Optical contrast map of a representative CrI_3_ flake (left) and the corresponding optical contrast per number of layers (right).^5^ Adapted with permission from ref (5). Copyright 2017 Springer Nature. (B) Left: Optical image of few-layer flakes of MnBi_2_Te_4_ exfoliated onto Al_2_O_3_. The corresponding layer number is labeled on selected flakes. The scale bar is 20 μm. Right: MnBi_2_Te_4_ transmittance versus layer number.^111^ Adapted with permission from ref (111). Copyright 2020 AAAS. (C) Optical image of few-layer Fe_3_GeTe_2_ flakes exfoliated onto Al_2_O_3_.^12^ (D) AFM image of the area in (C) marked by a solid black square.^12^ The scale bar is 2 μm. (E) Height profile plotted versus length along the white line in (D).^12^ Panels (C–E) are adapted with permission from ref (12). Copyright 2018 Springer Nature.

After exfoliated flakes are transferred onto a substrate, the universal method for identifying flake thickness is through optical contrast.^5,6,12,111,122,552,570−574^ As demonstrated by Novoselov et al.,^552^ single-layer flakes can be optically identified under a microscope if a suitable substrate is chosen. Light incident on the flake/substrate interface will interfere to generate an optical contrast that depends on flake thickness (Figure 52A–C), wavelength of the incident light and thickness of the substrate, and flake material (Figure 52A–C).^571,575^ Though optical contrast is an indirect measure of flake thickness, it can be combined with AFM imaging to map contrast to layer number (Figure 52D,E).^5,6,12,122^ AFM also screens for polymer or solvent residue introduced during the exfoliation process. In certain materials, Raman spectroscopy is used as a noninvasive probe to determine flake thickness by taking advantage of a systematic shift of particular Raman modes as a function of layer number in the few-layer limit.^122,574,576^

### Protection of 2D Samples

Atomic-scale magnetic flakes are often extremely sensitive to the nanofabrication steps required to produce functional devices, including exposure to air, moisture, conventional polymer solvents, and heating. Therefore, preparing flakes for device fabrication and measurement requires careful encapsulation in an inert environment. To solve this problem, the dry-polymer-transfer technique^577^ is used to completely encapsulate flakes in hBN, which simultaneously protects the flake from degradation during fabrication^578^ and provides a high-quality dielectric substrate.^579^ This approach, combined with the ability to perform exfoliation and flake searching inside an inert environment (such as a glovebox),^580^ allows for the preparation of a wide array of high-quality magnetic 2D materials with diverse properties for measurements.

### Crystal Growth and 2D Deposition

Currently, there are no scientific reports showing that monolayer or few-layer thick vdW magnetic crystals can be produced on large scales using commercially viable bottom-up synthesis techniques such as atomic layer deposition (ALD) or CVD. The lack of large-scale production methods stems from the limited environmental stability of many vdW magnetic crystals and/or a lack of knowledge surrounding surface chemistry that would enable the layer-by-layer deposition of vdW magnets in low dimensions, and for this reason, the scientific community is reliant on crystals produced by well-established bulk crystal synthesis routes. Additionally, bulk crystals produced from chemical vapor transport (CVT), sublimation, and flux zone growth techniques are highly crystalline and can be synthesized from precursors of the highest purity, rendering them free of magnetic impurities like Co, Fe, and Cr.

To produce these crystals, many different crystal growth techniques may be employed, but the most suitable technique can often be determined by considering the elemental precursors being used (chalcogen versus halogen) and by having a comprehensive understanding of the binary or ternary phase diagrams of the desired crystals. A survey of recent literature on vdW magnetic materials shows that chemical vapor transport (CVT), sublimation, and flux zone techniques are the most widely used. After these layered crystals are grown, a conventional mechanical exfoliation technique^552^ (as described above) can be used to obtain monolayer and few layer thick crystals on the desired substrate. While the previously mentioned techniques are currently the most common, recent studies are beginning to develop synthesis methods, which will expand our understanding of how to isolate low dimensional magnetic materials using bottom-up growth methods.

### Bulk vdW Crystal Growth

#### Vapor Transport

CVT reactions were established in the 1930s, and over the decades has proven to be an incredibly reliable and effective route to synthesize high-quality, defect-free crystals, including many layered magnetic crystals.^581−583^ To achieve the highest quality impurity-free crystals by CVT, high purity (3N+) precursors are vacuum sealed in thick (1–2 mm) quartz ampules. In addition to having the utmost control over the environmental conditions for the growth, the vacuum sealing process is necessary to minimize the ambient pressure inside the ampule, as incredibly high pressures can build within the ampule due to the high temperature thermal processing required for crystal growth.

In a typical vapor transport reaction, precursor materials are transported from a source across a temperature gradient to a sink, where Le Chatelier’s principle governs the transport direction. Exothermic reactions transport from a cold zone to a hot zone, whereas endothermic reactions transport from a hot zone to a cold zone.^584^ To ensure that the precursor materials transport across the temperature gradient, they must achieve a gaseous state which is typically facilitated by a transport agent.^585^ To illustrate these points, halogen-assisted transport is usually required for the endothermic transport of elemental precursors for transition metal chalcogenides and transition metal phophosulfides/selenides (MnPSe_3_, CoPS_3_, and NiPS_3_),^581^ while additional transport agents are typically not required for the growth of transition metal halides (CrI_3_ and CrBr_3_), as the halogen is already contained within the crystal matrix.^293^

Similar to CVT, physical vapor transport (PVT) or direct sublimation of precursor compounds may also be used to reliably produce bulk high-quality, single crystals of vdW magnets.^586,587^ It has been established that direct sublimation is a low-cost and effective technique, where researchers do not need advanced vacuum manifolds or complex multizone furnaces that are requirements for chemical vapor transport reactions. However, vacuum sealing precursor compounds in quartz ampules may also be used to synthesize high-quality layered crystals.^588^ To synthesize transition metal halides such as CrCl_3_, commercially available polycrystalline powders are positioned inside of a cold, single-zone tube furnace in either an open-ended or vacuum-sealed quartz tube. The furnace is then heated to the desired temperature for sublimation, and the desired material transports, nucleates, and crystallizes in the cold zone of the furnace. Large, high-quality crystals grow directly on the quartz walls, and can be obtained with in shorter times (24–48 h) as compared to CVT.^7^

#### Flux Zone Growth

Melt-phase synthesis routes are a common technique for the synthesis of ternary tellurium based 2D magnets (Cr_2_Ge_2_Te_3_ and Fe_3_GeTe_2_),^290,323,589^ and unlike CVT, which can require a transport agent foreign to the crystal matrix, tellurium may be used as a solvent for the precursors, from which high-quality crystals will precipitate and grow out of the melt as the solution cools. To achieve the highest quality crystals, stoichiometric quantities of flux (solvent) and precursors (solute) are loaded into an inert crucible and vacuum sealed (∼10^–5^ Torr). Careful consideration when choosing a suitable crucible must be made to avoid undesired reactions between the crucible and flux. Much like CVT reactions, rather than a compatible transport agent, a suitable flux must be selected that is either part of the crystal matrix or has complete immiscibility with the elemental precursors at the required synthesis temperatures.

The sealed ampule containing the precursors and flux are first heated above their melting temperatures, and at this point the furnace temperature is held constant so the elemental precursors can completely mix into a homogeneous melt. The ampule is then slow cooled over a period of several days, and during the cooling process the desired material precipitates out of the melt, where spontaneous nucleation and crystallization occurs. It must be mentioned that the above process is highly governed by a material system’s respective phase diagram, and that growth parameters and optimization can be accelerated by having an intimate understanding the desired alloy’s binary or ternary phase diagram. However, a material system’s phase diagram is likely unknown; in which case these parameters must be empirically determined. After the growth has completed, the flux must be removed from the bulk crystals, where the most common technique to remove this flux is *via* a high temperature centrifugation process.^590^

### Large-Scale Layer-by-Layer Deposition

While CVT, sublimation and flux methods are invaluable tools to discover and understand the novelties of champion material systems, these synthesis routes pose many challenges that make them unsuitable to be used in industrial manufacturing of thin films. The greatest challenge is that in order to isolate few-layer or monolayer thick sheets of vdW materials, they must first be exfoliated prior to being transferred onto a desired substrate. The most widely used technique for the exfoliation of vdW crystals being scotch-tape technique introduced by Novoselov^552,591−593^ and a more detailed review of this and other exfoliation techniques are covered (in the previous section). If these materials are ever to be considered viable options for future electronics applications, progress toward developing the fundamentals of bottom-up synthesis techniques of vdW magnetic materials must be made.

#### Vapor Deposition

**Figure 53:**
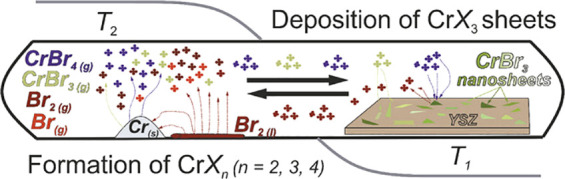
Scheme for the one-step synthesis and vapor transport of CrX_3_ (X = Br, I) micro- and nanosheets directly on YSZ substrates shown by the example of CrBr_3_. Prior to the CVT process for deposition of the respective nanolayers the introduction of chromium powder and bromine (Br_2_ in small sealed capillaries) lead to the formation of CrBr_3_ (solid) (Cr_(s)_ + 1,5 Br_2(l)_ → CrBr_3(s)_) and gaseous CrBr_*n*(g)_ (*n* = 2, 3, 4) at *T*_2_. By application of a temperature gradient (*T*_2_ → *T*_1_) chemical vapor transport is achieved for deposition of CrBr_3_ micro- and nanosheets on YSZ substrates directly. The reaction course is similar for the formation of CrI_3_, while in contrast to this scheme CrCl_3_ is utilized as presynthesized compound, that is not introduced by mixture of the elements. Reproduced with permission from ref (600). Copyright 2019 John Wiley and Sons.

**Figure 54:**
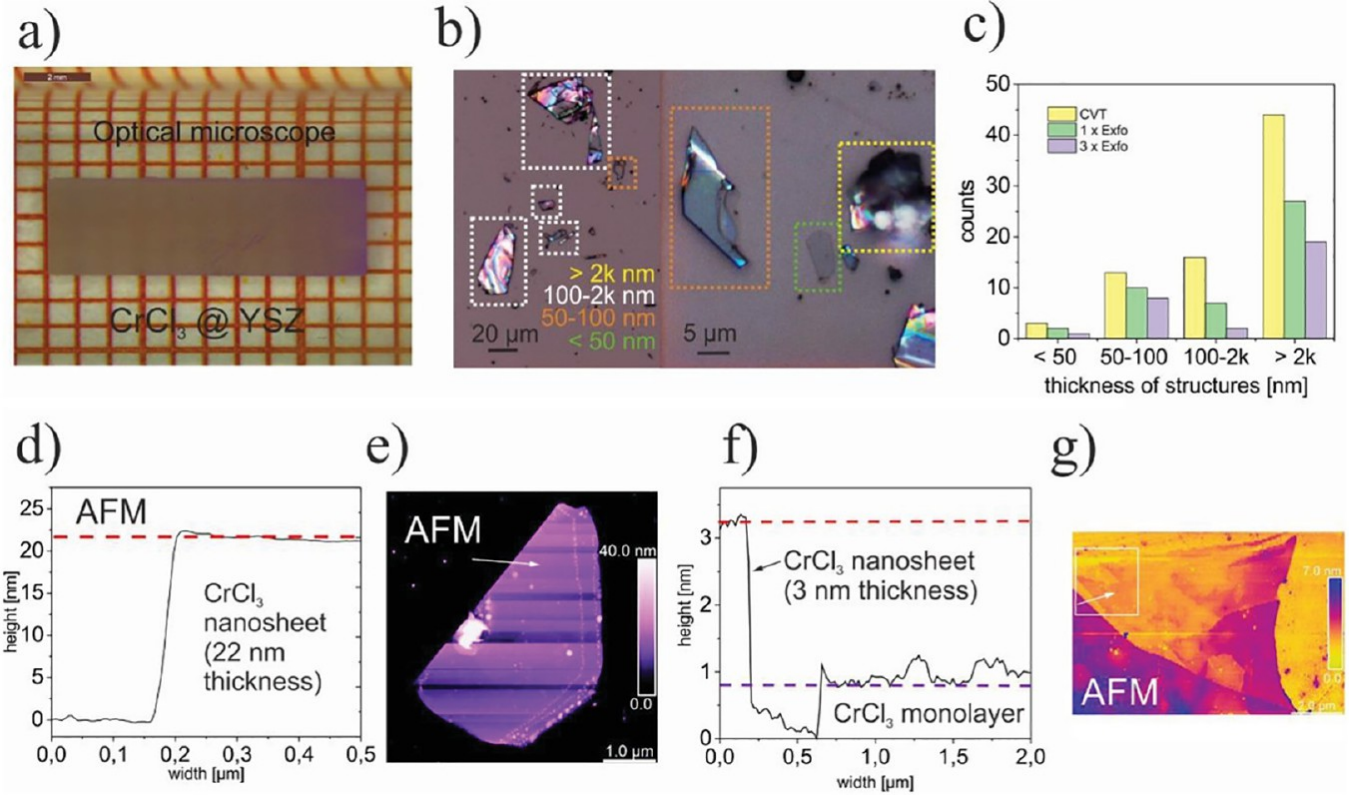
Crystal growth by vapor transports of CrCl_3_ on YSZ substrates. (a) Optical microscopy of CrCl_3_ micro- and nanocrystals at YSZ substrate. (b) Optical microscope image of YSZ substrate surface with CrCl_3_ sheets with respective thicknesses in dotted boxes. (c) Distribution of thicknesses of CrCl_3_ structures on a YSZ substrate after CVT (yellow), after one time of exfoliation (green) and after three times of exfoliation (purple). (d) AFM measurement the height profile of a CrCl_3_ nanosheet. (e) Corresponding AFM image of measurement of (d) the white arrow is indicating the measurement. (f) AFM measurement of the height profile a CrCl_3_ ultrathin sheet (red line) and monolayer (purple line) after three repeats of exfoliation. (g) Corresponding AFM image of measurement of (f) the white line is indicating the monolayer AFM measurement. All panels are reproduced with permission from ref (600). Copyright 2019 John Wiley and Sons.

Bottom-up synthesis of vdW magnets is still in its infancy, and researchers are just now beginning to unravel the fundamentals of how to synthesize the materials in their low-dimensional limits. It is no surprise that many of synthesized vdW magnets are chalcogen-based,^95,594−598^ as many CVD and PVD techniques have already been established for these material systems. As researchers begin to explore other classes of magnetic materials such as transition metal halides and ternary MPX_3_ materials (MnPS_3_, NiPS_3_, FePSe_3_) and MOHs (CrOCl), more experimental challenges present themselves as the growth dynamics become more complicated and as the precursors become more volatile and reactive. Recently, Grönke *et al*. produced thin flakes of a wide variety of vdW magnets using a modified CVT technique on different substrates (Figure 53).^599−601^ To achieve this, Grönke *et al*. computationally modeled the vapor transport reactions and determined several experimental parameters, such as precursor type, growth temperature, and transport rate of the desired materials. Furthermore, by predetermining the transport rates they were able to understand how growth times impact the thickness of the resultant films, which has enabled them to significantly accelerate their research efforts. They confirmed that CrX_3_ (X = I, Br, Cl) was successfully synthesized onto yttrium stabilized zirconia (YSZ) substrates (Figure 54), with only a small number of detectable impurities surficially incorporated into the crystal lattice (CrBr_3_) or adsorbed onto the surface of the crystals (CrI_3_ and CrCl_3_). While Grönke *et al*. showed that this technique may be applied to transition metal halides, it may have greater implications for other vdW magnetic crystals as researchers aim to better understand the surface/interface chemistry required to realize highly crystalline flakes in the monolayer or few-layer limit.

This method has also been expanded to dihalide systems, where earlier this year, Liu *et al*. synthesized thin flakes of NiI_2_ on hBN and Si/SiO_2_ using a conventional PVD, showing that a similar method used by Grönke *et al*. can be used to synthesize transition metal halides on more conventional substrates.^602^

#### Molecular Beam Epitaxy

The rebirth of studying layered vdW magnets has been sparked by the discovery of intrinsic ferromagnetism in monolayers of vdW systems, such as CrGeTe_3_ and chromium trihalides. Molecular beam epitaxy (MBE) is a very refined ultrahigh vacuum (UHV) bottom-up vacuum deposition technique enabling the layer-by-layer deposition of thin films, and naturally, MBE lends itself to the synthesis of layered vdW materials in their low dimensional limits.^95,99^ Being a UHV deposition method, it has become a fundamental tool for researchers and industry to control and study the impacts of precursor quality, interface and nucleation dynamics, in addition to the effects of alloying and doping of 2D materials at nanometer thicknesses.

**Figure 55:**
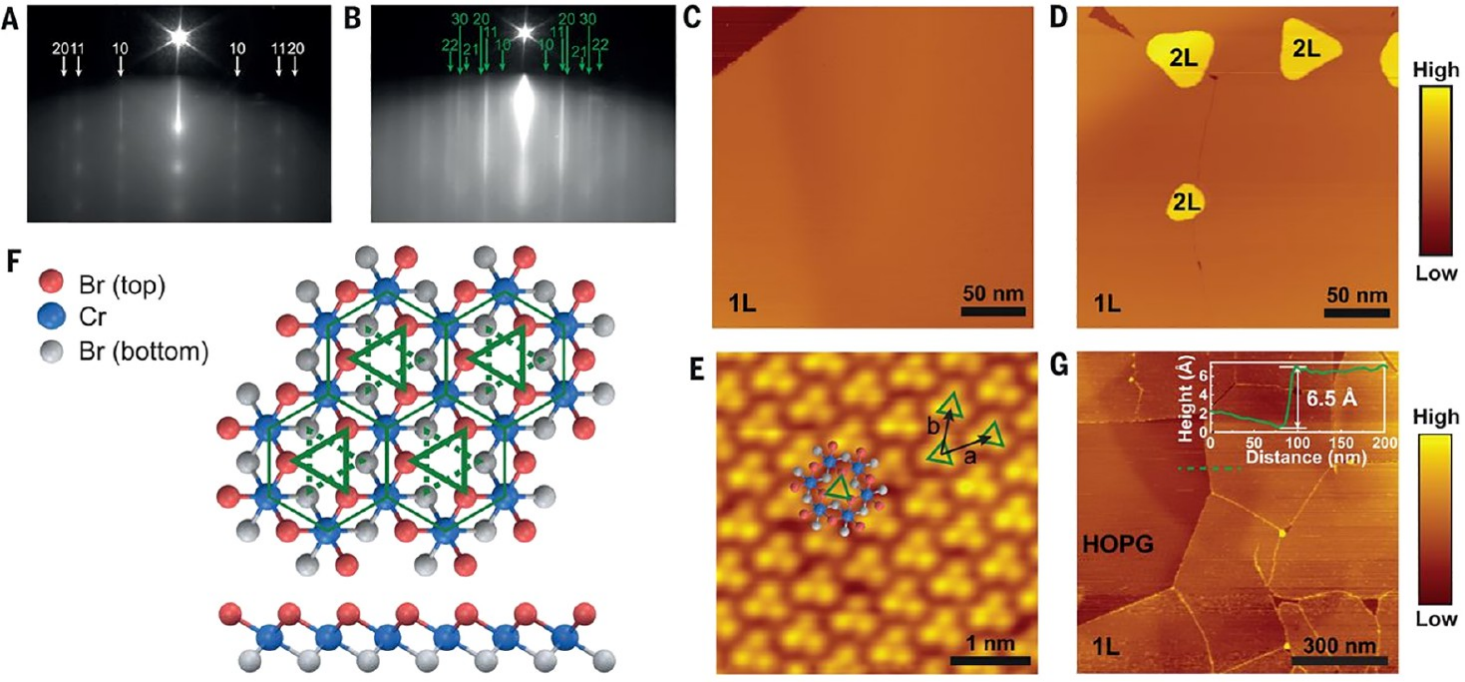
(A, B) RHEED patterns with indicated diffraction orders of (A) the bare HOPG substrate and (B) the MBE-grown CrBr_3_ film. (C, D) STM images of (C) the CrBr_3_ monolayer with (D) bilayer islands. The scan parameters were as follows: *V*_*b*_ = 1.1 V, *I* = 100 pA, *T* = 5 K for (C) and *V*_*b*_ = 1.5 V, *I* = 100 pA, *T* = 5 K for (D). (E) Atomically resolved image of a monolayer CrBr_3_ with an overlaid atomic structure. The scan parameters were as follows: *V*_*b*_ = 1.5 V, *I* = 500 pA, *T* = 5 K. The lattice constants were determined to be 6.3 Å for the primitive vectors **a** and **b**, consistent with the bulk values. (F) Illustrations of the top and side views of the monolayer CrBr_3_ atomic structure. The Cr atoms form a honeycomb lattice sandwiched by Br atoms. Within the Cr honeycomb lattice, the top and bottom surfaces of Br atoms form single triangles but with opposite orientation, indicated by solid and dotted green lines, respectively. (G) AFM image of monolayer CrBr_3_ with partial coverage. A line-cut profile across the monolayer and bare substrate is shown with a monolayer height of 6.5 Å. All panels are adapted with permission from ref (20). Copyright 2019 AAAS.

While MBE has been heavily used for the synthesis of magnetic transition metal chalcogenides, recent reports show that transition metal halides may be synthesized at monolayer scales using this method.^20,603^ Weijong Chen *et al*. successfully synthesized CrBr_3_ at the monolayer limit by using compound source MBE, where CrBr_3_ powder was used as the source of Bromine. Growth dynamics for CrBr_3_ films were determined by *in situ* reflected high energy electron diffraction (RHEED) (Figure 55) and STM. While this is an isolated study on CrBr_3_, it will inevitably be a launchpad for future epitaxial growths of halide based vdW magnets at larger scales on more conventional substrates.

### Synthesis of CrSBr

Beyond prototypical 2D magnets such as Cr_2_Ge_2_Te_6_, Fe_3_GeTe_2_, and CrX_3_ (X = halide),^5^ ternary chromium sulfide bromide (CrSBr) has emerged as an exciting 2D material due to its magnetic structure in which each CrSBr layer is ferromagnetically ordered in-plane and coupled antiferromagnetically to adjacent layers.^110^ The synthesis of CrSBr requires advanced processes, combining both solid state and molecular chemistry. The primary reagent is disulfur dibromide (S_2_Br_2_), a highly air sensitive liquid prepared by heating a mixture of solid sulfur and liquid molecular bromine in a sealed pressure vessel, followed by vacuum distillation.^604^ Beyond this, CrSBr synthesis follows the canon model of CVT in a multizone furnace with a temperature gradient between 1223 and 1153 K,^110,605^ with S_2_Br_2_ acting as both reagent and CVT agent. Although not yet realized experimentally, other members of the chromium chalcogenide halide (CrEX, E = chalcogenide) family are predicted to have extremely high magnetic transition temperatures (e.g., CrSeBr with *T*_*N*_ = 150 K), motivating the development of more controllable syntheses.^606−608^ A mixed halide compound CrSBr_1–*x*_Cl_*x*_ (*x* = 0.33) with the same structure as CrSBr was previously reported, giving hope for the possibility of synthesizing additional compositions.^609^

### Defect Studies in vdW Magnets

**Figure 56:**
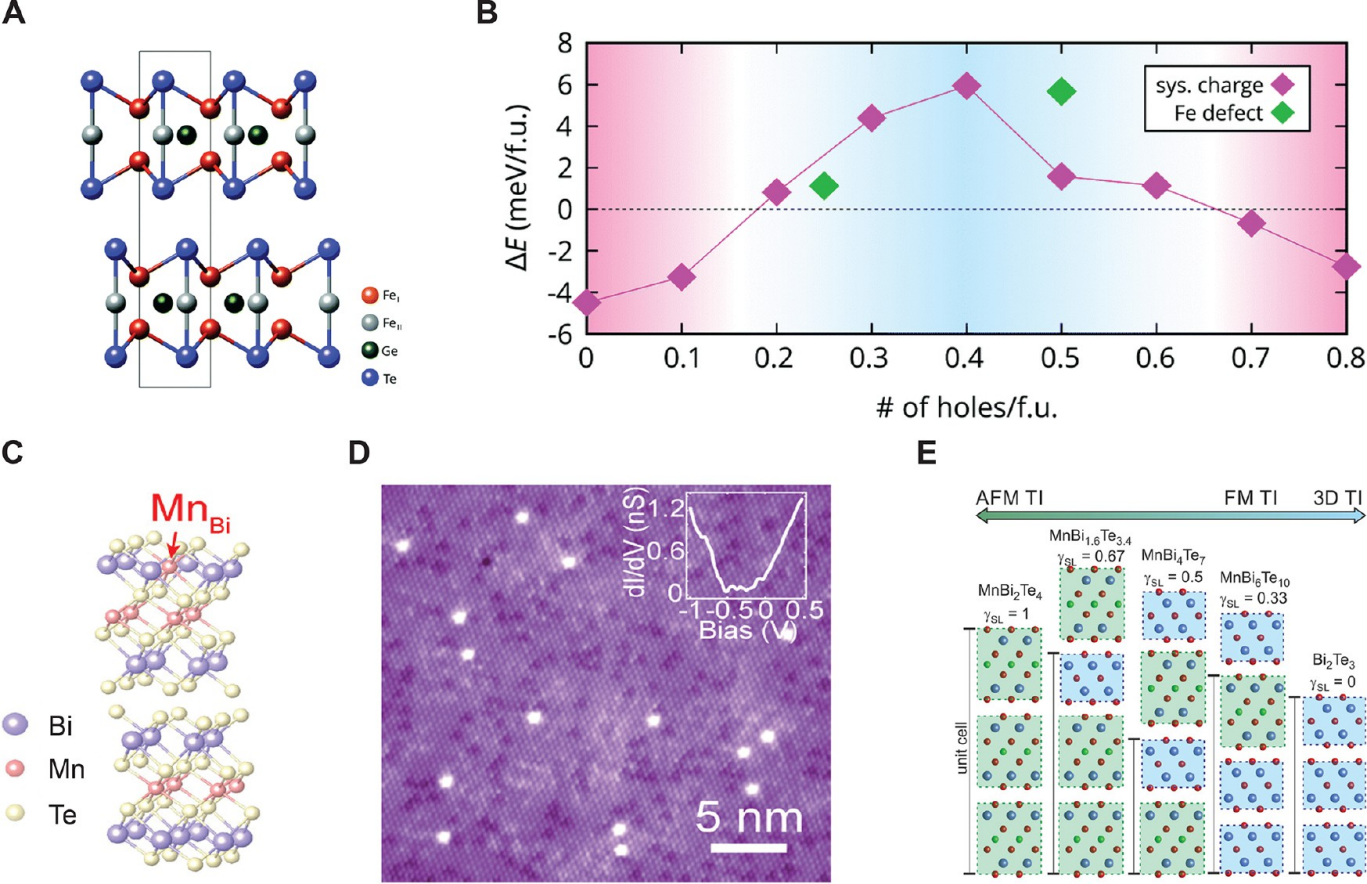
(A, B) The structure and ground states of iron-deficient Fe_3–*x*_GeTe_2_. (A) Side view of stoichiometric Fe_3_GeTe_2_. Fe_I_ (red) and Fe_II_ (silver) are two inequivalent Fe sites with +3 and +2 formal charges, respectively. The Fe_I_–Fe_I_ interactions are mostly responsible for interlayer AF ordering while Fe_I_-Fe_II_ and Fe_II_–Fe_II_ couplings are FM. With Fe defects or doping, Fe_I_–Fe_II_ and Fe_II_–Fe_II_ become dominant and push interlayer ordering into FM. (B) The calculated energy differences (Δ*E*) between interlayer AF and FM phases as a function of hole concentration. For Δ*E* > 0, FM is favored (between 0.2 and 0.6 holes per formula unit). Panels (A) and (B) are adapted with permission from ref (615). Copyright 2020 American Chemical Society. (C) The layered crystal structure of MBT. The red arrow indicates the Mn sites in which antisites have been identified. (D) STM of the surface of a cleaved MBT crystal; white spots show the presence of multiple antisite point defects. Panels (C) and (D) are adapted with permission from ref (616). Copyright 2020 American Chemical Society. (E) Crystal structure representation of the (MnBi_2_Te_4_)_*m*_(Bi_2_Te_3_)_*n*_ series, ranging from AF to FM with various compositions derived from codepositional MBE. Here, we see that with the addition of Bi_2_Te_3_ layers, the MnBi_2_Te_4_ layers cannot be coupled together and the material becomes less AF. This is a prime example of an off-stoichiometry defect. Panel (E) adapted with permission from ref (617). Copyright 2020 AIP Publishing.

While defects are an inevitable part of synthesis, they can significantly alter the properties of materials. This effect is often magnified at the 2D limit.^610−612^ Defects have been explored in traditional 2D materials but they remain largely unexplored in 2D magnets.^612−614^ Two materials in which the effects of defects have been examined are Fe_3_GeTe_2_ and MnBi_2_Te_4_. Fe_3_GeTe_2_ is a 2D metal with a layered FM ordering and a high *T*_*C*_ of 220 K decreasing to 130 K at the monolayer.^333^ It has received much attention due to the tunability of its *T*_*C*_*via* electrostatic gating,^12^ its large anomalous Hall current,^336^ and its corresponding potential as a spintronic material.^499^ However, the origins of its bulk magnetic ordering have come to light only recently: Theory predicts an interlayer AF structure with intralayer FM ordering, but all experimental probes show that it has both interlayer and intralayer ferromagnetism. This discrepancy comes from defects in CVT-grown Fe_3_GeTe_2_. Theory calculations show that Fe defects are highly favorable. There are three incommensurate Fe sites (Figure 56A). The one which dominates interlayer AF interactions (Fe_I_–Fe_I_) is greatly decreased by defects and doping, while the two interactions (Fe_II_–Fe_I_ and Fe_II_–Fe_II_) that contribute to interlayer FM ordering are increased (Figure 56B).^615^ Hence experimentally observed phases, such as Fe_2.75_GeTe_2_, are fully FM while completely stoichiometric Fe_3_GeTe_2_ is predicted to be AF between adjacent sheets.^77^ This demonstrates a clear opportunity for tuning bulk magnetic properties by controlling the number of Fe defects.

The relationship between defects and magnetic properties, and how they relate to the emergence of exotic insulating phenomena, has been thoroughly investigated in MnBi_2_Te_4_.^616,618−621^ By examining native Mn and Bi point defects (Figure 56D), Huang *et al*. quantified how Mn and Bi vacancies directly affect the local Fermi level by inducing localized defect states in the band gap.^619^ This work has led to further changes in the synthesis of MnBi_2_Te_4_ to either decrease defect concentration or control the electronic properties MnBi_2_Te_4_ through defect engineering. Controlling this system’s electronic states are critical for the observation of topological phenomena, such as the quantum anomalous Hall effect. In a different study, MBE was employed to produce off-stoichiometry phases by incorporating differing amounts of Bi_2_Te_3_ sub units (see Figure 56E); this off-stoichiometry leads to different stacking configurations of magnetic layers, resulting in modular magnetic ordering.^617,622^ Simultaneously, MBE was used to include point defects (Mn, Bi, Te vacancies), effectively chemically doping the system, to alter the Fermi level.^616^ By engineering stoichiometry and defect concentration through synthetic control, the electronic and magnetic state can be directly manipulated.^616^

### Challenges and Perspectives

While well-established bulk crystal synthesis techniques outlined above are critical for researchers to identify champion material systems and probe their fundamental material properties, very little is currently understood about how different bulk synthesis techniques impact the magnetic properties of vdW magnets. This raises many questions about how thermal processing, precursor materials, flux and transport agents impact the magnetic character of these materials. Adding to the complexity of the current state of vdW magnets, even less is known about how defect density, magnetic impurities, and overall crystallinity impact the performance of these material systems, especially in their 2D limits where these factors will have even a greater impact on their quantum magnetic phenomena (*e.g.*, skymionic effects). Commonly used chemical vapor transport samples are known to contain significant number of point defects and learning from our mistakes in diluted magnetic semiconductor will be essential to reaching reliable conclusions in these emerging fields. Initial reports in vdW materials show that flux growth is most suited to reduce these point defects but the question still remains if it can reach 6N or even higher purities. The purity is going to be a major challenge to overcome in order to reach quantum coherence in quantum devices involving 2D vdW magnets.

Another natural concern is around their material stability in air or ambient conditions. While it is true that some of the high-performance devices can operate under vacuum, it still raises questions about the photo degradation effects as well as long-term material stability. More studies are needed to clarify how one can improve the material stability through surface functionalization, encapsulation, or even curing chemically active defect sites without sacrificing the material properties.

Beyond the fundamental studies of these materials, the overarching aim is to bridge the gap between laboratory studies and the eventual development of next-generation technologies. To accomplish this, much work needs to be done to advance the large area (inch scale) synthesis and deposition of 2D vdW magnets, and researchers pushing the boundaries of layer-by-layer growths of these materials will need to overcome similar challenges that were paramount to producing large area TMD systems with high crystallinity and low defect densities. This gives a brief insight into the current state of vdW magnet synthesis and presents many exciting challenges and opportunities for scientists and engineers to solve which will establish the fundamentals and push the capabilities of vdW magnets from the lab and into cutting-edge quantum devices.

Key to understanding the role of defects in 2D magnets is to identify and quantify them both in the bulk and 2D regime. Methods of inspecting these features can be broadly divided by their spatial resolution and the directness by which they probe magnetic properties. The level of detail and resolution concerning magnetic properties obtained from various techniques is very often indirectly related to the feasibility of the experiment. On one hand, SQUID measurements give no microscopic details about spin configuration, but provide information about macroscopic magnetic properties and are widely available. On the other hand, spin-polarized STM can be used to gain electronic and magnetic information on the atomic scale but is prohibitively costly and time-consuming.^623^ Techniques such as Lorentz TEM^624^ and magnetic force microscopy^625^ can characterize magnetic domains between 2 and 20 nm,^626^ accessing the mesoscale, but also require expensive instrumentation and a high degree of training. To truly understand the magnetic effects of defects within a system, surveys utilizing a wide array of measurements are necessary. Crucial to improving our understanding of defects is making these myriad techniques widely available, or cultivating emerging atomic-level probes of magnetic defects such as nanopatterned SQUID,^627^ scanning SQUID,^627^ or NV-center magnetometry.^21^

## Mechanical Properties and Strain Engineering

Mechanical properties play an important role in the applications of 2D magnets, such as strain engineering and flexible electronic devices. The thickness reduction of vdW materials to the atomic scale normally leads to mechanical enhancements. For example, graphene has an intrinsic Young’s modulus of ∼1 TPa and fracture strength of ∼120 GPa,^628^ about 1–2 orders larger than those measured from bulk graphite.^629,630^ The same phenomena have also been observed from many other 2D materials. Atomically thin hBN, an insulating 2D material, has a Young’s modulus (*i.e.*, ∼865 GPa) and fracture strength (*i.e.*, ∼70 GPa) comparable to those of graphene.^631^ Although TMDs have relatively weaker interaction between metal and chalcogen atoms, the Young’s moduli and strengths of 1L MoS_2_ (∼270 and 23 GPa, respectively),^632^ 1L WS_2_ (302 and 47 GPa, respectively),^633^ and 1L WSe_2_ (258 and 38 GPa, respectively)^633^ are much higher than those of their bulk counterparts as well as most conventional materials. Two main reasons can be ascribed to this: a smaller probability of defects due to the dramatically decreased volume and less softening from the weak interlayer interactions at the atomic thicknesses.

**Figure 57:**
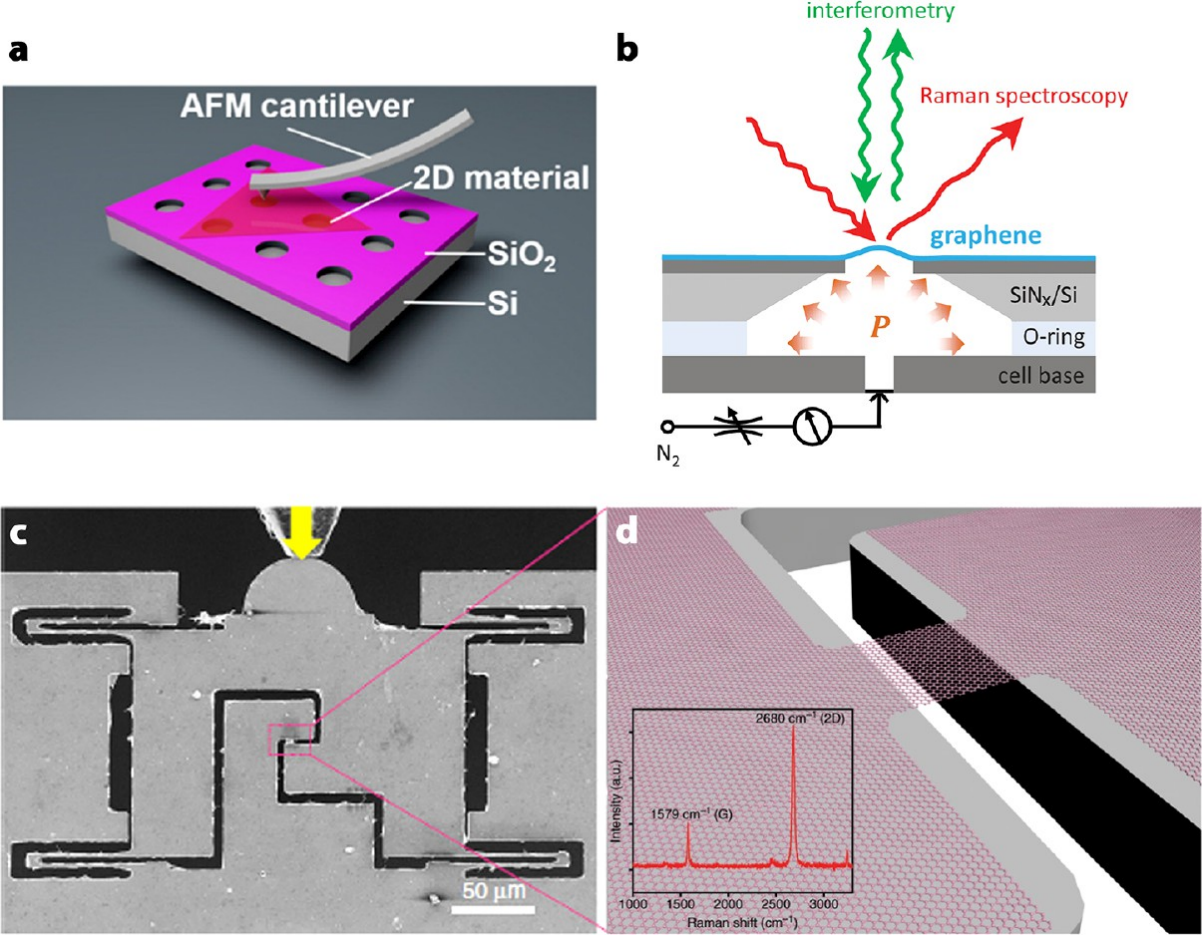
Common experimental techniques for measuring the Young’s modulus and strength of 2D materials. (a) Nanoindentation based on AFM.^634^ Adapted with permission under a Creative Commons CC BY license from ref (634). Copyright 2019 John Wiley and sons. (b) Bulge test involving interferometry and Raman spectroscopy.^635^ Adapted with permission from ref (635). Copyright 2017 American Physical Society. (c) Tensile testing push-to-pull micromechanical device controlled by an external pico-indenter in SEM, and the yellow arrow shows the loading direction. (d) Diagram showing the enlarged pink rectangle area in (c) with a suspended graphene sample after cutting by focused ion beam.^636,637^ Copyright 2009 Royal Society of Chemistry, copyright 2019 American Physical Society, copyright 2019 John Wiley and Sons, copyright 2017 American Physical Society, copyright 2020 Springer Nature, copyright 2014 Springer Nature. Panels (c) and (d) are adapted with permission under a Creative Commons CC BY 4.0 license from ref (636). Copyright 2020 Springer Nature.

Nevertheless, the mechanical properties of few-layer 2D materials are also subject to interlayer sliding. For example, although the in-plane covalent bonds in graphene are stronger than those in BN, the fracture strengths of 8–9L graphene and BN are close due to their different sliding tendencies under strain and compression, *i.e.*, few-layer graphene spontaneously slides between layers under an in-plane strain and large compression, while few-layer BN has large positive sliding energies under the same conditions to prevent it from sliding.^631^ Similar interlayer sliding phenomena have also been found in few-layer WS_2_ and WSe_2_.^633^ The Young’s modulus and strength of 2D materials are mostly measured by nanoindentation methods,^628,631−634^ along with bulge method (Young’s modulus only)^635,638^ and microtensile tests^636,637^ (Figure 57).

AFMs are usually used for the indentation measurements. In particular, a sharp tip at the end of a cantilever indents at the center of suspended 2D materials, and the nonlinear elastic properties and strength can be derived from the force-displacement relation (Figure 57a).^628,631−634^ For accuracy, the 2D material should not slide on a substrate during indentation, and the tip should have a larger stiffness than the 2D nanosheets (*e.g.*, diamond tip for graphene and BN). As atomically thin films, 2D materials are suitable for blister (bulge) tests, in which they are clamped over microholes and the stiffness can be calculated based on gas pressure-driven or electrostatically driven geometry changes (Figure 57b).^635,638−642^ In microtensile tests, suspended 2D materials of relatively large sizes are under uniform tensile stretch induced by special indentation-spring setups in scanning electron microscope (SEM), which is best at revealing the nonlocalized fracture strength and strain (Figure 57c).^636,637^ In addition to large Young’s modulus and strength, 2D materials also have extremely low bending moduli,^643,644^ strong adhesion to arbitrary surfaces,^638,645^ low fracture toughness,^637,646^ and increased friction coefficient.^647^ It should be emphasized that 2D materials are able to undertake large strains without failure,^628,631,648^ providing more opportunities for strain engineering. According to recent theoretical and experimental works, in-plane strain engineering can change the structural phase,^649,650^ electronic structure,^651,652^ and polarization^653,654^ of TMDs; strain can also induce pseudomagnetism in graphene.^655,656^

**Figure 58:**
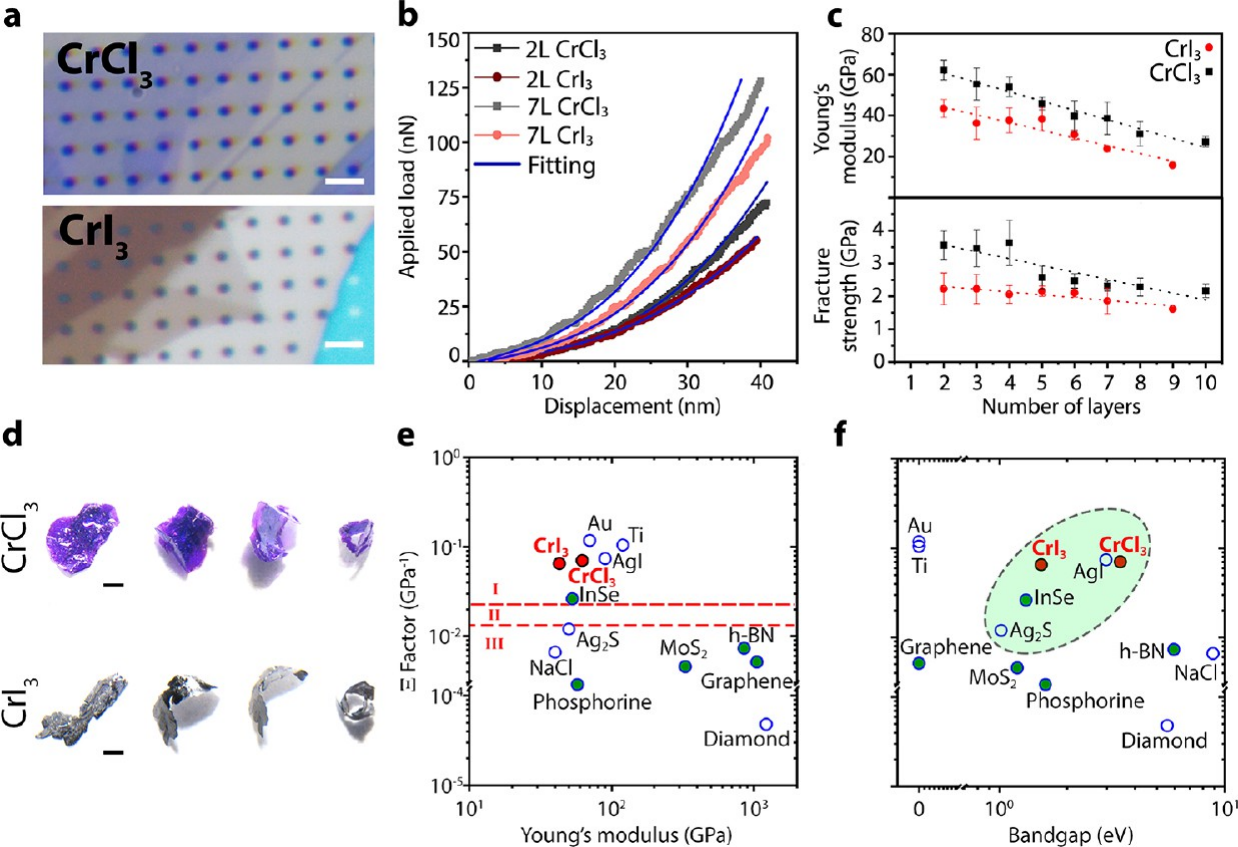
Mechanical properties of CrCl_3_ and CrI_3_. (a) Optical microscopy images of 2L and few layers CrCl_3_ and Crl_3_ suspended over microwells (600 nm in diameter) on a SiO_2_/Si substrate. (b) Load–displacement curves and the corresponding fittings of 2L and 7L CrCl_3_ and CrI_3_. (c) Volumetric Young’s modulus and breaking strength of 2–10L CrCl_3_ and CrI_3_, along with dashed lines that show the linear fits. (d) Demonstration of the good plasticity of bulk CrCl_3_ and CrI_3_ crystals *via* folding them into rings. (e) Deformability factor *versus* Young modulus, where I, II, and III correspond to plastic-flexible, potentially deformable and brittle-rigid regions, respectively. The experiential results of CrCl_3_ and CrI_3_ are shown as red-filled circles, and the other layered vdW materials are shown as green filled circles. (f) Deformability factor versus bandgap for the same materials as in (e), and the materials that may show exceptional plastic behavior are shown in the dashed line encircled green area.^659^ All panels are adapted with permission from ref (659). Copyright 2021 American Chemical Society.

There have been few experimental studies of the mechanical properties of 2D magnets. Very recently, the intrinsic elasticity and strength of atomically thin chromium trihalides, *i.e.*, CrX_3_ (X = Cl, I) mechanically exfoliated from their single crystals were measured by AFM-based nanoindentation at room temperature (Figure 58).^657^ The 2L CrI_3_ and CrCl_3_ had Young’s moduli of 62.1 ± 4.8 GPa and 43.4 ± 4.4 GPa, respectively, consistent with the theoretical predictions.^234,237,657,658^ Their fracture strengths were measured to be 3.6 ± 0.4 GPa and 2.2 ± 0.5 GPa, respectively; the maximum strains were in the range of ∼6.0–6.5%. The elasticity and strength of both materials decreased with increased layer thickness due to the high tendency of interlayer sliding, though this phenomenon was more prominent in CrCl_3_. The DFT calculations showed that CrCl_3_ and CrI_3_ had small sliding energy barriers in the equilibrium state and under in-plane strains.^659^ The interlayer sliding led to stress concentrations on the bottom layers during indentation, weakening the few-layer systems. This study indicates that atomically thin CrCl_3_ and CrI_3_ have one of the lowest elasticity and strength values among all 2D materials explored so far. The soft mechanical response and larger cleavage energy than shear (sliding) energy in the two materials give rise to the possibility of the superplastic phenomenon in their bulk crystals (Figure 58).

The number of theoretical investigations on the mechanical properties of 2D magnets is growing fast. The theoretical Young’s modulus (both 2D and 3D), fracture strength, Poisson’s ratio, and cleavage energy of monolayer magnets predicted so far are summarized in Table 8. Note that the fracture strength and strain of most 2D magnets have not been theoretically studied, except 2L CrCl_3_/CrI_3_^659^ and 1T VS_2_ and VSe_2_ along the zigzag direction.^660^

**Table 8:** DFT studies of the mechanical properties of 2D magnets

monolayer magnets	2D Young’s modulus (N/m)	theoretical thickness (Å)	Young’s modulus (GPa)	fracture strength (N/m)	Poisson’s ratio	cleavage energy (J/m^2^)	ref
FePS_3_	65.98–119.7	6.42	102.8–186.4		0.304	0.265	(1, 661, 662)
FePSe_3_	67.9–90.2	6.61	102.7–136.5		0.312	0.37	(1, 661, 662)
MnPS_3_	55.1–107.7	6.49	84.9–165.9		0.327	0.26	(1, 661, 662)
MnPSe_3_	36–60.8	6.67	54.0–91.2		0.35	0.23–0.24	(1, 661−663)
NiPS_3_	80.9–106.8	6.34	127.6–168.5		0.265	0.21	(1, 661, 662)
NiPSe_3_	78.8	∼6.52	120.9		0.275		(661)
CrSnTe_3_	64.8				0.283		(664)
CrSiTe_3_	40.8	6.86	59.5			0.35	(663)
CrGeTe_3_	38.3	6.9	55.5			0.38	(663)
K2CuF_4_	44.8					0.78	(665)
CrOCl	38.39–46.83					0.208	(666, 667)
CrI_3_	22–28	6.62	33.2–42.3	2.3–3.6 (2L)	0.253	0.155–0.28	(234, 237, 657−659)
CrBr_3_	28–29.3	6.11	45.8–48.0		0.278	0.19–0.295	(234, 658)
CrCl_3_	31–34	5.80–6.10	53.4–55.7	3.9–4.9 (2L)	0.297	0.13–0.3	(234, 658, 659)
FeSe	66.2–119.8	5.31–5.61	124.7–213.5		0.179–0.196		(657, 668, 669)
Fe_3_GeTe_2_	134 (C11)					∼0.01	(330)
VS_2_	85	5.71	148.9	12 (zz)	0.16		(660)
VSe_2_	53.8–71	6.1–7.0	88.2–101.4	9.8(zz)	0.18–0.229 5		(660, 670)
CrTe_3_	48–52	6.94	69.2–99.7			0.5	(671, 672)
Mn_3_Se_4_	18.76–25.97				0.371	0.58	(673)
Mn_3_Te_4_	22.6–29.42				0.293	0.53	(673)
Fe_2_Si	71	5.7	124.65				(674)
CoAsS	75.51				0.076		(675)
NiI_2_	45	6.54	68.8			0.26	(676)
NiBr_2_	50	6.09	82.1			0.242	(676)
NiCl_2_	54	5.8	93.1			0.223	(676)
CrPbTe_3_	46.9				0.318		(677)
CrPS_4_	36.8–56.4	6.2	59.4–91.0		0.28–0.44		(678, 679)

**Figure 59:**
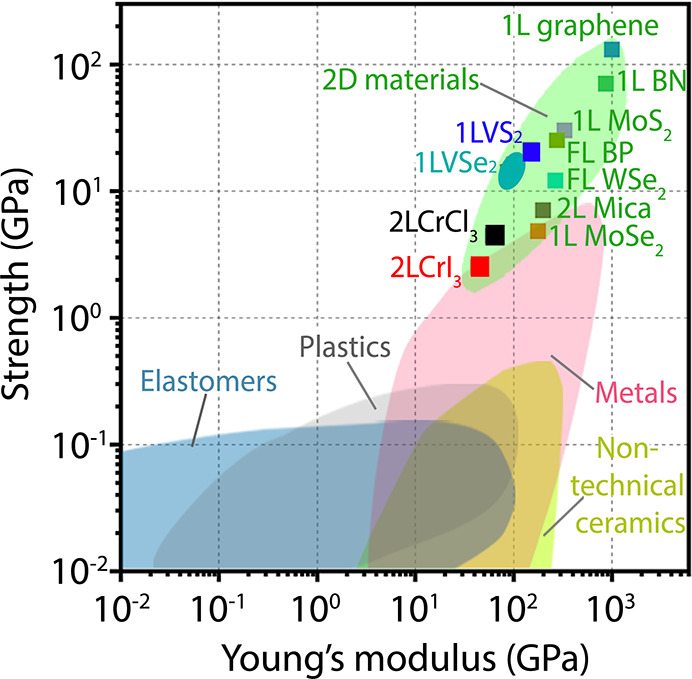
Modulus-strength graph. The Young’s modulus and fracture strength of 2D magnets are compared with those of conventional bulk materials and other 2D materials. Adapted with permission from ref (659). Copyright 2021 American Chemical Society.

Although 2D magnets have smaller Young’s moduli and strengths than most other 2D materials, they are stronger than their bulk counterparts and traditional magnetic thin films (Figure 59). For example, 1L FeSe has a theoretical Young’s modulus of 66.2–119.8 N/m (*i.e.*, 124.7–213.5 GPa);^657,668,669^ in comparison, that of FeSe thin films is only 41 GPa determined by acoustical measurements.^680^ A 1 μm-thick SmCo_5_ deposited by RF magnetron sputtering on Si 100) substrate has a reduced modulus of 43.09 ± 1.60 GPa measured by indentation,^681^ smaller than that of most of the 2D magnets listed in Table 8. Magnetic thin films comprised of magnetic particles in polymer matrix normally have even smaller modulus values. Strontium ferrite particles (SrFe_12_O_19_) suspended in a benzophenone tetracarboxylic dianhydrideoxydianiline/metaphenylene diamine polyimide matrix had biaxial modulus values in the range of 6 to 17.8 GPa, measured by a modified *in situ* load/deflection technique.^682^ We can also compare the strength values of 2L CrCl_3_ (3.6 ± 0.4 GPa) and 2L CrI_3_ (2.2 ± 0.5 GPa) with the “so-called” ultrahigh strength value of 40–400 μm-thick Fe – Co – Ni-based maraging steel and 316L austenite stainless steel magnetic sheets (∼1.61 GPa).^683^

**Figure 60:**
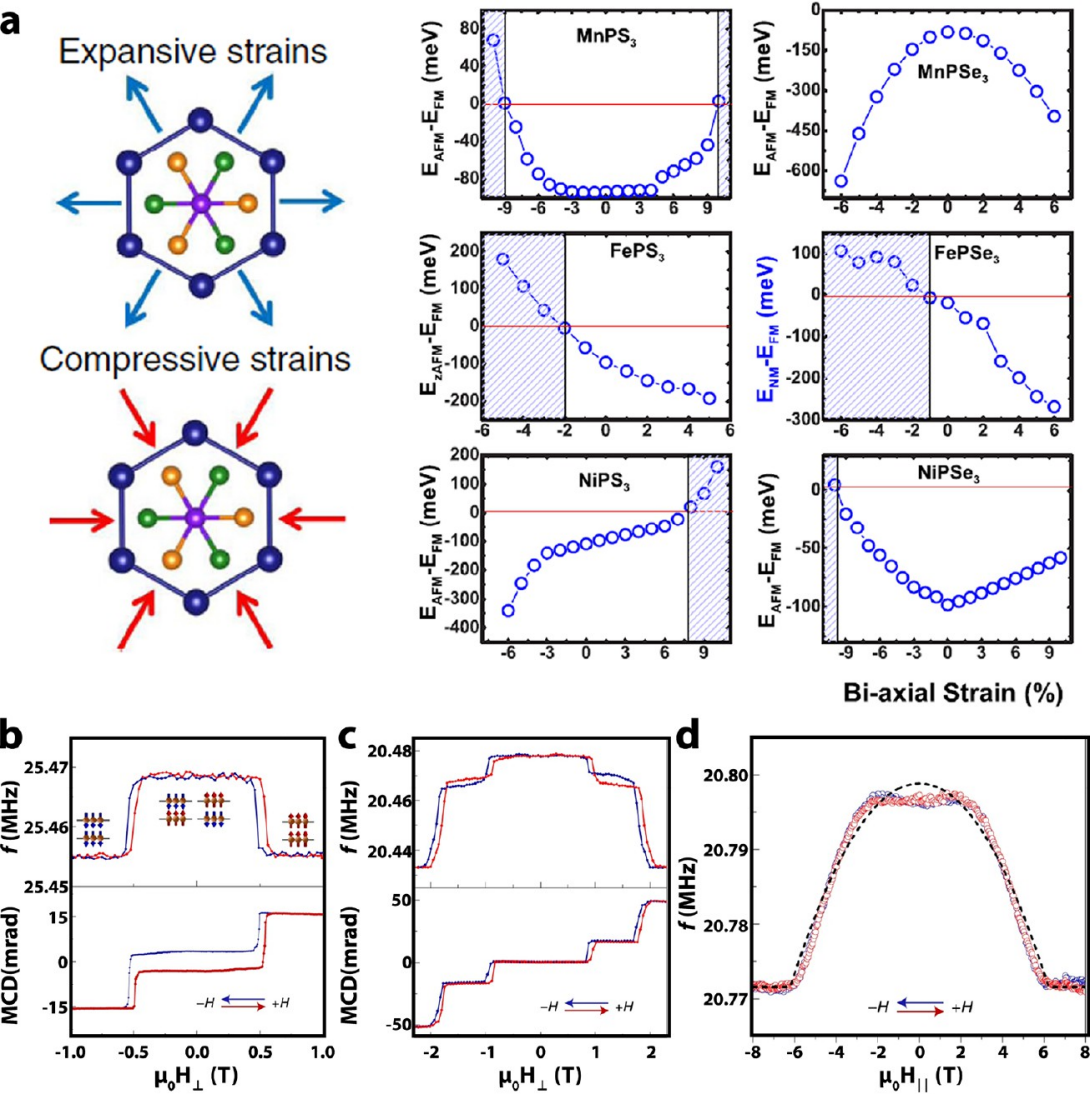
Mechanical properties of ternary compounds. (a) Changes in the magnetic configurations of various MPX_3_ (M = Mn, Fe, Ni; X = S, Se) compounds at zero carrier density under in-plane biaxial compressive and expansive strains. Adapted with permission from ref (684). Copyright 2016 American Physical Society. (b) Magnetostriction in 2L CrI_3_ and (c) 6L CrI_3_ resonators under an out-of-plane magnetic field (μ_0_*H*_⊥_) and in (d) 6L CrI_3_ resonator under an in-plane magnetic field (μ_0_*H*_∥_). The resonance frequency (b–d) and MCD (b, c) of the membranes as a function of the magnetic field, where the red (blue) lines correspond to the measurement for the positive (negative) sweeping direction of the field. Panels (b–d) are adapted with permission from ref (685). Copyright 2020 Springer Nature.

Although traditional magnetic thin films show good susceptibility to external strain^686,687^ for sensing applications,^688−690^ strain engineering of 2D magnets could be conducted at higher strains and may lead to intriguing phenomena. Tensile strain can change the coupling between local spins, Curie temperature, and transitions between FM and AF phases of 2D magnets.^234,330,657,666,667,677,678^ For example, the strength of the exchange coupling and spin polarization of VZ_2_ (Z = S, Se, Te) monolayers are able to be altered *via* strain modulation (−5% to 5%) due to the effect on the ionic–covalent interactions between V and Z atomic pairs, where the increased unpaired electrons in the interacting atoms change the magnetic moments.^691^ Similar phenomena are observed in semiconducting or insulating 2D transition-metal trichalcogenides (MYX_3_), and the strain required for magnetic phase change is chemical composition-dependent. For FePS_3_, FePSe_3_, and VPTe_3_ monolayers, just 1% of tensile strain is adequate to trigger phase transition according to theoretical studies (Figure 60a).^684^ In the case of CrPS_3_ and NiPS_3_, the FM–AF magnetic phase transition happens at strains greater than 4%; while larger strain of ∼9% is needed to observe such transition in VPSe_3_ and MnPS_3_.^684^ Another FM semiconductor, chromium telluride compounds with metalloids as the middle element, *i.e.* CrYTe_3_ (Y = Si, Sn, Ge) show enhanced ferromagnetism and significant changes in their Curie temperature under moderate tensile strains of ∼4–5%.^664,692−694^ 2D CrX_3_ (X = Cl, Br, I) also shows great potential in strain modulation under large strain,^695^ though it is not effective to modify their Curie temperatures.^658^ As shown by recent experiments, high-quality 2D CrX_3_ can sustain up to 6.0–6.5% strain without failure.^659^ Another experimentally strain-engineered 2D magnet is 1L FeSe, which could be stretched up to 5–6%.^696,697^ With their relatively low elasticity, 2D magnets should be highly sensitive to small stress variations. This great sensitivity to lattice deformation allows to experimentally detect one-step magnetization reversal, which has not been achieved in conventional magnetic materials.^698^ 2D magnets also provide good platforms to study magnetostriction at the atomic thickness. Mechanical resonators based on few-layer CrI_3_ showed changed resonant frequency under external magnetic field due to competition between minimizing the elastic energy and internal magnetic interactions (Figure 60b).^685^ Magnetostriction is useful in sensing and producing ultrasonic vibration or waves. In addition to in-plane strain, it has also been demonstrated that the magnetic ground state and interlayer magnetic coupling of atomically thin CrI_3_ can be altered by out-of-plane hydrostatic pressure.^22,23^ The transition of magnetic phase under hydrostatic pressure relates to the varied stacking order in CrI_3_, and the same effect may exist in other 2D magnets.^22^

### Challenges

There are still many research gaps and opportunities in the mechanical properties of 2D magnets, such as (1) The intrinsic mechanical properties of most 2D magnets are still waiting for experimental investigations despite of the theoretical predictions. With the well-established experimental techniques for measuring the mechanical properties of other 2D materials, the difficulty mainly lies on the fabrication (*e.g.*, by mechanical exfoliation) of suspended high-quality 2D magnets suitable for these tests. There could be additional technical challenges for dealing with 2D magnets that are not stable in air. (2) The large-scale synthesis of 2D magnets, such as by CVD and MBE are beneficial to their practical applications. However, based on the experience from other 2D materials, these 2D magnets could have lower than intrinsic mechanical strength and fracture strain due to the presence of large numbers of defects and grain boundaries, especially that the detrimental effect of vacancies on the strength of 2D materials is much more dramatic than that of bulk materials. Hence it is essential to study the mechanical properties of imperfect 2D magnets, which has not been theoretically and experimentally studied yet. (3) There are many exciting opportunities in the magneto-mechanical properties of 2D magnets. The aforementioned magnetostriction effect is one of them. Although the magnetostriction of CrI_3_ has been demonstrated, the magnetostrictive coefficient has not been quantified. Moreover, many applications of the magneto-mechanical coupling in 2D materials could be explored. The magneto-mechanical coupling in doping-enabled magnetism in intrinsically nonmagnetic 2D materials could be an interesting direction to explore as well.^382,699^ (4) Although the influence of strain on magnetic behaviors and other physical properties of 2D magnets has been extensively studied theoretically, there are just a few experimental studies so far. It is of particular interest to experimentally demonstrate one-step magnetization reversal in 2D magnets, as these effects are difficult to be achieved in conventional magnets because high tensile strain is normally required. The high strain susceptibility of the magnetic properties of 2D magnets offers great potential for various applications, such as in spintronics.

## Spin Excitations and Topological Properties

### Introduction

2D vdW magnetic materials have a long and distinct history.^42^ Compared with other 2D magnetic systems, including quasi-2D metallic magnetic materials, monolayer of magnetic ions on a substrate, monolayer organic molecules containing magnetic atoms deposited on a substrate *via* Langmuir–Blodgett technique, and 2D electron gases, one of the advantages of 2D vdW magnetic materials is that they can potentially be mechanically cleaved into a monolayer and therefore provide a platform to study fundamental physics without the influence of a substrate.^62,84,700^ Technologically, one can also develop spintronic devices with insulating thin 2D magnetic materials to avoid Ohmic heating.^62^ The field of 2D vdW magnetic materials took off a few years ago with the experimental demonstration of magnetic order in single atomic layer of several vdW 2D magnetic materials including FePS_3_,^32,701^ CrI_3_,^5^ Cr_2_Ge_2_Te_6_,^6^ VSe_2_,^18^ and MnSe_2_.^17^ While these developments are very exciting, they also call for a more detailed examination of the magnetic properties of these vdW materials, both in bulk form as well as with decreasing layer thickness.

For spin rotational invariant systems with short-range magnetic interactions describable by a Heisenberg Hamiltonian, it has been shown rigorously by Mermin and Wagner that there cannot be long-range FM or AF order at finite temperature in the 2D (monolayer) limit.^40^ This is because the continuous symmetry of the isotropic Heisenberg Hamiltonian leads to gapless long-wavelength (low-*Q*) spin waves that can be excited at any finite temperature, with detrimental effects on long-range magnetic order in low dimensions. However, when spin rotational invariance of the system is broken, say by anisotropic magnetic interactions in a 2D Ising model, long-range magnetic order can occur below a transition temperature when anisotropic interactions open a gap in the spin-wave spectrum and suppress the effect of thermal fluctuations.^33,34^ Finally, for the 2D XY model,^702^ there is no transition to a long-range magnetic order below a finite temperature where the magnetic susceptibility diverges. Instead, the diverging susceptibility is associated with the onset temperature of topological order, called Kosterlitz–Thouless temperature *T*_*KT*_ below which spin correlations are characterized by an algebraic decay and the bound pairs of vortex and antivortex spins.^64^ In addition to the 1D Ising,^33,34^ 2D XY,^702^ and 3D Heisenberg Hamiltonian,^703^ 2D honeycomb lattice magnetic materials with spin *S* = 1/2 can be described by a Kitaev Hamiltonian, which is an exactly solvable model that can realize many emergent phenomena such as Z_2_ gauge field, quantum spin liquid (QSL) states, and topological order.^704^

2D vdW magnetic materials provide a broader and flexible approach for studying 2D magnetism, particularly in our ability to test different forms of spin Hamiltonian’s described above.^84^ In bulk crystal form, they exhibit highly anisotropic, quasi-2D electronic properties that are often qualitatively different from common 3D materials.^705^ To understand 2D magnetism,^5^ one must first determine the magnetic exchange couplings of these materials. In their bulk form, this can be achieved by neutron scattering, while Raman and tunneling spectroscopy can be used to understand the magnetic properties of thin flakes. Within the past two decades, the possibility of isolating a single molecular layer from cleavage has further led to numerous discoveries of unexpected 2D physics, such as Dirac Fermions in graphene.^706,707^ Recently, the discovery of FM order in ultrathin vdW films attracted considerable attention, as the interlayer coupling is eliminated in these clean 2D systems. These vdW magnetic materials not only allow experimental tests of magnetic theories, but also enable nanoscale spintronic devices more efficient than current transistor-based electronics.^213^ An advantage of 2D vdW films for fundamental research is their large and clean surface, which allows the manipulation of electronic states by gating and by the formation of heterostructures.^708,709^ For example, electrostatic doping was shown to switch net magnetization in bilayer CrI_3_.^9,11^ Moiré engineering, the formation of tunable superlattices by two misaligned vdW monolayers, has led to strongly correlated electronic and superconducting phases that are intimately related to magnetism.^197,198,710^

Compared with 3D magnetic materials, 2D magnetic interactions and frustrations play an important role in exotic phenomena like QSL^711−714^ and high-temperature superconductivity.^715,716^ The key to observable 2D long-range magnetic order is the presence of the anisotropic spin-exchange interaction, as it opens a gap for the low-energy spin excitations that allow the development of long-range magnetic order at finite temperature. In vdW magnetic materials with honeycomb, triangular, square, and kagome lattice structures, all four fundamental spin Hamiltonians (the Ising, XY, Heisenberg, and Kitaev) can be realized.^84,211,665,717,718^ The ground state of the system is sensitive to superexchange interaction and interlayer coupling.^235^ In many materials, the ground state may switch depending on lattice parameters.^719^ Therefore, for both fundamental understanding and practical applications of 2D magnetism,^62,720^ it is critical to study and control the spin Hamiltonian in these materials. In many cases, the spin anisotropy that controls the magnetic order of the system is determined by crystal field and/or SOC. By carefully measuring the magnetic order and spin excitations spectra in bulk and atomically thin crystals, one can model spin Hamiltonians of the system.

Neutrons—with their wavelengths comparable to atomic spacing and energies close to those of spin excitations in solids—have played a role in determining the lattice and magnetic properties of bulk vdW magnetic materials. Some of the reasons for this role are as follows: (1) A neutron carries a magnetic moment that interacts with localized magnetic ions and unpaired itinerant electrons in solids. (2) A neutron is a weak interacting probe. A neutron scattering cross section is determined by the static and dynamic correlation functions of the system, without the need for correcting the influence of the probe. (3) Neutrons are a highly penetrating bulk probe and therefore insensitive to surface imperfections, and therefore an ideal probe to study bulk magnetic properties of 2D vdW magnetic materials. (4) Neutron polarization analysis can determine magnetic anisotropy of the system, and therefore provide a way to directly probe the SOC-induced magnetic anisotropy of spin excitations and magnetic ordered moment direction.

**Figure 61:**
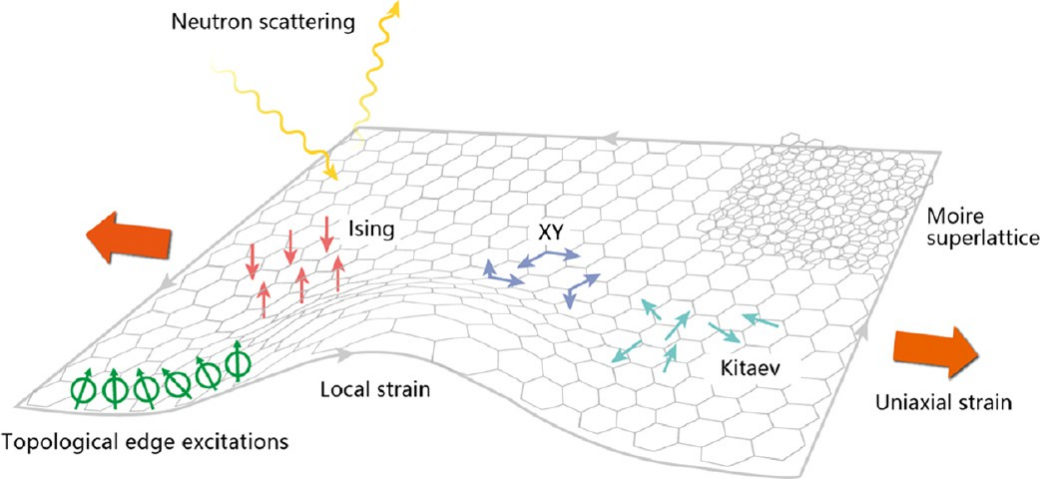
Magnetic order and spin excitations in vdW magnetic materials with tunable fundamental spin Hamiltonian’s and structural parameters probed by neutron scattering methods. Adapted with permission from ref (84). Copyright 2018 Springer Nature.

Despite its advantages, neutron scattering is limited to bulk samples. In few-layer 2D flakes with lateral dimensions on the micron scale, or in systems where the surface exhibits different behavior than the bulk, it is necessary to utilize alternative probes. The purpose of this section of the review is to first discuss recent progress on understanding bulk magnetism and spin dynamics in vdW magnetic materials obtained by neutron scattering and muon spin relaxation (Figure 61). We shall then describe how inelastic tunneling spectroscopy (IETS) and Raman spectroscopy can be used to probe for magnetic excitations down to the 2D limit as well as on the surface of bulk crystals. Finally, we will also discuss possible future directions in these areas.

### Neutron Scattering in Bulk vdW Magnets

**Figure 62:**
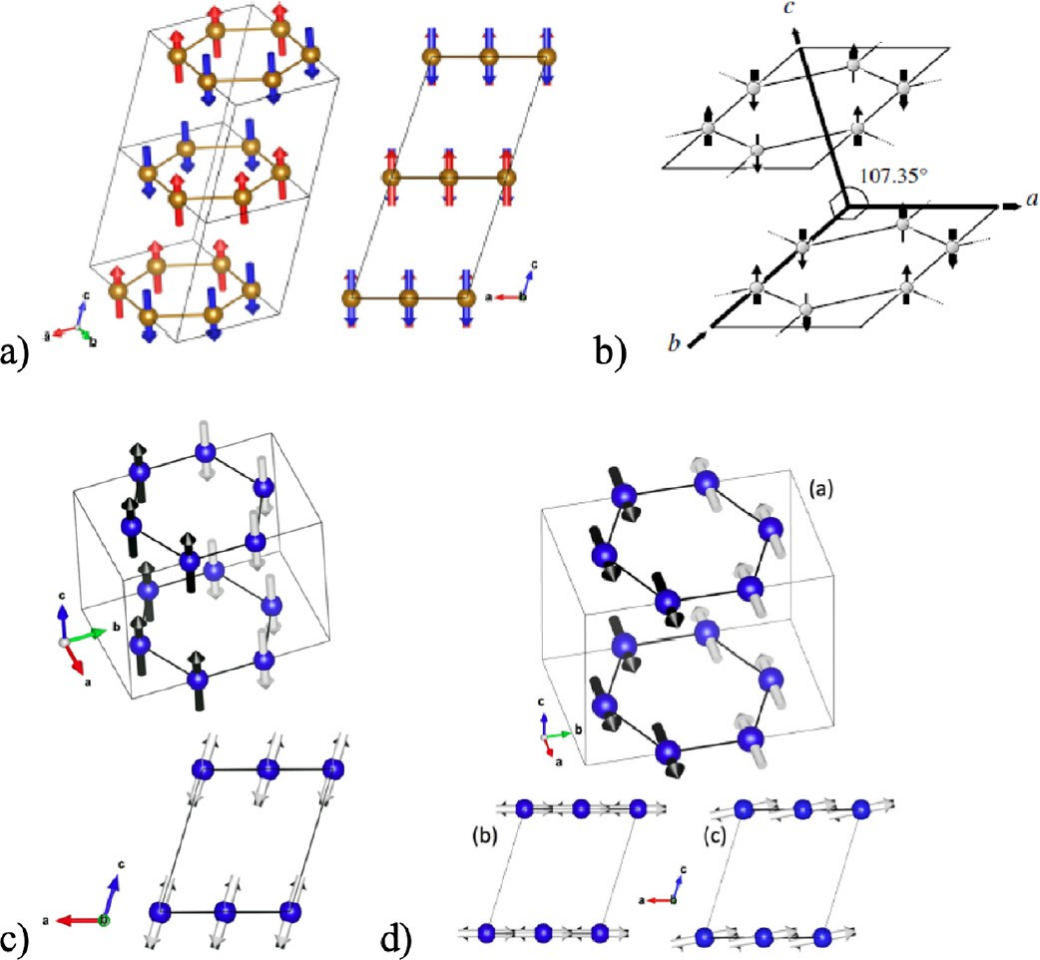
Magnetic structures of (a) FePS_3_.^724^ Adapted with permission from ref (724). Copyright 2016 American Physical Society. (b) MnPS_3_.^318^ adapted with permission from ref (318). Copyright 2006 American Physical Society. (c) NiPS_3_.^317^ adapted with permission from ref (317). Copyright 2015 American Physical Society. (d) CoPS_3_.^729^ adapted with permission from ref (729). Copyright 2017 IOP Publishing. All of these materials have honeycomb lattice structure and are antiferromagnetically ordered.

We begin by discussing layer transition metal (TM) thio(seleno) phosphate materials TMPS(Se)_3_ where TM = V, Cr, Mn, Fe, Co, Ni, Cu, Zn, Pd, Ag, and Cd.^721−723^ For a subset of these materials with magnetic transition metal ions, magnetism can be described by 2D Ising (FePS_3_),^32,724,725^ 2D Heisenberg (or possibly XY) (MnPS_3_),^318,726,727^ and 2D XY (NiPS_3_, CoPS_3_)^317,728,729^ Hamiltonian’s. While crystal structures of these materials are somewhat different, the transition metal magnetic ions all have a honeycomb lattice structure. Figure 62a–d shows magnetic structures of FePS_3_,^724^ MnPS3,^318^ NiPS_3_,^317^ CoPS_3_,^729^ respectively. In all cases, the systems order antiferromagnetically with different magnetic structures (Figure 62a–d). We first discuss FePS_3_, where the magnetic structure is collinear AF with moment perpendicular to the honeycomb lattice along the *c*-axis as shown in Figure 62a.^724^ There are three twinned domains rotated 120°, resulting in overlapping spin waves from different domains at the same reciprocal space point.^725^ Spin waves of FePS_3_ were measured by inelastic neutron scattering experiments and modeled *via* a Heisenberg Hamiltonian with single ion anisotropy and biquadratic exchange interaction.^725^ In most local moment systems, a Heisenberg Hamiltonian with single ion anisotropy should be able to describe spin waves. The biquadratic exchange interaction, originally proposed to explain spin waves in AF ordered parent compound of iron-based superconductors,^730,731^ is needed to understand spin wave deviations from Heisenberg Hamiltonian near the zone boundary.^725^ For AF MnPS_3_,^721^ neutron scattering experiments have studies magnetic order and spin waves of the system. Spin wave dispersions of MnPS_3_ can be well fitted by a Heisenberg Hamiltonian with first-, second-, and third-nearest in-plane neighbors magnetic exchange couplings.^726^ From neutron diffraction experiments, it was argued that the critical behavior of the material over most of the temperature range is more representative of an XY-like system instead of a classical Heisenberg system.^727^ For XY antiferromagnet NiPS_3_^317^ and CoPS_3_,^729^ their magnetic structures are rather similar except for the moment direction (Figure 62). Inelastic neutron scattering experiments were carried out on powder samples of NiPS_3_ and CoPS_3_ determined approximate magnetic exchange couplings.^728,732^ When NiPS_3_ was cleaved to monolayer, the AF order in bulk disappears consistent with the expectation of the XY magnetism in the atomically thin limit.^211^

Next we discuss magnetic order and spin excitations in honeycomb lattice vdW magnetic materials, including semiconducting CrGeTe_3_,^733^ metallic Fe_3_GeTe_2_,^323,333,734^ and semiconducting chromium trihalides CrX_3_ where X = Cl, Br, I.^735^ In the monolayer limit, long-range FM order persists in some of these materials. There has been a huge amount of recent work on monolayer or few layers of these materials.^62,700^ For semiconducting FM CrGeTe_3_ with ordered moment direction along the *c*-axis below the Curie temperature *T*_*C*_,^733^ critical scattering measurements indicate that FM phase transition is second order in nature.^279^ Unfortunately, there are no inelastic neutron scattering experiments to determine spin waves and magnetic exchange couplings. For metallic FM Fe_3_GeTe_2_, it was found that Curie temperature of the system depends sensitively on the stoichiometry of the iron with reduced TC for iron deficient samples.^323^ Inelastic neutron scattering measurements on Fe_2.75_GeTe_2_ synthesized from flux method mapped out the spin excitations spectrum and found a spin gap of 3.7 meV at the Γ point, providing information of the magnetic exchange interactions and anisotropy.^736^ However, because of the Fe deficiency and the corresponding disorder, the excitations are rather broad and damped, precluding a detailed modeling of its spin Hamiltonian.

In the case of FM chromium trihalides with honeycomb lattice structure,^735^ while the role of the underlying structure in stabilizing the 2D ferromagnetism is under intense investigation,^120,235^ the honeycomb structure provides another interesting physics of massless magnetic Dirac particle analogous to the massless electrons near Dirac points in graphene.^737−739^ In this picture, magnons become massless at the Dirac K/K′ points with linear dispersions due to the exchange frustrations between two sublattices of the honeycomb lattice. The presence of these Dirac points are robust against finite next-nearest-neighbor exchanges, which will only shift positions of the Dirac points. The linear spin wave bands across the Dirac points have been experimentally observed in 2D ferromagnets CrBr_3_^124^ and Cr_2_Si_2_Te_6_.^740^ Similar magnon band crossings have also been observed in the 3D antiferromagnet Cu_3_TeO_6_.^741,742^ If the SOC induced antisymmetric DMI exists between the next-nearest neighbors in honeycomb lattice, a gap will appear in the spin wave spectra of the system at the Dirac K/K′ points, causing the magnon bands to become topological.^743^ For CrBr_3_, inelastic neutron scattering experiments found no evidence of a gap at the Dirac point,^124^ although these measurements were taken in 1971 and additional neutron time-of-flight measurements using spallation neutron source would be desirable. These results, if confirmed, would suggest that the SOC-induced DMI in CrBr_3_ is insufficient to induce topological spin excitations.

**Figure 63:**
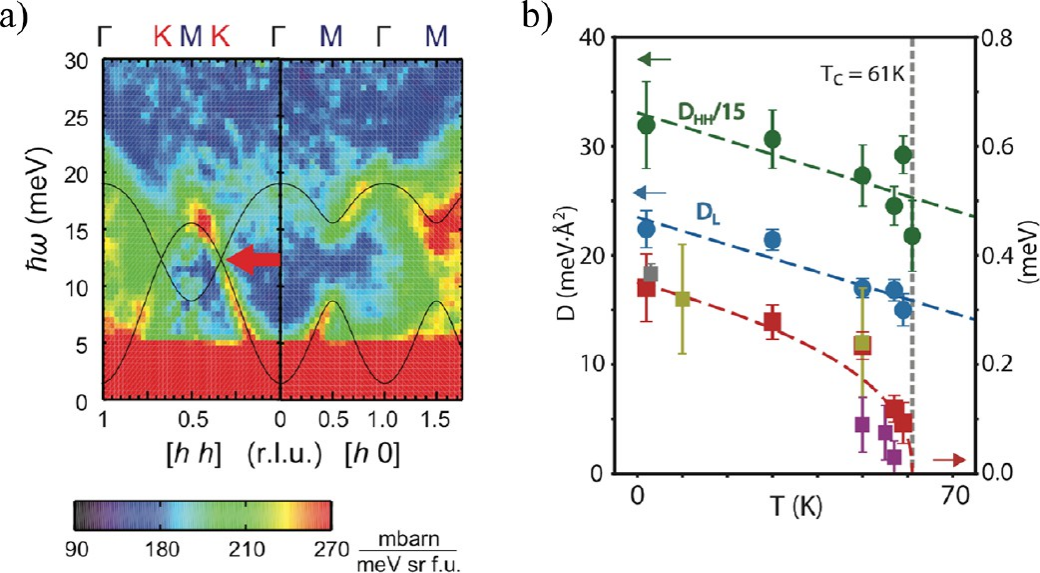
(a) A spin gap at Dirac point in CrI_3_ suggests that spin excitations in this system can have chiral and topological edge mode. Adapted with permission under a Creative Commons CC BY 4.0 license from ref (744). Copyright 2018 American Physical Society. (b) The FM phase transition in CrI_3_ is weakly first-order and controlled by spin gap. Adapted with permission from ref (745). Copyright 2020 American Physical Society.

In the case of CrI_3_, which has larger SOC compared with CrBr_3_, neutron time-of-flight measurements of the magnon bands indeed reveal a spin gap at the Dirac points as shown in Figure 63.^744^ Although these results are interesting, the next-nearest neighbor DMI induced gap may not be the only interpretation of the data as Heisenberg–Kitaev Hamiltonian may also account for the observed spin gap near the Dirac points.^746,747^ In addition, the FM phase transition in CrI_3_ was found to be a weakly first order instead of second order in nature, and controlled by SOC instead of magnetic exchange couplings as in a conventional Heisenberg ferromagnet.^748^ Inelastic neutron scattering on different families of FM honeycomb lattice vdW materials should be able to test whether the microscopic origin of the observed 2D FM order is due to SOC-induced magnetic anisotropy,^120,235^ and determine if Heisenberg–Kitaev Hamiltonian is an appropriate description of the spin dynamical behavior in honeycomb lattice ferromagnets. By systematically tuning the strength of SOC in chromium trihalides,^586,749^ one can test if the observed spin-gap arises from SOC-induced DM effect or Kitaev interaction.^746,747^ Indeed, recently neutron scattering measurements suggest that the observed spin Dirac gap in CrI_3_ is induced by the next nearest neighbor DMIs.^750,751^

**Figure 64:**
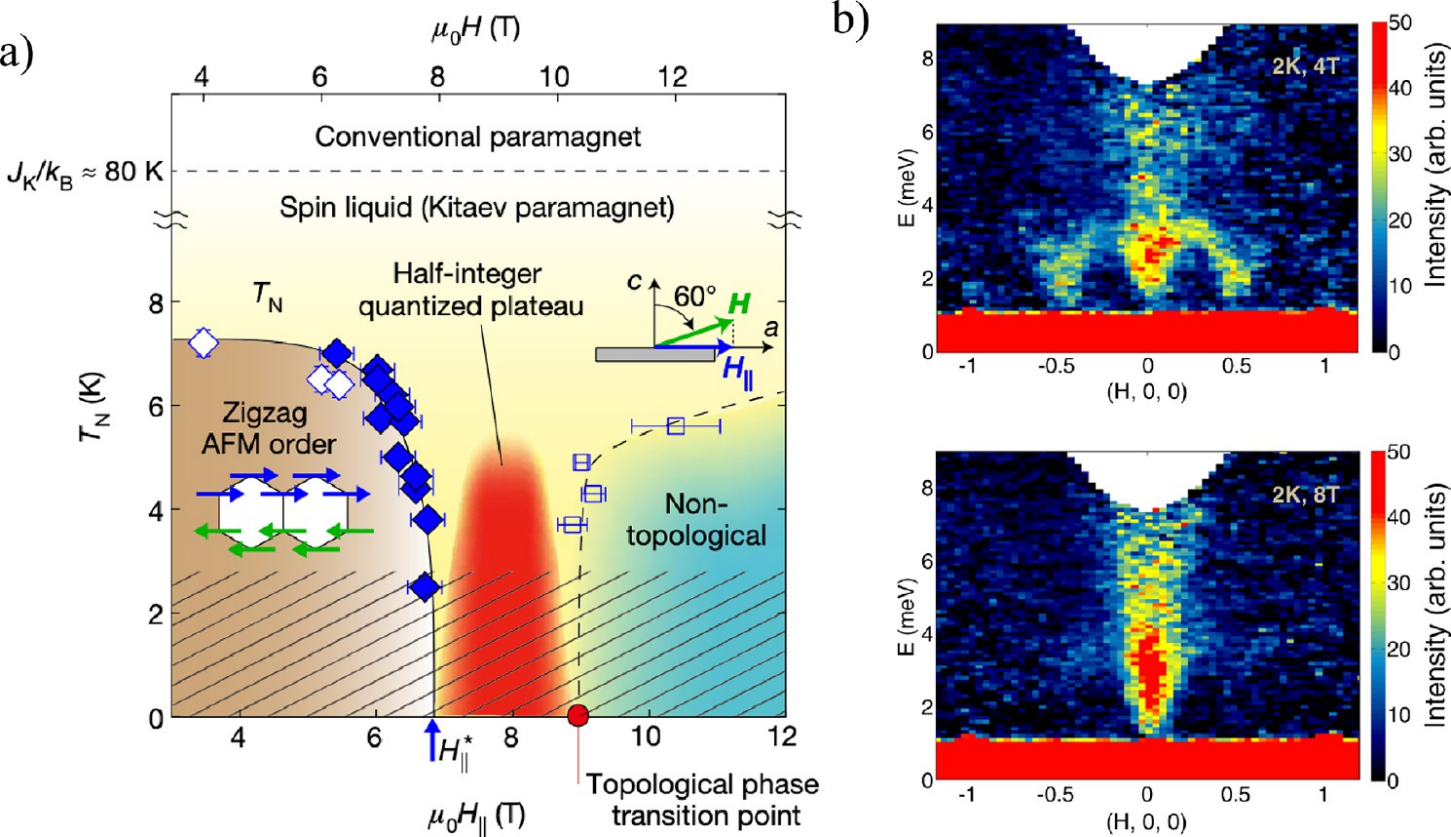
(a) The phase diagram of α-RuCl_3_ adapted with permission from ref (752). Copyright 2018 Springer Nature. At zero field, the system forms a zigzag magnetic structure as shown in the left inset. For in-plane magnetic field between 7 and 9 T, the system is believed to be in Kitaev QSL state. For fields above 9 T, the system becomes non topological from thermal transport measurements. (b) Wave vector/energy dependence of spin excitation of α-RuCl_3_ at 4 and 8 T, adapted with permission under a Creative Commons CC BY 4.0 from ref (753). Copyright 2018 Springer Nature. One can clearly see spin waves stemming from zigzag ordered wave vector (0.5, 0, 0) at 4 T. The scattering centered around Γ points is believed to arise from fractionalized excitations of a Kitaev QSL, which is enhanced upon suppression of spin waves with a 8 T in-plane magnetic field.

In addition to AF and FM order, geometric magnetic frustration in 2D materials can host a QSL phase, in which the spins of unpaired electrons in a solid are quantum entangled but do not show magnetic order in the zero-temperature limit.^712−714^ Because such a state may be important to the microscopic origin of high-*T*_*c*_ superconductivity^716^ and useful for quantum computation,^704,754^ experimental realization of QSL is a long-sought goal in modern condensed matter physics. Models supporting the existence of QSLs in 2D spin-1/2 kagome, triangular, honeycomb, and 3D pyrochlore lattice systems indicate that all QSLs share deconfined spinons, elementary excitations from the entangled ground state which carry spin *S* = 1/2 and thus are fractionalized quasiparticles, fundamentally different from the *S* = 1 spin waves in conventional 3D ordered magnets.^714^ In particular, honeycomb lattice magnetic materials are of interest because a QSL can arise from the exactly solvable Kitaev model with *S* = 1/2 Ising spins on a honeycomb lattice.^704^ Over the past several years, honeycomb lattice magnetic materials such as A_2_IrO_3_ (A = Cu, Li, or Na)^755−758^ and α-RuCl_3_^718,753,759,760^ have made considerable impact on the community and provided constraints on testing the Kitaev’s QSL Hamiltonian. However, all honeycomb lattice materials investigated so far have symmetric off-diagonal exchange interactions beyond Kitaev’s Hamiltonian (referred as the Γ term),^747,761−764^ and therefore induce long-range magnetic order. In the case of α-RuCl_3_, Ru spins order in zigzag AF structure with moment in the plane (see inset of Figure 64a).^760^ Upon application of an in-plane magnetic field above 7 T, the long-range magnetic order is suppressed and the system is believed to reach a field-induced Kitaev QSL state with half-integer thermal hall conductance plateau.^752^ Inelastic neutron scattering experiments reveal spin waves from the zigzag order coexisting a continuum of magnetic scattering center around the Γ point (Figure 64b). When an in-plane magnetic field of 8 T is applied, spin waves from the zigzag order are suppressed and the continuum of magnetic scattering, possibly arising from fractionalized quasiparticles of a Kitaev QSL, is still present (Figure 64c).^753^ In addition to neutron scattering experiments, recent resonant elastic X-ray scattering experiments on a single crystal α-RuCl_3_ using the Ru L_2_ and L_3_ edges (2838 and 2967 eV) established the Hamiltonian of the system, revealing that the Kitaev interaction is FM and the Γ term is AF comparable in size as the Kitaev interaction.^765^

**Figure 65:**
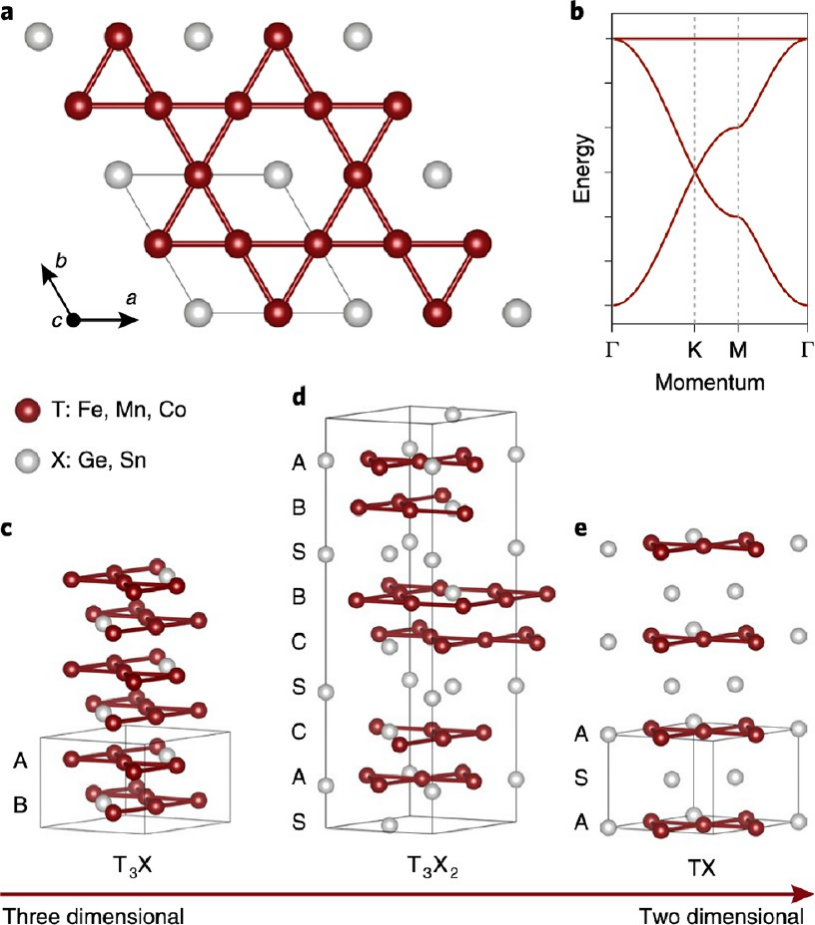
(a) The 2D kagome lattice structure. (b) Calculated electronic dispersion and flat band. (c, d, e) Crystal structures of T_3_X, T_3_X_2_, and TX, respectively, where *T* = Fe, Mn, Co, and X = Ge, Sn. All panels are adapted with permission from ref (766). Copyright 2019 Springer Nature.

**Figure 66:**
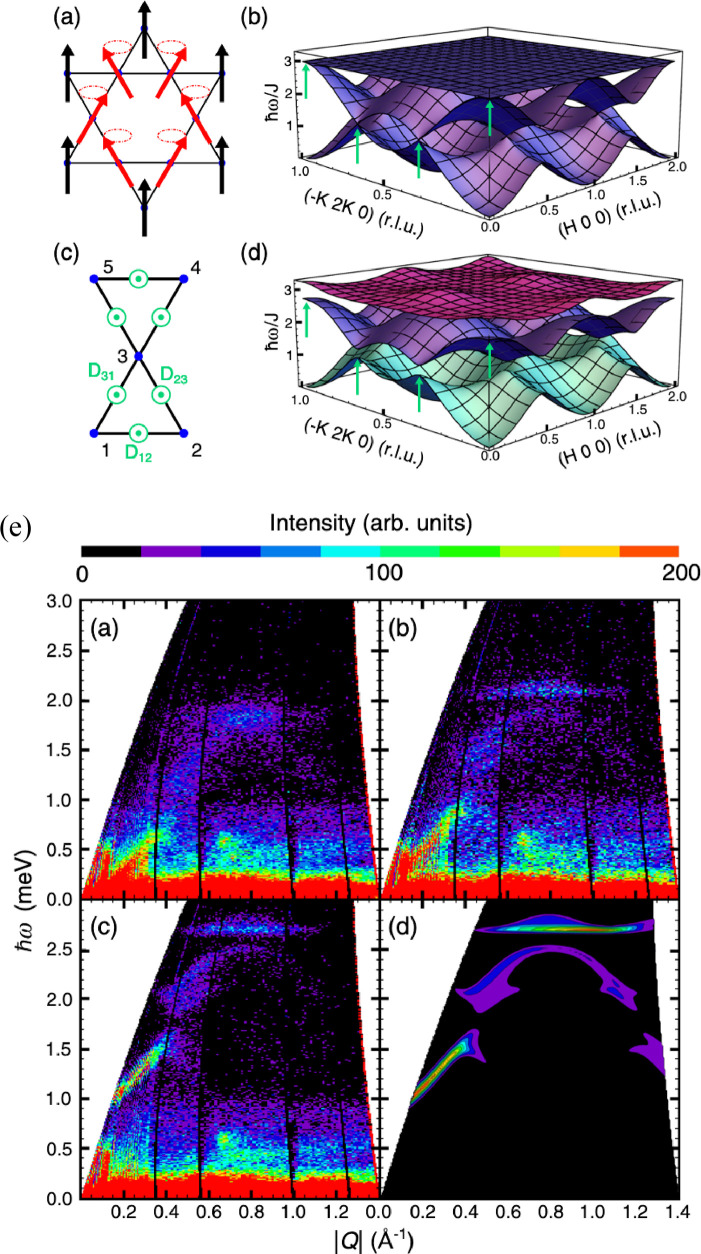
(a) Red arrows indicate in-plane spin directions at zero field. Black arrows indicate the moment direction in a *c*-axis aligned field. (b) Spin-wave branches for one acoustic and two optical bands. The middle and top green arrows indicate Dirac points and band top, respectively. (c) Nearest-neighbor DMI direction. (d) DMI induced spin gap at Dirac points. (e) Neutron scattering measured spin waves energy/moment map at (a) 0 T, (b) 2 T, (c) 7 T; (d) Calculated neutron spectra for 7 T. All panels are adapted with permission from ref (767). Copyright 2015 American Physical Society.

In addition to honeycomb lattices, 2D kagome lattices with arrangements of corner-sharing triangles and hexagons are incredible models with which to study magnetic frustration, electronic correlation, and topological electronic structures (Figure 65).^530,767−771^ Their competing spin interactions largely impede the development of a long-range magnetic order, frequently leading to the emergence of complex magnetic structures, interesting magnetic behaviors, and exotic states.^772,773^ For example, magnetic frustration in kagome lattice can lead to a QSL ground state.^530^ A kagome lattice ferromagnet can have topological magnon bands,^767,768^ leading to possible topological magnetic edge states.^529^ Topologically robust magnetic edge states are appealing for low-energy spending, fast spintronic devices, which would revolutionize modern-day technologies in applications such as sensing, information, and communication.^774,775^ It is predicted, taking SOC into account, that the kagome lattice can realize a 2D Chern insulating phase with quantized anomalous Hall conductance at 1/3 and 2/3 fillings.^776^ For real materials with three dimensions, the finite inter layer interaction may drive the mass gap to be closed and reopen along the *c*-axis, which is the case of 3D Weyl semimetal with broken time-reversal symmetry.^777^ Furthermore, the flat band also carries a finite Chern number that mimics the phenomenology of LLs, which further enrich the phase diagram that can be achieved in kagome lattices. Recently, ARPES experiments on kagome lattice antiferromagnet FeSn revealed the presence of an extremely flat electronic band about 0.23 eV below the Fermi level.^766^ Although a flat electronic band is expected in the single-orbital nearest-neighbor kagome model (Figure 66b), the microscopic origin of the observed flat band in FeSn remains a mystery.^526^ Since the presence of a flat band is extremely important to understanding the transport, electronic, magnetic, and superconducting properties of 2D magnetic vdW materials,^84,778^ it will be important to sort out structural and magnetic properties of kagome lattice materials.

**Figure 67:**
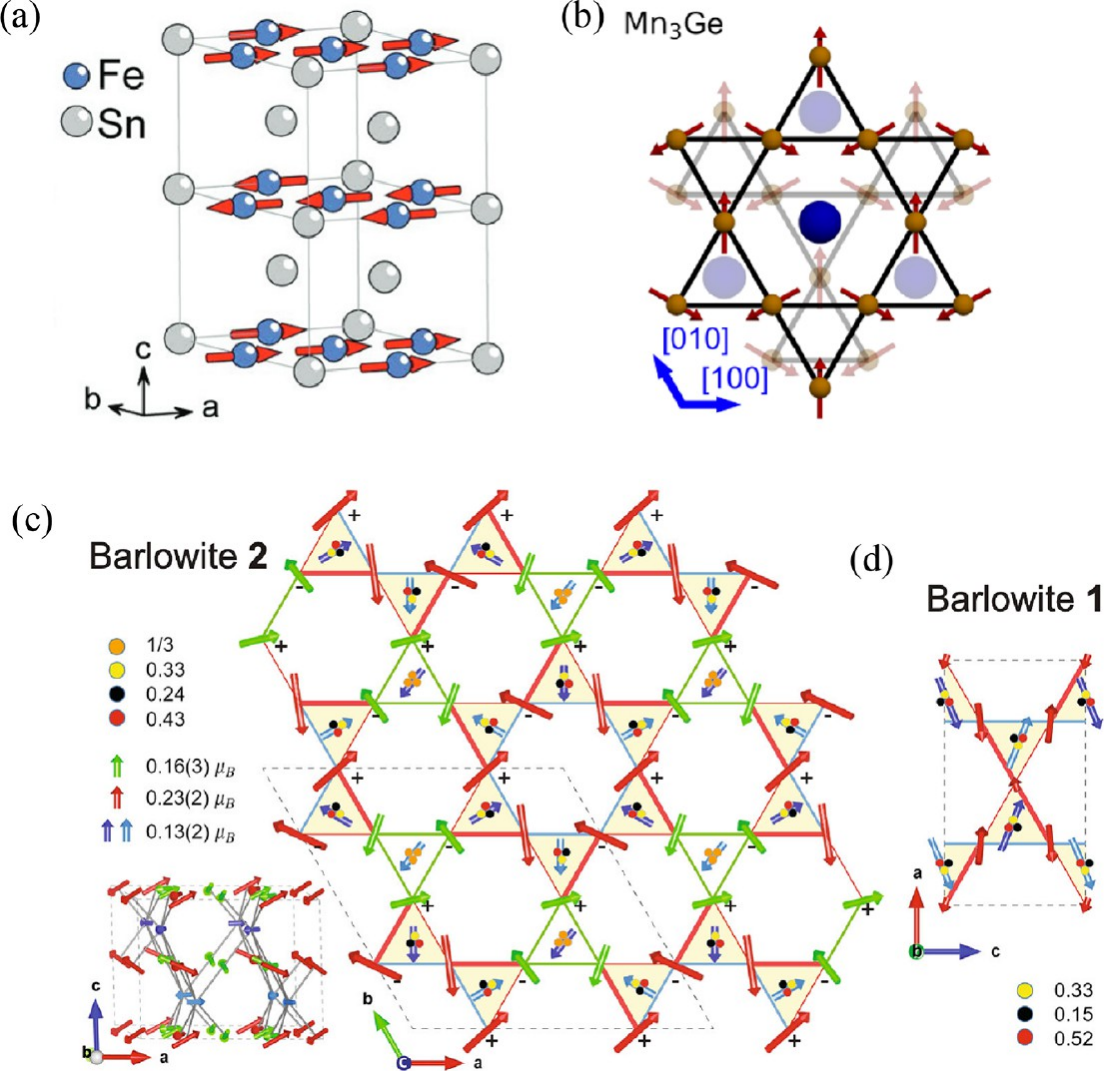
(a) A-type AF structure of FeSn in 2D kagome lattice structure, adapted with permission from ref (779). Copyright 2019 American Physical Society. (b) The antichiral noncollinear structure of Mn_3_Ge, adapted with permission from ref (673). Copyright 2020 American Physical Society. (c, d) Complicated noncollinear magnetic structures of barlowite with proximate 2D kagome lattice structure, adapted with permission under a Creative Commons CC BY 4.0 license from ref (780). Copyright 2020 Springer Nature.

In the case of an insulating kagome lattice ferromagnet Cu(1,3-bcd), the FM moment direction is in the kagome lattice plane without external applied magnetic field (Figure 66a).^767^ Because there are three magnetic atoms per unit cell in a kagome lattice, one expects three spin wave branches including one acoustic and two optical modes with one of the optical band being flat in momentum space due to the geometry of the kagome lattice (Figure 66b). Spin waves from acoustic and first optical branches meet at the Dirac points as shown by the green arrows of Figure 66b. At zero field, there is no nearest-neighbor DMI since the ordered moment is in the plane. When a 7 T *c*-axis aligned magnetic field is applied, the moment is tuned along the *c*-axis, giving rise to finite nearest neighbor DMI that opens a gap at the Dirac points and induces topological spin excitations (Figure 66e(a–d)].^767,768^ It will certainly be interesting to carry out neutron scattering experiments on 2D kagome lattice magnetic materials such as T_3_X, T_3_X_2_, and TX where T= Fe, Mn, Co, and X = Ge, Sn. For example, FeSn is a metallic A-type antiferromagnet with interesting properties (Figure 67a).^779^ On the other hand, Mn3Ge are semimetallic antiferromagnets where Mn atoms form antichiral noncollinear spin structure [Figure 67(b)].^745^ For distorted kagome lattice magnets such as crystalline barlowite (Cu_4_(OH)_6_FBr), magnetic structures of the systems can be rather complicated as shown in Figure 67c, depending on the details of the lattice distortion.^780^ Another interesting kagome lattice magnet is YMn_6_Sn_6_, which exhibits helical spin structure at zero external magnetic field.^781,782^ Recently, it was found that application of an in-plane magnetic field can induce a topological Hall effect (THE) near room temperature,^783^ which is typically associated with a magnetic field-induced skyrmion lattice or noncollinear spin texture with nonzero scalar spin chirality that can induce nonzero Berry curvature acting as fictitious magnetic field for the conduction electrons to give rise to the THE.^475,784−789^ Neutron diffraction experiments reveal that an in-plane magnetic field can actually change the helical spin structure into a double fan spin structure with *c*-axis components,^527^ different from the skyrmion lattice^786^ and inhomogeneous magnetic domain formation.^790^

As we can see from these examples, magnetic structures of kagome lattice magnets can be quite different depending on details of the magnetic exchange interactions and subtle lattice distortions. Clearly, future inelastic neutron scattering experiments to sort out spin excitations in these materials are critical to understand their exotic magnetic interactions and resulting anomalous topological and transport properties.

### Tunneling and Raman Spectroscopy of 2D Magnetic Layers and Surfaces

While neutron scattering can determine the full magnon dispersion relation in magnetic materials across the Brillouin zone, the technique is limited to bulk crystals. Below, we shall describe how IETS and Raman spectroscopy can be used to observe magnon behavior in layered magnets down to the 2D limit, as well as on the surfaces of bulk crystals. We shall focus on recent progress in the chromium trihalide family (CrX_3_, X = I, Cl, Br), although both techniques can be in principle generalized to other 2D magnets. By comparing with spin wave calculations for the honeycomb lattice, a simple spin Hamiltonian can be extracted for all three 2D systems, yielding information on the nearest neighbor exchange energy and anisotropy. In particular, strong easy-axis anisotropy in the direction perpendicular to the layers assists in stabilizing 2D ferromagnetism in monolayers. The surface layers of bulk CrI_3_ are further shown to host different magnetic order than those in the interior.

**Figure 68:**
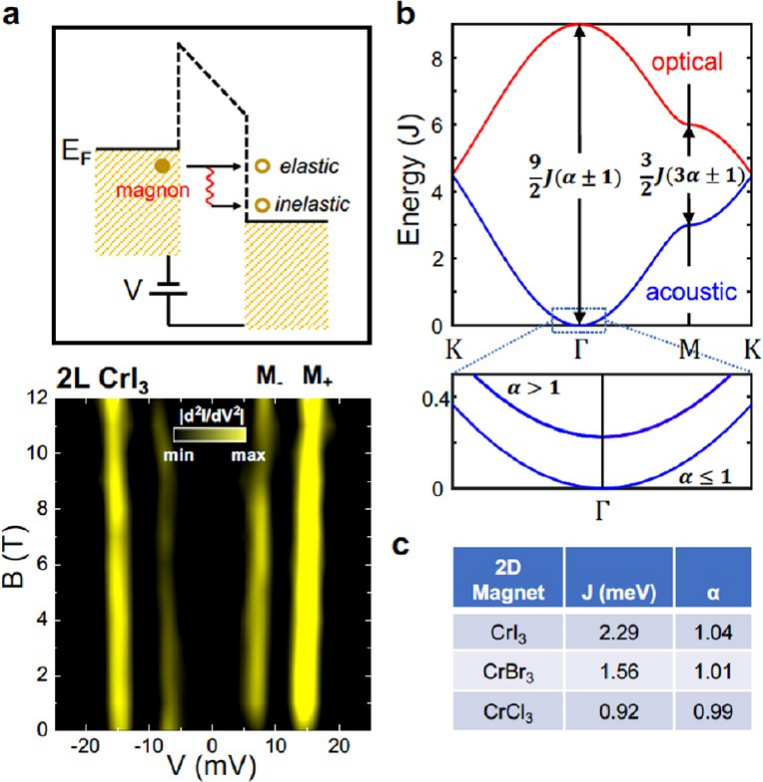
(a) Top: Schematic illustrating IETS mechanism. Bottom: Color plot of IETS spectra (|(*d*^2^*I*)/(*dV*^2^)| versus *V*) taken on bilayer CrI_3_ as a function of out-of-plane magnetic field (easy axis) shows two dispersing magnon modes. Bottom panel adapted with permission from ref (120). Copyright 2019 National Academy of Sciences of the United States of America. (b) Top: Spin-wave calculations of magnon dispersion in 2D CrI_3_ from anisotropic Heisenberg model with nearest-neighbor interactions. Adapted with permission from 217. Copyright 2018 Springer Nature. Magnon energies are shown at high-symmetry Γ and M points as a function of nearest-neighbor exchange energy *J* and anisotropy α. Zoom-in of the acoustic branch at the Γ point shows an energy gap for α > 1. (c) Extracted *J* and α values for all three 2D chromium trihalides from IETS measurements.

Electron tunneling is an old and established spectroscopic technique. The voltage *V* applied between two metal electrodes separated by a thin insulating barrier directly relates to the maximum energy of the tunneling electrons. While the overall current *I* is largely dominated by electrons that tunnel across the barrier elastically, when *eV* reaches the energy of an excitation within the barrier or at the interface, such as a phonon or magnon for magnetic insulators, electrons can lose energy to this excitation and tunnel inelastically. These two tunneling processes are shown schematically in the upper panel of Figure 68a. In particular, inelastic tunneling events open a secondary conduction channel and can be seen as either abrupt jumps in the differential conductance *dI*/*dV* at various *V*, or peaks in (*d*^2^*I*)/(*dV*^2^).^791^ Magnon excitations can often be distinguished from phonons by examining the evolution of the jumps or peaks with applied magnetic field.^792^

One key advantage of the IETS technique is that the lateral size of the tunnel junction can be reduced to submicron dimensions using nanofabrication or, more recently, mechanical transfer techniques, making it a more localized probe of magnon behavior. Mechanical transfer is particularly suitable for exploring 2D magnets that are sensitive to air as vdW heterostructures can be fully assembled in inert gloveboxes.^580^ One drawback of IETS is that the tunneling process, unlike optical spectroscopy, does not obey rigorous selection rules. It therefore is not immediately apparent which momentum values correspond to the observed magnon energies, although positions with higher density of states are likely to contribute more strongly.^126^ As such, close comparison with spin wave theory is needed to obtain quantitative exchange parameters.

Recently, several groups have performed IETS on graphene tunnel junctions incorporating ultrathin layers of magnetic insulators CrX_3_.^14,120,131^ A schematic of the common device geometry is shown in Figure 71F. The lower panel of Figure 68a shows |(*d*^2^*I*)/(*dV*^2^)| versus *V* taken for bilayer CrI_3_ as a function of magnetic field applied along the easy axis (normal to the layers). The spectra are shown as a 2D color plot for clarity. As features at positive *V* are reproduced at negative *V*, only two modes are visible. The peaks shift toward higher energy with increasing magnetic field by the Zeeman energy with *g*-factor ∼2, indicating that they arise from magnons. Qualitatively similar features have also been observed for bilayer CrBr_3_ and CrCl_3_, although the peak positions are different due to different magnetic exchange parameters between the three materials.^120^

The magnon energies can be compared with linear spin wave theory for the 2D anisotropic Heisenberg model, whose Hamiltonian at zero magnetic field can be written as , where *J* > 0 is the FM exchange coupling between spin components  and  on nearest-neighboring sites, and α is the exchange anisotropy along the out-of-plane *z* direction. The calculated magnon dispersion is plotted in the upper panel of Figure 68b for α = 1, which shows an acoustic (optical) branch at lower (higher) energy, crossing at the K point due to inversion symmetry of the underlying honeycomb lattice. The M point corresponds to a van Hove singularity with high density of states, and so are most likely to be seen in IETS, while photons can couple to Γ point excitations with zero momentum. For an out-of-plane easy axis, as in the case for CrI_3_, the energies at these specific momenta are  and .^120^ In particular, for α > 1, there is a gap for magnon excitations, as shown in the lower panel of Figure 68b. Identifying the momentum positions for the IETS peaks and matching with the magnon dispersion thus allows for the exchange energy *J* and anisotropy α to be determined for 2D CrX_3_.

Based on full spin wave calculations with changing magnetic field, the strongest |(*d*^2^*I*)/(*dV*^2^)| peaks were assigned to be near the *M* point for CrI_3_. The extracted *J* and α values for all three 2D CrX_3_ are shown in the table in Figure 68c. Both quantities increase from CrCl_3_ to CrBr_3_ to CrI_3_. The evolution of *J* is consistent with that of the critical temperatures (*T*_*c*_ values) in the three bulk compounds.^735^ Upon reducing sample thickness from few layers to monolayer, *T*_*c*_ in CrBr_3_ is seen to decrease by 10 K,^120^ while *T*_*c*_ in CrI_3_ decreases only by 1 K.^5,16^ This can be attributed to the larger α in CrI_3_, which increases the magnon excitation gap and helps to stabilize ferromagnetism in the 2D limit.

**Figure 69:**
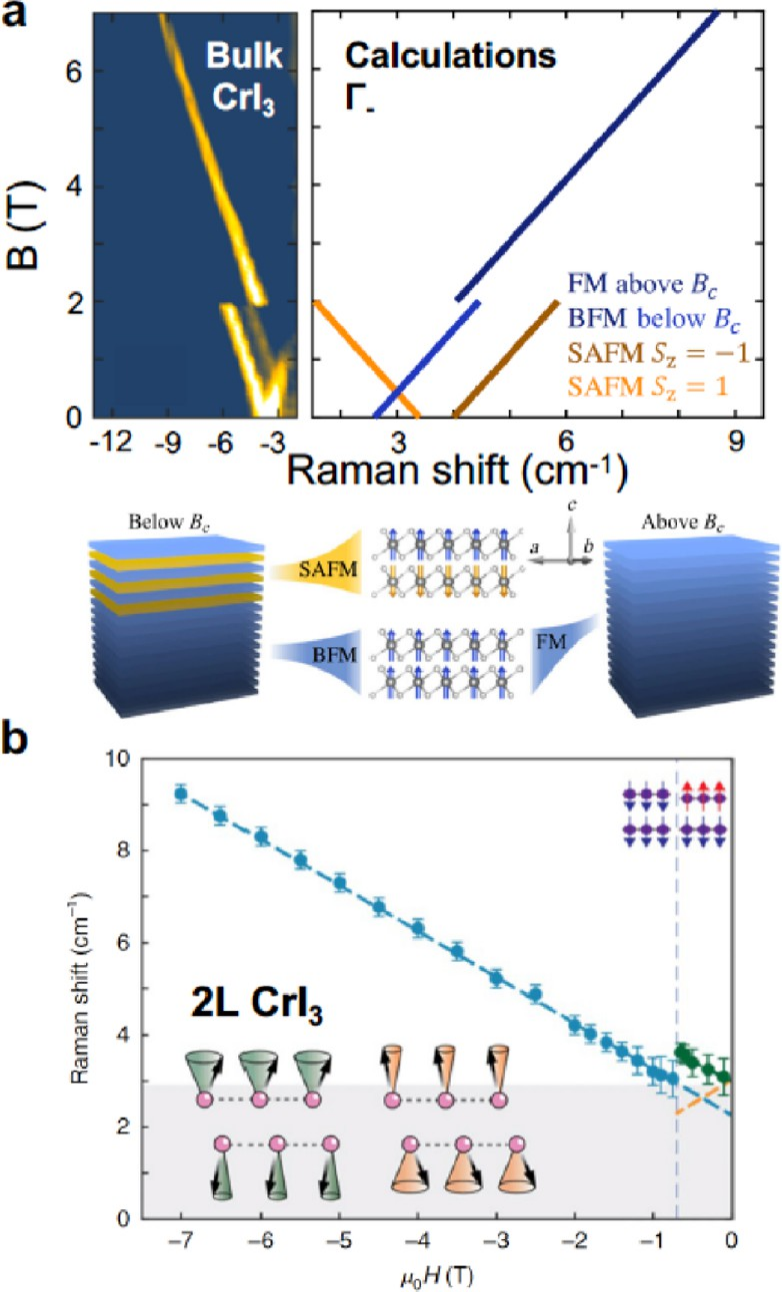
(a) Top: Experimental Raman spectra (left) and theoretical calculations (right) of magnon modes in bulk CrI_3_ as a function of out-of-plane magnetic field (easy axis). Above *B*_*c*_ ∼ 2 T, a single mode is observed as expected for a fully spin-polarized state for all layers. Below *B*_*c*_, two additional modes are seen that disperse oppositely with field, corresponding to the layer-AF order on surface layers. Bottom: Schematic of spins in bulk CrI_3_ above and below *B*_*c*_. Adapted with permission under a Creative Commons CC BY 4.0 license from ref (218). Copyright 2020 American Physical Society. (b) Field-dependent Raman spectra of bilayer CrI_3_. Only layer-AF magnons modes are obtained. Adapted with permission from ref (794). Copyright 2020 Springer Nature.

While IETS is able to detect magnon modes at momenta with high density of states in CrX_3_, Raman spectroscopy specifically selects for zero-momentum excitations at the Γ point. The technique is most commonly used to probe for higher-energy phonon excitations; however, recent state-of-the-art methods have allowed modes to be resolved within a few wavenumber (or sub-meV) from the laser line.^218^ The upper left panel of Figure 69a shows ultralow energy Raman spectra taken on bulk CrI_3_ as a function of magnetic field. Negative wavenumbers correspond to anti-Stokes scattering.

Above a critical out-of-plane magnetic field of *B*_*c*_ ∼ 2 T, a single peak is observed which blueshifts with increasing magnetic field by the Zeeman energy. This mode corresponds to the Γ point acoustic magnon in spin-polarized layers (layer-FM state), and extrapolation to zero field yields an energy of ∼2.5 cm^–1^ = 0.3 meV, close to that expected from the exchange parameters extracted from IETS. Below the critical field, this mode can still be seen, although there is a small discontinuity at the transition field. Interestingly, two additional modes appear in this region, which disperse in opposite directions with increasing field. These modes correspond to Γ point acoustic magnons in the layer-AF state—layers with spin direction aligned (antialigned) with the magnetic field yield increasing (decreasing) magnon energy with increasing magnetic field. Overall, these features compare well with the theoretical calculations shown in the upper right panel of Figure 69a.

Previously, bulk CrI_3_ was understood to be a ferromagnet,^227,735^ while ultrathin layers host a layered-AF ground state.^5,16^ These results show that even bulk CrI_3_ exhibits a mixed-phase structure with AF layers likely residing on the surface and FM layers in the interior (see the schematic shown in the lower panel of Figure 69a). Applying a field above *B*_*c*_ polarizes all of the spins, rendering a layered-FM state for both surface and bulk layers. In accordance with this picture, Raman spectra taken on ultrathin CrI_3_ (down to bilayer) only show Raman modes corresponding to the layered-AF state, as surface layers constitute the entire sample (see Figure 69b).^218,794^

These results highlight the importance of using microscopic probes to investigate magnetic excitations and interactions in 2D samples, as atomically thin samples or surfaces may exhibit different phenomena than their bulk counterparts. While we have focused on IETS and Raman measurements in this section of the review, we note that other techniques such as microwave absorption spectroscopy and pump–probe Kerr rotation have also been used to resolve Γ point magnons in CrX_3_.^28,795^ The latter study on bilayer CrI_3_ has further shown that the magnon frequency can be substantially tuned using an external gate, an opportunity unavailable for bulk crystals.

### Future Challenges

While the field of vdW magnetism has seen immense progress in the past few years, there are still many hurdles to be overcome. A comprehensive investigation of magnetic excitations in atomically thin samples is especially challenging from a technical perspective. Currently, neutron scattering is one of the few techniques capable of determining the energy of magnons as a function of momentum. However, it is inherently a bulk probe and requires large crystals. On the other hand, while IETS, Raman, and other optical techniques can be applied to monolayers, they can only resolve magnons at select momenta, making it difficult to extract higher-order exchange terms in the spin Hamiltonian, such as the biquadratic exchange predicted for CrX_3_.^796^ This is particularly a concern if magnetic properties of thin samples or surfaces deviate from their bulk counterparts, in which case neutron scattering may no longer serve as an accurate reference.

In order to resolve the full magnon dispersion in magnetic insulator monolayers, it may be possible to use graphene with a well-defined relative twist angle as tunneling contacts. By systematically varying the twist angle, one may potentially be able to select for the momentum of the magnons that couple to electrons tunneling between the K points of the two graphene layers. In prior works that observe magnons in CrX_3_ using IETS,^14,120,131^ the relative twist angle of the graphene/graphite contacts have not been carefully controlled, which may partially explain the slightly different IETS peak positions observed across different devices incorporating the same magnetic insulator.

Finally, both IETS and Raman scattering have limitations in the energy range that can be accessed. Tunneling devices break down under large electric fields, and while arbitrarily small voltages can be applied to the junction, the lower energy limit is effectively set by the width of the IETS peak (∼meV).^797^ For Raman scattering, spectral leakage from the laser line prevents measurements down to arbitrarily low wavenumber. While recent work has demonstrated the ability to resolve Γ point magnons in CrI_3_ down to ∼3 cm^–1^ (∼0.4 meV), other systems such as CrCl_3_ possess a smaller anisotropy with low-energy magnons in the GHz range (μeV).^795^ Other techniques such as Brillouin scattering may be needed to access this energy window.

Another way to potentially resolve these difficulties in studying magnetism of thin films is to develop resonant inelastic/elastic X-ray scattering (RIXS/REXS).^798^ RIXS-based techniques may combine the advantages of a few existing methods to characterize 2D magnetism. On the high throughput end, optical imaging can detect strain-induced band gap changes or polarized refraction at video speed, but the spatial resolution is diffraction-limited to about 0.5 μm.^246^ Scanning-probe-based NV-center magnetometry recently demonstrated atomic resolution in 2D FM materials, but the speed is as low as a few seconds per pixel.^21^ Lorentz electron microscopy provides high speed and high resolution only for ferromagnetism and is not sensitive to spin orders without net magnetization.^799^ In addition, RIXS-based techniques can probe the spin dynamics of both ferromagnets as well as antiferromagnets, as long as kinematic scattering conditions are satisfied. Furthermore, RIXS/REXS can be element-selective, thus allowing experiments to be carried out if there is more than one magnetic ion in a 2D magnetic material. However, current RIXS also has drawbacks due to the trade-off among energy resolution, flux, and spatial resolution in synchrotron light sources, which are fundamentally limited by brilliance. RIXS has limited energy resolution of ∼10 meV (L-edge resonance) that only applies to materials with large magnetic exchange couplings.^800,801^ These wavelengths are too long to cover the entire Brillouin zone in the reciprocal space, for example, of iron based and cuprate superconductors.^802^ K-edge RIXS uses shorter wavelength, but the energy resolution is much worse.^803^ Even with these constraints, one can utilize RIXS/REXS to study magnetism of thin films of selected materials.

## Heterostructures, Twisted Layers, and Interfaces

### Introduction

A pivotal advancement in the 2D materials community was the development of techniques to fabricate vertical heterostructures—stacks consisting of multiple layers of different 2D materials.^408,549,553,804−811^ Such techniques are especially versatile since they only rely on vdW interactions to produce heterostructures, allowing for nearly infinite possibilities by combining vdW materials with different electronic, magnetic, or physical properties. With the recent discovery of 2D magnetism within the family of available vdW materials, opportunities have arisen for incorporating 2D magnetic layers within more complex heterostructures.^62,213,809^ Stacking 2D magnets into vdW heterostructures offers an alternative setup to create spin and pseudospin textures, topological superconductivity, and other exotic quantum phases.^84,812^ The potential of magnetic vdW devices in spintronics, optoelectronics, and quantum technology applications is invigorating for contemporary research. There are certain advantages arising from the atomic thickness of 2D materials compared to their bulk counterparts. These include the increased strength of exchange interactions, which fundamentally alters the electronic structure of proximitized 2D systems, and the inherent flexibility of 2D materials which enables precise fabrication of vdW heterostructures.

In this section, we will overview three areas of ongoing research in vdW heterostructures fabricated from magnetic materials. We will begin by introducing the techniques used to incorporate magnetic 2D materials into heterostructures followed by a discussion of how heterostructures can be used to investigate the fundamental properties of 2D magnetism under multiple external parameters including carrier doping, electric field, and isotropic pressure. We will then discuss how heterostructures incorporating 2D magnets can be used to engineer phenomena through the control of adjacent layer twist angle and the proximity effect, in which magnetization can be induced in a nonmagnetic layer from an adjacent 2D magnet. In this context, the weak vdW nature of interlayer interactions not only relaxes the need to consider lattice mismatch between contiguous 2D layers, but also makes interlayer twist angle and stacking order design ingredients for the bespoke magnetic systems.^812^ vdW heterostructures offer tunability of 2D magnetism. Some of the tuning mechanisms include built-in electric fields and charge transfer at interfaces between constituent 2D layers; strain, lattice reconstruction, and orbital hybridization at interfaces with moiré patterns due to lattice mismatch or twisting between layers; and band structure renormalization due to exchange interactions, dielectric screening and spin–orbit proximity coupling, to name a few.^213^

### Heterostructure Fabrication

**Figure 70:**
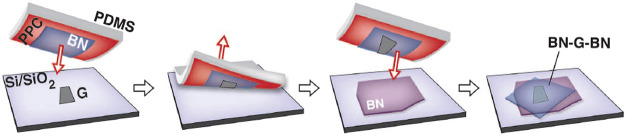
Process flow for vdW heterostructure fabrication. Schematic of the dry-polymer-transfer process used to fully encapsulate a 2D flake with hBN (here, it is graphene).^577^ This process relies only on the vdW interactions between hBN and other 2D flakes and can be used to pick up and transfer a number of different 2D materials. Reproduced with permission from ref (577). Copyright 2013 AAAS.

After 2D vdW crystals are isolated and identified on a suitable substrate (often SiO_2_/Si), the next step in fabricating a heterostructure is to remove the flakes from the substrate and prepare them for the incorporation of additional layers. Generally, this is accomplished by using an adhesive polymer (such as PMMA/PVA or PDMS/PPC/PC/PCL) that bonds to the flake more strongly than the flake bonds to the substrate.^553,577,579,804,805,808,811^ The primary method used for manipulating 2D magnets is the dry-polymer-transfer process in which a polymer is prepared on a glass slide which is then deterministically placed onto the desired 2D material.^553,577,804^ Once the flake is covered by the polymer, it can be slowly and controllably delaminated from the substrate by micromechanical manipulation or modulation of the sample temperature. This transfer process can be repeated with additional flakes to produce a multiple layer stack consisting of any number of desired 2D materials (typically limited to <10 flakes). Once the desired number of layers has been picked up, the entire heterostructure can be deposited onto a substrate by melting the transfer polymer (see Figure 70).

Due to the extreme sensitivity of most known 2D magnets to air, water, and solvents,^247,813,814^ heterostructures and devices using magnetic flakes must be prepared under inert conditions.^580^ Therefore, heterostructures are assembled using the dry-polymer-transfer process inside a glovebox environment. To protect flakes during the nanofabrication steps needed to create devices and subsequent transfer into measurement systems, encapsulated heterostructures are fabricated which sandwich the 2D magnet between two layers of hBN.^578,580,815^ The hBN serves both as an effective protective coating^578,580^ as well as a high quality dielectric material^579^ for gated measurements.

### Contact Methods

**Figure 71:**
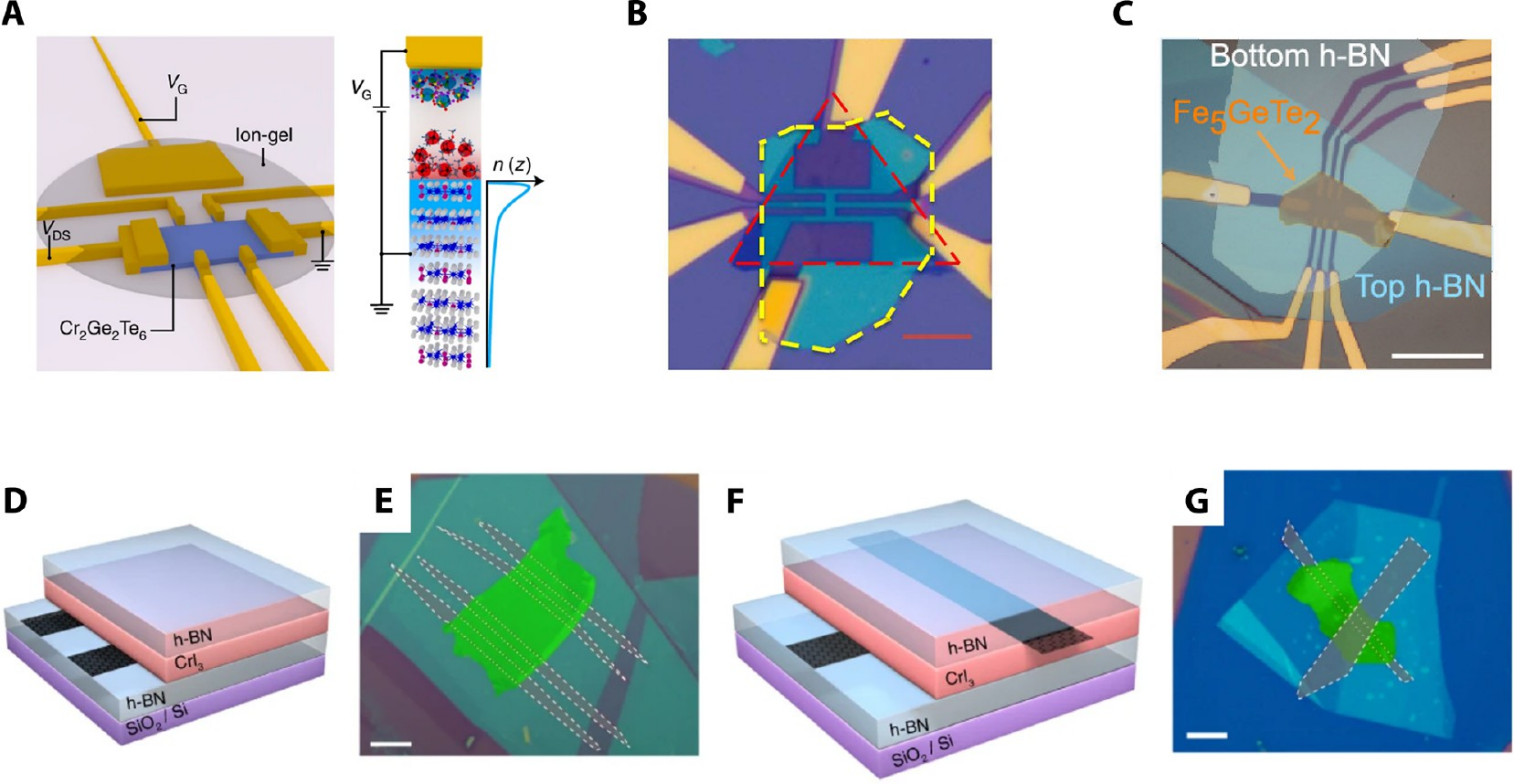
Contact methods for exfoliated vdW magnets. (A) Schematic of a Cr_2_Ge_2_Te_6_ flake top contacted with metal electrodes under an ion-liquid gate.^86^ Adapted with permission from ref (86). Copyright 2020 Springer Nature. (B, C) Optical image of a 5.8 nm-thick Fe_3_GeTe_2_ (B)^123^ and a 28 nm-thick Fe_5_GeTe_2_ (C)^87^ flake contacted from the bottom with prepatterned metal electrodes. In (B) the red dashed line is the Fe_3_GeTe_2_ and the yellow dashed line is the hBN. The red scale bar is 10 μm.^123^ In (C) the scale bar is 20 μm.^87^ Panel (B) was adapted with permission from ref (123). Copyright 2018 Springer Nature. Panel (C) was adapted with permission from ref (87). Copyright 2019 American Chemical Society. (D–G) Schematics (D, F) and corresponding false-colored optical images (E, G) of CrI_3_ flakes contacted by graphene electrodes for lateral (D, E) and tunneling (F, G) transport measurements.^15^ In (E) and (G), the scale bars are both 5 μm.^15^ Panels (D–G) are reproduced with permission from ref (15). Copyright 2018 Springer Nature.

The variety of electronic properties observed in 2D magnetic compounds coupled with the differing sensitivities to air, water, and solvents has required the development of various contact methods for fabricating electronic transport devices. Certain less sensitive 2D magnets (such as multilayer Cr_2_Ge_2_Te_6_,^10,86^ Fe_3_GeTe_2_,^12,77,109,123,500^ Fe_5_GeTe_2_,^816^ Fe_0.25_TaS_2_,^497^ or MnBi_2_Te_4_^111^) can be directly contacted using lithography and metal deposition without an encapsulating layer (Figure 71A). However, in the few-layer limit, most 2D magnets require indirect contact techniques combined with protection by an hBN layer. This ranges from direct etching and deposition after encapsulation in an inert environment (such as metallic Fe_4_GeTe_2_)^88^ or prepatterned metal electrodes for metallic Fe_3_GeTe_2_,^123^ (Figure 71B), CrTe_2_,^278^ and Fe_5_GeTe_2_,^87^ (Figure 71C) to intermediate graphene contacts for the semiconductor Cr_2_Ge_2_Te_6_.^10^ For the most sensitive magnetic materials (CrX_3_ family), they cannot be exposed to ambient conditions at any stage of fabrication, necessitating full encapsulation with hBN and graphene intermediate contacts (Figure 71D–G for two different contact schemes).^8,9,11,13−16,22,23,112,117−120,131,492,504^ To date, most available 2D magnetic semiconductors have poor charge transport properties. As additional 2D magnets with functional semiconducting properties are identified, an upcoming challenge will be fabricating devices with low-resistance contacts. Many of the contact techniques used for nonmagnetic 2D semiconductors^817−819^ (such as lateral junctions or phase contact engineering) are currently not feasible. Significant progress can be made if contact geometries are developed (such as metal electrodes embedded in hBN for simultaneous contact and protection),^820^ more metallic 2D materials can be identified (for work function engineering), or if more stable 2D magnets can be discovered. A promising candidate for the latter is the CrEX family from which CrSBr (a layered AF semiconductor)^110^ has shown superior air-stability down to the monolayer limit.^122^

### Overview of Heterostructures Based on Magnetic Materials

**Figure 72:**
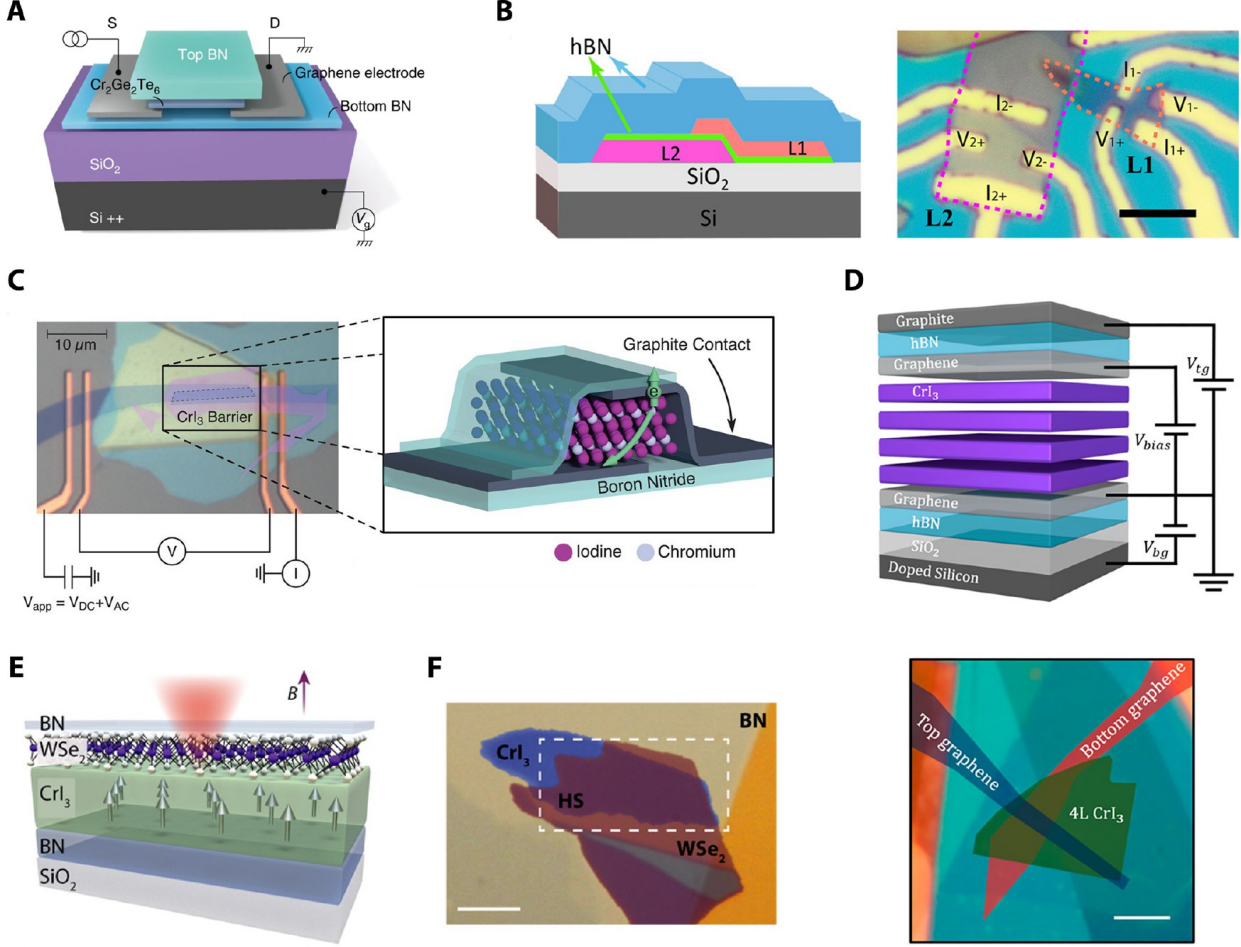
Heterostructures fabricated from 2D vdW magnets. (A) Schematic of a Cr_2_Ge_2_Te_6_ flake fully encapsulated with hBN and contacted by graphene electrodes.^10^ Reproduced with permission from ref (10). Copyright 2018 Springer Nature. (B) Schematic (left) and corresponding optical image (right) of a MTJ fabricated from Fe_3_GeTe_2_ electrodes separated by an hBN barrier.^109^ In the right panel, the dotted lines outline the edges of the two Fe_3_GeTe_2_ flakes. The scale bar is 5 μm. Reproduced with permission from ref (109). Copyright 2018 American Chemical Society. (C) Optical image (left) and corresponding cartoon (right) of a spin-filter MTJ utilizing CrI_3_ as the tunnel barrier between graphene electrodes.^14^ Reproduced with permission from ref (14). Copyright 2018 AAAS. (D) Schematic (top) and a false-colored optical image (bottom) of a spin-field-effect transistor fabricated from 4-layer CrI_3_.^504^ Graphene acts as both transistor electrodes and local electrostatic gates. The scale bar is 5 μm. Reproduced with permission from ref (504). Copyright 2019 American Chemical Society. (E, F) Schematic (E) and false-colored optical image (F) of a heterostructure proximitizing CrI_3_ with WSe_2_.^206^ The scale bar in (F) is 5 μm. Panels (E, F) are reproduced with permission under Creative Commons CC BY-NC 4.0 license from ref (206). Copyright 2017 AAAS.

Utilizing the versatility of the vdW stacking process, a variety of heterostructures have been fabricated from 2D magnets to understand the nature of magnetism in the 2D limit and fabricate functional spintronics. One example is full hBN encapsulation with the incorporation of electrodes such as prepatterned metal electrodes (Fe_5_GeTe_2_,^87^ see Figure 71B) or graphene electrodes (Cr_2_Ge_2_Te_6_,^10^ or CrI_3_^15^) (Figure 72A), which allows for the fabrication and measurement of lateral transport devices from conducting air-sensitive 2D magnets. The hBN has the additional advantage of being optically transparent, which allows for simultaneous characterization of the sample magnetization using optical probes.^10,15^

The second class of heterostructures are tunnel junctions fabricated from vdW magnets. Using layered FM 2D crystals, a heterostructure resembling a canonical MTJ can be fabricated from all vdW materials using either hBN, MoS_2_, or graphite (with Fe_3_GeTe_2_)^109,115,500^ or a naturally formed Ta_2_O_5_ surface (that forms upon the exposure of Fe_0.25_TaS_2_^497^ to ambient conditions) as the tunnel barrier (Figure 72B). The atomically sharp and clean interfaces naturally formed during stacking give rise to high quality tunneling interfaces. Due to the abundance of naturally layered AF vdW crystals, spin-filter MTJs can be easily created utilizing an AF 2D flake as the tunnel barrier between nonmagnetic metal electrodes (an alternative to the classical FM/I/FM MTJ structure). This concept has been demonstrated many times using the CrX_3_ compounds (CrI_3_,^11,13−16,22,23,112,120,492,504^ CrBr_3_,^112,120,131^ and CrCl_3_^112,117−120^), which boast exceptionally large on/off switching ratios upon a transition from an AF to a FM configuration, making them enticing for spintronic applications (Figure 72C).

Due to the aforementioned optical transparency of the constituent layers within the spin-filter-MTJ heterostructures, optical probes (such as Raman spectroscopy,^23,118^ MCD,^8,13,22,504^ and Kerr rotation^9,15^) are used to correlate tunneling measurements with direct measurements of the sample magnetization and structure, making these heterostructures a model system for investigating the nature of magnetism in the 2D limit. Toward this end, more complex heterostructures have been fabricated to include dual graphite gates (Figure 72D), which allows for the measurement of the magnetic properties (through either optics or tunneling transport) as a function of carrier doping or local electric field^8,9,11,492,504^ and hydrostatic pressure.^22,23^

Beyond studying the intrinsic properties of 2D magnets, heterostructures can be fabricated for the purpose of inducing magnetization in a nonmagnetic 2D layer through the proximity effect. For example, it was shown in a heterostructure consisting of CrI_3_ and WSe_2_ that the valley polarization in WSe_2_ was directly linked to the magnetization of the CrI_3_ and was tunable with an external magnetic field (Figure 72E,F).^206^

Band structure engineering can be used to induce magnetic response in nonmagnetic 2D materials. For instance, magnetism from itinerant electrons can be tuned by twistronics, where properties of vdW materials are controlled by changing twist angle between constituent 2D layers. With recent advances twistronics provides a strategy to reliably fabricate devices with arbitrary rotational order and *in situ*, thus significantly facilitating research in vdW heterostructures.^821−823^

**Figure 73:**
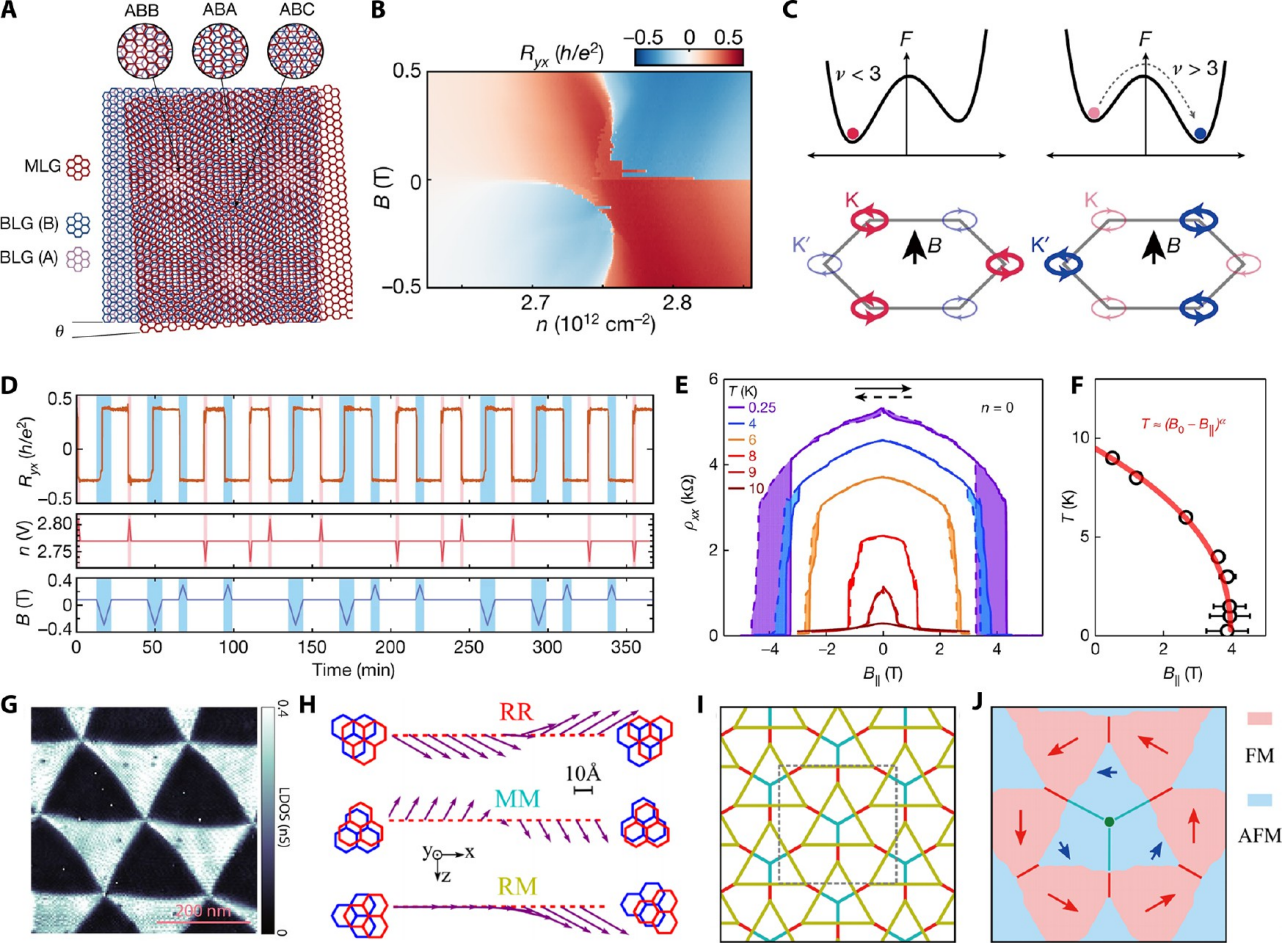
2D magnetism controlled by twistronics and stacking order. (A) Variation of stacking order in small twist angle θ monolayer–bilayer graphene (tMBG).^828^ (B) Transverse resistance *R*_*yx*_ map measured versus total carrier density *n* and the magnetic field *B* at *T* = 6.4 K in a tMBG device with θ = 1.25° near ν = 3 orbital magnetic state presents a magnetization reversal driven by the change in *n* or *B* due to the non-negligible contribution from the topological edge states in large moiré unit cell area. ν = *nA* is the number of carriers *n* per moiré unit cell *A*.^828^ (C) In *B*-field, ν = 3 state switches between K and K′ valley polarization as doping level changes across the gap.^828^ (D) Nonvolatile switching between K and K′ valley-polarized magnetic states independently controlled by either *n* or *B*.^828^ Panels (A–D) reproduced with permission from ref (828). Copyright 2020 Springer Nature. (E) Temperature-dependent hysteresis (highlighted by colored areas) observed in magnetic field in rhombohedral graphite.^832^ (F) Phase diagram of the critical behavior in (E) characteristic to strongly correlated electronic systems.^832^ Panels (E, F) are reproduced with permission from ref (832). Copyright 2020 Springer Nature. (G) Scanning tunneling spectroscopy map of a small θ double bilayer graphene showing Bernal (black) and rhombohedral (white) stacking domains.^833^ Reproduced with permission under a Creatice Commons CC BY-NC 4.0 license from ref (833). Copyright 2021 National Academy of Sciences. (H–J) Rhombohedral (R) and monoclinic (M) stacking configuration and magnetic domains in a small θ twisted bilayer CrI_3_. (H) Three types of stacking domain walls in this system. Arrows represent the stacking vectors.^834^ (I) Sketch of the magnon network at θ = 0.1°.^834^ (J) Stacking and magnetic domain patterns of the gray rectangle area in (I). Red (blue) arrows represent stacking vectors for R (M) stacking. Red (cyan) lines represent the RR (MM) stacking domain walls.^834^ Panels (H–J) are reproduced with permission from ref (834). Copyright 2020 American Physical Society.

In rotated graphene bilayers, a moiré superlattice results in the formation of moiré minibands which become particularly flat at specific magic angles.^824^ These flat minibands have vanishing Fermi velocity resulting in a large density of states which gives rise to electron–electron interactions. Recent experiments on magic-angle twisted bilayer graphene (MAG)^710,824,825^ triggered the use of twistronics for tuning interaction strength in 2D materials, where research on moiré superlattices in MAG revealed a score of correlated phases including ferromagnetism and quantum anomalous Hall state.^700^ For instance, at a fractional filling (3/4) of a moiré miniband, electronic interactions make MAG magnetic, as signified by a strong FM hysteresis and a strong anomalous Hall effect.^826^ Aligning MAG to a hBN substrate increases the strength of interactions and stabilizes magnetism, leading to a stronger orbital magnetization and a clear quantum anomalous Hall effect.^255,827^ This incipient magnetism could arise from strong interactions leading to spin-valley polarization of moiré minibands, characteristic for an orbital Chern insulator state. Notably, magnetic order of these MAG devices can be controlled by a nanoampere electrical current, showing potential for electrically controllable magnetic memory applications. Using twisted monolayer–bilayer graphene allows for the control of orbital Chern insulator magnetic states directly with electric fields (Figure 73A,D), which is crucial in designing reconfigurable circuits and ultralow-power magnetic memory^828^ for spintronics and multiferroics applications. Recently, spatially resolved measurements of the local electronic compressibility in MAG revealed a peculiar high-temperature state with a large spontaneous magnetization. From this broken spin-valley symmetry state, more fragile low-temperature correlated ground states stem.^829^ At quantizing magnetic fields, MAG devices demonstrate field-stabilized orbital magnetic states, demarcated by first-order phase transitions, and driven by the interplay of moiré periodicity and magnetic length scale.^830,831^

Another pure carbon material which exhibits emergent correlated phases is a multilayer rhombohedral graphene (RG), a simple material with flat electronic bands but without a moiré superlattice.^832,833^ VdW technology enabled the study of electronic transport in high-quality multilayer RG,^835^ which revealed strong correlations and behavior characteristic of multiferroic materials^832^ (shown in Figure 73E,F), while scanning tunneling spectroscopy revealed that tetralayer RG has a correlated many body broken symmetry state probably of an excitonic insulator or a ferrimagnet nature (Figure 73G).^833^

### Twistronics in Ferromagnetic Materials

Twisting magnetic 2D materials could lead to an interesting interplay between magnetism and topology.^700^ For instance, tunable magnetic moiré skyrmions (topologically protected vortex-like magnetization textures) are predicted to form when a 2D ferromagnet is twisted on an AF substrate.^490,836^ A magnonic (spin wave) analogue of MAG is theorized in a simple twisted FM bilayer model without lattice relaxation; DMIs are predicted to result in a rich topological magnon band structure.^837^ By fully accounting for lattice relaxation, theoretical calculations of a twisted bilayer CrI_3_ predict stacking domain walls which would host stable 1D magnon channels (originating from Goldstone modes of the spin Hamiltonian) arranged into an interconnected moiré magnon network^834^ (Figure 73H–J). Recently developed general formalism of twisted (anti)ferromagnetic bilayers should stimulate further theoretical and experimental research in magnetic moiré heterostructures.^778^

### Effects of Strain and Hydrostatic Pressure

**Figure 74:**
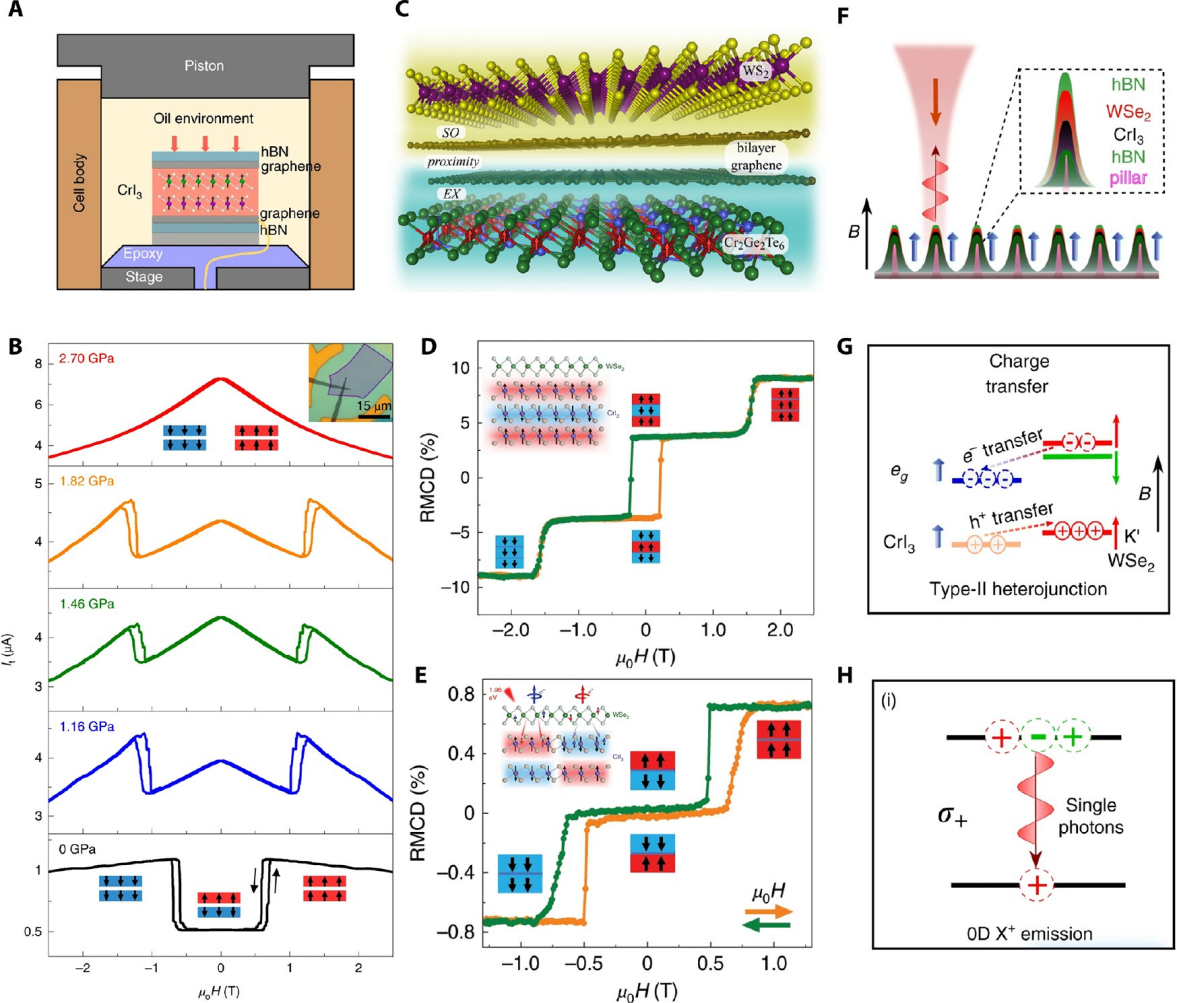
Tuning magnetic vdW heterostructures. (A) Schematic of a high-pressure setup for a MTJ. Yellow line represents electrical leads.^22^ (B) Tunnel current *I*_*t*_ versus magnetic field *H* at pressures from 0 to 2.7 GPa in a bilayer CrI_3_ MTJ. Insets show spin alignments and optical image of the MTJ.^22^ Panels (A, (B) are reproduced with permission from ref (22). Copyright 2019 Springer Nature. (C) Proposed vdW heterostructure where exchange (EX) and spin–orbit (SO) coupling can be swapped by an electric field. Cr_2_Ge_2_Te_6_ magnetization is denoted by red arrows.^840^ Adapted with permission from ref (840). Copyright 2020 American Physical Society. (D, E) Dependence of RMCD on magnetic field in heterostructures of WSe_2_ and trilayer (D)/bilayer (E) CrI_3_ (shown in the insets).^203^ Panels (D, E) are reproduced with permission from ref (203). Copyright 2020 Springer Nature. (F–H) Photoemission and spin-dependent charge transfer in hBN encapsulated CrI_3_/WSe_2_ heterostructure. (F) A schematic of the heterostructure deposited on nanopillar array.^841^ (G) Spin-dependent charge transfer from spin-polarized states in WSe_2_ to CrI_3_ results in the highly p-doped WSe_2_, where an exciton can be turned into a localized charged trion (0D X^+^) *via* a hole capture process (H). Arrows in red and green (blue) denote the spin direction in WSe_2_ (CrI_3_).^841^ Panels (F–H) are reproduced with permission under a Creative Commons CC BY license from ref (841). Copyright 2020 Springer Nature.

Tuning material parameters such as interlayer separation or stacking order *via* the application of pressure or strain is an effective method for controlling magnetism in vdW materials.^700^ For instance, a hydrostatic pressure of 1 GPa significantly affects the Curie temperature of Cr_2_Ge_2_Te_6_,^838^ while higher pressures reorient its spins from out-of-plane to in-plane.^283^ Furthermore, interlayer exchange coupling depends on layer separation and stacking order which can both be tuned by hydrostatic pressure, as seen in hBN/graphene/CrI_3_/graphene/hBN heterostructures, pressure induces an AF-to-FM transition in bilayer CrI_3_^22,23^ (Figure 74A,B). Nanoscale structural modifications also induce switching between FM and AF ordering, as was in the case of magnetic transition and enhanced magnetization observed in hBN- or graphene-encapsulated CrI_3_ flakes indented with a diamond scanning probe.^21^ Another possibility in controlling magnetism in vdW materials is through strain.^700^ A FM phase transition at room temperature is predicted in CrWI_6_ and CrWGe_2_Te_6_ monolayers subjected to an in-plane tensile strain,^19^ while monolayer chromium trihalides show AF phase transition upon a compressive strain.^839^

### Proximity Effects at vdW Interfaces

In atomically thin vdW materials, magnetic proximity effects become dominant, as even short-range effects exceed the thickness of contiguous 2D crystals.^812^ The proximity of 2D magnets can break time reversal symmetry in nonmagnetic 2D materials, leading to valley polarization in TMDs, quantum anomalous Hall effect in topological insulators, and other emerging phenomena including multiferroicity and topological superconductivity.^213^ For instance, FM exchange interactions from the 2D magnet Cr_2_Ge_2_Te_6_ induce an anisotropic spin texture in proximitized graphene.^450^ Similarly, the proximity of magnetic CrSBr to a bilayer graphene results in a large spin polarization of the graphene conductivity, making CrSBr/graphene heterostructure a promising system for spintronics applications.^452^*Ab initio* calculations for a Cr_2_Ge_2_Te_6_/PtSe_2_ heterobilayer suggest that 2D magnetism can be significantly enhanced by proximity of a 2D material with strong SOC.^842^ Another theoretical work predicts that the proximity of a bulk semiconductor substrate to a 2D CrI_3_ significantly increases its FM exchange interactions.^843^ Furthermore, in vdW moiré superlattices magnetic proximity effects can be tuned by twisting and/or strain between the vdW layers (Figure 74D,E).^844^ vdW engineering in combination with circular dichroism measurements demonstrate an interesting approach to control and probe a layer-dependent magnetic proximity effect between monolayer WSe_2_ and bi/trilayer CrI_3_, promising for spin- and valleytronics applications.^203^ The proximity of hBN encapsulated graphene to a 2D magnet allows for the detection of proximitized magnetic order *via* graphene-based ballistic Hall micromagnetometry.^82^

Combining a 2D ferromagnet and a superconductor into a vdW heterostructure provides a tunable route to the exotic state of a topological superconductor–a key element for topological quantum computing, due to the emergence of one-dimensional Majorana edge modes which are robust against disorder. MBE is a scalable approach to creating such designer topological heterostructures. For example, MBE was used to grow a 2D ferromagnet CrBr_3_ on the superconductor NbSe_2_, resulting in a high-quality interface in which signatures of Majorana modes were detected.^83,845^ This approach can be extended to other hybrid superconductor-magnet heterostructures, for example, NbSe_2_ and VSe_2_ give clean and atomically sharp interfaces, where a decrease in the superconducting gap of NbSe_2_ due to a magnetization of the VSe_2_ sheet was observed.^846^ Even more opportunities to study exotic strongly correlated behavior can be found in organic–inorganic heterostructures *via* molecular intercalation.^847^

### Spin–Orbit Coupling and Optical Control of Magnetic Properties

Light–matter interactions provide an excellent opportunity to both probe and control magnetically ordered phases in vdW materials with considerable SOC.^84^ For instance, ferromagnetism in 2D materials can be directly probed using helicity-resolved Raman spectroscopy due to the spin angular momentum carried by circularly polarized light.^225^ Spin-dependent charge transfer between spin-split bands of WSe_2_ and CrI_3_ can be used to generate charged excitons, which can be arranged into large-scale deterministic arrays of quantum emitters *via* strain-inducing nanopillars^841^ (Figure 74F–H). Contributing to spin-photonics applications, room-temperature spin polarization can be achieved in heterostructures of monolayers of WS_2_ or WSe_2_ with PbI_2_, in which the spin polarization can be tuned by PbI_2_ layer thickness, temperature, or excitation energy.^848^ A theoretical proposal unites the fields of twistronics, spintronics, and many-body physics by engineering a heterostructure of a twisted WSe_2_ bilayer sandwiched between magnetic CrBr_3_.^849^ This moiré system features flat bands with tunable valley and spin ferromagnetism emerging from the interplay between twist engineering, SOC, and exchange proximity and provides a starting point to study strongly correlated systems tunable *via* exchange bias. Another theoretical work suggests that exchange and SOCs can be swapped by an applied electric field in a bilayer graphene (BLG) sandwiched between a 2D ferromagnet Cr_2_Ge_2_Te_6_ (CGT) and a monolayer WS_2_, where CGT provides proximity exchange coupling to the bottom layer of BLG, while WS_2_ induces a SOC to the top BLG layer (Figure 74C).^840^ This doubly proximitized BLG heterostructure can be further extended to systems comprising (anti)ferromagnets, ferroelectrics, topological insulators, and superconductors, and offers a platform to explore emergent spin physics.

### Electrical Control of Magnetic Properties

Electrical control of 2D magnets offers another convenient tuning knob. In a bilayer CrI_3_ antiferromagnet, electric fields and electrostatic doping can affect the spin-flipping magnetic field, modulate the Curie temperature, and induce an AF-to-FM transition.^8,9,11^ The high quality of the interface in the MoSe_2_/CrBr_3_ heterostructure leads to a strong splitting of valley excitons in MoSe_2_ in zero magnetic field, with a distinct electric field dependence indicating a potential for electrical control of magnetization.^204^ vdW heterostructure multiferroics bring forward low-dimensional magnetoelectric physics and spintronic applications thanks to the increased efficiency of electrical control of magnetism enabled by their inherent coupling between magnetic and electric orders. Although some theoretical works predict single-phase 2D magnetoelectric multiferroics in CuMP_2_X_6_ (M = Cr, V; X = S, Se), CrN, and CrB_2_ systems,^850,851^ competing requirements of the orbital filling for ferroelectricity (empty d-orbitals) and ferromagnetism (filled d-orbitals) make these systems scarce. Another computational research circumvents this by suggesting multiferroics based on a 2D heterostructure comprised of FM Cr_2_Ge_2_Te_6_ and ferroelectric In_2_Se_3_, where the magnetism of Cr_2_Ge_2_Te_6_ switches following the polarization reversal of In_2_Se_3_ which in turn becomes a magnetic semiconductor due to the proximity effect.^852^

### Conclusions and Outlook

The advent of techniques to fabricate heterostructures consisting of multiple layers of vertically stacked 2D vdW materials has allowed for the creation of myriad systems by combing materials with different electronic, magnetic, or other physical properties. With the recent surge of 2D magnetic materials, opportunities have emerged for fabricating heterostructures incorporating magnetic vdW materials. These magnetic heterostructures provide unparalleled access to a wide range of condensed matter systems with many exotic properties. Designer magnetic materials offer countless opportunities for engineering desired properties tunable by electrical, optical, mechanical, chemical, and other external stimuli, which could lead to hybrid artificial heterostructures with applications in spintronics, data storage, optical communications, and quantum computing.^213^ There are many challenges still present including developing more stable materials, tuning magnetic phases that exist at room temperature, optimizing fabrication techniques to more reliably fabricate heterostructures with multiple layers and various configurations, improving contacts to 2D magnets for functional electronics, and creating 2D magnets on a wafer scale for industrial processing.^213^

## Theory and Simulations

### Introduction

Experimental measurements present significant technical challenges in the area of 2D magnetic materials due their characteristically low magnetic ordering temperatures and general volatility. In contrast, simulation methods have advanced considerably in the past 20 years in terms of fundamental capabilities, accuracy and speed. Today it is possible to routinely perform magnetic first-principles and atomistic simulations with high accuracy and develop a deep and fundamental understanding of the underlying physical properties of 2D magnets. The broad availability of advanced simulation software and high performance computing facilities has pushed the frontier for 2D materials discovery from the lab to the desktop computer. Such a shift is important in the field of condensed matter and even at the birth of this exciting field it is clear that existing approaches are able to uncover a plethora of exciting magnetic phenomena with untold possible applications. In this section we aim to provide a basic framework in terms of fundamental theory and simulations techniques for exploration of 2D vdW magnetic materials in a feasible manner.

### Theory

The general theory of magnetism is very well established and expressed in terms of a generalized spin Hamiltonian describing the magnetic interactions in the system. It is important to note the deliberate absence of nonmagnetic contributions to the Hamiltonian in this formalism. The effective parameters (i.e., exchange, single-ion anisotropy, magnetic moment) in the spin Hamiltonian are always computed *ab initio* with the full spin Hamiltonian including lattice, kinetic energy and relativistic contributions that are simply mapped to a set of magnetic interactions based on their symmetry and order. The general spin Hamiltonian is given by
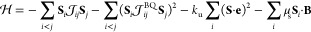
14where **S**_*i*,*j*_ are unit vectors describing local spin directions,  and  are the bilinear and biquadratic exchange tensors respectively, *k*_u_ is the single-ion uniaxial anisotropy constant, μ_s_ is the local spin moment, and **B** = μ_0_**H** is the externally applied magnetic (induction) field in tesla. The standard bilinear exchange interaction is given by a summation of all pairwise interactions for the index *i* < *j* to avoid double counting with the vector-tensor-vector multiplication:
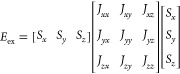
15Here the exchange matrix includes three physical interactions in a compressed format for ease of computation and expressiveness. The tensor can be expanded in the following way:
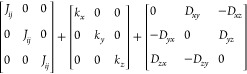
16and includes isotropic exchange interactions *J*_*ij*_, 2-ion anisotropy *k*_*x*_, *k*_*y*_, *k*_*z*_, and Dzyaloshinskii–Moriya interactions *D*.^744^ In this form, the exchange tensor also encapsulates the Kitaev interaction^704^ and the *XXZ* model within a more general framework that extends beyond nearest neighbor interactions.^853^ The biquadratic exchange is a higher order exchange term that is an important correction for 2D magnets necessary to properly describe temperature dependent properties^796^ and spin waves.^725^

The principal assumption of the Heisenberg model is that of fixed length, localized magnetic moments, where the energy of the system is described purely in terms of coherent rotation of moments. This is generally a good approximation for bulk Fe, Co, and Mn, and less so for bulk Ni and Cr which have a more itinerant character. In 2D materials the validity of the local moment approximation is generally better than the bulk case^796^ due to the semiconducting character of the materials and enhanced electron localization. In the case of more itinerant-like spins longitudinal moment fluctuations can likely be represented by a Landau expansion of the Stoner model.^854^

The final energetic contribution of importance is the dipole–dipole field, or magnetostatics in bulk magnets. The dipole–dipole interaction is long ranged and critically important for the magnetic ground state spin orientation and the formation and evolution of magnetic domain structures. Considering a localized magnetic moment **m**_*i*_ = μ_*i*_**S**_*i*_ at position **r**, the field **B**_*i*_ from all other dipoles **m**_*j*_ = μ_*j*_**S**_*j*_ with position vector  is given by
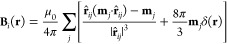
17where δ(**r**) is the 3D Dirac δ-function and represents the self-field of the dipole. In conventional micromagnetic simulations, the magnetostatics are accounted for with the continuum approximation, but in 2D materials where the domain walls are exceptionally narrow, it is important to consider the effects of the lattice structure and thermal spin fluctuations on the dipole field.^31^ Direct calculations of the dipole–dipole field for large systems is computationally expensive but possible on large scale parallel computers. Other faster techniques such as fast Fourier transforms rely on translational invariance of the spins which is problematic for certain structures but in particular honeycomb lattices. Hierarchical approaches are promising in this regard, combining locally exact computations and far-field approximations for the ideal balance of accuracy and computational efficiency.

### Monte Carlo Methods

The *ab initio* parameters give important insight into the fundamental interactions in 2D materials, but present only the case of fully ordered moments at zero temperature. However, experimental measurements are always conducted at finite temperature and so the temperature dependence of the properties is an important component of validating simulations against experimental data. Simple analytical approaches such as mean field and random phase approximation^855^ allow the estimation of Curie temperatures of magnetic materials, but today Monte Carlo Metropolis methods provide a fast and efficient way of finding equilibrium thermodynamic properties of a magnetic system.^856^ The Metropolis algorithm^857^ takes a random spin with direction **S**_*i*_ and changes its spin direction to a trial direction . The next step is to calculate the change in energy between the initial and final states . The trial direction is either accepted or rejected based on an acceptance probability (*P*):
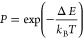
18where *k*_B_ is the Boltzmann constant and *T* is the absolute temperature. If the change in energy is less than zero then the probability is greater than one and the spin is automatically accepted. This is repeated *N* times with *N* corresponding to the number of atoms in the system, completing one Monte Carlo step.

An important aspect of Monte Carlo Metropolis simulations is the sampling method used to select trial spin positions . Early models of 2D magnets assumed an Ising model, conceived by Ernst Ising in 1925,^33^ where the spin system is evolved by spin flips  assuming a global quantization axis, usually defined by symmetry. As discussed later, this assumption leads to artifacts and a significant overestimation of the Curie temperature that are unrealistic. A natural extension of the Ising model is the classical Heisenberg model in two and three dimensions. Note that the dimensionality refers to the spatial dimensionality of the lattice coordinates: The Heisenberg model explicitly describes an atomic spin with the freedom to orientate anywhere in 3D space. Even though quantum mechanical effects are neglected, the Heisenberg model is capable of accurately modeling phase transitions, temperature dependent properties and surface and finite size effects. In the Heisenberg model the random spin positions must obey the principle of detailed balance.^857^ The simplest way of satisfying this is if the moves are uniformly distributed over the unit sphere.

An efficient computational method for this was devised by Marsaglia^858^ and is known as the uniform method.^856^ This computational method is efficient at high temperatures, however is inefficient at low temperatures, since due to the exchange energy only moves with a small change in spin position will be accepted. Another possibility for sampling the surface is a Gaussian method, sampling a Gaussian distribution around the initial spin position the width of which is dependent on the temperature. This improves the acceptance rate (by sampling small spin moves) to achieve better computational efficiency but also presupposes that small moves are thermodynamically preferred, though in the case of exchange coupling this is a reasonable approximation. To ensure that all spin states are accessible, it is often beneficial to combine these Monte Carlo trial moves (spin-flip, random and Gaussian) into the sampling approach, found by Hinzke and Nowak.^859^ The three possibilities of trial moves allow the system to equilibrate quickly at any temperature by allowing large changes in the spin position at high temperatures using the uniform and spin flip sampling and smaller changes at low temperature using the Gaussian sampling. More recently an adaptive sampling algorithm for Heisenberg spin models was developed by Alzate-Cardona *et al*.^860^ which uses Gaussian sampling with a dynamically tunable width to achieve a constant acceptance rate of 50%, which was found to be more efficient at all temperatures compared to the previous approaches. In addition constrained Monte Carlo methods^861^ allow the determination of temperature dependent properties such as the effective exchange coupling^862,863^ and magnetic anisotropy^864^ though such properties are so far unexplored in 2D VdW materials.

### Ising versus Heisenberg Descriptions of 2D Magnets

At this point it is useful to discuss the different Monte Carlo methods that have been used to describe the magnetism of 2D materials. Earlier works described the magnetism of perpendicularly magnetized 2D magnets as Ising-like,^5,866^ in reference to the simple model solved by Ernst Ising^33^ and used as a prototypical model for magnetic ordering. However, this classification is unhelpful as the global quantization axis for atomic spins in the Ising model leads to three erroneous physical effects that do not apply to real systems. First, the global quantization axis leads to an infinite magnetic anisotropy, preventing a coherent rotation of the system by an external magnetic field, such as seen for hysteresis measurements. Second, the existence of spin waves is completely forbidden as spins are only permitted to exist along a quantization axis. Third, the removal of infinitesimal spin rotations leads to an artificially flat temperature dependence of the magnetization at low temperatures, and an unrealistically high Curie temperature when considering realistic exchange parameters calculated *ab initio* using density functional theory. While the Ising model has its place in fundamental statistical physics, it is wholly inappropriate to model the temperature dependent properties of actual 2D magnetic materials. This has unfortunately led to the prediction of excessively large Curie temperatures in a number of studies.^32,120,294,867−870^

**Figure 75:**
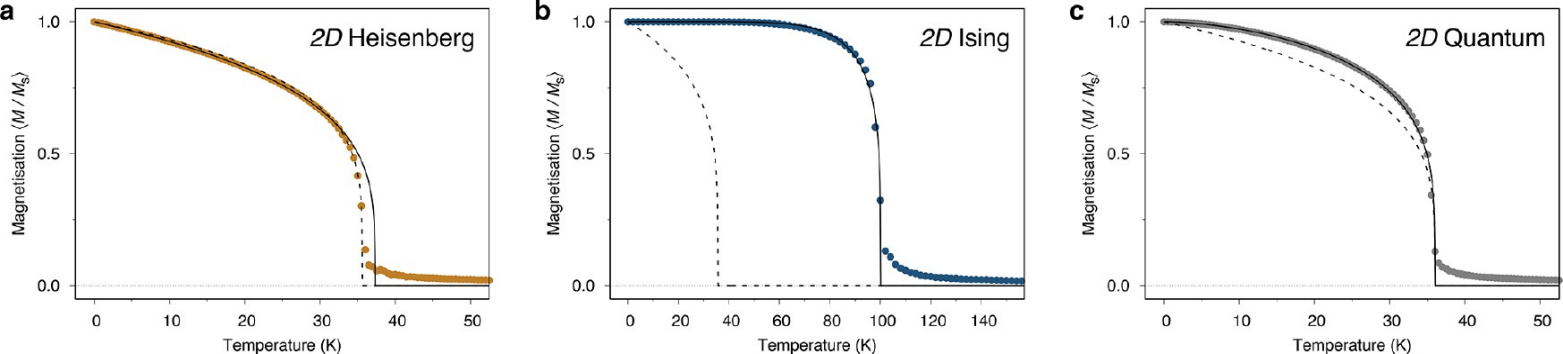
Comparison of different Monte Carlo models calculating the Curie temperature of CrI_**3**_. (a) Classical 2D Heisenberg. (b) Classical Ising model. (c) Quantum-like simulation with temperature rescaling. All panels are adapted with permission from ref (865). Copyright 2015 American Physical Society.

The 2D Heisenberg model, where spins are permitted to orientate anywhere in 3D space but sites are confined to two dimensions, provides a much better prediction of magnetic ordering temperatures that closely match the experimentally measured values^31,796,871^ of 2D magnets. To explore the qualitative and quantitative differences between the Heisenberg and Ising models, we consider calculations of the temperature dependent magnetization of CrI_3_ in Figure 75. Based on the *ab initio* parameters of Wahab *et al.*,^31^ the 2D Heisenberg model finds a Curie temperature of 36 K, which is close to the experimental value. The temperature dependence of magnetization *m*(*T*) of the 3D Heisenberg model is accurately described in the whole temperature^865^ by the equation
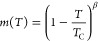
19where *T* is the temperature, *T*_C_ is the Curie temperature and β = 0.340 ± 0.001 is the critical exponent. In 2D materials of finite size there is a crossover in the critical exponent between the low temperature behavior and high temperature behavior, where small quantitative differences in the fitting function and the data appear. In our example in Figure 75a, fitting to the low temperature data up to *T*/*T*_C_ = 0.9 yields a critical exponent of β = 0.2480 ± 0.0007 and a slight overestimation of the Curie temperature, while fitting to the high temperature regime *T*/*T*_C_ > 0.9 yields an exponent of β = 0.212 ± 0.028 and an accurate estimate of the Curie temperature. We note that both of these exponents are substantially lower than the exponent β = 0.34 for the 3D Heisenberg model. Others have stated that critical exponents such as β are universal to particular classes of model^279,294^ but more recent data presents a more complicated story. First, the critical exponent for the 2D Heisenberg model of CrI_3_ with *ab initio* parameters is closer to 2.1 in the vicinity of the Curie temperature and significantly different from the exponent for the 3D case. Second, the exponent in reality depends on the different parameters in the Hamiltonian, particularly the presence of significant magnetic anisotropy and higher order exchange interactions. In particular a nearest neighbor exchange and uniaxial anisotropy for the 2D Heisenberg model yield an exponent of β = 0.34, identical to the case for the 3D Heisenberg model. At the time of writing there is little work on critical exponents in pure 2D Heisenberg systems, but this is likely to be an area of focus in the next few years and critically important for determining where universality does and does not apply in 2D magnetic systems.

In Figure 75b, we show the simulated temperature dependent magnetization using an Ising Monte Carlo model for the same parameters, showing an unrealistically large predicted Curie temperature and essentially flat temperature dependent magnetization at low temperatures. The fit to the 2D Heisenberg model data in Figure 75a is shown for comparison. As previously noted, both of the main features of the Ising model are physically unrealistic and such simulations do not provide helpful comparisons to real 2D magnets, and as such should no longer be used. The final point to discuss is the disparity between experimentally measured temperature dependent magnetization curves and those obtained from a Heisenberg model, both in three^865^ and two^31^ dimensions. The main characteristic feature of Heisenberg models is the linear variation of the magnetization with temperature as *T* → 0. This is a consequence of the classical nature of the Heisenberg model, while the underlying heat bath responsible for spin fluctuations is quantum mechanical in nature.^31,865^ A relatively simple adaptation of the Heisenberg model is able to reproduce the quantum nature of the heat bath observed experimentally by fitting the temperature dependent magnetization to the Curie–Bloch equation^865^ which interpolates between the Bloch law behavior as *T* → 0 and Curie behavior as *T* → *T*_c_:
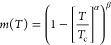
20where α is the Bloch exponent determining the shape of the magnetization curve as *T* → 0. The Curie–Bloch equation accurately describes the temperature dependence of the magnetization of 3D^865^ and 2D^31^ magnets in the full temperature range, while retaining a natural link to the critical behavior (with the same exponents) near the Curie temperature.^865^ The classical limit is recovered for α = 1. A convenient phenomenological extension to Heisenberg models is through spin temperature rescaling, which allows the direct mapping of the classical Heisenberg model onto the Curie–Bloch equation,^865^ as shown in Figure 75c. The rescaling mimics the effects of the quantum heat bath by reducing the strength of thermal fluctuations at low temperatures and allows for quantitatively accurate simulations of temperature dependent properties and dynamics of 2D magnets that are directly comparable to experiment. The full nature of the heat bath is a complex question and other approaches may reveal the microscopic details and interactions that will enable a fully *ab initio* description of the microscopic spin flip and spin scattering processes.^872^

### Atomistic Spin Dynamics

Atomistic spin dynamics (ASD) is a more recently developed framework for simulating the dynamics of localized magnetic spin moments^856,873,874^ based on the atomistic Landau–Lifshitz–Gilbert equation (LLG).^875^ The LLG equation combines the quantum mechanical precession of atomic moments around an externally applied magnetic field with a phenomenological relaxation term that allows fro energy transfer from the spin system to the heat bath. Most studies of 2D magnets have so far focused on equilibrium properties computed using Monte Carlo Metropolis methods, but ASD enable an exciting class of dynamical simulations that have a correspondence to the time-dependent dynamics of materials.^876^ The time-dependent behavior described by the LLG equation is given by
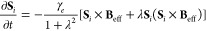
21where **S**_*i*_ is a unit vector describing the direction of atomic spin moment *i* with an effective magnetic field (**B**_eff_). The effective field causes the atomic moments to precess around the field, where the frequency of precession is determined by the gyromagnetic ratio of an electron (γ_*e*_ = 1.76 × 10^11^ T^–1^ s^–1^), and λ is the microscopic Gilbert damping constant describing the coupling to the heat bath.^875^ The effective magnetic (induction) field is given by the first derivative of the spin Hamiltonian:
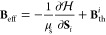
22where μ_*s*_ is the local spin moment and augmented by a stochastic thermal field. In the LLG equation, the damping term λ models the energy dissipation of the system, considering energy transfer to and from the lattice and electron degrees of freedom. Formally the LLG equation does not include any description of temperature and is only valid at 0 K, and so to include the effects of temperature effects, we use Langevin dynamics.^877^ The effect of temperature is introduced by coupling the system to a heat bath in the form of a fluctuating thermal field  into the effective field^873^ described by
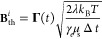
23where **Γ**(*t*) is a Gaussian distributed random number in three dimensions and Δ*t* is the integration time step. This formalism assumes a white noise approximation where the time correlation between the spin fluctuations induced by the thermal field must be shorter than the spin motion. This assumption is justified for metallic systems in that the time-scale of the electron heat bath is much faster than the spin system. For insulators and semiconductors the validity of this assumption is less clear and further work is needed to assess the noise correlations, for example through spin–lattice dynamics simulations.^878^

### Codes for Atomistic Simulations

There are now a number of standard software packages available for computing temperature dependent magnetic properties using atomistic models including vampire,^856^uppasd,^874^spirit,^879^ and fidimag(880) which contain built-in and well-tested routines for computing basic properties such as the Curie temperature, spin dynamics and hysteresis loops. Different codes also implement energy minimization algorithms to determine energy barriers and temperature dependent magnetic properties such as the effective exchange coupling and anisotropy. Most of these packages support parallel simulations using multiple CPUs and GPUs that enable much faster calculations than available from typical serial codes, enabling much larger simulations of 2D materials over much longer time scales more readily comparable with experiment.

### Electronic and Magnetic Structure

First-principles methods of different flavors are the most common approaches for the calculations of electronic and magnetic properties of 2D magnets. In particular, the development of spin DFT over the past two decades have allowed the fast exploration of spin-dependent properties. On the backbone of these approaches is the treatment of the underlying open-shell problem within nonrelativistic and the relativistic frameworks.^881^ Indeed, the development and improvement of DFT methods for open-shell (*e.g.*, unpaired electrons) materials is currently one of the most important and challenging topics in theoretical chemistry/physics up to date.^882^ Several alternatives have been developed that may overcome this limitation.^883^

2D magnetic materials are not immune to such problems, which careful investigations are desired to address possible shortcomings. The majority of simulation results published so far have either undertaken vast amount of computations using plain DFT functionals (*i.e.*, linear density approach (LDA), generalized gradient approach (GGA)),^888,889^ or used Hubbard-*U* approaches (*i.e.*, DFT+*U*).^341,890^ A very few papers have been published using approaches beyond mean-field theory for magnetic layered materials (*e.g.*, CrI_3_, VSe_3_)^891,892^ with conclusions slightly different from those by using standard DFT. On the current efforts to develop a critical mass of knowledge in a fast-pace field, DFT remains the low-cost choice for most of the research groups working on 2D materials. In particular, for high-throughput screening investigations where thousands of systems are explored systematically through different work-flows, it is paramount to use methods that provide reliable and prompt results even though they are not at high accuracy. For instance, this approach can be used to find materials with Curie temperatures at room temperature.

**Figure 76:**
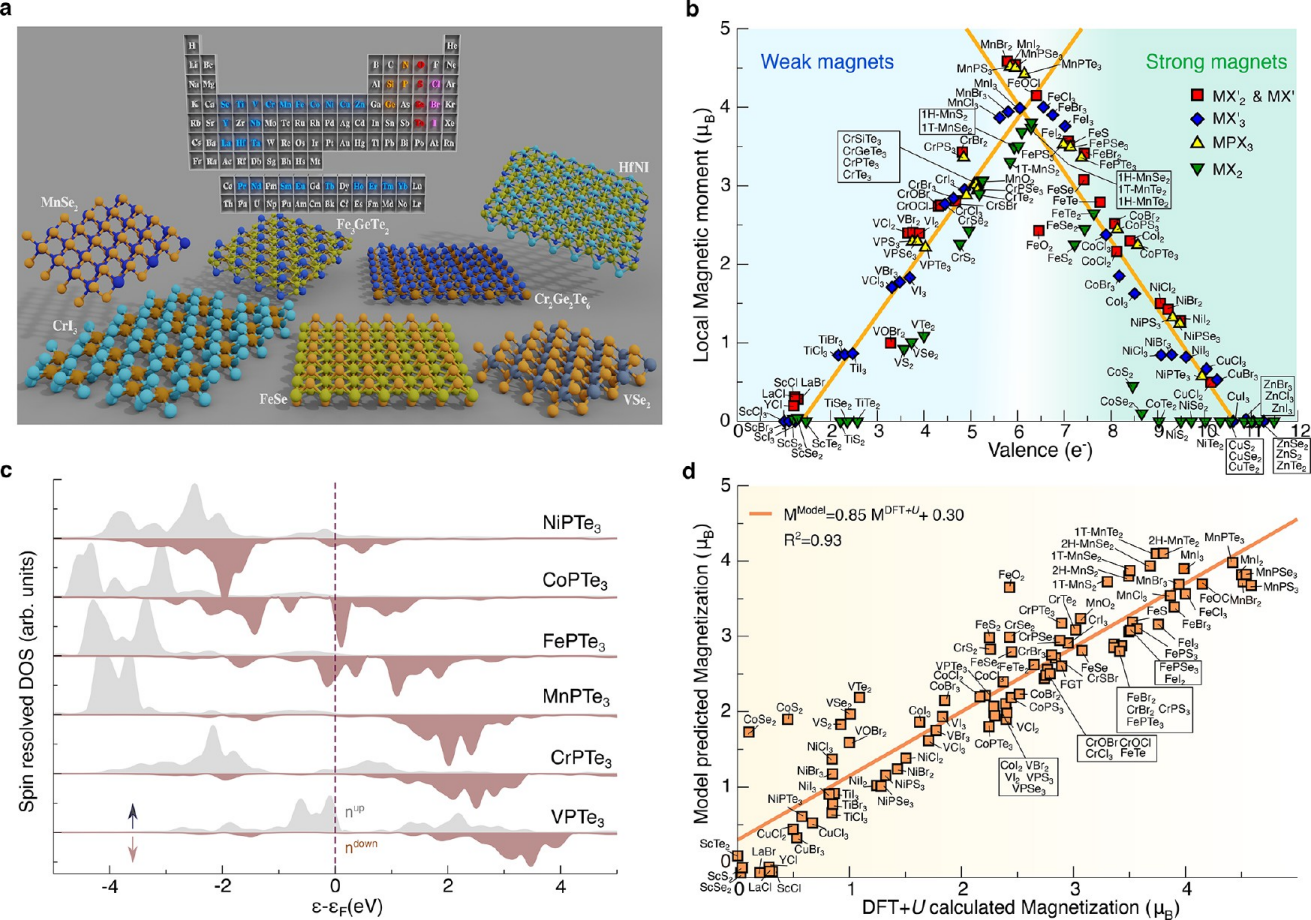
Slater-Pauling or volcano plot for 2D magnets. (a) High-throughput screening undertook over several crystal structures and elements of the periodic table including formulas MX′_2_, MX, MX′_3_, MPX_3_, MX_2_, and CrFTe_3_ with M = Sc–Zn, La, Y; X′=Cl, Br, I; X=O, S, Se, Te; F=Si, Ge. The simulations included mainly transition metals with 3d electrons, but some with 4d and 5d were included for comparison. (b) Variation of the local magnetic moment *M*(μ_B_) at the metal atom as function of its valence *Z*(e^–^). Bader charge analysis was used to extract *Z* for each metal atom at the compound. The solid lines show a fit to the data set on two different regimes according to the filling of the valence. The positive slope (weak magnets) can be fairly well fitted using *M*^+^ = 0.84*Z* – 1.15 (with a linear regression coefficient *R*^2^ = 0.96) and the negative (strong magnets) with *M*^–^ = −0.87*Z* + 9.27 (*R*^2^ = 0.90). An electron counting argument can be used to explain both regimes as discussed in the text. (c) Spin resolved density of states (DOS) for monolayer MPTe_3_ (M = V, Cr, Mn, Fe, Co, Ni) as function of the energy ε displaying the spin up density *n*^*up*^ (faint gray) and spin down *n*^*down*^ (faint brown) at opposite sides. The energy is shifted to the Fermi energy ε_*F*_ at zero. (d) Variation of the model predicted magnetization versus DFT + *U* calculated magnetization for the compounds showed in (a). Calculations were performed using the VASP code^884^ using a 21 × 21 × 1 *k*-sampling grid, the Dudarev (GGA+*U*) scheme^885^ with Hubbard *U* values following those in ref (341). The energy cutoff is set to 600 eV, the convergence criteria for energy to 10^–7^ eV and for the forces to 0.01 eV/Å. In order to avoid interactions between the layers, we applied periodic boundary conditions with a vacuum space of 25 Å. We used the projector augmented wave (PAW)^886^ methods with a plane wave basis. The Vosko–Wilk–Nusair modification scheme^887^ is applied for the spin-polarized calculations. All images in this figure are original, and no permissions are required.

Another interesting problem is the organization of the different 2D magnets that may be discovered or already exist in terms of simple descriptors. One approach is to organize the layers *via* the magnetic moment *M* and the valence charge *Z* of the transition metal in a so-called Slater–Pauling curve.^893,894^ This plot was successfully used in the past to study the magnetic properties of pure metals and alloy compounds (*i.e.*, Fe–V, Co–Cr, Fe–Pt)^894^ since it provides a generic picture in terms of a simple electron counting argument as discussed in the following. Moreover, in other fields such as in catalysis, the definition of volcano plots define species with high chemical activity for specific chemical reaction,^895^ which in the context of magnetism would be translated in compounds with high magnetic moments. Indeed, by using first-principle methods (see caption in Figure 76 for details) we show that despite of the crystal structure, elements considered and chemical formula (Figure 76a), the Slater–Pauling curve holds for 2D vdW magnetic materials (Figure 76b). The plot has two behaviors which can be used to distinguish the 2D magnets in two classes *via* the position of the ascending (positive slope) and descending (negative slope) branches:

- weak magnets: , ascending branch
- strong magnets: , descending branch

The positive (+) and negative (−) slopes of *M* can be well fitted by
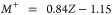
24
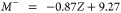
25with a linear regression coefficient of *R*^2^ = 0.96 and *R*^2^ = 0.90, respectively.

One of the main reasons for the two different gradients corresponds to the amount of filling of the *d*-band following the Hund’s first rule. The materials with  have their majority spin density of states (DOS) *n*^*up*^ being filled successively with the change of the metal atom until it is completely full. In this process the magnetization increases linearly with the additional charge reaching maximum values of *M* ∼ 4.5 μ_B_ for Mn-based 2D materials (*i.e.*, MnBr_2_, MnPX_3_ (X = S, Se, Te), MnI_2_). Once *n*^*up*^ is filled, the remaining electrons can only be added to minority spin states *n*^*down*^ leading to a decrease of *M* or . A nonmagnetic state is obtained as the spin-down band is fully saturated. This effect can be seen systematically, for instance, in MPTe_3_ (M = V, Cr, Mn, Fe, Co, Ni) compounds as we computed the spin resolved DOS displayed in Figure 76c. The terms weak and strong magnets are defined in terms of the filling of the 3d-band as the latter represents those with a full *n*^*up*^, while the former corresponds to an empty *n*^*down*^.

We also noticed that some compounds may have substantial charge transfer between the 3d-states at the transition metal and the sp electrons provided by the chalcogens or halides since the valence *Z* slightly shifted from the ideal valence for the 3*d*-shell atom. This indicates that depending on the nonmagnetic ion forming the 2D compound the local magnetization varies accordingly. However, by using eqs 24 and 25 we can still estimate with good accuracy relative to DFT+*U* calculations the value of the magnetization of any material just using *Z* as an input parameter (Figure 76d). We obtained an almost 1:1 comparison between model and DFT+*U* simulations (*M*^*model*^ = 0.85*M*^DFT+*U*^ + 0.30, *R*^2^ = 0.93) when we considered all computed materials. A better estimation with angular coefficient roughly of 1 between *M*^*model*^ and *M*^DFT+*U*^ can be extracted if the 2D magnets are separated by each corresponding families:

- *M*^*model*^ = 0.94*M*^DFT+*U*^ + 0.13, *R*^2^ = 0.98 for MX_2_ (M = V–Co; X = O, S, Se, Te).
- *M*^*model*^ = 0.93*M*^DFT+*U*^ + 0.20, *R*^2^ = 0.94 for MX_2_ (M = V, Mn, Fe, Co, Ni, Cu; X = Cl, Br, I).
- *M*^*model*^ = 0.90*M*^DFT+*U*^ + 0.27, *R*^2^ = 0.98 for MX_3_ (M = V–Cu; X = Cl, Br, I)
- *M*^*model*^ = 1.00*M*^DFT+*U*^ + 0.02, *R*^2^ = 0.98 for MPX_3_ (M = V–Ni; X = S, Se, Te)

It is worth mentioning that although the Slater–Pauling curve provides a simple but yet powerful tool to interpret a broad range of materials, the particular characteristics of each compound may influence its magnetic features. Such as whether a more itinerant component is present in which an atomic picture would be no longer valid. On that, additional theory in terms of the Stoner model^896,897^ or more sophisticated approaches would be required.^854^

### Future Challenges and Outlook

The field of 2D magnets presents an exciting opportunity to probe magnetism down to the atomic scale and over the coming years will provide continuous challenges to our theoretical understanding of magnetism. The ability of first-principles methods to make quantitative predictions of the fundamental interactions in 2D magnets is a triumph of modern computational physics that will help to guide their future discovery.^232,744,898−900^ Despite this, many challenges still remain in developing a deeper understanding of 2D magnets. At the electronic level, *ab initio* approaches often rely on pseudopotentials that must be carefully parametrized or include the right amount of core-states to ensure accurate results. vdW interactions that dominate the interlayer magnetic properties present a particular difficulty and more accurate approaches such as the inclusion of vdW parametrization on hybrid functionals may assist in higher accuracy on the computation of magnetic properties and exchange parameters. These ingredients are critical in the search for high Curie temperature 2D magnets and predicting the properties of functionalized materials^867,901^ and heterostructures.^868,902−904^ High-throughput calculations^796,890,905^ allow for rapid identification of suitable 2D materials for different applications, while machine learning may also assist in the automated searching of larger parameter spaces. One of the most exciting aspects of 2D magnets is the crossover with spintronics, providing means to probe and manipulate electron spins at the nanoscale. Spintronics is a rapidly developing area in its own right, but understanding the interactions of electrical currents and magnetic textures in 2D materials is a significant challenge both fundamentally in terms of spin transport but also computationally in being able to model experimentally accessible time and length scales.

For elevated temperatures, the current atomistic approach used within the 2D Heisenberg model has some basic approximations assuming a fully classical heat bath^865^ and fully localized magnetic moments. While the latter approximation seems to be reasonable for a broad class of current 2D magnets,^796^ it may be necessary to introduce longitudinal spin fluctuations^906−909^ to better describe the itinerant characteristics of local moments. An outstanding problem common to both 3D and 2D magnetic materials is the nature of the heat bath that drives thermal spin fluctuations and allows for energy dissipation. In metallic systems the conduction electrons play a critical role in mediating spin–lattice energy transfer and is reasonably well described by Langevin dynamics.^856^ In insulating systems the spin and lattice systems are directly coupled, with recent developments allowing explicit treatment of coupled spin and lattice degrees of freedom.^878^ Several popular vdW systems are either insulators or semiconductors which present a particular challenge where the lattice and electron degrees of freedom are likely to be important to the resulting spin dynamics. The other component is that all these approaches are classical in nature, and neglect the quantum nature of the heat bath and localized spin flip (Elliott–Yafet) scattering events.^910,911^ Spin temperature rescaling^31^ is a phenomenological approach that gives better ensemble agreement with experimental data, but additional approaches founded in quantum thermodynamics are needed to better describe the nature of the heat bath in magnetic systems.^872^ On that, additional developments are needed together with efficient computer implementations.

The next few years will be groundbreaking in evolving our understanding of magnetism at the 2D limit, where computational methods will play a leading role in this endeavor. Experimental data will challenge theory and its underlying assumptions, while modeling can explore unexpected physics and materials at low cost and high speed to guide experiments toward the cutting edge at different forefronts. Such juxtapositions are rare in scientific discovery. It is undoubtedly an exciting time in determining the fundamental nature of magnetic materials that will lay the foundations of pioneering technologies into the future.

## Perspectives and a Forward-Looking Approach

The last several years have seen significant advances in both the fundamental understanding and potential practical implementations of 2D magnetic materials on device platforms. It is important to recognize that this rapid development in science and engineering has been a driven by a focused effort by many research groups around the world. The result has been a flurry of science on the magnetism of atomically thin layers currently culminating in the emergence of truly transformational technologies with the potential to significantly alter the landscape for data storage, information processing and spintronics. A forward-looking approach to identify challenges to pursue and problems to solve will serve as a guideline for many scientists to enter the 2D world. In the following section, we have summarized some of the challenges discussed in this work that will help ensure fast progress of the field of 2D vdW magnetic materials.

### Fundamental Aspects

**Figure 77:**
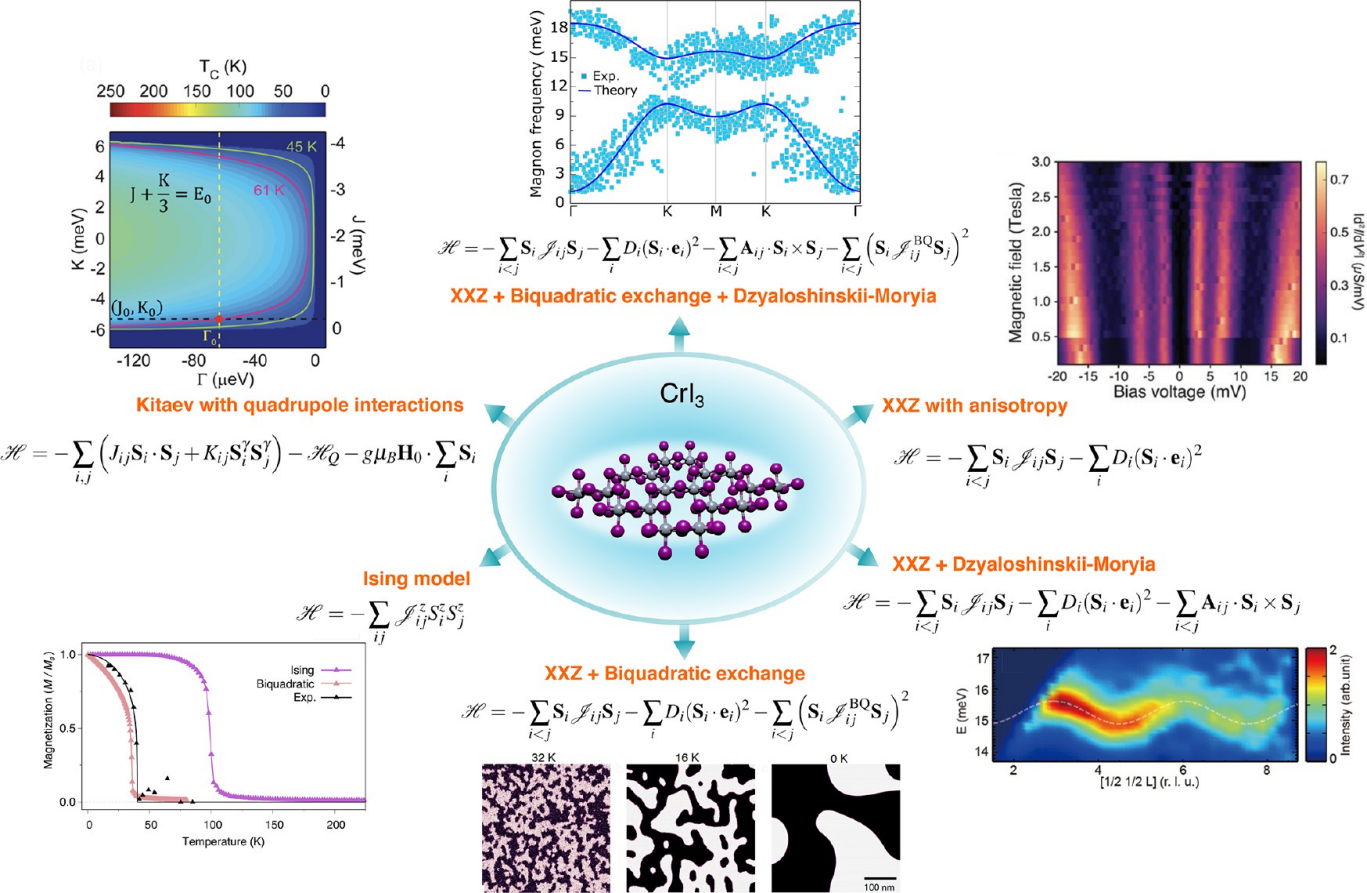
Comparison of spin Hamiltonians used to model the magnetic properties of CrI_**3**_. The two most commonly used Hamiltonians in the literature have been the Ising and the Heisenberg (XXZ) models which also includes magnetic anisotropy. The latter was used to understand inelastic tunnelling spectra for MTJs.^14^ The former was initially assigned to CrI_3_^5^ but its gross overestimation of the Curie temperature relative to experimental data made it unrealistic to account for the interactions in the system. Overall, depending on the property being measured, other alternatives have been considered: (i) for angle-dependent FM resonance measurements,^746^ the Kitaev model with quadrupole–quadrupole interactions and the Zeeman coupling was implemented; (ii) for inelastic neutron scattering to extract the magnon dispersion of bulk CrI_3_,^744,745^ the XXZ model either including DMI or adding biquadratic exchange with DMI^796^ have been proposed; and (iii) for the magnetic domains and domain walls on CrI_3_, a Hamiltonian taking into account biquadratic exchange was utilized.^31^ Starting with image at top, and going clockwise, panels adapted with permission under a Creative Commons CC BY license from refs (796). Copyright 2020 Springer Nature. Adapted with permission from ref (14). Copyright 2018 AAAS. Adapted with permission from ref (745). Copyright 2020 American Physical Society. Reproduce with permission from ref (31). Copyright 2021 John Wiley and Sons. Adapted with permission from ref (746). Copyright 2018 AAAs.

The description of 2D magnets using different spin Hamiltonians is clearly one of the fundamental aspects to be addressed in the forthcoming years. Several models have been used to date to describe the spin interactions of layered materials (see previous section on Theory and Simulations for additional details). We use CrI_3_ as an example of how different spin Hamiltonians have been implemented by different research groups to understand its properties (Figure 77). The importance of such relies on the predictive power of unforeseen phenomena using an archetypal spin model as well as understanding measured properties. For instance, the spin gap observed at the Dirac point in bulk CrI_3_ has actively been modeled by different Hamiltonians^744,745^ without a more conclusive picture until very recently with more refined measurements.^750^ This suggests that instrumental resolution is a key feature for measurements of the topological properties of vdW materials, which then provides high-resolution data for modeling. It is also worth mentioning the large variation of magnetic parameters (*e.g.*, exchange integrals, anisotropies, DMI, *etc.*) observed on the theoretical side. DFT of different varieties have been popular tools to compute them.^85^ However, most of the quantities important for parametrizing spin Hamiltonians are at the limit of the numerical libraries used to compile the software to carry out such tasks. That is, small variations on some input parameters (*e.g.*, *k*-sampling, energy cutoff, basis set, pseudopotentials, functionals, Hubbard-*U* value) on the codes may have large implications on the calculated quantities. This calls for a more careful analysis of the approaches used to extract magnetic parameters for 2D magnets to ensure reproducibility and consistent data across different research groups. A successful case has been applied in other contexts,^912^ which provides ideas that could be applied toward the standardization of modeling approaches.

Furthermore, an important underlying aspect to be studied is the emergence of complex magnetic-field-induced spin textures such as skyrmions and merons in layered systems displaying high Curie temperatures including Fe_3_GeTe_2_,^913,914^ Fe_5_GeTe_2_,^915^ or Co-doped Fe_5_GeTe_2_. The goal is to evaluate their potential for applications in, for example, magnetic skyrmions race track memories.^916,917^ Fe_3_GeTe_2_ has already been grown *via* MBE, while Co-doped Fe_5_GeTe_2_ has shown Curie temperatures above room temperature, suggesting intriguing prospects for the development of such memories. Spin textures characterized by spin chirality were found to affect the Berry phase of the charge carriers leading to a type of topological transport even at room temperature. Such topological transport, emerging at relatively low magnetic fields, can yield a sizable Nernst response that could be explored for thermoelectric applications. Initial results on heterostructures combining these compounds with topological insulators indicate a pronounced enhancement of their Curie temperature, suggesting a promising path to improve their performance in similar applications.

Another interesting challenge to be addressed is the practical validity of the famous Mermin–Wagner theorem^40,76^ on vdW materials. Even though the theorem has been one of the cornerstones of the field, past experience on graphene^918,919^ has taught us the opposite. It is well accepted nowadays that finite size ripples (*i.e.*, structural distortions) help to stabilize the intrinsic crystalline order at finite temperature in graphene. Similar arguments also apply for the majority of atomic layered materials found so far in the literature.^920^ However, if additional ingredients could be present to induce long-range magnetic order on 2D magnets without the need to fulfill the Mermin–Wagner theorem, *i.e.*, the dependence on magnetic anisotropies, it would be certainly a step forward to unanticipated fundamental physics and practical applications. Indeed, recent theoretical results^921^ have demonstrated that the applicability of the Mermin–Wagner theorem is far more limited than initially predicted. That is, only for large length scales, *i.e.*, near the diameter of the known universe (∼ 10^25^ m), 2D materials will display no net magnetic order at finite temperatures. This indicates that for implementations in real devices within the typical micrometer range, 2D magnets with no anisotropy constants could be used. Experimental validation of these predictions would be a leap forward for the exploration of a broad range of compounds with isotropic magnetic properties that potentially could be fabricated in 2D.

### Devices, Synthesis and Related Challenges

Some of the main challenges are (i) to find 2D magnets with *T*_*c*_ at or above room temperature, (ii) to develop large-scale synthesis methods that are able to produce good quality atomic layers over large areas, (iii) refinements in device fabrication and integration in current technologies, and (iv) chemically stable materials under environment conditions.

For (i), a few materials have recently been found or predicted to hold magnetism at promising temperature ranges, *e.g.*, 300–850 K. For (ii), two successful examples obtained by MBE growth^78,83^*via* MBE growth provide evidence that a pathway toward a bottom-up approach may be within reach in the lab. Moreover, CVD is a popular method that has been used for years to synthesize nonmagnetic 2D materials. Recent demonstrations on the synthesis of magnetic layers suggest that similar developments in CVD growth and fine-tuning of growth parameters to control material properties will be a crucial enabling step. Regarding (iii), the fabrication of devices with low-resistance contacts using device geometries needs to be developed for a broad usage of 2D magnets. Finally, for (iv), encapsulation *via* nonmagnetic materials (*e.g.*, hBN, polymers) as well as substrate supports can reduce fast oxidation and preserve pristine properties long enough for use in practical applications. A critical development will be to find encapsulation materials that can simultaneously enhance magnetic properties and preserve chemical stability while being compatible with device fabrication and processing.

### Magneto-Optics

A few challenges can be pointed out such as the relation between the birefringent effects and the total magnetization which is more challenging in 2D magnets than in their 3D counterparts (see Magneto-Optical Phenomena section). This calls for the combination of complementary techniques, *e.g.*, magneto-PL, for a full characterization of the magnetic features. Raman spectroscopy is also a reliable technique for characterization of FM and AF samples. However, the rapid degradation of devices after a few days of environmental exposure suggests that additional improvements would be welcome. If sample conditions can be finely controlled in optical environments, several phenomena can experimentally be probed. For instance, dynamic magnetization processes can be induced by ultrafast laser excitations down to the 2D limit. It is unclear how atomically thin layers behave on read and write operations at times required by the industry. High-frequency signals within the terahertz (THz) regime is also one of the challenging issues in the field. The flexibility and control of the magnetic properties of vdW materials which can be mechanically manipulated with monolayer accuracy provide a horizon for device developments. Indeed, the fifth-generation of common wireless communication systems operates in frequencies on the order of hundreds of GHz up toward THz.^922^ Therefore, magnetic components for wireless networks would have to be adapted to a high-frequency domain. Investigations on 2D magnets that would result in compact, low-dissipation, low-cost THz spintronic devices may change our perspectives on information and communication technologies.

### Imaging Approaches

In terms of techniques, two popular methods, MOKE and MCD, suffer of considerable signal-to-noise ratio. The signal level from vdW magnets typically results in low detection signal within the range of 10^–3^–10^–6^ (Magnetic Imaging section). The application of high magnetic fields on thin layers is also challenging due to interferences between the magnetic induction of the sample and stray fields which ramp up detection. Furthermore, SP-STM and MFM may need to be adapted to measure spin signals from magnetic insulating materials (Magnetic Imaging section). In terms of analysis, the image reconstruction for techniques that probe stray field maps (*e.g.*, NV magnetometry) seems the one that stands out the most. A better description of algorithms that may take into account different components of the magnetic field in order to produce real-space magnetization images is worth exploring. A comparison with atomistic simulations helps to give hints about the sample magnetic structure. However, the dependence of stray fields with the tip–sample region puts limitations on the technique resolution and exploration of more fine features, such as domain walls or spin-textures (see Magnetic Imaging section).

### Mechanical and Thermal Properties

The interplay between softness and magnetic properties still provides a substantial challenge on the measurements of mechanical and thermal features based conventional techniques such as using AFM to indent suspended 2D magnets (see Spin Excitations and Topological Properties section). Improving sample quality is a critical step in this goal since the presence of defects, grain-boundaries, and chemical stability all affect the properties. In addition, there is much room for exploration of magneto-mechanical phenomena that are largely unknown in 2D magnets. A number of opportunities in terms of (i) strain-induced magnetic phases, (ii) coupling between heat and magnetism *via* mechanical properties (*e.g.*, magnetocaloric effect), and (iii) development of magnetic actuators and sensors. Indeed, central quantities in heat management such as the thermal conductivity and expansion coefficients are yet to be determined. These ingredients are critical in miniaturized modern devices where magnetic layers may be integrated and have an active role. In these applications, how spin effects may influence phononic thermal transport and what type of SOC may be present on different materials and interfaces are topics of increasing interest.

### Spin Excitations and Topological Features

After the measurements of spin excitations on CrI_3_,^744,745,750^ and their implications on topological magnons on 2D vdW magnetic layers, several possibilities from a broad family of materials are potential candidates for exploration (see Heterostructures, Twisted Layers, and Interfaces section). Several difficulties need to be overcome, for example, quantum fluctuations in low dimensional magnets are particularly concerning since they may rule out the appearance of spin waves and obscure any topological behavior. Materials with spin-1/2 (*e.g.*, α-RuCl_3_) would be avoided since they display large quantum fluctuations.

**Figure 78:**
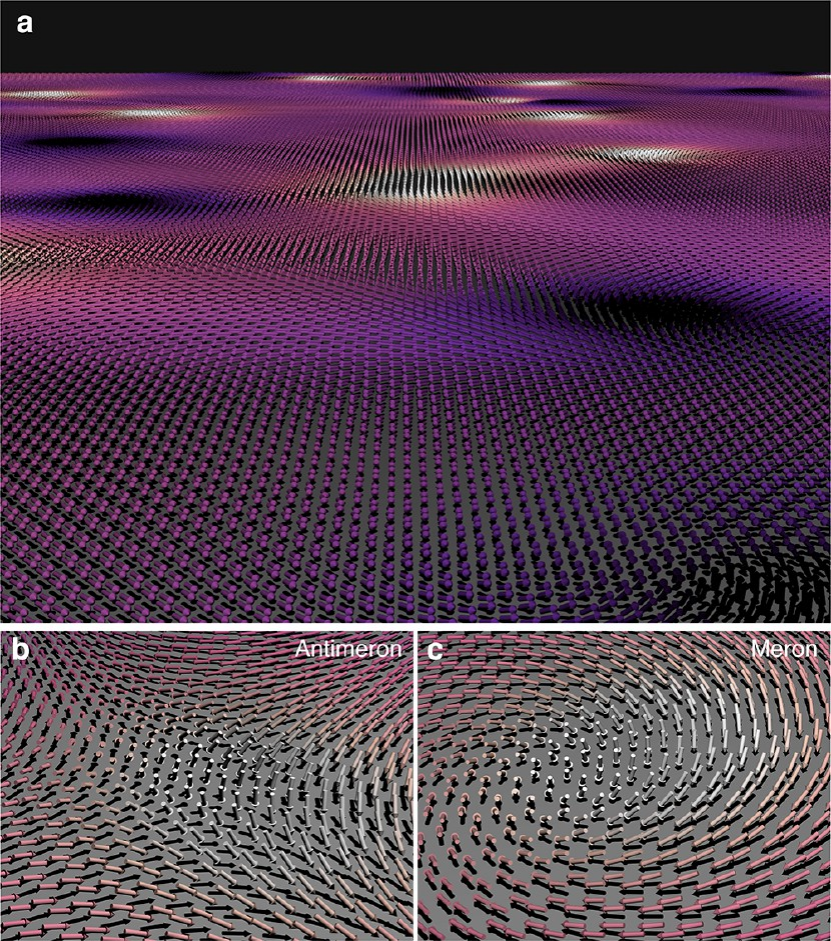
Merons and antimerons on 2D magnet CrCl_3_. (a) Artistic view of the presence of topologically nontrivial spin quasiparticles merons and antimerons on monolayer CrCl_3_.^876^ The different colors follow the orientation of the spins throughout the layer. (b, c) Local views of antimerons and merons, respectively, with their local spin configurations at Cr atoms.

The hunt for topologically nontrivial spin textures in 2D magnets (*e.g.*, skyrmions, merons, domain-walls, spirals, *etc*.) is also attracting substantial interest from both fundamental and applied directions (Figure 78). For the former, the stabilization of spin quasiparticles in strongly confined layers may provide the necessary platform for studying underlying interactions without disturbance from underlying substrates, such as in terms of two-body (*K*_*ijkl*_(**S**_*i*_·**S**_*j*_)^2^), three-body (*K*_*ijk*_(**S**_*i*_·**S**_*j*_)(**S**_*i*_·**S**_*k*_)) and four-body coupling terms (*K*_*ijkl*_(**S**_*i*_·**S**_*j*_)(**S**_*k*_·**S**_*l*_)), which may result in magnetic frustration, topological phases with anyonic excitations,^923^ and dynamics of rare quasiparticles.^876,914^ These phenomena can be studied at a level not yet achieved in more classical compounds. Furthermore, chiral magnetic interactions beyond DMI may lead to exotic noncollinear spin textures (*i.e.*, hedgehog, monopole) as recently predicted.^924^ If information can be stored in such spin-polarized structures, it may also be controlled in racetrack platforms^510^ through electrical stimulus. The difficulties ahead would be to find vdW materials with such characteristics where high velocities for the spin quasiparticle at low currents and when negligible pinning or decoherence effects are present. In this context, high-throughput screening using multiscale complementary theoretical techniques^31,925,926^ will be crucial.

### Spintronics

Devices based on spin will be a key area for 2D vdW magnets particularly in the transition from fundamental research to applications. Several challenges have been in the field for decades, which may now find potential solutions in 2D vdW magnets, as was described in more detail in the earlier section on spintronics. For instance, reduced dimensionality, control of magnetism *via* electrical means, enhanced thermal stability, sharp interfaces and transfer of the magnetic layers on different substrates are milestones toward a generation of spintronic devices with low energy consumption. One of the key ingredients would be the reduction of critical currents (below 2.0× 10^6^ A cm^–2^) in spin-transfer torque (STT) in information storage platforms, *e.g.* magnetic random-access memory (MRAM). At the moment little is known about STT phenomena in magnetic vdW layers and their interfaces. Currently, open questions in the field are surrounding (i) what substrates may enhance thermal stability and magnetic properties of 2D sheets, (ii) induce chiral interactions, and (iii) fast domain wall motion under STT current for high density memories and/or spin logic implementations. A few reports^445,925^ have appeared in the literature but additional exploration is urgently needed.

## Vocabulary

**2D vdW magnets**: a family of two-dimensional materials which display magnetic properties with a crystal structure of in-plane covalent bonding and weak interlayer (van der Waals) interactions
**Slater–Pauling curve or volcano plot for 2D vdW magnets**: a linear scaling relationship between magnetization and atomic number *via* a simple counting argument through the filling of the valence of *d*-shell of the metallic atom composing the material. Such plot passes through a maximum and decreases at low/high electron valence with approximately triangle-shape profile similarly as a volcano
**Spin waves**: collective excitations of the spin ordering which propagates along the magnetic lattice. Such excitations are incompatible with the Ising model given the continuous symmetry required. Spin waves are also known as magnons from a quasiparticle perspective
**2D magnetic genome**: a thorough revision of *the state of the art* achievements on the field of 2D magnetic materials providing a forward-looking approach on the main challenges to be delivered. Each included subject corresponds to a fundamental piece (“gene”) that would compose a large set of information (“genome”) needed to build that field and allow it to grow and develop promptly
**Meron**: topological nontrivial spin textures with a topological charge 1/2, which is one of the fundamental solutions of the Yang–Mills field equations
**Ising model**: simplistic spin model where only two discrete spin states (*i.e.*, up or down) are considered throughout the magnetic ordering. The model is appropriate for spin systems where the energy is invariant to reflecting every spin to its opposite orientation
**Heisenberg model**: continuous-symmetry-based spin model where spins can point anywhere in the unit sphere. In this model, the energy of a given spin configuration is invariant to rotating every spin in the same way around the unit sphere
